# Regioselective Substitution of BINOL

**DOI:** 10.1021/acs.chemrev.4c00132

**Published:** 2024-05-09

**Authors:** Lin Pu

**Affiliations:** Department of Chemistry, University of Virginia, Charlottesville, Virginia 22904, United States

## Abstract

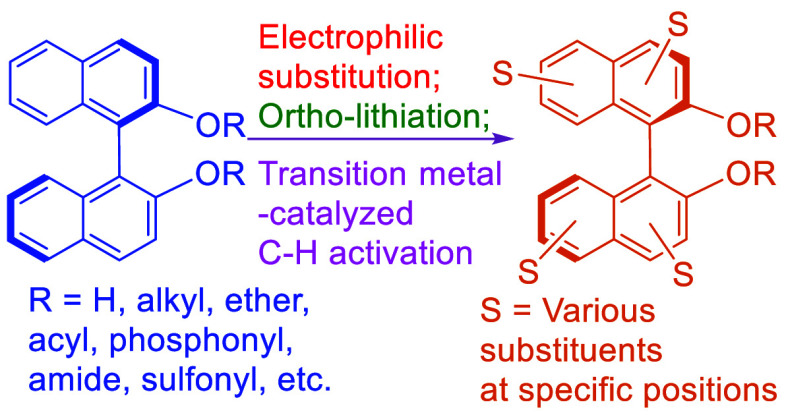

1,1′-Bi-2-naphthol (BINOL) has been extensively used as the chirality source in the fields of molecular recognition, asymmetric synthesis, and materials science. The direct electrophilic substitution at the aromatic rings of the optically active BINOL has been developed as one of the most convenient strategies to structurally modify BINOL for diverse applications. High regioselectivity has been achieved for the reaction of BINOL with electrophiles. Depending upon the reaction conditions and substitution patterns, various functional groups can be introduced to the specific positions, such as the 6-, 5-, 4-, and 3-positions, of BINOL. Ortho-lithiation at the 3-position directed by the functional groups at the 2-position of BINOL have been extensively used to prepare the 3- and 3,3′-substituted BINOLs. The use of transition metal-catalyzed C–H activation has also been explored to functionalize BINOL at the 3-, 4-, 5-, 6-, and 7-positions. These regioselective substitutions of BINOL have allowed the construction of tremendous amount of BINOL derivatives with fascinating structures and properties as reviewed in this article. Examples for the applications of the optically active BINOLs with varying substitutions in asymmetric catalysis, molecular recognition, chiral sensing and materials are also provided.

## 1 Introduction

1,1′-Bi-2-naphthol (BINOL) is a fascinating and versatile chiral molecule that has garnered enormous attention in the fields of chemistry and materials science. It represents a unique class of atropisomeric compounds characterized by two naphthol rings connected by a 1,1′-bond with restricted rotation which imparts stable chiral configuration. Many methods have been developed to either resolve the racemic mixture of BINOL into its two enantiomers,^1−16^ (*R*)- and (*S*)-BINOL, or directly prepare one of the enantiomers via asymmetric synthesis,^17−35^ leading to the optically active BINOL.

The optically active BINOL has also become commercially available in large scale. When the optically active BINOL in dioxane solution was heated at 100 °C for 24 h, no racemization was observed.^2^ The energy barrier for the racemization of BINOL was determined to be 37.8 kcal/mol by heating its diphenyl ether solution at 220 °C.^36^ Under strongly acidic or basic conditions, BINOL undergoes a faster racemization, but the alkyl ethers of BINOL possess further enhanced stability. For example, a macrocyclic crown ether derivative of BINOL showed no racemization when heated at 208 °C in diethylene glycol for 6 h.^2^

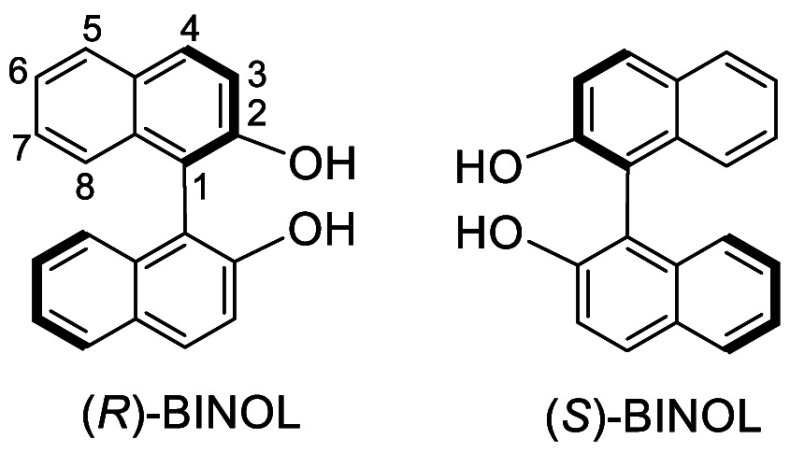



BINOL has exhibited many exceptional properties which have propelled it to the forefront of research in the realm of asymmetric synthesis, catalysis, sensing, and chiral materials.^37−50^ It has become indispensable for many chemists and other researchers striving to create intriguing molecular, supramolecular, and macromolecular structures with precise control over their stereochemistry. Optically active BINOL not only can be directly used to conduct many asymmetric catalytic processes in the presence of various metal complexes but has also served as the starting material for the preparation of a great number of chiral catalysts, molecular probes, polymers, dendrimers, metal–organic frameworks (MOFs), and covalent organic frameworks (COFs), etc.

The direct electrophilic substitutions of the optically active BINOL are among the most convenient ways to modify the structure of BINOL. Remarkable regioselectivity has been observed in these reactions which has allowed the selective introduction of functional groups at the 6-, 5-, 4-, and 3-positions for the construction of many appealing chiral structures for diverse applications. The ortho-lithiation of BINOL at the 3-position has allowed the introduction of a variety of functional groups at this position and has been extensively used in modifying the steric and electronic environment of asymmetric catalysts and molecular hosts.^51,52^ In recent years, transition metal-catalyzed C–H bond activation has also been utilized to functionalize BINOL at the 3-, 4-, 5-, 6-, and 7-positions with high regioselectivity. This paper will focus on reviewing these three types of regioselective substitution of the optically active BINOL. The closely related reactions of the racemic BINOL are also included. Examples of the optically active substituted BINOLs used in asymmetric synthesis, molecular recognition, chiral sensing, and materials are provided. Although substitutions of the hydroxy groups at the 2,2′-positions of BINOL with other functional units are also very useful to synthesize structurally modified BINOLs,^37−46^ they will not be discussed in this review.

## 2 Electrophilic Substitution at 6-Position

### 2.1 Dibromination

**Scheme 1:**
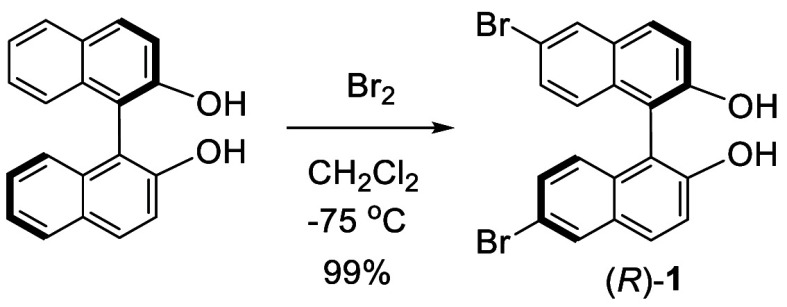
Bromination of (*R*)-BINOL

In 1979, Sogah and Cram reported that when (*R*)-BINOL was treated with 2.7 equiv bromine in CH_2_Cl_2_ at −75 °C for 2.5 h, (*R*)-6,6′-dibromoBINOL, (*R*)-**1**, was obtained in 99% yield (Scheme 1).^53^ When (*R*)- or (*S*)-BINOL dimethyl ether was reacted with bromine at −50 °C, the corresponding (*S*)-6,6′-dibromoBINOL dimethyl ether was obtained in 90% yield.

**Figure 1:**
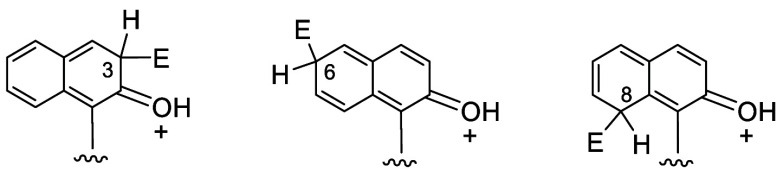
Resonance stabilization of electrophilic attack at the 3-, 6-, or 8-position of BINOL.

**Figure 2:**
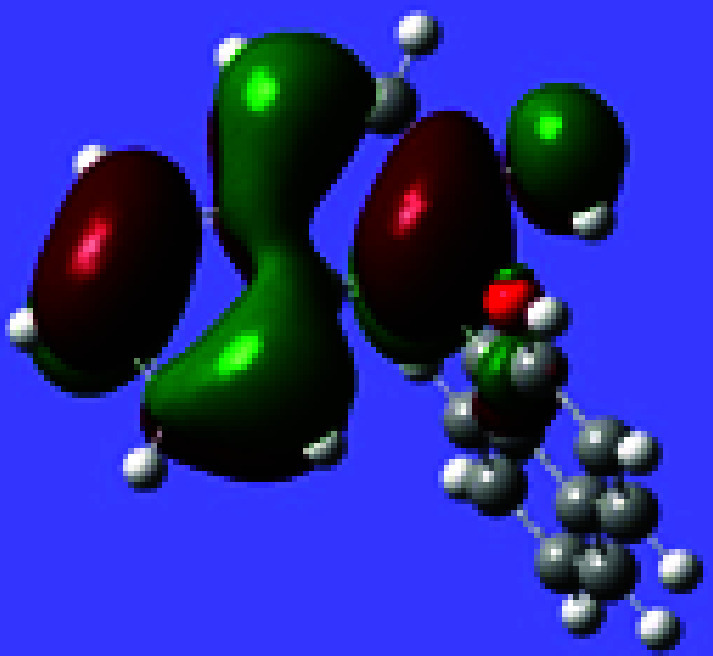
HOMO of (*S*)-BINOL.

In one of the naphthol rings of BINOL, although the electron-donating hydroxy group can provide resonance stabilization for the carbon cation intermediates formed by electrophilic attack at the positions 3, 6, and 8 as shown in Figure 1, only the 6-brominated product was obtained and no bromination at the 3- or 8-positions. Figure 2 gives the HOMO orbital of (*S*)-BINOL obtained by a density functional theory (DFT) calculation using the Gaussian program (B3LYP) with 6-31G basis set. It shows that a node plane goes through the 3-carbon of the naphthol ring, which should make electrophilic addition at this position unfavorable. The steric environment around the 8-position should hinder substitution at this position. Thus, a highly regioselective electrophilic substitution at the para 6-position is generally observed as the most favorable reaction, giving the 6,6′-dibrominated compound (*R*)-**1** as the predominated product.

**Figure 3:**
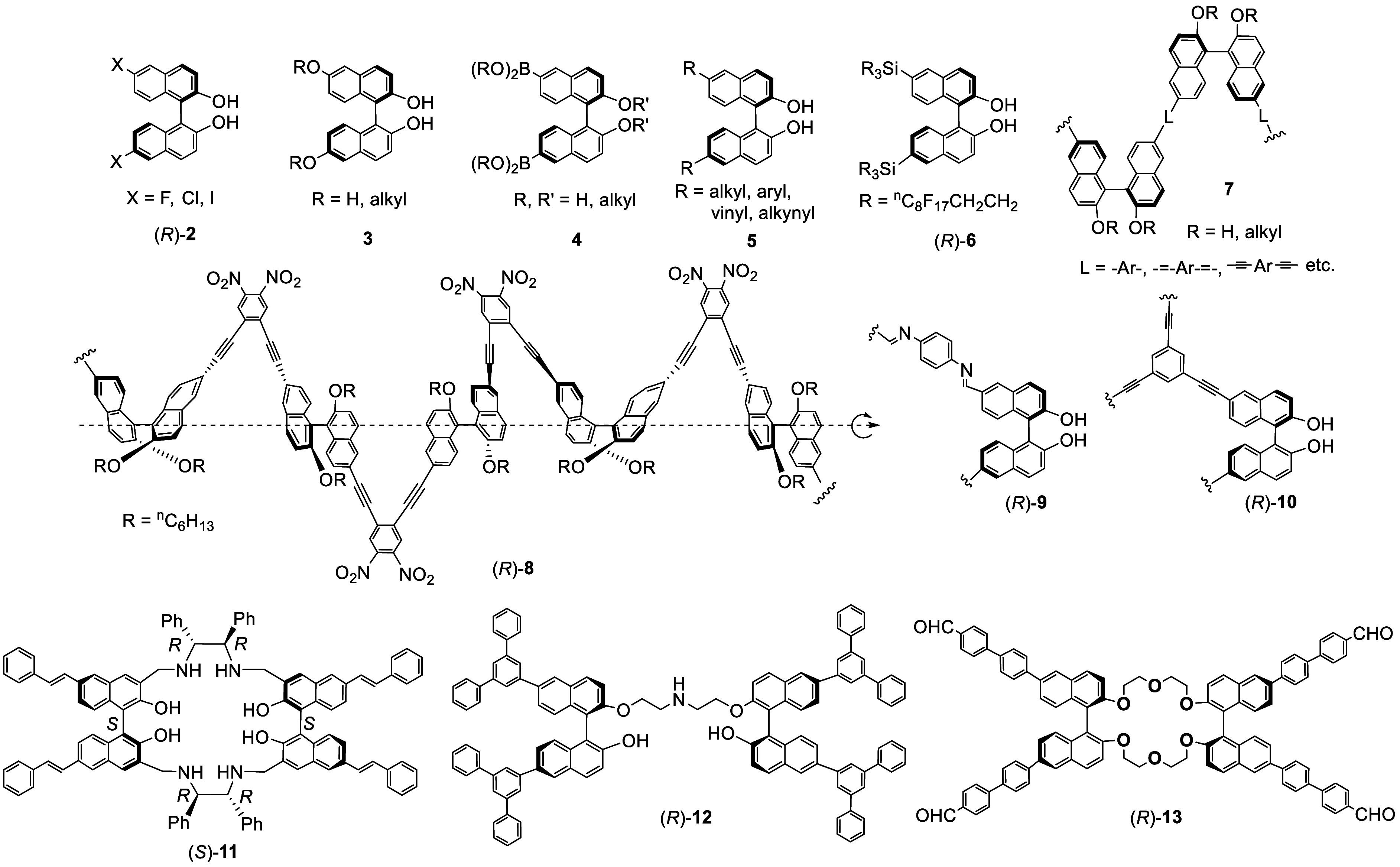
Examples of materials synthesized from 6,6′-dibrominated BINOLs.

The optically active 6,6′-Br_2_BINOL has allowed the synthesis of many other BINOL derivatives as represented by the examples in Figure 3. The bromine atoms of (*R*)-**1** were converted to other halogen atoms of compounds (*R*)-**2** by protection of the hydroxyl groups of (*R*)-**1**, followed by lithiation, halogenation with iodine, hexachloroethane, or *N*-fluorobenzenefulfonimide, and then deprotection.^54^ These compounds were used in combination with Zr(O^*t*^Bu)_4_ to catalyze the asymmetric Mannich-type reaction. Compound (*R*)-**3** (R = H) was prepared from the reaction of the dimethoxymethyl ether of (*R*)-**1** with ^*n*^BuLi, followed by addition of B(OMe)_3_ and H_2_O_2_ and then hydrolysis.^55^ It was used for asymmetric synthesis. Transition metal-catalyzed synthesis of compounds **3** from **1** were also developed.^56−58^ The 6,6′-di(boronic acid or ester)-substituted BINOLs **4** were synthesized as the precursors to prepare other derivatives of BINOL via the Suzuki coupling reactions.^59−67^ Compounds **5** with various alkyl, aryl, vinyl, and alkynyl substituents at the 6,6′-positions were prepared for a variety of study.^68−74^ The fluorous BINOL ligands such as the 6,6′-di(perfluoroalkylsilyl)BINOL (*R*)-**6** was synthesized for the biphasic (organic/fluorous) asymmetric catalysis.^75,76^ Other 6,6′-disilylBINOLs were also prepared for asymmetric catalysis.^77^

The main chain chiral conjugated polymers **7** were synthesized by the cross-coupling polymerization of (*R*)-/(*S*)-**1** or compounds **4** with various conjugated linkers.^60−63,78−80^ (*R*)-6,6′-DiethynylBINOL dihexyl ether was prepared from (*R*)-**1**, which was then polymerized with aryl dihalides to give the propeller-like helical polymers such as (*R*)-**8**.^81,82^ The applications of the BINOL-based chiral conjugated polymers in asymmetric catalysis,^83,84^ fluorescent sensing,^85,86^ nonlinear optics,^87,88^ circularly polarized luminescence,^89^ and others are explored.^90^ (*R*)-6,6′-DiformylBINOL, prepared from (*R*)-**1**, was used as a monomer to polymerize with aromatic diamines to form polymers such as (*R*)-**9**. These polymers in combination with Ti(O^*i*^Pr)_4_ were used as heterogeneous catalysts for the asymmetric reaction of ZnEt_2_ with aromatic aldehydes.^91^ Polymerization of the diacetate of (*R*)-**1** with 1,3,5-triethynylbenzene was used to prepare the chiral microporous polymer (*R*)-**10** for the enantioselective fluorescent recognition of chiral amino alcohols.^92^

(*S*)-6,6′-DistyrenylBINOL was used to prepare the macrocyclic compound (*S*)-**11**. This compound was used for the enantioselective fluorescent recognition of α-hydroxycarboxylic acids.^70^ A bisBINOL compound containing 6,6′-dendritic aryl groups, (*R*)-**12**, was used for enantioselective fluorescent recognition of mandelic acid with the fluorescent signal amplified by the dendritic aryl groups.^93^ The bisBINOL crown ether (*R*)-**13** with 6,6′-arylaldehyde groups was used to construct COFs by condensation with *para*-phenylenediacetonitrile, and the resulting material was used for the enantioselective fluorescent recognition of chiral amino alcohols.^94^

3,3′-Substituted BINOLs, prepared by ortho-lithiations of the hydroxyl protected derivatives of BINOL followed by further conversions (see section 7), were also directly reacted with bromine to give the 6,6′-dibromo products^95−106^ such as (*R*)-**14a**,^102^ (*R*)-**14b**,^103^ and (*S*)-**15**.^104−106^ Neither the electron-donating groups nor the electron-withdrawing groups at the 3,3′-positions of BINOL have changed the bromination selectivity at the 6,6′-positions. This indicates that the 2,2′-electron-donating oxygen atoms dominate the selectivity. Compounds (*R*)-**14a**,**b** were used as ligands in combination with metal complexes to catalyze the asymmetric hetero-Diels–Alder reaction and alkyne addition to aldehydes.^102,103^ Compound (*S*)-**15** was used to prepare the polymer-based fluorescent sensors such as (*S*)-**16**^104^ and (*S*)-**17**^105,106^ for the enantioselective recognition of chiral amino alcohols and amino acids.
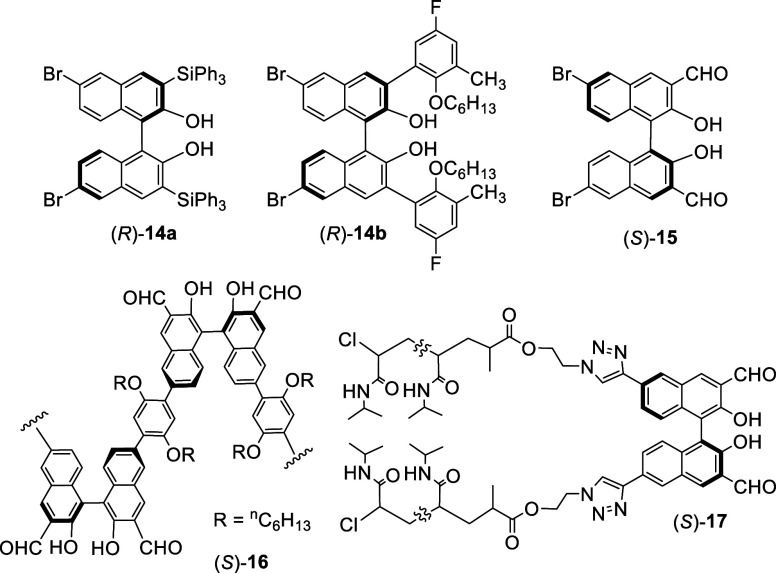


**Scheme 2:**
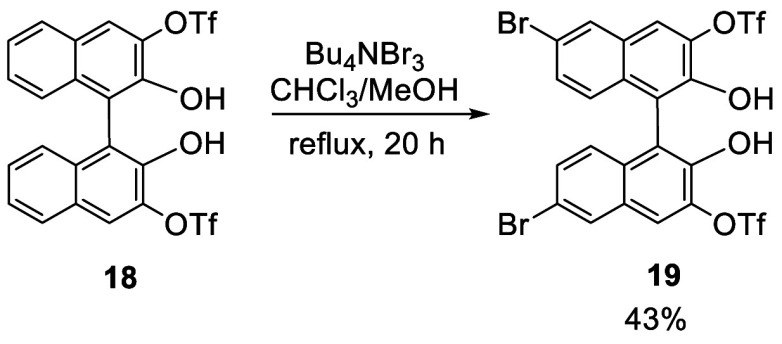
Bromination with Bu_4_NBr_3_

Tetrabutylammonium tribromide (TBATB) was used for bromination of a 3,3′-ditriflate substituted BINOL (Scheme 2).^107^ When racemic **18** was heated with TBATB in a mixed solvent of chloroform and methanol at reflux for 20 h, the 6,6′-dibrominated product **19** was obtained in 43% yield. This compound was used to prepare bowl-shaped cyclophanes in the construction of proton-responsive supramolecular polymers.

**Scheme 3:**
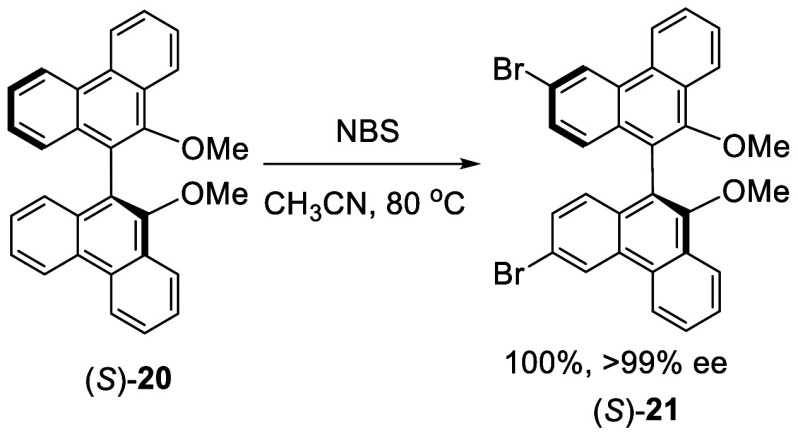
Bromination with NBS

*N*-Bromosuccinimide (NBS) was used for the bromination of a BINOL analogue, the dimethyl ether of biphenanthrol (*S*)-**20** (Scheme 3).^108^ When (*S*)-**20** was treated with 5 equiv of NBS in refluxing acetonitrile for 2 h, the dibrominated product (*S*)-**21** was obtained in quantitative yield with >99% ee.

### 2.2 Monobromination

Monobromination of (*R*)-BINOL by reaction with 0.5 equiv bromine in CH_2_Cl_2_ at −78 °C gave (*R*)-**22** in 47% yield.^109^ Compound (*R*)-**22** was converted to the styrene-based monomer (*R*)-**23**, which was subjected to free radical polymerization to generate a polymeric BINOL. The use of the polymer in the asymmetric ZnEt_2_ addition to aldehydes and the asymmetric Michael addition was studied.^109^ Monobromination of (*R*)-BINOL dimethyl ether also occurred in 40% yield by reaction with 1 equiv of bromine in CH_2_Cl_2_ at −78 °C.^110^
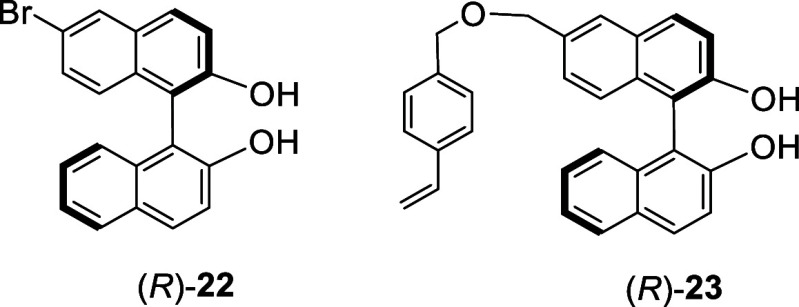


**Scheme 4:**
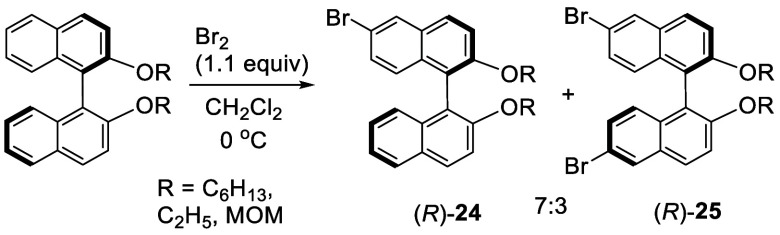
Bromination to Give a Mixture of Mono- and Dibromo-BINOLs

Reaction of (*R*)-BINOL alkyl ethers with 1.1 equiv of Br_2_ (diluted in CH_2_Cl_2_ and slowly added) at 0 °C gave a mixture of the monobromo product (*R*)-**24** (70%) and the dibromo product (*R*)-**25** (30%) (Scheme 4).^111^ This mixture was directly used for the subsequent reaction without separation.

**Scheme 5:**
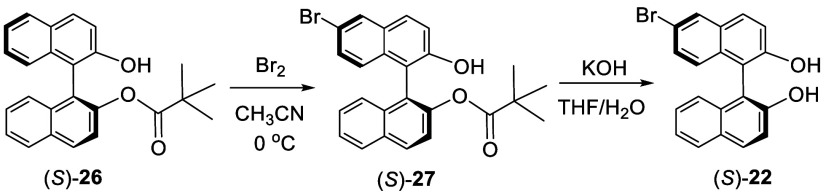
Selective Monobromination

In order to obtain the monobromoBINOL in high yield and avoid the separation from the dibromo product and the unbrominated starting material, one strategy was developed by converting BINOL to a bulkyl monoester, BINOL monopivalate (**26**), to deactivate one of the 6-positions followed by bromination at 0 °C in acetonitrile to selectively form **27** (Scheme 5).^112,113^ Hydrolysis of **27** gave the optically active monobromoBINOL **22** in quantitative yield.^113^ Cai reported the racemic version of this reaction earlier,^112^ and Uozumi reported the preparation of the optically active (*S*)-**22** from (*S*)-**26**.^113^

The monobromoBINOLs were used to prepare a variety of BINOL derivatives,^114−118^ including many polymer- or solid-supported BINOLs. For example, the Suzuki coupling of (*S*)- or (*R*)-**22** with polystyrene (PS) beads functionalized with boronic acid gave the PS-supported BINOL (*S*)- or (*R*)-**28**, which was used to catalyze the asymmetric oxidation of thioethers.^114^ (*S*)-BINOL was also immobilized on carbon nanotubes to form hybrid materials such as (*S*)-**29** for asymmetric catalysis.^117^ (*R*)-BINOL was linked to silica gel to form (*R*)-**30** as a chiral stationary phase for HPLC analysis.^118^
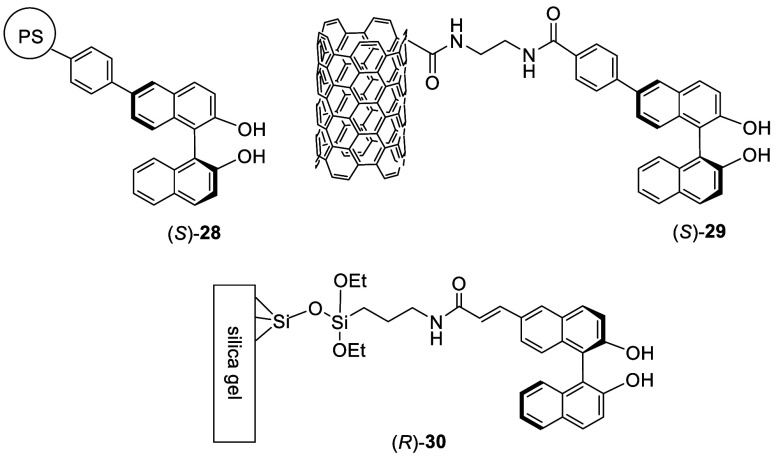


### 2.3 Iodonation

**Scheme 6:**
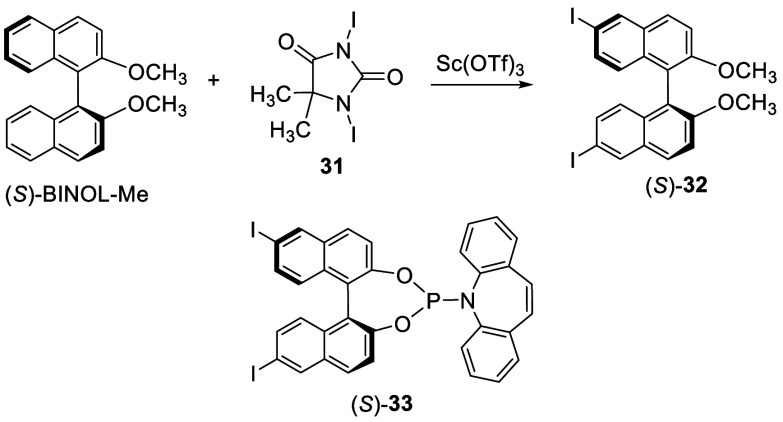
Iodonation of (*S*)-BINOL-Me

1,3-Diiodo-5,5-dimethylhydantoin (**31**) (2.0 equiv) in the presence of Sc(OTf)_3_ (10 mol %) was found to be an efficient iodonation agent for BINOL, which converted (*S*)-BINOL dimethyl ether [(*S*)-BINOL-Me] to (*S*)-**32** in 98% yield at room temperature over 12 h (Scheme 6).^119^ The optically active 6,6′-diiodoBINOL was used to prepare chiral phosphoramidite ligands such as (*S*)-**33** for the Ir-catalyzed asymmetric allylic substitution.^120^

**Scheme 7:**
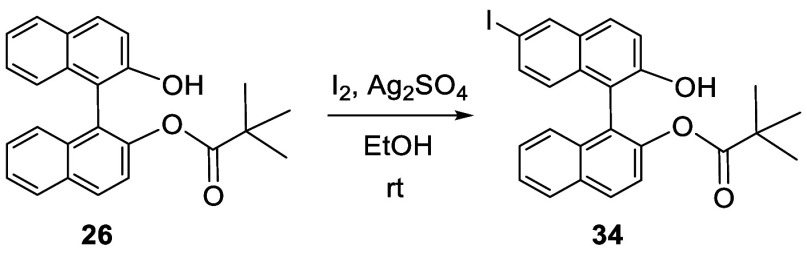
Selective Monodiodonation

When BINOL monopivalate **26** was treated with Ag_2_SO_4_ and I_2_ in ethanol at room temperature, the 6-iodo product **34** was obtained in 95% yield (Scheme 7).^121,122^ This compound led to the synthesis of a variety of 6-substituted BINOL derivatives via transition metal-catalyzed cross-coupling reactions and showed better reactivity than the corresponding bromo analogues.

### 2.4 Nitration

**Scheme 8:**
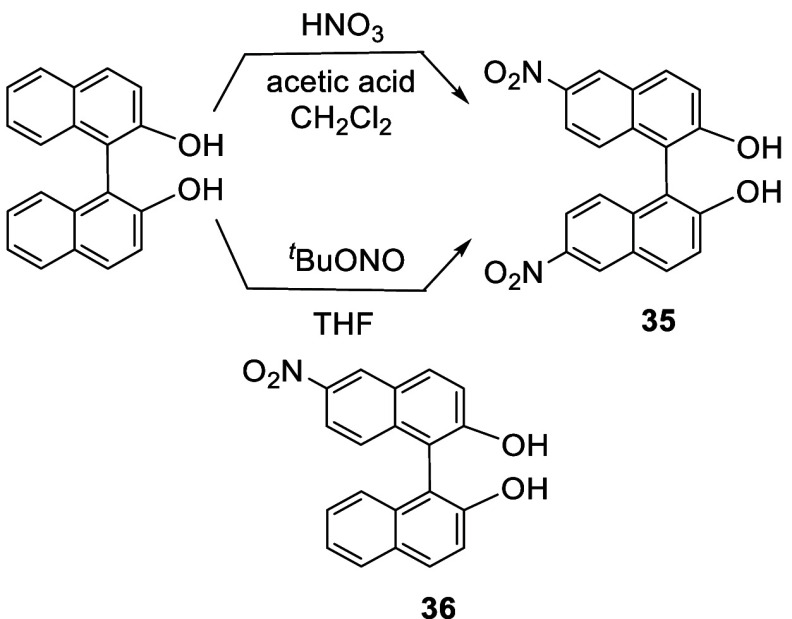
Nitration of BINOL

When racemic BINOL in acetic acid/CH_2_Cl_2_ solution was treated with nitric acid at room temperature for 16 h, 6,6′-dinitroBINOL (**35**) was obtained in 74% yield (Scheme 8).^123^ The nitro groups were converted to amine groups by hydrogenation of the dimethyl ether of **35**. Compound **35** was also obtained in 61% yield by reacting BINOL with ^*t*^BuONO in THF at room temperature (Scheme 8).^124^ 6-NitroBINOL (**36**) was obtained in 62% yield from the reaction of BINOL with concentrated nitric acid in dioxane at room temperature.^125^

The 6,6′-diamine compound (S)-**37** was obtained from the hydrogenation of the dimethyl ether of (*S*)-**35**, which was then polymerized with a dialdehyde linker to give a polymer **38**.^126^ This polymer showed fluorescence enhancement in the presence of F^–^.
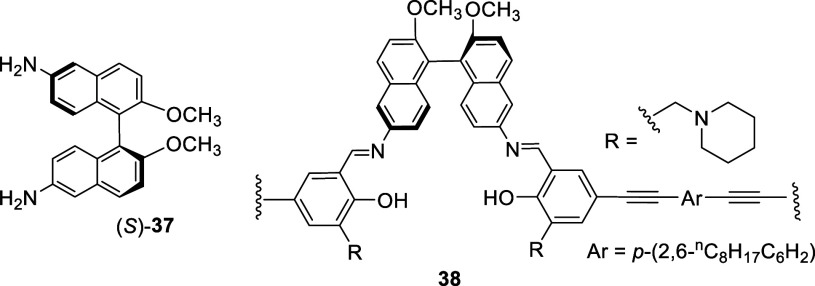


Nitration of a (*R*)-3,3′-diarylBINOL with fuming nitric acid in CH_2_Cl_2_ gave (*R*)-**39** in 78% yield. This compound was used to prepare the corresponding phosphoric acid (*R*)-**40** to catalyze the kinetic resolution of secondary alcohols.^127^ Other similar compounds were also prepared for asymmetric catalysis.^128−130,100^
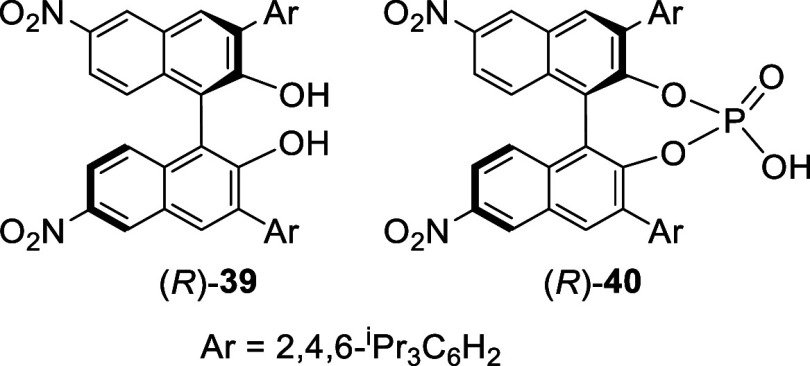


**Scheme 9:**
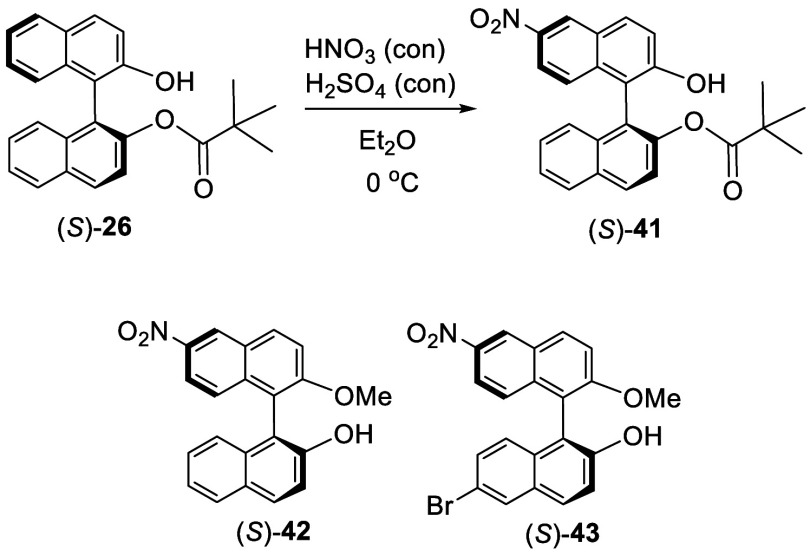
Selective Mononitration

Reaction of (*S*)-BINOL monopivalate (*S*)-**26** with HNO_3_(conc)/H_2_SO_4_(conc) in ether at 0 °C gave the mononitro compound (*S*)-**41** in 98% yield (Scheme 9).^113^ Methylation of (*S*)-**41** followed by KOH promoted hydrolysis gave compound (*S*)-**42**. Reaction of (*S*)-**42** with Br_2_ in acetonitrile solution gave (*S*)-**43** in 100% yield. In (*S*)-**43**, two different substituents were introduced to the 6,6′-positions of BINOL. Grinding the solid mixture of **26** with AgNO_3_ and I_2_ gave **41** in 57% yield.^121^

**Scheme 10:**
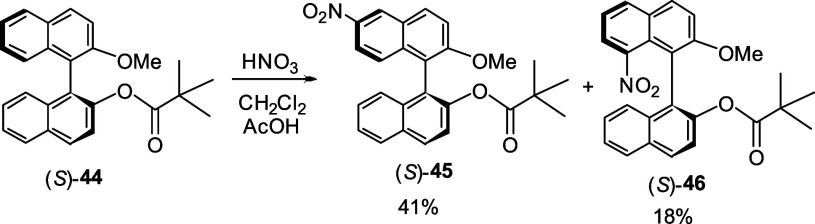
Nitration to Give 6-Nitro and 8-Nitro BINOL Products

When the optically active (*S*)-**44** was treated with fuming nitric acid in CH_2_Cl_2_/acetic acid, a mixture of 6-nitro and 8-nitro products (*S*)-**45** and (*S*)-**46** were obtained (Scheme 10).^131^ These compounds were characterized by X-ray analyses.

### 2.5 Sulfonation or Thiolation

**Scheme 11:**
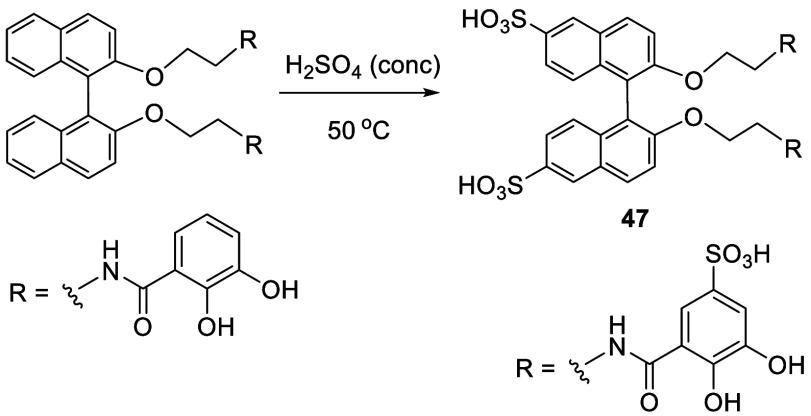
Sulfonation of a BINOL Alkyl Ether

Treatment of a BINOL dialkyl ether with concentrated sulfuric acid at 50 °C for 16 h gave the 6,6′-disulfonic acid BINOL **47** in 75% yield (Scheme 11).^132^ In this reaction, two additional sulfonic acid groups were also introduced to the R groups. This compound showed significant affinity for uranyl ion.

**Scheme 12:**
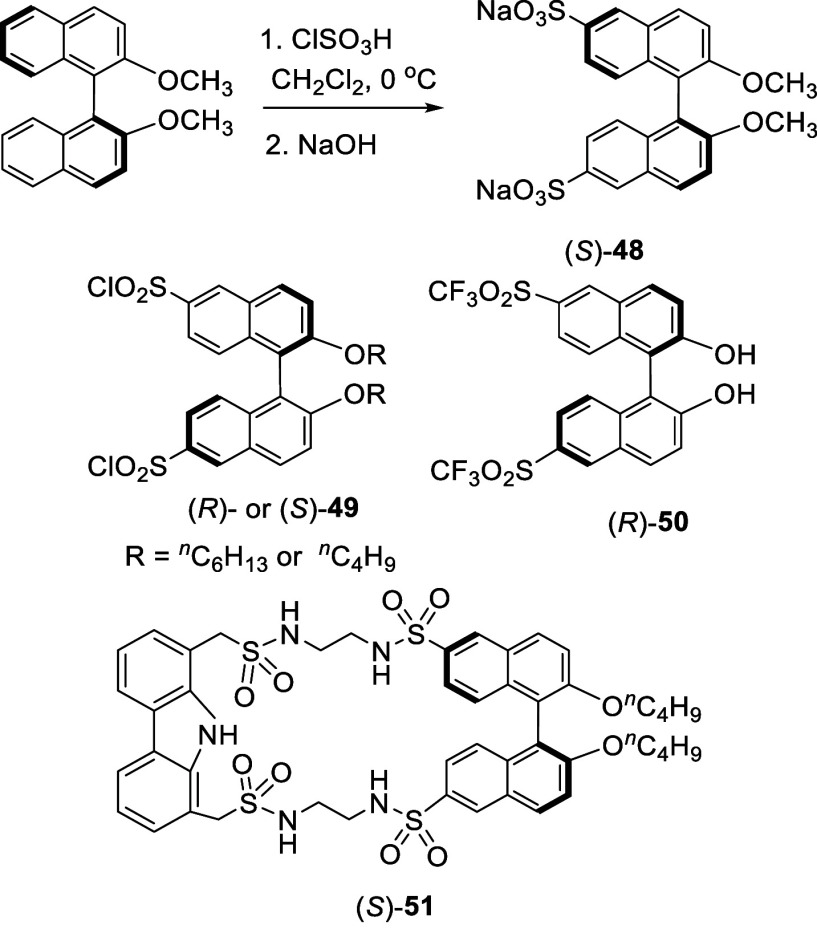
Sulfonation of (*S*)-BINOL-Me with ClSO_3_H

Chlorosulfonic acid (2.4 equiv) was also used as the sulfonation agent to react with (*S*)-BINOL-Me in CH_2_Cl_2_ at 0 °C for 7 h (Scheme 12). After treatment with NaOH, (*S*)-**48** was obtained in 85% yield.^133^ (*S*)-**48** was used to form an ionic polymer with a chiral quaternary ammonium salt to catalyze the asymmetric alkylation of a glycine ester. Reaction of (*R*)-BINOL dihexyl ether with chlorosulfonic acid (12 equiv) in CHCl_3_ at 0 °C to room temperature gave the 6,6′-dichlorosulfonyl product (*R*)-**49** in 40% yield.^134^ (*R*)-**49** was then converted to (*R*)-**50** to catalyze asymmetric reactions such as an alkyne addition to aldehydes, a Zr-based Mannich-type reaction and an organocatalyzed Morita–Baylis–Hillman transformation. The macrocycle (*S*)-**51** was synthesized from (*S*)-**49** (R = ^*n*^C_4_H_9_) and used for enantioselective extraction of phenylglycine from aqueous solution to chloroform.^135^

**Scheme 13:**
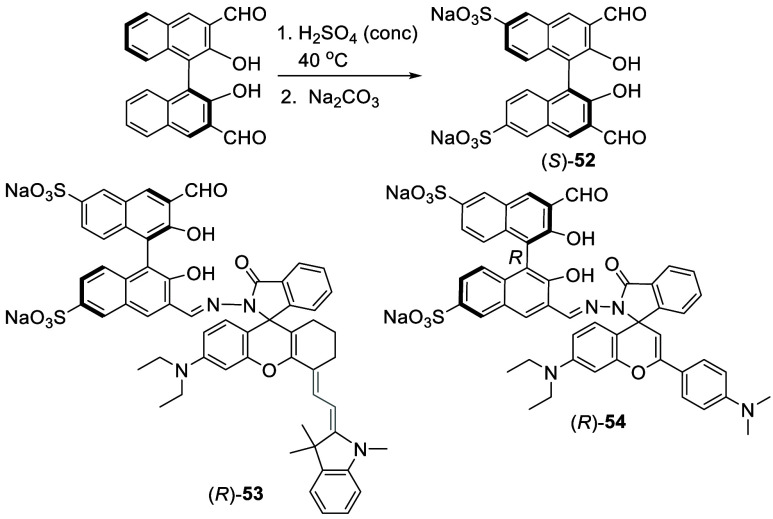
Sulfonation of 3,3′-DiformylBINOL

When (*S*)- or (*R*)-3,3′-diformylBINOL was treated with concentrated sulfuric acid at 40 °C for 18 h followed by addition of Na_2_CO_3_, compound (*S*)- or (*R*)-**52** was obtained in 24% yield (Scheme 13).^136^ (*S*)- or (*R*)-**52** was used as a water-soluble fluorescent probe for the enantioselective recognition of amino acids in aqueous solution. Compound (*R*)-**53** with emission >730 nm, obtained from the condensation of (*S*)-**52** with a rhodamine derivative, was used as a near-IR fluorescent probe for enantioselective recognition of amino acids.^137^ Combination of the green light emitting probe (*S*)-**52** with the red light emitting probe (*R*)-**54** allowed visual quantification of amino acid enantiomeric composition.^138^

**Scheme 14:**
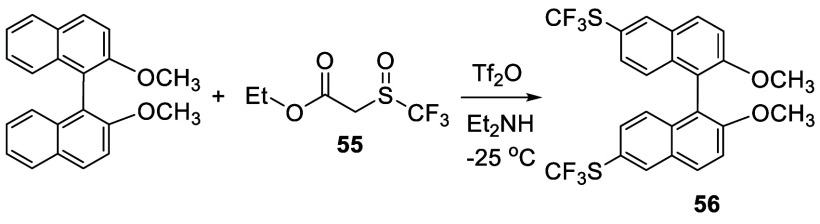
Trifluoromethyl Thiolation of BINOL-Me

Trifluoromethyl sulfoxide **55** was used as a trifluoromethyl thiolation agent to react with BINOL-Me at −25 °C to room temperature in the presence of Tf_2_O and Et_2_NH to give the 6,6′-dithiolated product **56** in 28% yield (Scheme 14).^139^

**Scheme 15:**
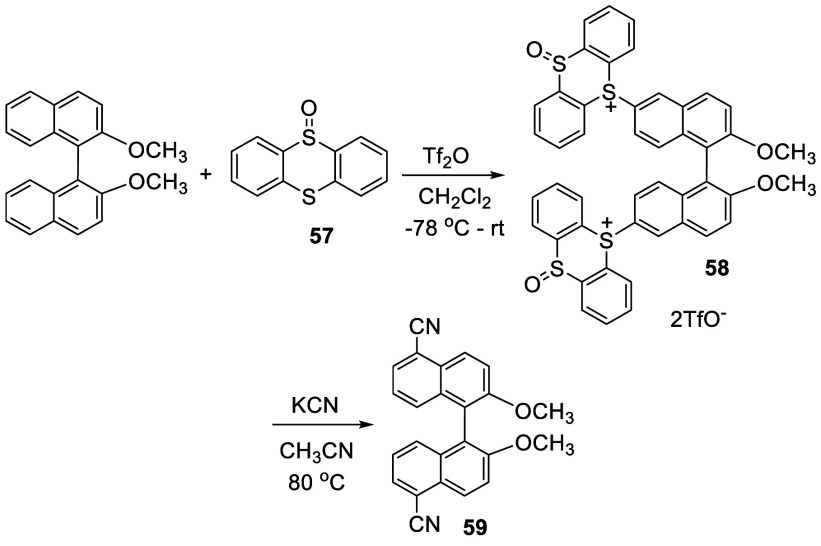
Reaction of BINOL-Me with Thianthrene-*S*-oxide

Thianthrene-*S*-oxide **57** was used to react with BINOL-Me in the presence of Tf_2_O in CH_2_Cl_2_ at −78 °C to room temperature which gave the 6,6′-disubstituted product **58** in 45% yield (Scheme 15).^140^ Treatment of **58** with KCN in acetonitrile at 80 °C for 12 h, a nucleophilic *cine*-substitution occurred to give the 5,5′-dicyano product **59** in 51% yield. Thus, this reaction allows the 6,6′-substituion of BINOL to be used to selectively functionalize the 5,5′-positions.

### 2.6 Alkylation

**Scheme 16:**
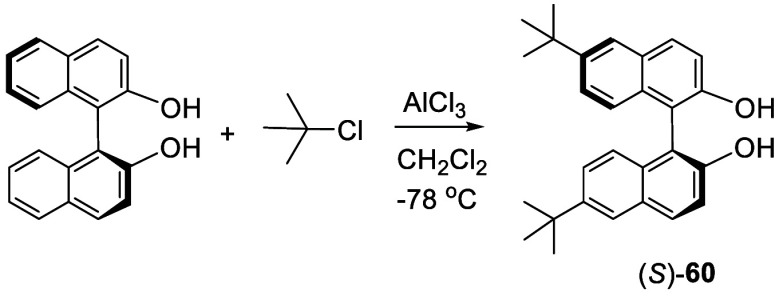
Friedel–Crafts Alkylation of BINOL

When (*R*)- or (*S*)-BINOL was treated with ^*t*^BuCl (8 equiv) in the presence of AlCl_3_ (1.6 equiv) in CH_2_Cl_2_ at −78 °C for 12 h, the pure 6,6′-^*t*^Bu_2_BINOL (*R*)- or (*S*)-**60** was obtained in ∼80% yield after hexane extraction (Scheme 16).^141^ NMR and X-ray analyses established the structure of this compound. This compound was used to prepare various phosphite and phosphoramidite derivatives to catalyze the asymmetric reduction of ketones. When the reaction of BINOL with ^*t*^BuCl in the presence of AlCl_3_ was conducted at 0 °C or room temperature, black materials were generated.

**Scheme 17:**
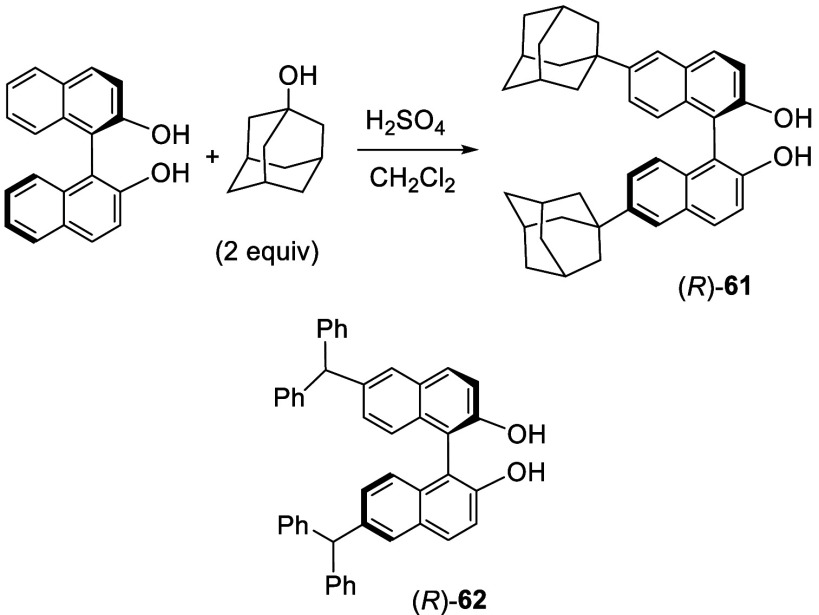
Sulfuric Acid Promoted Reaction of BINOL with 1-Adamantanol

Reaction of (*R*)-BINOL with 2 equiv of 1-adamantanol in CH_2_Cl_2_ in the presence of concentrated sulfuric acid gave a product in 60% yield, which was initially reported as a 3,3′-disubstituted compound^142^ and was later corrected as the 6,6′-di(adamantan-1-yl)BINOL (*R*)-**61** on the basis of a single crystal X-ray analysis (Scheme 17).^143^ This compound was converted to the corresponding chiral phosphoric acid to catalyze the asymmetric conjugated hydrocyanation of aromatic enones. A similar synthesis of (*R*)-**61** was later reported in 95% yield.^144^ Compound (*R*)-**62** was obtained in the same way by using diphenylmethanol in place of 1-adamantanol, and it was used to prepare catalysts for tandem Michael addition/enantioselective Conia-ene cyclization.^145^

**Scheme 18:**
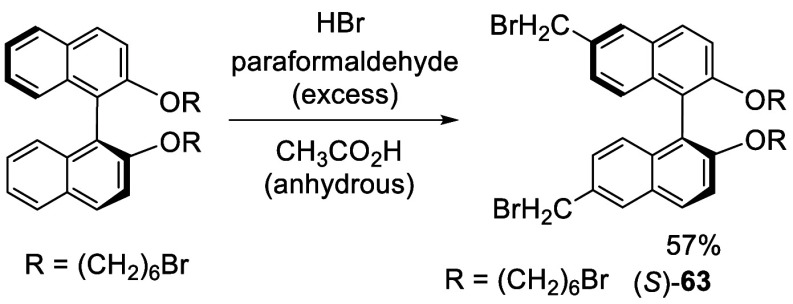
Bromomethylation of a BINOL Alkyl Ether

The 6,6′-dibromomethylBINOL dialkyl ether (*S*)-**63** was synthesized from the reaction of a BINOL alkyl ether with a large excess amount of paraformaldehyde and HBr in glacial acetic acid at room temperature for 36 h (Scheme 18).^146^ This compound was applied for chiral molecular switch study.

### 2.7 Acylation

**Scheme 19:**
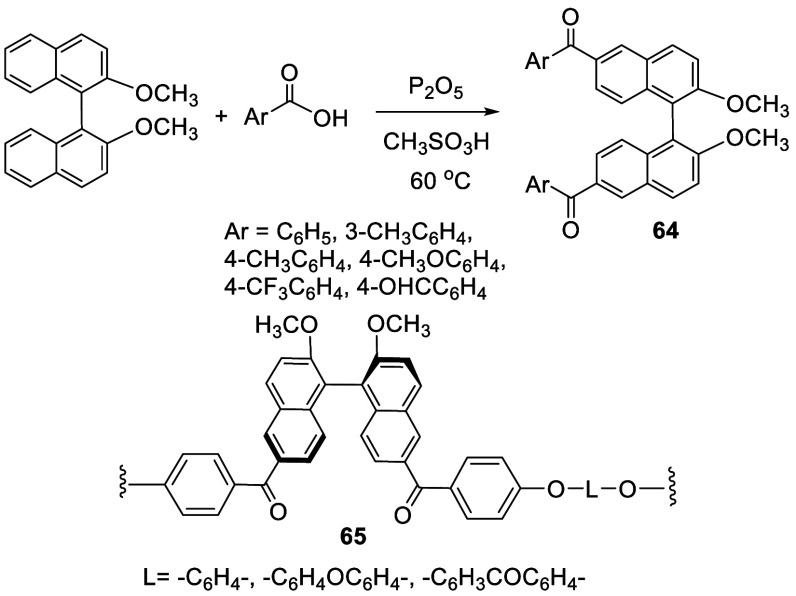
Acylation of BINOL-Me with Aryl Carboxylic Acids

Aryl carboxylic acids were used for the 6,6′-diacylation of BINOL-Me in the presence of P_2_O_5_ and methanesulfonic acid. The reactions were conducted at room temperature or 60 °C over 24 h to give the aryl ketones **64** in 71–100% yields (Scheme 19).^147^ Chiral polyaromatic ketones **65** were synthesized from the polymerization of (*S*)-**64** (Ar = 4–F-C_6_H_4_) with diols in the presence of K_2_CO_3_.^148^ Fluorescence properties of similar BINOL-based polyketones were studied.^149^

**Scheme 20:**
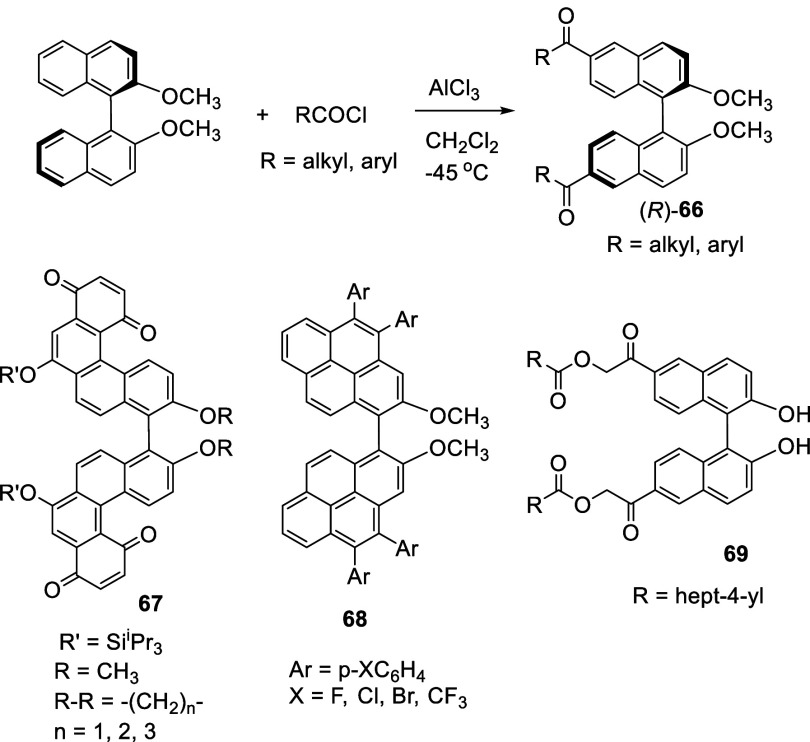
Acylation with Acyl Chlorides

Alkyl and aryl acyl chlorides were used to react with (*R*)-BINOL-Me in the presence of anhydrous AlCl_3_ in CH_2_Cl_2_ at −45 °C to room temperature over 3–8 h to give the 6,6′-diacyl compound (*R*)-**66** in 68–87% yields (Scheme 20).^150^ These BINOL-based ketones were used to synthesize compounds such as **67** and **68** with extended fused arenes for optical and electrochemical study.^151,152^ The 6,6′-diacyl compound **69** showed enhanced two-photon absorption in the near-infrared region, and it can be used for photocleavable drug delivery.^153^

Reaction of ethyl succinyl chloride (1.1 equiv) with (*R*)-BINOL-Me was conducted in the presence of AlCl_3_ (1.1 equiv) at 0 °C to room temperature to give the monoacyl product (*R*)-**70** in 60% yield.^154^ (*R*)-**70** was converted to a polystyrene supported binaphthyl-2,2′-bis(diphenylphosphine) (BINAP) ligand for asymmetric hydrogenation. It was also converted to a polyethylene glycol-supported BINOL (*R*)-**71**, which in combination with Ca^2+^ was used to catalyze the asymmetric Michael addition and epoxidation.^155^
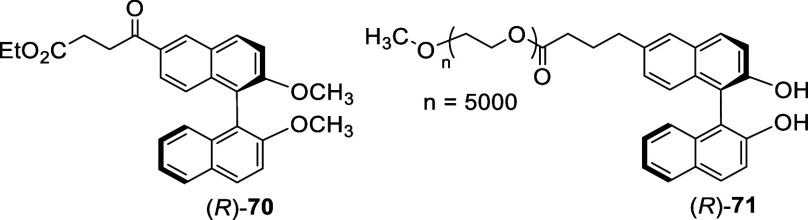


6-Acylation of a 6-substituted BINOL derivative **72** was conducted in the presence of ArCOCl and AlCl_3_ at 0 °C for 4 h. After hydrolysis, a BINOL-based hydroxy ketone derivative **73** with two different functional groups at the 6,6′-positions was obtained in 74% yield.^156^
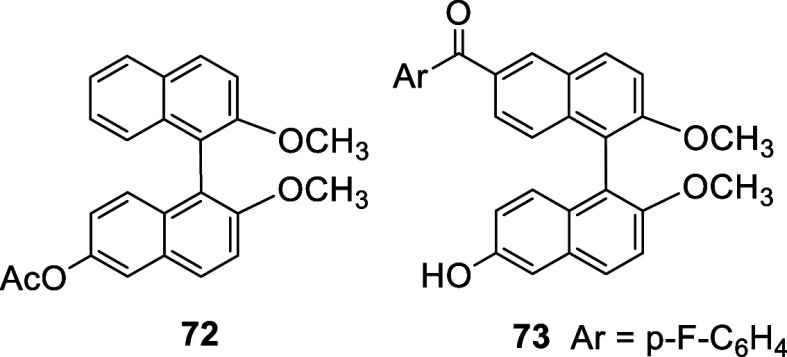


## 3 Electrophilic Substitution at 5-Position

**Scheme 21:**
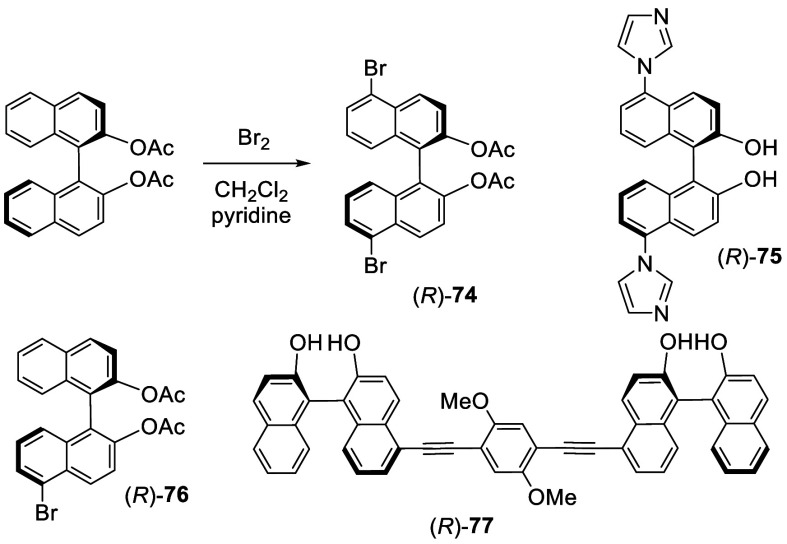
Bromination at 5,5′-Positions of BINOL Diacetate

In 2011, Yang et al. reported that when the acetate of (*R*)-BINOL was treated with bromine (6 equiv) in CH_2_Cl_2_ in the presence of pyridine at room temperature for 81 h, a 5,5′-dibromination took place to give (*R*)-**74** in 42% yield (Scheme 21).^157^ The structure of this compound was confirmed by a single crystal X-ray analysis of its derivative (*R*)-**75** prepared from the reaction of (*R*)-**74** with imidazole in the presence of CuI followed by hydrolysis. The reduced electron-donating effect of the acetate groups versus the hydroxyl groups of BINOL have changed the regioselectivity of the electrophilic substitution from the 6-position to the 5-position. The monobromide (*R*)-**76** was later prepared under the condition similar to the synthesis of (*R*)-**74** and was used to make the dimeric compound (*R*)-**77**.^158^ Formation of (*R*)-**74** and (*R*)-**76** from the bromination of BINOL diacetate demonstrates that the regioselectivity in the electrophilic substitution of BINOL is highly sensitive toward the electronic property of the 2,2′-hydroxyl groups.

In 2014, Agnes et al. conducted X-ray analyses on the products obtained in the bromination of BINOL. Formation of a small amount of a 5-brominated side product was identified.^159^ That is, besides the formation of the predominated product 6,6′-Br_2_BINOL, (*R*)- or (*S*)-**1**, the 5,6′-dibromoBINOL **78** was also generated, albeit in very low yield. This indicates that the 5-position could be the next reactive site after the 6-position in the electrophilic substitution. The 5,5′-dibromination shown in Scheme 21 demonstrates that when the electron-donating effect of the two hydroxyl groups is reduced, the 5,5′-positions becomes more reactive than the 6,6′-positions.
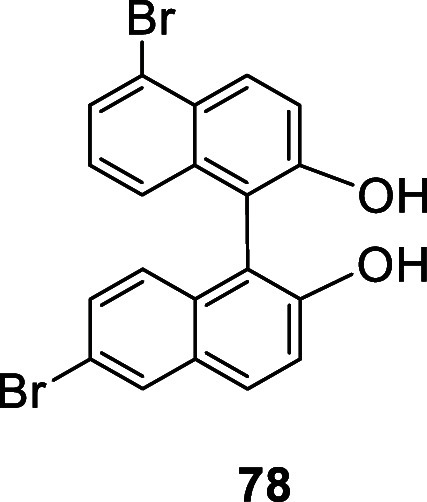


**Scheme 22:**
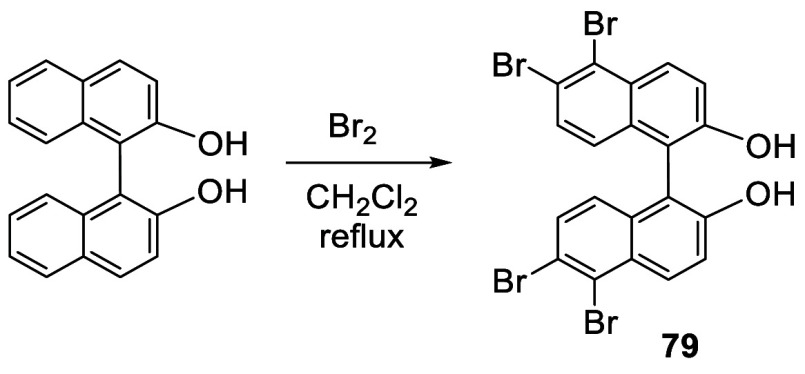
Tetrabromination at 6,6′,5,5′-Positions of BINOL

In 1978, Cram et al. reported that when racemic BINOL was heated with bromine (10 equiv) in CH_2_Cl_2_ at reflux for 19 h, 5,5′,6,6′-tetrabromoBINOL **79** was obtained in 81% yield (Scheme 22).^160^ This indicates that when the 6,6′-positions of BINOL are occupied by bromine atoms, further electrophilic substitution occurs at the 5,5′-positions.

**Scheme 23:**
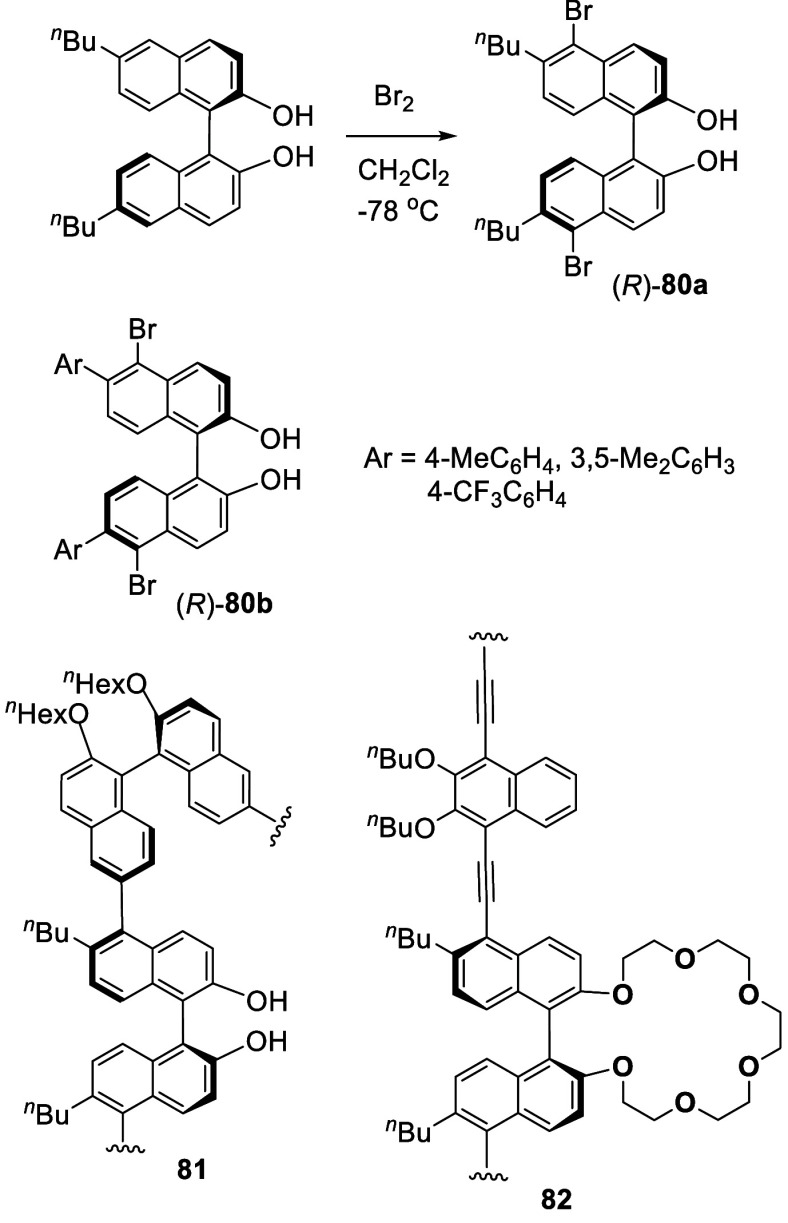
Bromination of 6,6′-DibutylBINOL

In 2008, Cheng and co-workers reported that the reaction of (*R*)-6,6′-^*n*^Bu_2_BINOL with bromine in CH_2_Cl_2_ at −78 °C over 1 h gave the 5,5′-dibromo product (*R*)-**80a** in 99% yield (Scheme 23).^161^ 6,6′-Diaryl substituted BINOLs were also brominated at −15 or 0 °C to give (*R*)-**80b** in 77–83% yields. (*S*)-**80a** was polymerized with the 6,6′-diboronic acid functionalized (*S*)-BINOL dihexyl ether, and the resulting polymer **81** was used to catalyze the asymmetric alkyne addition to aldehydes.^162^ The 5,5′-polymerized crown ether **82** was synthesized and its fluorescence responses toward metal cations were studied.^163^

**Scheme 24:**
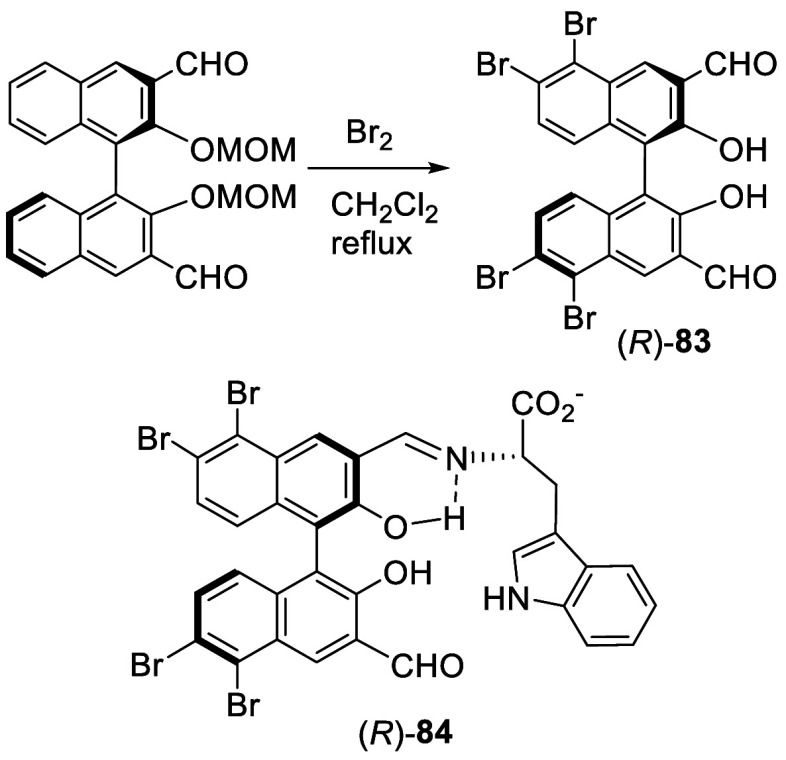
Tetrabromination at 6,6′,5,5′-Positions of a 3,3′-DiformylBINOL-MOM

When (*R*)-3,3′-diformylBINOL dimethoxymethyl ether was reacted with bromine (6 equiv) in refluxing CH_2_Cl_2_ for 6 h, the 5,5′,6,6′-tetrabromo product (*R*)-**83** was obtained (Scheme 24).^164^ The two MOM groups were removed during the reaction. 2D NMR analyses were used to establish the structure of (*R*)-**83**. This tetrabromination increased the water solubility of 3,3′-diformylBINOL and allowed the enantioselective fluorescent recognition of chiral amino acids in the presence of Zn^2+^ to be conducted in aqueous solution. Reaction of (*R*)-**83** with 1 equiv of tryptophan gave (*R*)-**84**, which showed dual responsive fluorescence emissions and can be used to simultaneously determine the concentration and enantiomeric composition of an amino acid sample by one fluorescence measurement.

**Scheme 25:**
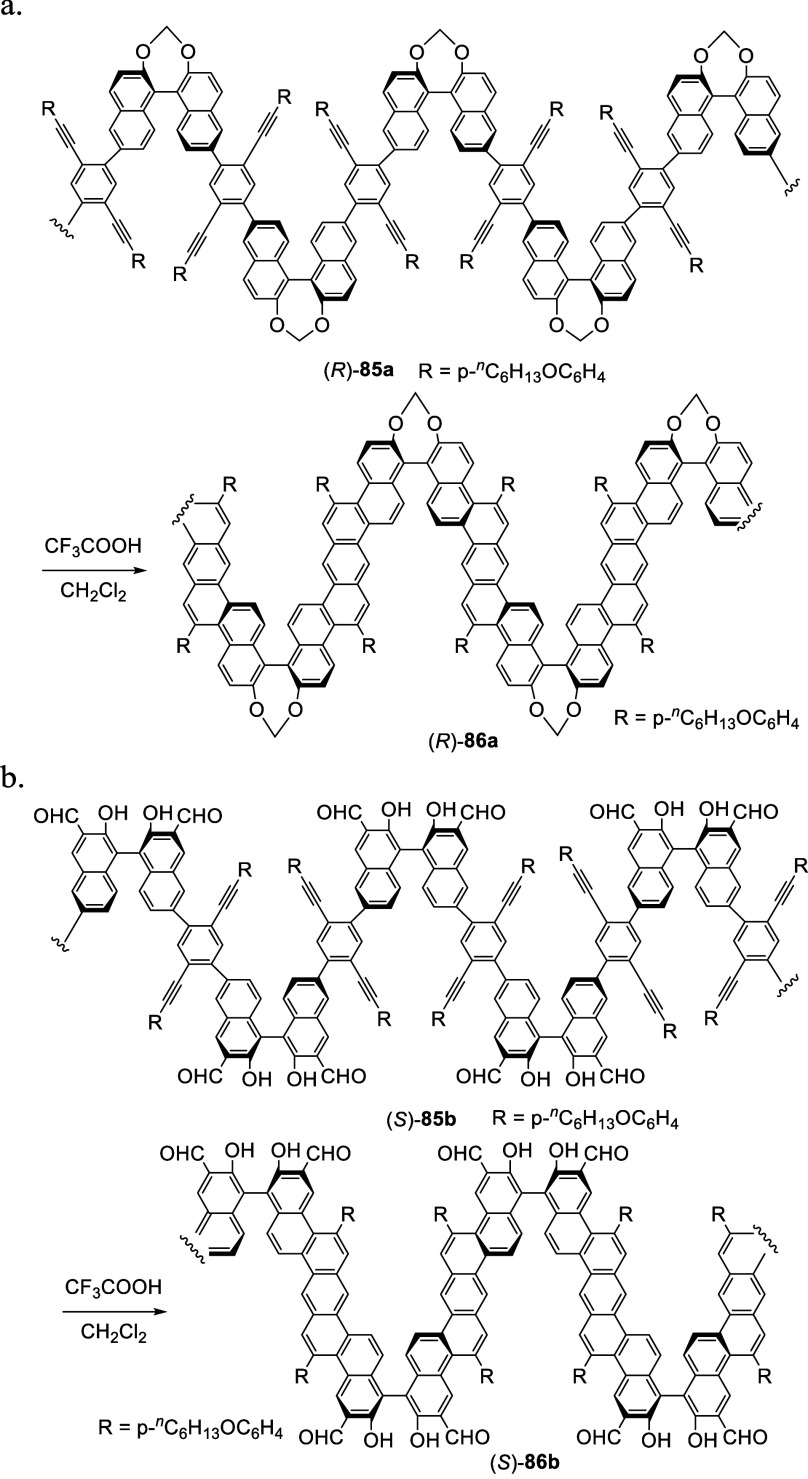
Synthesis of Helical Ladder Polymers by Selective Cyclization at the 5-Positions of BINOL Units

An intramolecular electrophilic substitution at the 5-position of BINOL was used to prepare helical ladder polymers as shown in Scheme 25.^165,166^ Treatment of the 6,6′-polymerized BINOLs (*R*)-**85a** and (*S*)-**85b** with CF_3_CO_2_H in CH_2_Cl_2_ led to an intramolecular cycloaromatization to form the helical ladder polymers (*R*)-**86a** and (*S*)-**86b** with fused polyaromatic rings in the repeating units. In the conversions of the polymers from (*R*)-**85a** and (*S*)-**85b** to (*R*)-**86a** and (*S*)-**86b**, the intramolecular electrophilic substitution is expected to occur selectively at the 5-position but not at the 7-position, which is supported by the reactions shown in Scheme 22–24. No electrophilic reaction at 7-positon is observed for BINOL and its derivatives. The fluorescence response of polymer (*S*)-**86b** toward amines was studied.^166^

## 4 Electrophilic Substitution at 4-Position

**Scheme 26:**
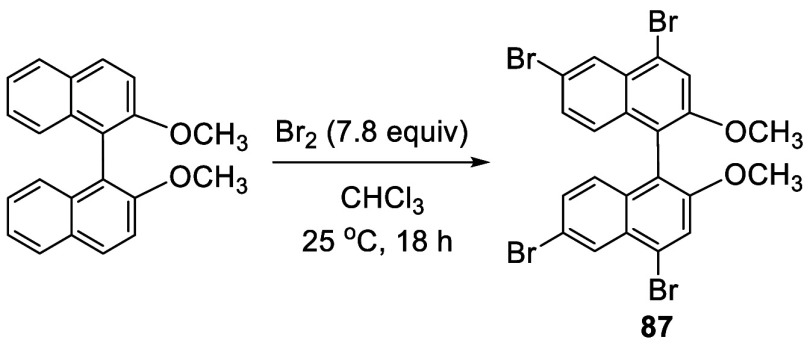
Tetrabromination at 4,4′,6,6′-Positions of BINOL-Me

In 1978, Cram et al. reported that when racemic BINOL-Me was treated with bromine (7.8 equiv) in CHCl_3_ at 25 °C for 18 h, the 4,4′,6,6′-tetrabrominated compound **87** was obtained in 91% yield (Scheme 26).^160^ This result is in sharp contrast to the reaction of BINOL with excess bromine in refluxing CH_2_Cl_2_, which gave the 5,5′,6,6′-tetrabromo product **79** (see Scheme 22). This indicates that there is a subtle difference between the reactivity at 4- and 5-positions. After the 6-position is substituted with a bromine atom, further bromination of the unalkylated BINOL containing the more electron-donating hydroxyl groups occurs at 5-position, but that of the alkylated BINOL containing the less electron-donating alkoxy groups occurs at the less sterically hindered 4-position.

**Scheme 27:**
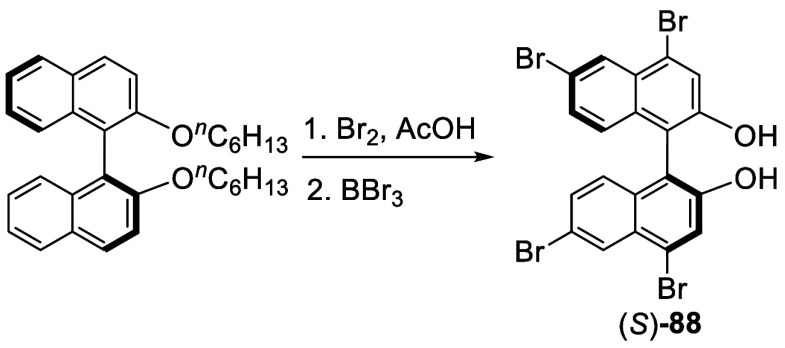
Synthesis of the Optically Active 4.4′,6,6′-TetrabromoBINOL

In 1999, Hu et al. prepared the optically active 4,4′,6,6′-tetrabromoBINOL (*S*)-**88** from the reaction of (*S*)-BINOL dihexyl ether with bromine in acetic acid solution at room temperature for 6 h, followed by removal of the hexyl groups with BBr_3_ (Scheme 27).^167,168^

**Figure 4:**
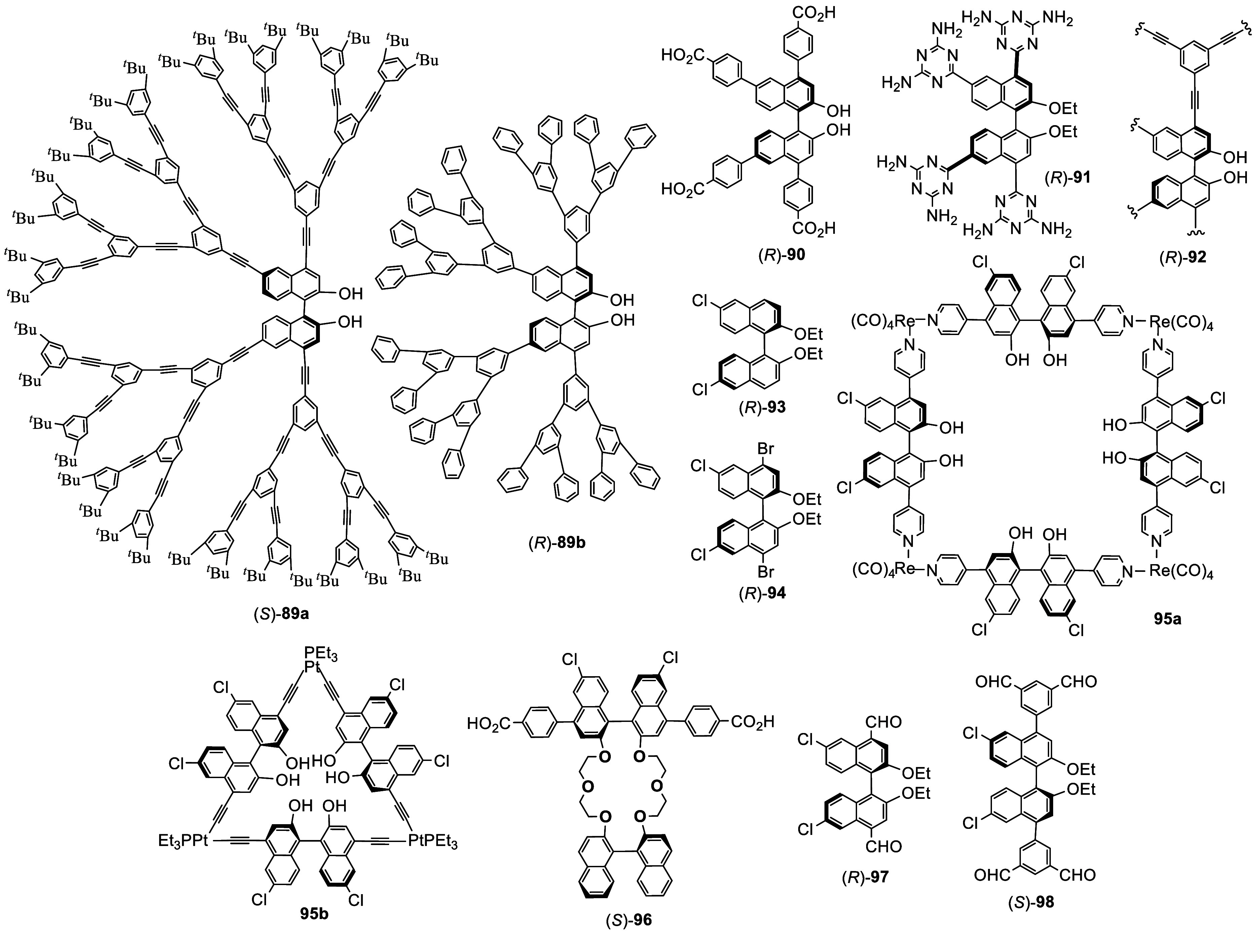
Examples for the application of 4,4′-dibromination of BINOLs.

Figure 4 shows a few examples of the chiral materials obtained by using the 4,4′,6,6′-tetrasubstituted BINOLs. Dendrimers like (*S*)-**89a** and (*R*)-**89b** were prepared from (*S*)- and (*R*)-**88**, respectively, and they showed light harvesting property as well as greatly enhanced fluorescent response in the enantioselective recognition of chiral amines.^167,169,170^ The tetracarboxylic acid substituted compounds such as (*R*)-**90** were synthesized to construct chiral porous MOFs by coordination with metal complexes such as Cu(NO_3_)_2_ and CdCl_2_ for enantioselective fluorescent sensing and asymmetric catalysis.^171,172^ Compound (*R*)-**91** was found to undergo self-assembly to form a chiral microporous hydrogen-bonded organic framework and was used for the enantioselective separation of chiral alcohols.^173^ The cross-coupling polymerization of the diacetate of (*R*)-**88** with 1,3,5-triethynyl benzene was conducted to generate the chiral microporous polymer (*R*)-**92** for the enantioselective fluorescent recognition of chiral amino alcohols.^174^

6,6′-Br_2_BINOL diethyl ether was converted to the 6,6′-dichloride (*R*)- or (*S*)-**93** by reaction with CuCl in DMF at 110 °C for 48 h, which was then reacted with bromine to give the 4,4′-dibromo product (*R*)- or (*S*)-**94** (Figure 4).^175^ The optically active **94** and its derivatives were used to make chiral MOFs,^175^ molecular square **95a**(176) and molecular triangle **95b**.^177^ These materials were used to conduct asymmetric catalysis and enantioselective fluorescent sensing. The 4,4′- bis(phenylcarboxylic acid) groups of the bisbinaphthyl-based crown ether (*S*)-**96**, made from (*S*)-**94**, was reacted with metal complexes such as Zn(NO_3_)_2_·4H_2_O to form MOFs.^178^ The 4,4′-dialdehyde (*R*)- or (*S*)-**97**, prepared from (*S*)-**94**, was used to condense with tetrakis(4-aminophenyl)ethane to form a chiral COF. This material was exfoliated into nanosheets for enantioselective fluorescent recognition of α-pinene.^179^ A 4,4′-substituted BINOL-based tetraaldehyde (*S*)-**98** was also used to make chiral porous organic cages for enantioselective recognition.^180^

When (*S*)-6,6′-^*t*^Bu_2_BINOL diethyl ether was treated with bromine in CH_2_Cl_2_ solution at −78 °C, (*S*)-**99** with 4.4′-dibromination was obtained in 99% yield.^181^ The cross-coupling reaction of (*S*)-**99** and its adamantanyl analogue (*R*)-**100a** with aryl tris-boronic acids and aryl tris- or tetra-terminal alkynes were used to make chiral porous polymers such as (*R*)-**100b** for enantioselective fluorescent recognition of limonene, α-pinene, and a chiral amine.
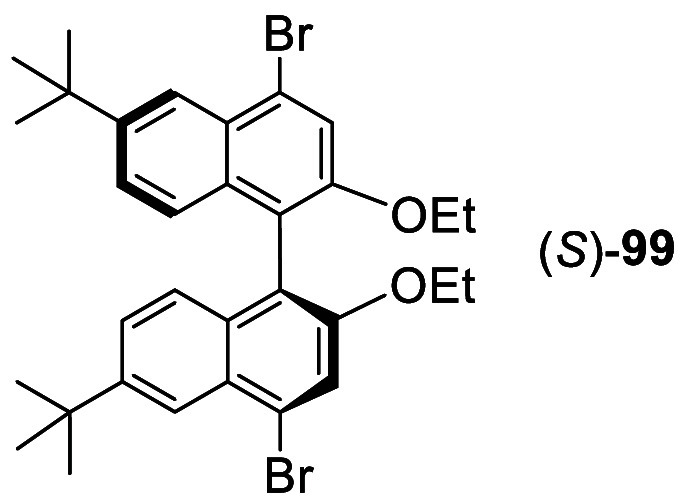
This paper reports that the bromination of 6,6′-^*t*^Bu_2_BINOL diethyl ether occurs at the 4,4′-position, which is in contrast to the reported 3,3′-dibromination of 6,6′-^*t*^Bu_2_BINOL to form (*S*)-**122**, where a single crystal X-ray analysis has confirmed the structure (see Scheme 36).^141^
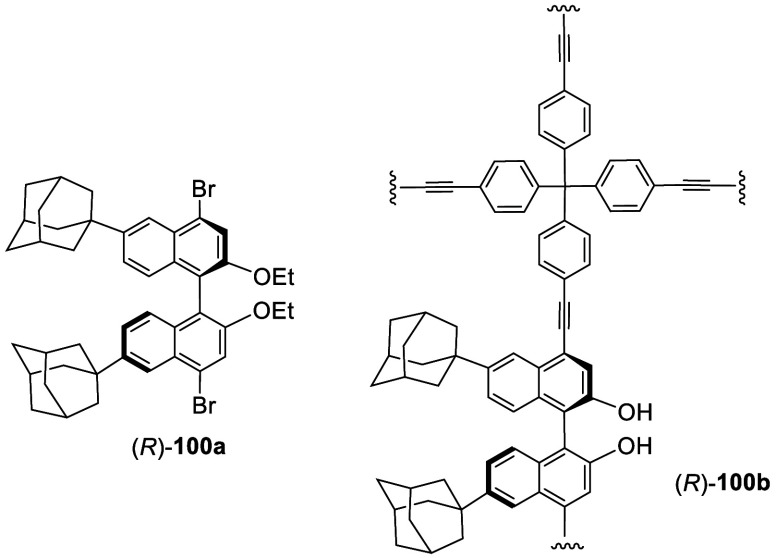


**Scheme 28:**
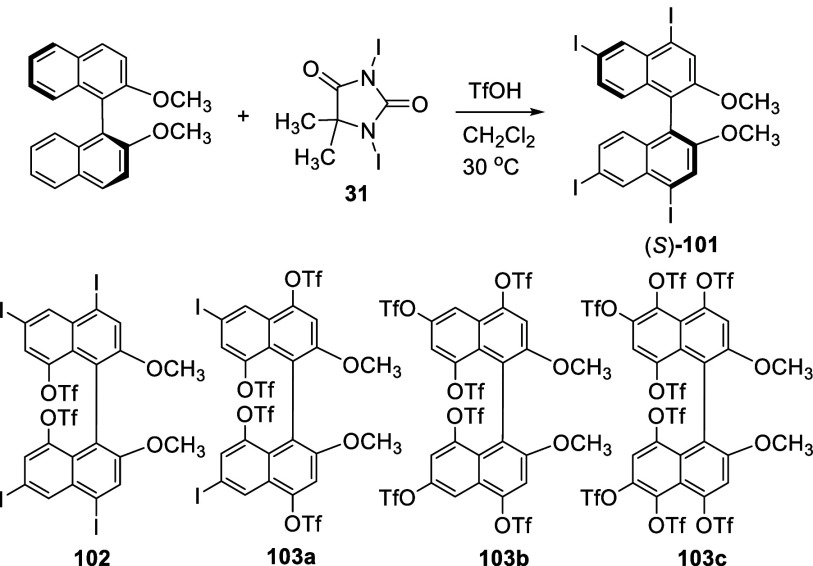
Tetraiodonation at 4,4′,6,6′-Positions of (*S*)-BINOL-Me

Treatment of (*S*)-BINOL-Me with diiodohydantoin **31** (2 equiv) and TfOH (10 mol %) in CH_2_Cl_2_ at 30 °C over 1 h gave the 4,4′,6,6′-tetraiodo product (*S*)-**101** in 74% yield (Scheme 28).^182^ This product was characterized by a single crystal X-ray analysis. When an excess amount of TfOH (5 equiv) was used for the reaction, the 8,8′-ditriflated product **102** was isolated in 21% yield with complete racemization. Compounds **103a** (10%), **103b** (24%), and **103c** (32%) were also obtained respectively when excess amount of both **31** (up to 15 equiv) and TfOH (up to 60 equiv) were used, and the structure of **103c** was characterized by X-ray analysis. When (*S*)-**101** was reacted with TMSOTf (2.2 equiv) and PhI(OCOCF_3_)_2_ (2.0 equiv) in CH_2_Cl_2_ at room temperature for 1 h, (*S*)-**102** was obtained in 45% yield in 69% ee as determined after removal of its methyl groups. This reaction indicates that the triflation of (*S*)-**101** by **31** and TfOH might involve a hypervalent iodine species, such as I(OTf)_3_, which could be generated from the disproportionation of TfOI formed from the reaction of **31** with TfOH. This hypervalent iodine might promote an oxidative nucleophilic substitution via a single electron transfer process to give the observed triflated BINOL products. When 6,6’-diiodo- or 6,6’-dibromoBINOL-Me was treated with TMSOTf (4.2 equiv) and PhI(OCOCF_3_)_2_ (4.0 equiv) in CH_2_Cl_2_ at room temperature for 1 h, compound **103a** or its 6,6’-dibromo analogue was also obtained in 43−50% yield.

An intramolecular electrophilic substitution at the 4-position of BINOL was used to prepare chiral ladder polymers similar to the reaction at the 5-position shown in Scheme 25. Treatment of the 3,3′-polymerized BINOL (*R*)-**104** with CF_3_CO_2_H in CH_2_Cl_2_ led to an intramolecular cycloaromatization at the 4-positions of the BINOL units to form the helical ladder polymer (*R*)-**105** with fused polyaromatic rings in the repeating units.^165^
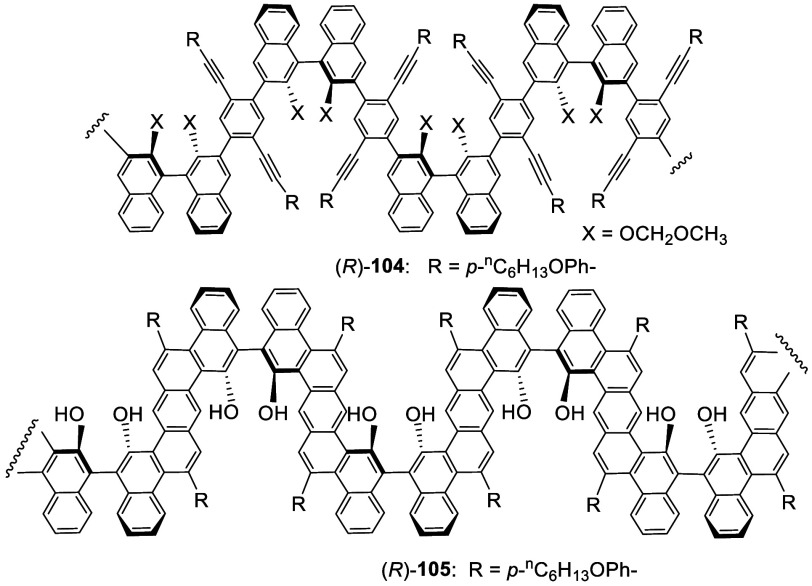


## 5 Electrophilic Substitution at 3-Position

**Scheme 29:**
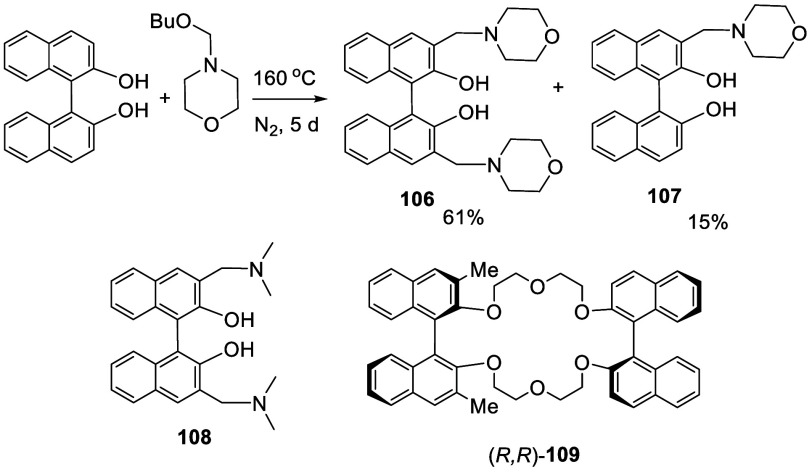
Mannich-Type Reaction of Racemic BINOL

In 1978, Cram et al. reported the Mannich type reaction of racemic BINOL with excess amount of (4-butoxymethyl)morpholine (no solvent) at 160 °C under N_2_ for 5 d to give the 3,3′-diaminomethyl product **106** in 61% yield (Scheme 29).^160^ The mono substituted product **107** was also obtained in 15% yield. The reaction of BINOL with dimethylamino-isobutoxymethane at 160 °C for 9 d gave the product **108** in 33% yield. These compounds were converted to 3,3′-dimethylBINOL, which was then resolved to the optically active product to build chiral crown ethers such as (*R,R*)-**109** for chiral recognition.

**Scheme 30:**
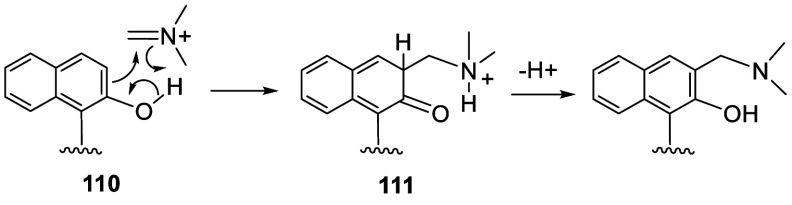
Proposed Mechanism for the Mannich Type Reaction of BINOL

In the reactions to form compounds **106**–**108**, the electrophile is an iminimium ion generated from the alkoxymethyl amine, which can react with BINOL via a transition state like **110** (Scheme 30). Formation of the intermediate **111** from **110** gives the regioselective aminomethylation at the 3-position. The intermediate **111** undergoes tautomerization and proton loss to give the final product.

**Scheme 31:**
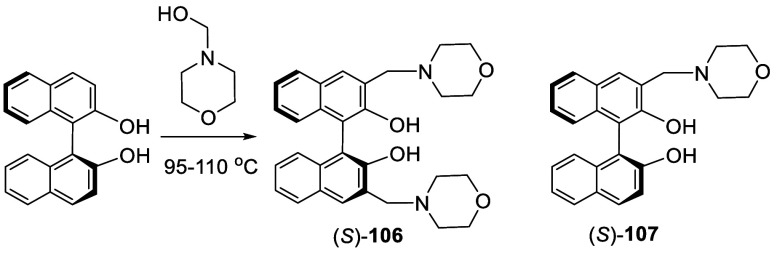
Synthesis of the Optically Active Aminomethyl BINOLs

In 2005, Qin et al. reported the use of the optically pure (*S*)-BINOL to react with morpholinomethanol, generated from the reaction of morpholine with paraformaldehyde without purification, to synthesize the optically active (*S*)-**106** (Scheme 31).^183,184^ When the reaction was conducted at 160 °C, the resulting product **106** was completely racemizaed. When the reaction was conducted at 95–100 °C under 30 psi N_2_ for 3 d, (*S*)-**107** was obtained in 60% yield with >99% ee. When the react was conducted at 110 °C under 30 psi N_2_ for 3 d, (*S*)-**106** was obtained in 55% yield with 75% ee which after recrystallization gave (*S*)-**106** in 37% yield and >99% ee. The crystal structure of (*S*)-**106** was obtained by X-ray analysis. (*S*)-**106** was used to catalyze the asymmetric reactions of aldehydes with diphenylzinc and TMSCN.

**Scheme 32:**
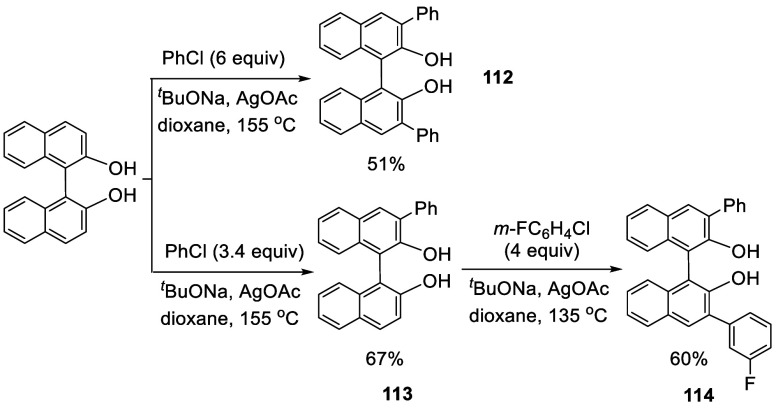
Direct Arylation of BINOL

**Scheme 33:**
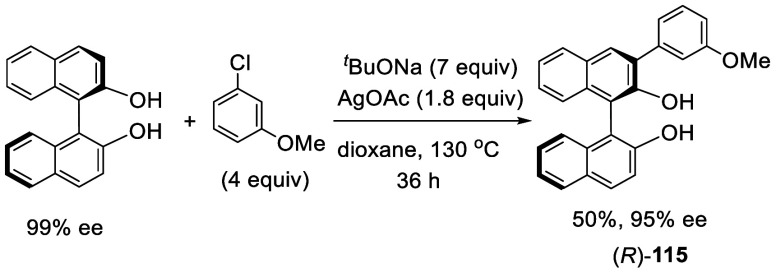
Arylation of the Enantiomerically Pure BINOL

In 2013, Truong and Daugulis reported the 3- and 3,3′-arylation of BINOL by reaction with aryl chloride in the presence of ^*t*^BuONa and AgOAc at elevated temperature (Scheme 32).^185^ When BINOL was reacted with 6 equiv of PhCl, 9 equiv of ^*t*^BuONa, and 2 equiv of AgOAc in dioxane at 155 °C sealed under N_2_ for 95 h, the 3,3′-diphenylBINOL **112** was obtained in 51% yield. The monophenyl product **113** was also obtained by the reaction of BINOL with 3.4 equiv of PhCl, 6 equiv of ^*t*^BuONa, and 1.7 equiv of AgOAc at 155 °C for 48 h. Reaction of **113** with an more electron-deficient *m*-fluorochlorobenzene at a lower temperature gave the unsymmetrically 3,3′-diarylated product **114** in 60% yield. When the enantiomerically pure (*R*)-BINOL was used to react with *m*-chloroanisole, the resulting monoarylated product (*R*)-**115** was obtained with high enantiomeric purity (Scheme 33). Thus, the extent of racemization was very low even though the reaction was conducted at 130 °C.

**Scheme 34:**
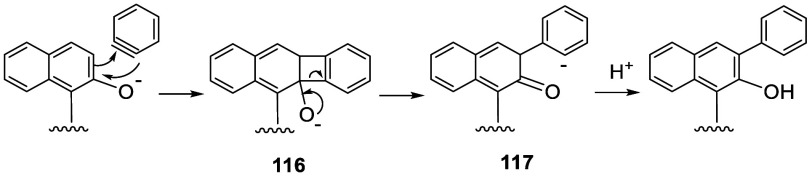
Proposed Mechanism for the Arylation of BINOL

A benzyne addition mechanism might be involved in these reactions as shown in Scheme 34. Benzyne generated from the reaction of phenyl chloride with ^*t*^BuONa and AgOAc can react with the deprotonated BINOL to form the 4-membered ring intermediate **116**. Ring-opening of **116** can give **117**, which upon protonation and tautomerization should give the arylated product.

**Scheme 35:**
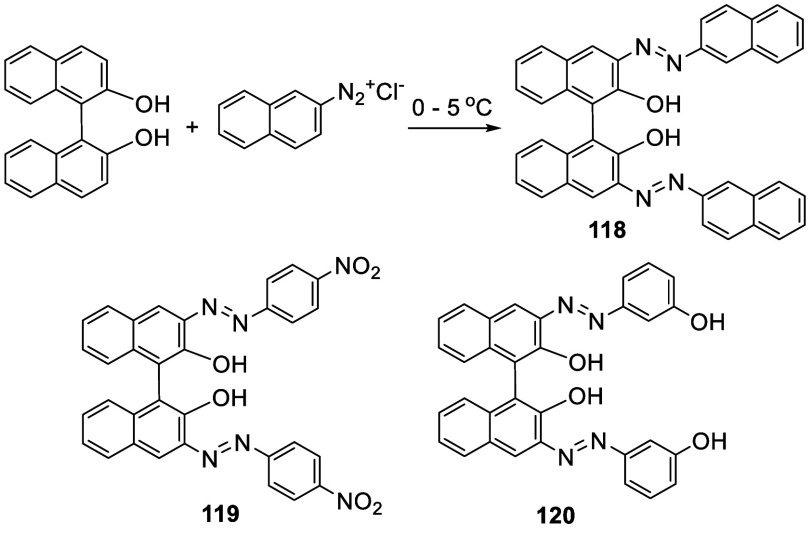
Reaction of BINOL with Aryl Diazonium Salts

Reaction of the alkaline solution of BINOL with the diazonium chlorides prepared from β-naphthylamine at 0–5 °C gave the 3,3′-bis(diazene) compound **118** (Scheme 35).^186^ This compound was used to prepare the dinuclear complexes of metal ions including Co(II), Cu(II), Ni(II), Zn(II), Cd(II), and Hg(II). Compounds **119** and **120** as well as their metal complexes were also prepared in a similar way.^187^

**Scheme 36:**
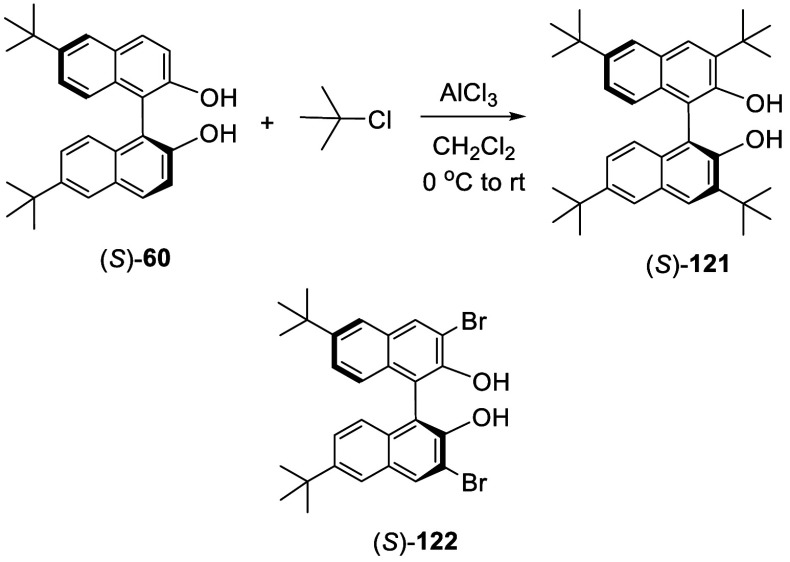
Friedel–Crafts Alkylation of (*S*)-6,6′-^*t*^Bu_2_BINOL

It was found that when the 6,6′-positions of BINOL are substituted with bulky groups such as ^*t*^Bu or 1-adamantanyl, the subsequent electrophilic substitutions take place at the least sterically hindered 3-position even though this position is not electronically favorable as shown by the HOMO orbital of BINOL in Figure 2. When (*S*)-6,6′-^*t*^Bu_2_BINOL, (*S*)-**60**, was reacted with ^*t*^BuCl and AlCl_3_ further at 0 °C for 1 h and at room temperature for 12 h, (*S*)-3,3′,6,6′-^*t*^Bu_4_BINOL, (*S*)-**121**, was obtained in 8–12% yield (Scheme 36).^141^ Reaction of (*S*)-**60** with excess bromine in CH_2_Cl_2_ at −78 °C for 3 h gave the 3,3′-dibromo product (*S*)-**122** in 80% yield. The structures of these 3,3′-substituted compounds were established by NMR and X-ray analyses.

**Scheme 37:**
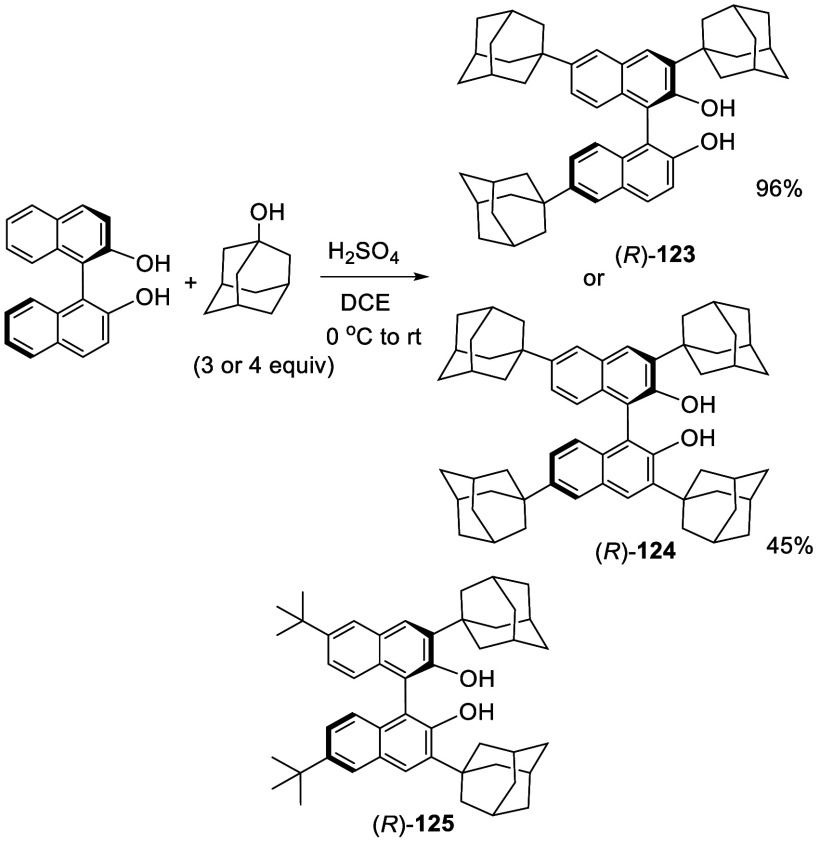
Preparation of Tri- and Tetra-adamantanyl BINOLs

Reaction of BINOL with 2 equiv of 1-adamananol in the presence of sulfuric acid gave 6,6′-diadamantanylBINOL (*R*)-**61** (see Scheme 17). When 3 and 4 equiv of 1-adamantanol were used to react with (*R*)-BINOL in the presence of sulfuric acid, the tri- and tetra-substituted BINOLs (*R*)-**123** and (*R*)-**124** were obtained, respectively (Scheme 37).^144^ The structure of (*R*)-**123** was established by 2D NMR analyses. (*R*)-6,6′- ^*t*^Bu_2_BINOL, (*R*)-**60**, was also used to react with 1-adamantanol in the presence of sulfuric acid to give the 3,3′-diadamantanyl substituted product (*R*)-**125**. These compounds were used to catalyze the reaction of ZnEt_2_ with aldehydes, and the unsymmetric ligand (*R*)-**123** showed higher enantioselectivity than the symmetric ones.

When (*R*)-6,6′-Br_2_BINOL, (*R*)-**1**, was treated with 1-adamantanol (3.1 equiv) in the presence of concentrated sulfuric acid at 0 °C for 30 min and then at room temperature for 47 h, the 3-adamantan-1-yl product (*R*)-**126** was obtained in 46% yield.^188^ This compound was found to undergo oxidative dearomatization with oxone to form **127** in 54% yield. Formation of racemic 3,3′-dinitro compound **128** was reported from the nitration of racemic **1** with *t*-butyl nitrite in THF at room temperature. (*R*)-6,6′-Di(methoxycarbonyl)-BINOL, prepared from (*R*)-**1**, was reported to react with bromine (10 equiv) at room temperature in ethyl acetate for 48 h to give (*R*)-**129** with 3,3′-dibromination in 73% yield.^189^
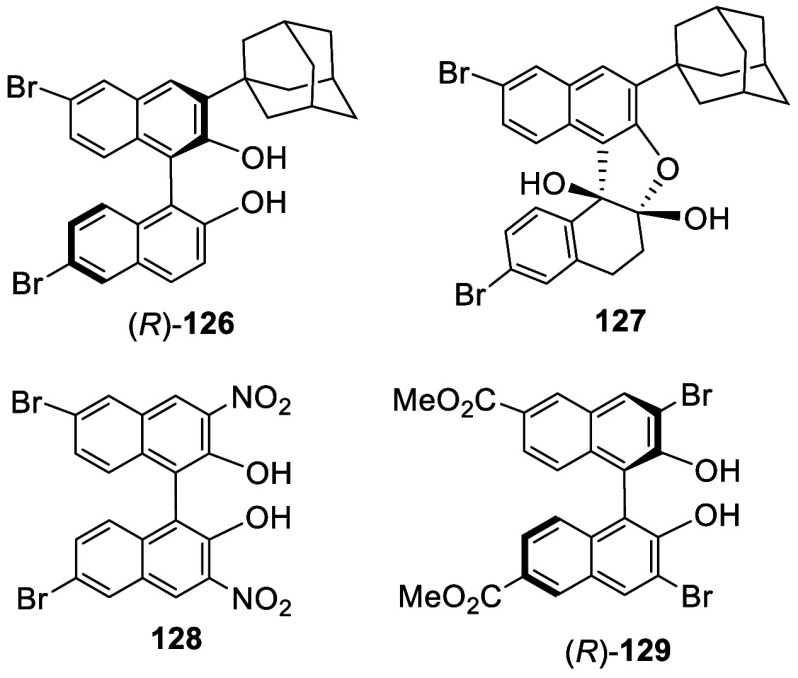


**Scheme 38:**
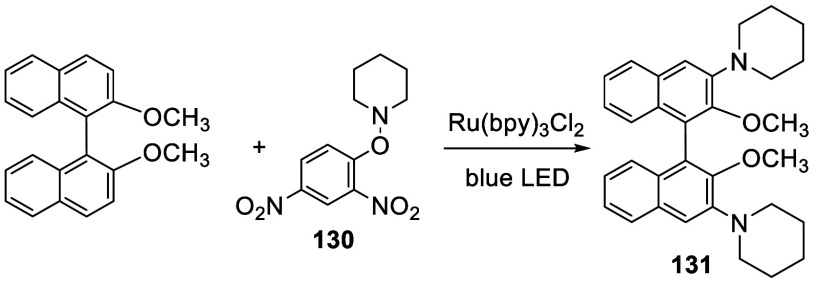
Photo-Amination of BINOL-Me

*O*-Aryl hydroxylamines such as **130** could produce an amminum radical cation in the presence of HClO_4_ (2 equiv) and a photocatalyst Ru(bpy)_3_Cl_2_ (2 mol %) under blue LED irradiation at room temperature. Its reaction with BINOL-Me was reported to give the 3,3′-disubstitutied product **131** in 56% yield (Scheme 38).^190^

Because the ^1^H NMR spectrum of a 3,3′-disubstituted BINOL is similar to that of its 4,4′-disubstituted regioisomer, more characterization may be needed to confirm the structures of a few of the 3,3′- and 4,4′-substituted BINOLs such as compounds **99**, **100a**, **128**, **129**, and **131**.

## 6 Electrophilic Substitution at 8-Position

As discussed in section 2.1, the 2-hydroxyl group of BINOL can provide resonance stabilization for the carbon cation intermediates formed from the electrophilic attack at the 3-, 6-, and 8-positions of the 2-naphthol ring (see Figure 1). The node plane in the HOMO orbital of BINOL (see Figure 2) makes the electrophilic reaction less favorable at the 3-position, and the steric hindrance makes the reaction less favorable at the 8-position. Thus, very few examples for the electrophilic substitution of BINOL at 8-position were reported. In section 2.4, nitration of (*S*)-**44** gave 8-nitro product (*S*)-**46** as a minor product in 18% yield (see Scheme 10).^131^ In this example, the high reactivity of the nitration agent might have partially overcome the steric hindrance at the 8-position to give the observed low yield production of the 8-substituted compound. The major product was the 6-substituted compound (*S*)-**45**. In Scheme 28, it is shown that when BINOL-Me was treated with excess iodonation agent **31** and TfOH, triflation at the 8,8′-positions can take place to give compounds **102** and **103a**–**c** after the 4,4′,6,6′-positions were substituted.^182^ The triflation reaction may be an oxidative nucleophilic substitution by TfO¯ involving a single electron transfer between a hypervalent intermediate like I(OTf)_3_ and the BINOL compound.

## 7 Ortho-lithiation at 3-Position

Because the 3- and 3,3′-positions of BINOL are close to the active sites when BINOLs are applied in asymmetric synthesis, catalysis, and molecular recognitions, a great number of the 3- and 3.3′-substituted BINOL derivatives have been synthesized in order to modify the steric and electronic environment of BINOL for those applications. Most of those compounds are prepared by ortho-lithiation of BINOL, directed by the ether, methoxymethyl ether, and other functional groups at the 2,2′-positions, followed by reactions with a great variety of electrophiles.

In this section, the ortho-lithiation reactions are classified according to the O-protecting groups (P) of the BINOL substrates as shown by **132a**. Among these, the conversions of **132a** containing P = Me, MeOCH_2_, and P(O)R_2_ to the products **132b** are further categorized according to the electrophilic additions used to introduce the X groups after the ortho-lithiation.
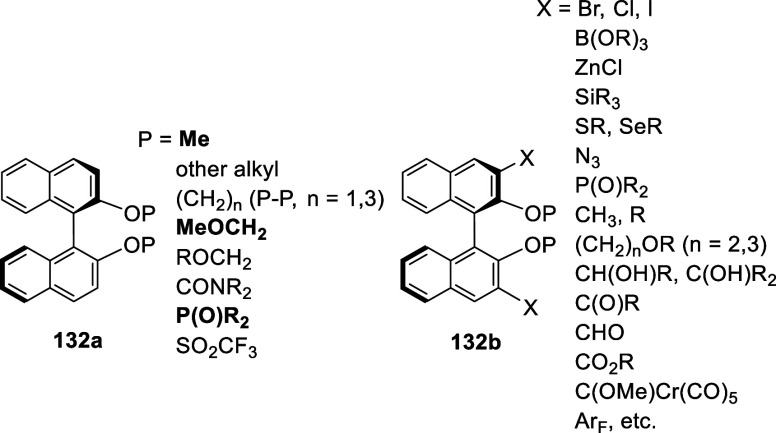


### 7.1 Reactions of BINOL Methyl Ether

Ortho-lithiation of BINOL-Me was used to prepare many 3- and 3,3′-substituted BINOLs. BINOL-Me was generally prepared from the reactions of BINOL with MeI in the presence of K_2_CO_3_^191^ or with Me_2_SO_4_ in the presence of NaOH^212^ or with NaH followed by reaction with Me_2_SO_4_.^215^

#### 7.1.1 Halogenation

**Scheme 39:**
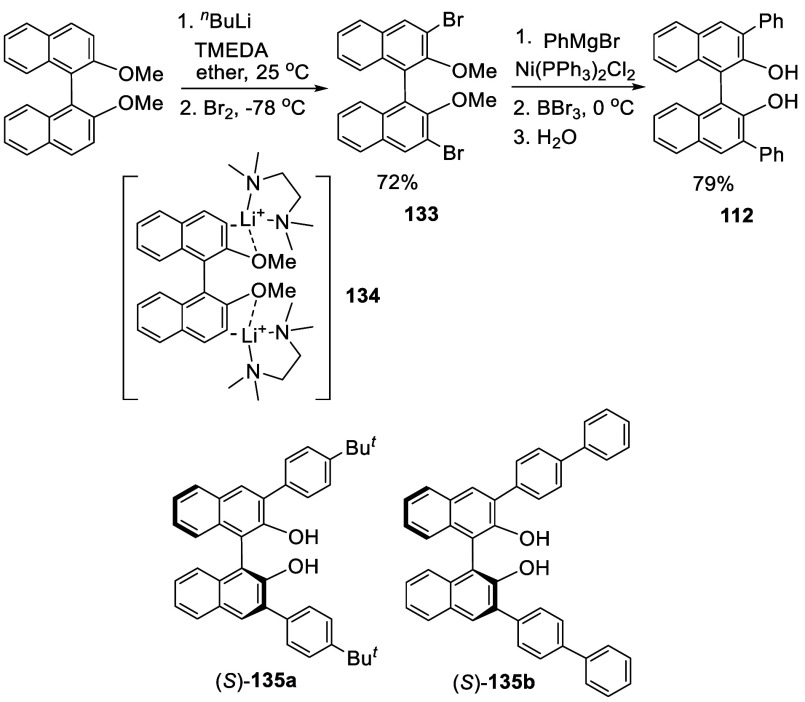
Ortho-lithiation Bromination of BINOL-Me

In 1981, Cram and co-workers reported the ortho-lithiation of racemic, (*R*)- and (*S*)-BINOL-Me with ^*n*^BuLi in the presence of tetramethylethylenediamine (TMEDA) to make racemic, (*R*)- and (*S*)-3,3′-dibromoBINOL **133** via an intermediate like **134** (Scheme 39).^191^ In this reaction, the methoxy groups of the substrate directed the lithiation at the ortho-position and TMEDA increased the basicity of ^*n*^BuLi for deprotonation. The reaction was conducted by treatment of BINOL-Me with 2.3 equiv of ^*n*^BuLi and 2.1 equiv of TMEDA at 25 °C for 3 h, followed by addition of excess bromine (9.4 equiv) at 75 °C for 10 min and then at 25 °C for 4 h to give **133** in 72% yield. This compound was used to prepare 3,3′-substituted BINOLs, which were then used to make crown ethers for chiral recognition. For example, the Ni(II)-catalyzed cross coupling of **133** with PhMgBr gave the 3,3′-diphenyl compound and the methyl ether was hydrolyzed after treatment with BBr_3_ to give 3,3′-Ph_2_BINOL, **112**. In the same way, the optically active (*S*)-BINOL-Me was used to prepare various (*S*)-3,3′-diarylBINOLs such as (*S*)-**112**, (*S*)-**135a**, and (*S*)-**135b**.^192^ These compounds were used in combination with BH_3_·THF to promote the asymmetric Diels–Alder reactions. (*R*)- or (*S*)-**133** were also used to prepare other BINOL-based compounds for various applications.^193−197^

**Scheme 40:**
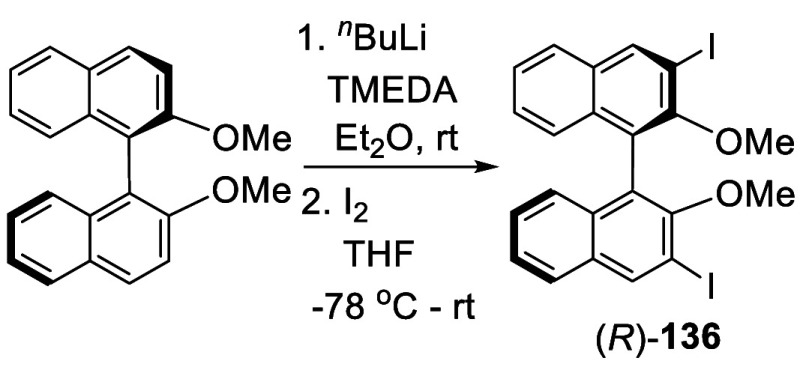
Ortho-lithiation Iodonation of (*R*)-BINOL-Me

Reaction of the ortho-lithiated (*R*)-BINOL-Me with I_2_ gave (*R*)-3,3′-I_2_BINOL-Me, (*R*)-**136** (Scheme 40).^198−200^ The reaction was conducted by treatment of BINOL-Me in Et_2_O with 3.7 equiv of ^*n*^BuLi and 3.7 equiv of TMEDA at 20 °C for 6 h, followed by addition of excess I_2_ (4.0 equiv) at −78 °C, which gave (*R*)-**136** in 90% yield.^199^ This compound was used to prepare 3,3′-diarylBINOLs for the Y(III)-catalyzed asymmetric olefin hydroamination/cyclization.^200^ Other BINOL-based compounds were also prepared from (*R*)- or (*S*)-**136** for various applications.^201−206^

#### 7.1.2 Borylation

**Scheme 41:**
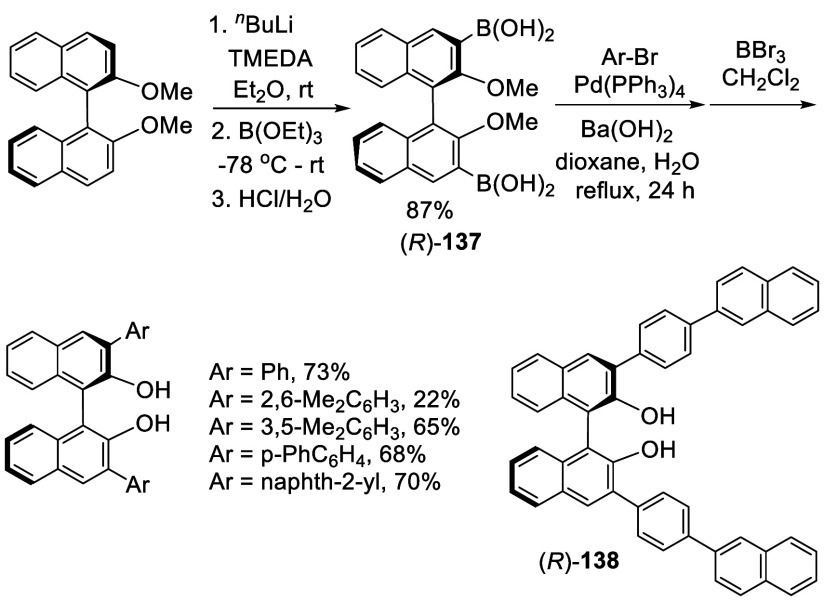
Ortho-lithiation Borylation of (*R*)-BINOL-Me

In 1998, Jørgensen and co-workers prepared (*R*)-3,3′-diboronic acid BINOL, (*R*)-**137**, from the reaction of (*R*)-BINOL-Me with ^*n*^BuLi-TMEDA, followed by addition of B(OEt)_3_ at −78 °C and then acidic hydrolysis (Scheme 41).^207^ (*R*)-**137** was purified by recrystallization from toluene and was used to prepare a series of optically active 3,3′-diaryl BINOLs by the Suzuki couplings with aryl bromides, followed by removal of the methyl groups with BBr_3_. A few of these compounds and others such as (*R*)- and (*S*)-**138** were used in combination with BH_3_·THF to promote the asymmetric Diels–Alder reactions.^192,208−210^

Racemic **137** was prepared in 48% yield by ortho-lithiation of racemic BINOL-Me followed by addition of B(OMe)_3_, which was used to react with a variety of aryl halides (Ar-X) to prepare a number of racemic 3,3′-diaryl BINOLs.^211^ The use of B(OMe)_3_ gave lower yield than the use of B(OEt)_3_ (87%) for the synthesis of the BINOL 3,3′-diboronic acid **137**.
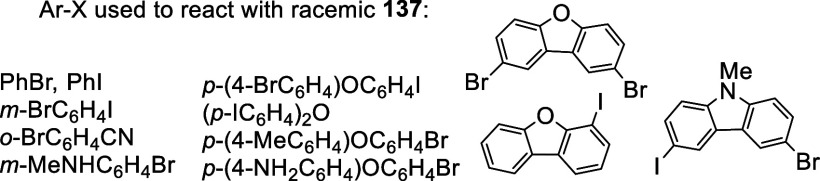


#### 7.1.3 Zincation

**Scheme 42:**
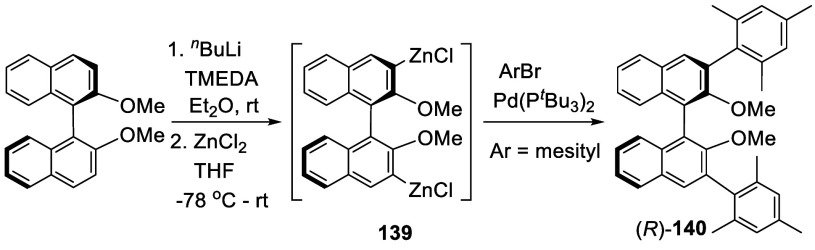
Ortho-lithiation Zincation of (*R*)-BINOL-Me

Transmetalation of the ortho-lithiated (*R*)-BINOL-Me with ZnCl_2_ was conducted to generate an arylzinc complex like **139** (Scheme 42).^200^ The Negishi coupling of **139** with mesityl bromide in the presence of Pd(P^*t*^Bu_3_)_2_ catalyst gave (*R*)-**140** in 74% yield in this one-pot reaction. When other ArBr (Ar = 2,6-^*i*^Pr_2_C_6_H_3_, 2,4,6-^*i*^Pr_3_C_6_H_2_) were used, lower yields (40–50%) were observed. After deprotection with BBr_3_, the resulting 3,3′-diarylBINOLs in combination with Y(III) complexes were used to catalyze the asymmetric olefin hydroamination/cyclization.

#### 7.1.4 Phosphorylation

**Scheme 43:**
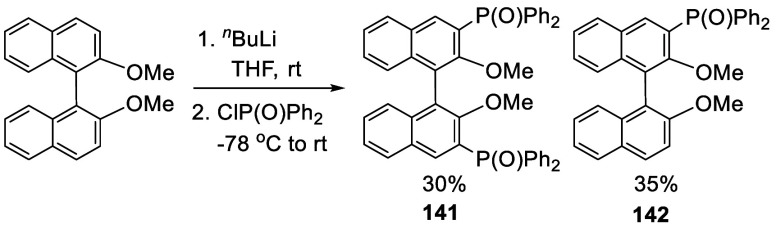
Ortho-lithiation Phosphorylation of BINOL-Me

BINOL-Me was reacted with 2.9 equiv of ^*n*^BuLi in THF solution at room temperature for 3 h, followed by addition of diphenylphosphinic chloride at −78 °C to give the mono- and diphosphorylated products **141** and **142** (Scheme 43).^212^ In these reactions, TMEDA was not used.

#### 7.1.5 Addition to Aldehydes and Ketones

**Scheme 44:**
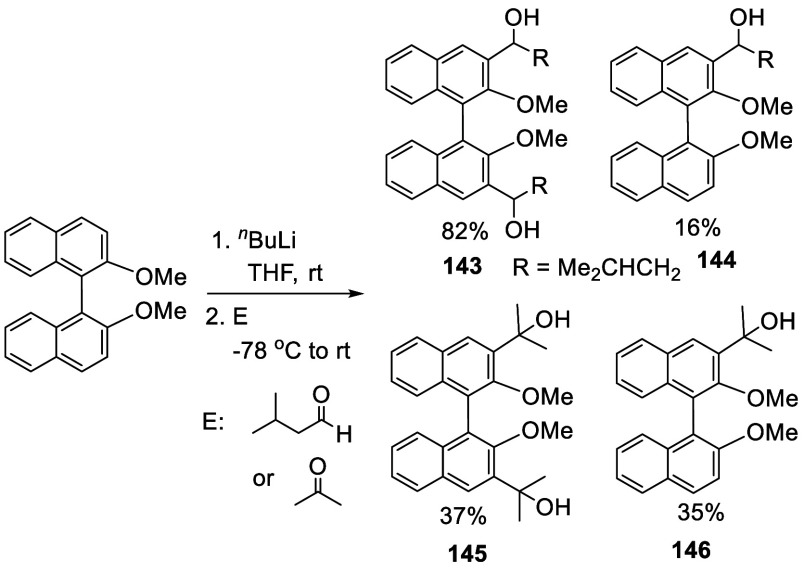
Addition of the Ortho-lithiated BINOL-Me to Isovaleraldehyde and Acetone

When BINOL-Me was treated with 2.9 equiv of ^*n*^BuLi in THF solution at room temperature for 3 h followed by the addition of isovaleraldehyde and acetone at −78 °C, the mono- and disubstituted compounds **143**–**146** were obtained (Scheme 44).^212^

**Scheme 45:**
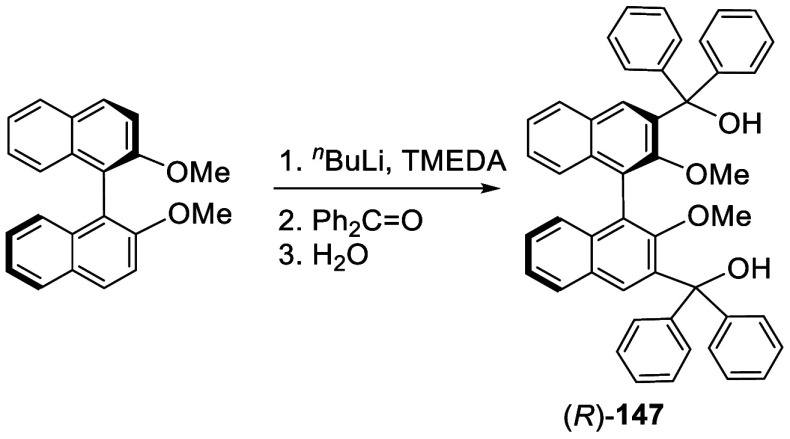
Addition of the Ortho-lithiated (*R*)-BINOL-Me to Benzophenone

When the ortho-lithiated (*R*)-BINOL-Me was reacted with benzophenone, (*R*)-**147** was obtained in 60% yield (Scheme 45).^213^ The fluorescence of this compound was studied.

#### 7.1.6 Addition to Acyl Chloride

**Scheme 46:**
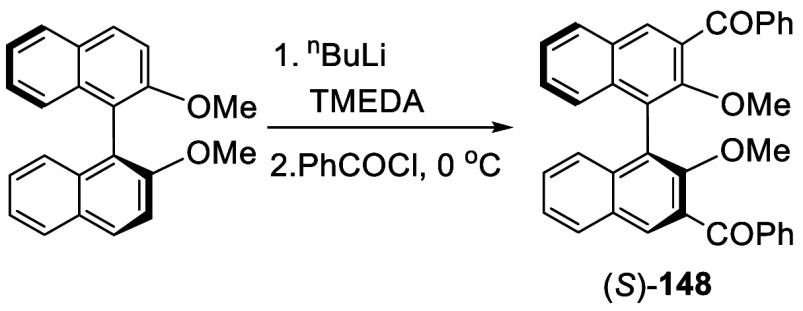
Addition of the Ortho-lithiated (*R*)-BINOL-Me to Benzoyl Chloride

After (*S*)-BINOL-Me was treated with 5 equiv of ^*n*^BuLi in Et_2_O at room temperature for 3 h, addition of 5 equiv of benzoyl chloride gave (*S*)-3,3′-dibenzoylBINOL, (*S*)-**148**, in 72% yield upon acidic workup (Scheme 46).^214^

#### 7.1.7 Addition to DMF

**Scheme 47:**
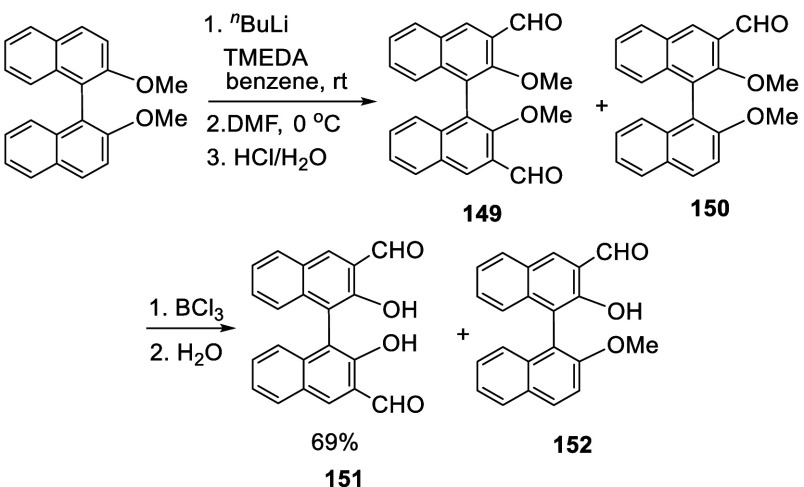
Addition of Ortho-lithiated BINOL-Me to DMF

When the ortho-lithiated BINOL-Me was reacted with DMF at 0 °C, a 3,3′-diformyl product **149** was obtained in 70% yield after acidic hydrolysis (Scheme 47).^215^ The monoformyl product **150** was also obtained in 20% yield. Because BCl_3_ was found to only promote the cleavage of the ether at the ortho-position of the carbonyl, a mixture of **149** and **150** were deprotected with BCl_3_ rather than BBr_3_. This gave a mixture of **151** and **152**, which were easily separated because, unlike **151**, compound **152** was not soluble in NaOH solution.

When the ortho-lithiation of the optically active (*S*)-BINOL-Me was conducted in THF at 0 °C, compound (*S*)-**149** was obtained only in 24% yield. However, when the ortho-lithiation was conducted in refluxing Et_2_O solution followed by reaction with DMF, (*S*)-**149** was obtained as the only product in 76% yield.^216^ This compound was enantiomerically pure as determined by HPLC analysis.
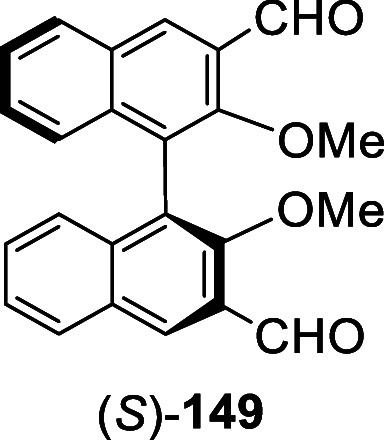


(*S*)- or (*R*)-**149** was used to prepare compound (*S*)- or (*R*)-**153** as a fluorescent probe for chiral discrimination of monosaccharides,^217^ and sugar acids.^218,219^ Compound (*S*)-**154** was synthesized from (*S*)-3,3′-diformylBINOL, (*S*)-**151**, for optical study.^220^
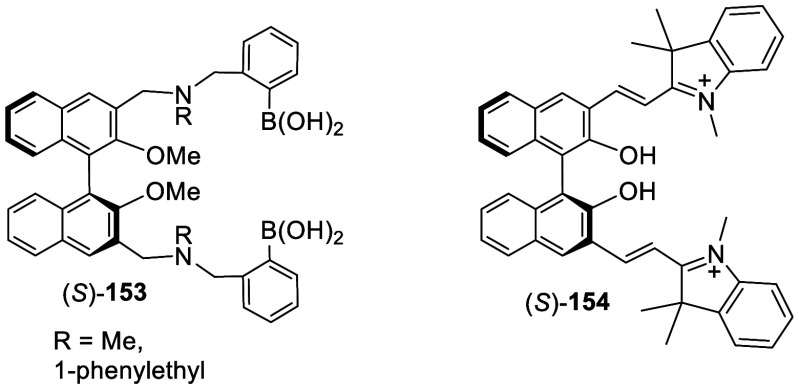


#### 7.1.8 Addition to CO_2_

**Scheme 48:**
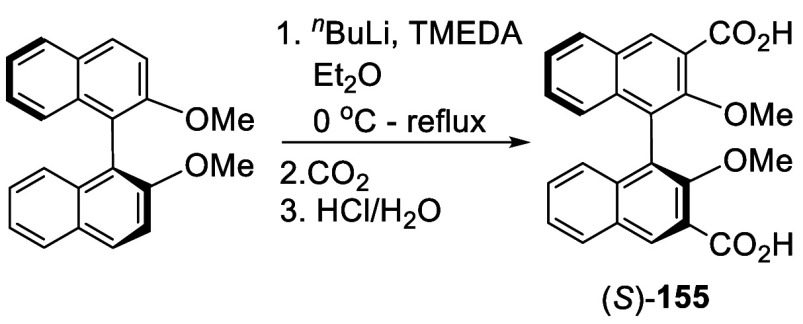
Addition of the Ortho-lithiated (*S*)-BINOL-Me to CO_2_

Ortho-lithiation of (*S*)-BINOL-Me with ^*n*^BuLi and TMEDA in refluxing Et_2_O was used to prepare a 3,3′-dicarboxylic acid BINOL (Scheme 48).^221^ After the lithiation, dry CO_2_ gas was introduced to the reaction mixture at 0 °C to give (*S*)-**155**, which was isolated in 66% yield after acidic workup and recrystallization from benzene. This compound was used as a precursor to modify the structure of iron porphyrins to catalyze the asymmetric epoxidation of styrene derivatives.

#### 7.1.9 Addition to Cr(CO)_6_

**Scheme 49:**
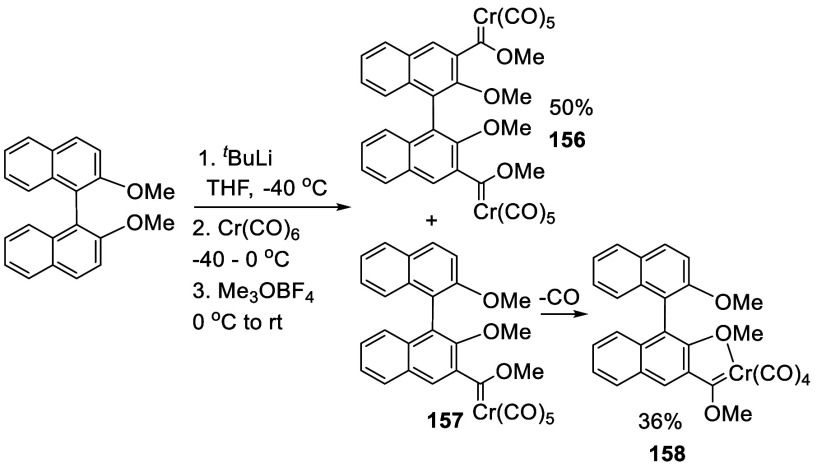
Addition of the Ortho-lithiated BINOL-Me to Cr(CO)_6_

**Scheme 50:**
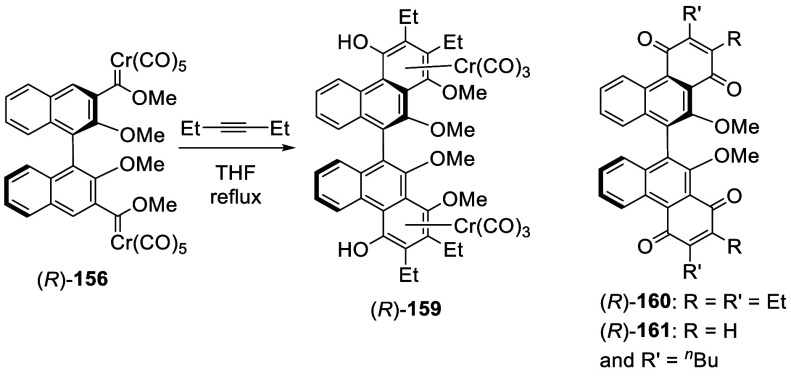
Cycloaddition of the BINOL Carbene (*R*)-**156** with 3-Hexyane

Ortho-lithiation of racemic (*R*)- and (*S*)-BINOL-Me were used to prepare the Fischer-type carbene complexes.^222^ After the racemic BINOL-Me was treated with 4 equiv of ^*t*^BuLi in THF at −40 °C for 6 h, Cr(CO)_6_ was added to react at −40 °C for 5 h and then at 0 °C for 15 h (Scheme 49). Removal of the solvent followed by the addition of Me_3_O^+^BF_4_^–^ in CH_2_Cl_2_ gave a mixture of products **156**, **157**, and **158**. After column chromatography, the biscarbene product **156** was obtained in 50% yield, and the monocarbene compound **157** was converted to the monocarbene **158**, which was isolated in 36% yield. The structure of **156** was established by a single crystal X-ray analysis. The optically active (*R*)-/(*S*)-**156** and (*R*)-/(*S*)-**158** were obtained and their CD and UV–vis absorption spectra were studied. (*R*)-**156** was reacted with 3-hexyne in refluxing THF, with further substitutions at the 4,4′-positions of the BINOL unit to give the benzannulation product (*R*)-**159** (Scheme 50). When the benzannulation was followed with oxidative demetalation in the presence of (NH_4_)_2_Ce(NO_3_)_6_, the chiral bisphenanthrenequinones (*R*)-**160** and (*R*)-**161** were obtained in 36–56% yields.^223^

**Scheme 51:**
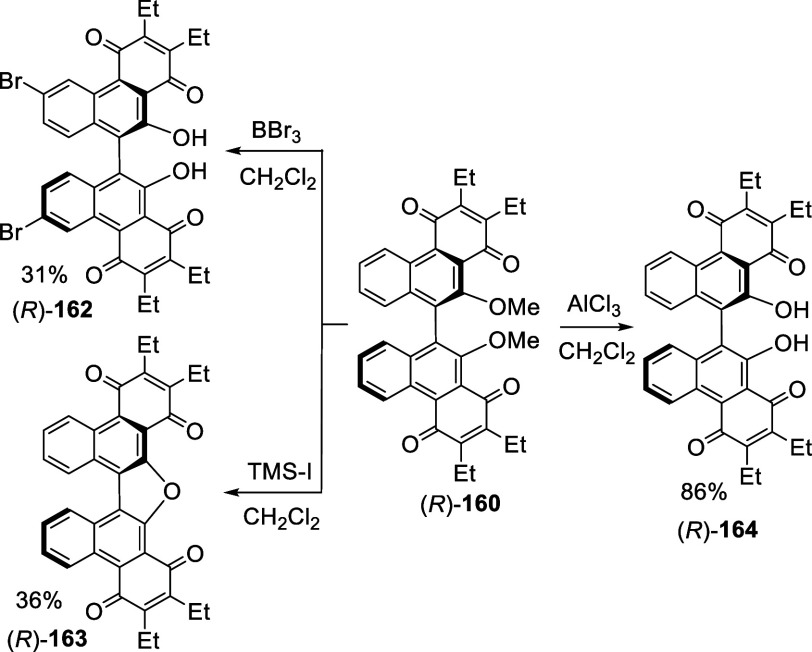
Attempted Demethylation Reactions of (*R*)-**160**

Several methods were tested for the demethylation of (*R*)-**160** and different products were obtained depending upon the reagents used (Scheme 51).^223^ Using excess amount of BBr_3_ not only removed the methyl groups but also brominated at the 6,6′-positions to give (*R*)-**162**. The use of TMS-I produced a central furan ring in the product (*R*)-**163** whose X-ray structure was obtained. When AlCl_3_ (10 equiv) were used, clean demethylation took place to give (*R*)-**164**. The two quinone units of compounds (*R*)-**160**, (*R*)-**161**, and (*R*)-**164** were shown to be electrochemically independent by their CV plots.

### 7.2 Reactions of Other BINOL Alkyl Ethers

**Scheme 52:**
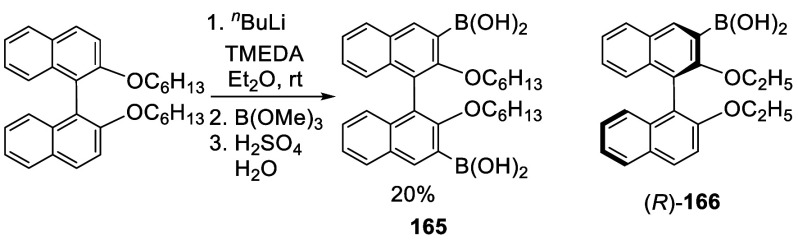
Ortho-lithiation and Borylation of BINOL Dihexyl Ether

BINOL dihexyl ether was used to prepare the diboronic acid **165**, which was purified by column chromatograph on silica gel eluted with ethyl acetate/petroleum ether followed by recrystallization from ethyl acetate (Scheme 52).^224^ Compound **165** was then converted to the corresponding boronic ester to make polymers for LED application. The monoboronic acid (*R*)-**166** was synthesized from (*R*)-BINOL diethyl ether in a similar way in 51% yield after purification by column chromatography on silica gel.^111^

**Scheme 53:**
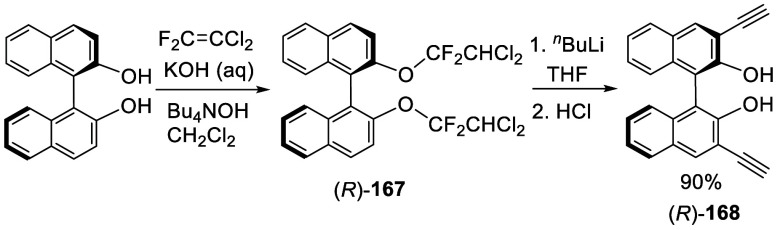
Conversion of (*R*)-BINOL Haloalkyl Ether to (*R*)-DiethynylBINOL

**Scheme 54:**
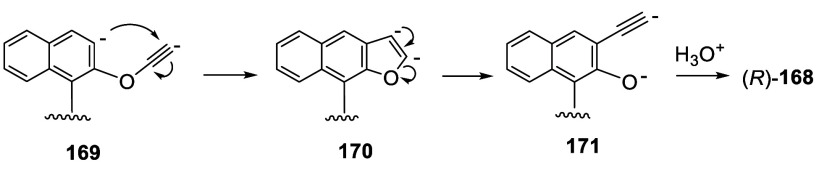
Proposed Mechanism for the Formation of (*R*)-**168**

A BINOLbis(dichlorodifluoroethyl) ether (*R*)-**167** was prepared from (*R*)-BINOL in 96% yield (Scheme 53).^225^ When this compound was treated with 12 equiv of ^*n*^BuLi in THF at −78 °C to room temperature, a remarkably clean rearrangement took place, which upon quenching with 1 N HCl gave (*R*)-3,3′-diethynylBINOL, (*R*)-**168**, in 90% yield. The base-promoted dehalogenation of (*R*)-**168** could generate an anionic intermediate like **169**, which could undergo cyclization to form **170** (Scheme 54).^226^ Ring-opening of **170** would give **171**, which upon acidic workup would give the product (*R*)-**168**. This compound was used to prepare bisBINOL macrocycles by Sonogashira coupling reactions.

### 7.3 Reactions of Alkyl Bridged BINOLs

**Scheme 55:**
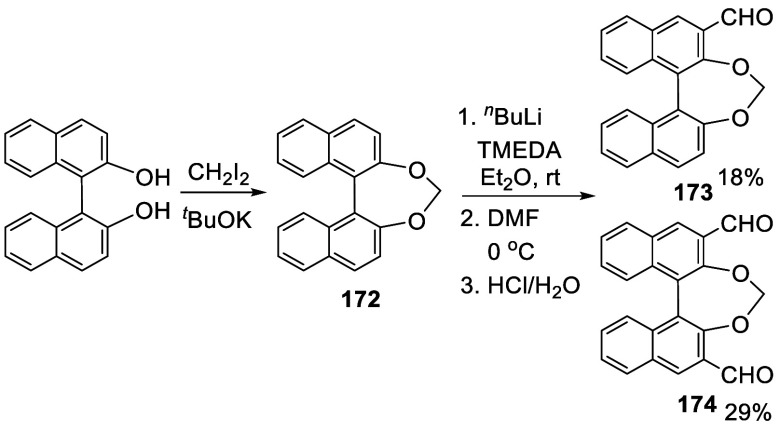
Addition of the Ortho-lithiated Methylene Bridged BINOL to DMF

BINOL with a methylene bridge was synthesized from the reaction of BINOL with CH_2_I_2_ in the presence of ^*t*^BuOK (Scheme 55).^216^ The resulting product **172** was subjected to ortho-lithiation followed by reaction with DMF to give the monoaldehyde **173** and the dialdehyde **174** in low yields.

**Scheme 56:**
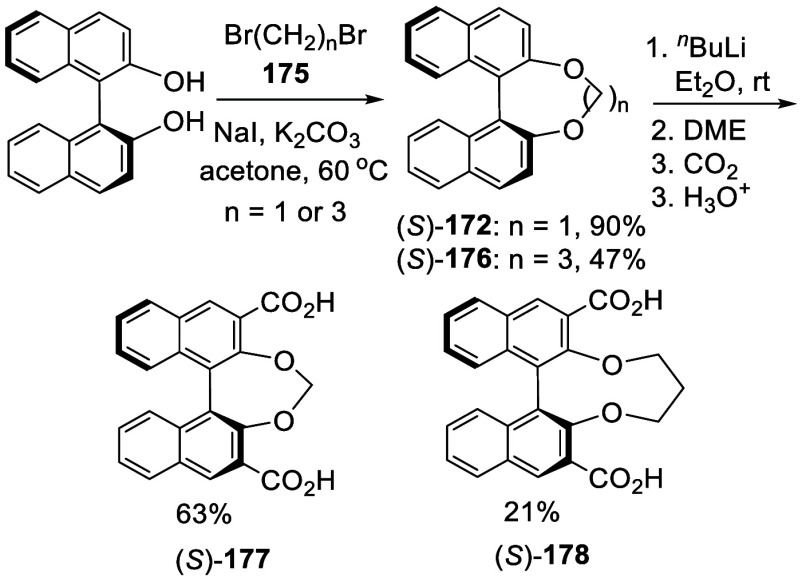
Addition of the Ortho-lithiated Alkyl Bridged BINOLs to CO_2_

Reaction of (*S*)-BINOL with Br(CH_2_)*_n_*Br (**175**, *n* = 1 or 3) in the presence of NaI and K_2_CO_3_ in acetone at 60 °C gave compounds (*S*)-**172** and (*S*)-**176** (Scheme 56).^227^ In the reaction of (*S*)-**172** with ^*n*^BuLi, DME (dimethoxyethane) was added instead of TMEDA. After the ortho-lithiation, CO_2_ gas was introduced, which upon acidic workup gave (*S*)-**177** in 63% yield. For the reaction of (*S*)-**176** to synthesize (*S*)-**178**, a mixed solvent of Et_2_O and THF was used and DME was also added. These compounds were used to prepare polymeric membranes.

### 7.4 Reactions of BINOL Methoxymethyl Ether

Ortho-lithiation of BINOL dimethoxymethyl ether (BINOL-MOM) followed by reaction with a broad range of electrophiles has been used to prepare many 3,3′-disubstituted or 3-monosubstituted BINOLs for diverse applications. This section is classified according to the reactions of the ortho-lithiated BINOL-MOM with various electrophiles. BINOL-MOM was prepared from the reaction of BINOL with MOMCl in the presence of dry K_2_CO_3_^212^ or with NaH followed by addition of MOMCl.^295,311^ The MOM groups can be easily removed by treatment with HCl(aq) or CF_3_CO_2_H.

#### 7.4.1 Halogenation

**Scheme 57:**
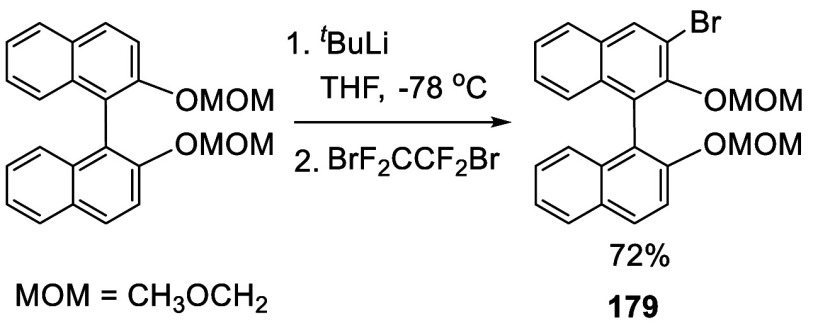
Monobromination of BINOL-MOM by Ortho-lithiation

In 1992, Snieckus and co-workers first reported the use of the MOM groups at the 2,2′-positions of BINOL to direct the ortho-lithiation at the 3-positions to prepare various 3- or 3,3′-functionalized BINOLs.^228^ When BINOL-MOM was treated with 2.2 equiv of ^*t*^BuLi in THF at −78 °C for 1 h, followed by the addition of 1,2-dibromotetrafluoroethane, the monobrominated compound **179** was obtained in 72% yield (Scheme 57).

**Scheme 58:**
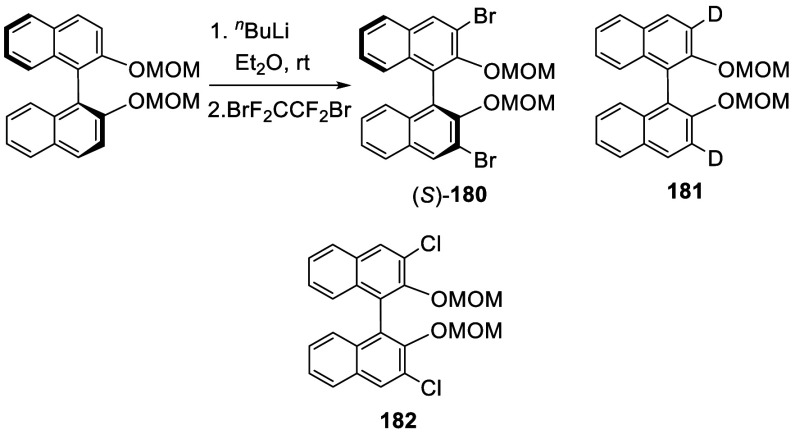
Dibromination of (*S*)-BINOL-MOM by Ortho-lithiation

When (*S*)-BINOL-MOM was treated with 3 equiv of ^*n*^BuLi in Et_2_O at room temperature for 3 h followed by the addition of 1,2-dibromotetrafluoroethane, the 3,3′-dibromo product (*S*)-**180** was obtained in 84% yield (Scheme 58).^228^ Retention of the chiral configuration of BINOL in this reaction was established. When D_2_O was added to quench the ortho-lithiated BINOL-MOM, the 3,3′-dideuteride compound **181** was obtained in 93% yield. 1,2-Dibromotetrachloroethane was used in place of 1,2-dibromotetrafluoroethane to produce (*R*)-**180** in 84% yield.^229,230^ Br_2_ in pentane was also used to react with the ortho-lithiated (*S*)-BINOL-MOM to give (*S*)-**180** in 90% yield.^231^ When hexachloroethane was used as the electrophile after BINOL-MOM was lithiated under these conditions, 3,3′-dichloroBINOL-MOM **182** was obtained in 95% yield.

**Scheme 59:**
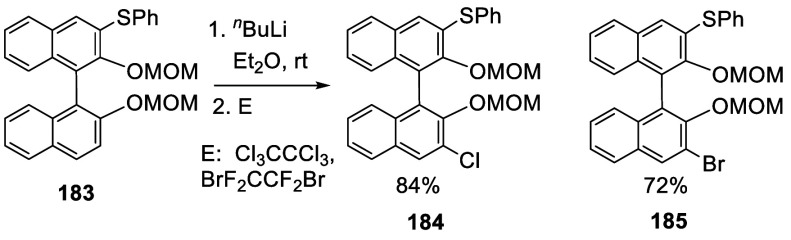
Ortho-lithiation Halogenation of a 3-Substituted BINOL-MOM

A 3-monothiolated BINOL-MOM **183** (see Scheme 68 for synthesis) was treated with 2 equiv of ^*n*^BuLi in Et_2_O at room temperature for 3 h, which was then reacted with a chlorination or a bromination agent to give the unsymmetrically 3,3′-disubstituted products **184** and **185** in 72–84% yields (Scheme 59).^228^

In the reactions of BINOL-MOM with an alkyl lithium, intermediates like **186** and **187** may be involved to direct the ortho lithiation at the 3-position of BINOL before the subsequent reaction with electrophiles.
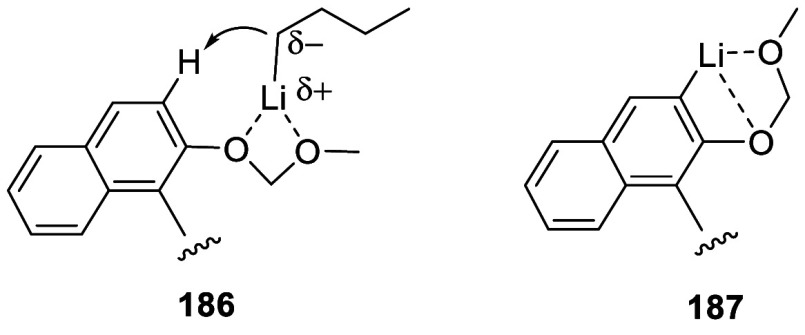


**Scheme 60:**
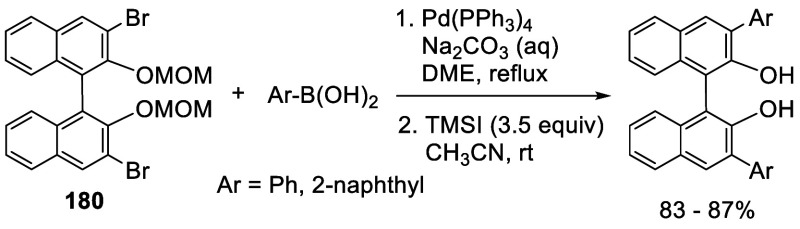
Conversion of 3,3′-Br_2_BINOL-MOM to 3,3′-Diaryl BINOLs

3,3′-Br_2_BINOL-MOM was used to make other BINOL derivatives.^95,228−230,232,233,260,262^ For example, the Pd-catalyzed Suzuki coupling of the dibromoBINOL-MOM **180** with aryl boronic acids followed by removal of the MOM groups with TMSI gave the 3,3′-diarylBINOLs (Scheme 60).^228^

**Scheme 61:**
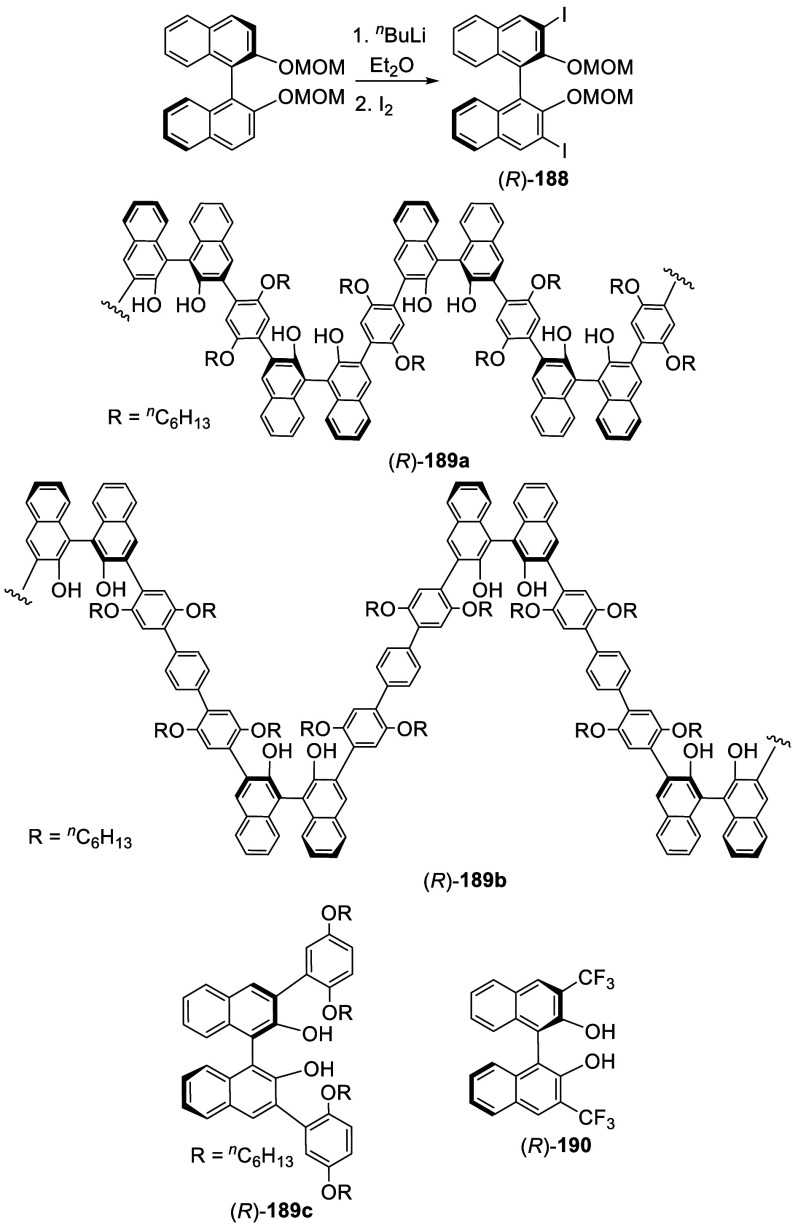
Diiodonation of (*R*)-BINOL-MOM by Ortho-lithiation

Addition of I_2_ following the ortho-lithiation of BINOL-MOM with 2.5 equiv of ^*n*^BuLi in Et_2_O at room temperature gave 3,3′-diiodoBINOL-MOM, **188**, in 71% yield (Scheme 61).^228^ The optically active (*R*)-**188** was obtained similarly for various applications.^114,229,230,234−251^ For example, it underwent the Suzuki coupling with aryl diboronic acids to generate the polymers (*R*)-**189a**,**b** and the monomer (*R*)-**189c** to catalyze the asymmetric reaction of alkylzincs with aldehydes,^234−236^ the asymmetric epoxidation of enones,^237^ and the asymmetric reduction of ketones.^238^ Coupling of (*R*)-**188** with ArB(OH)_2_ followed by deprotection with Amberlyst 15 in refluxing CH_3_OH/THF gave various (*R*)-3,3′-diarylBINOLs.^229^ (*R*)-**188** was also used to react with FSO_2_CF_2_CO_2_Me in the presence of CuI to give (*R*)-3,3′-di(trifluoromethyl) BINOL, (*R*)-**190**, after deprotection. These compounds were used for the asymmetric allyboronate addition to aldehydes and ketones.^229^

#### 7.4.2 Borylation

**Scheme 62:**
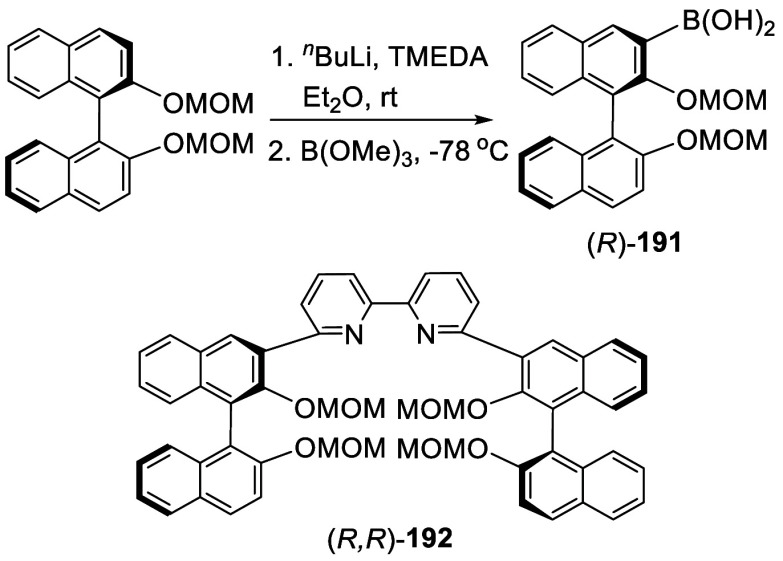
Monoborylation of (*R*)-BINOL-MOM by Ortho-lithiation

Reaction of (*R*)-BINOL-MOM with 1.5 equiv of ^*n*^BuLi and TMEDA in Et_2_O at room temperature followed by the addition of B(OMe)_3_ at −78 °C and acidic workup gave the monoboronic acid (*R*)-**191** as the major product in 55% yield (Scheme 62).^111^ The diboronic acid minor product was removed by column chromatography on silica gel. The BINOL monoboronic acid was used to make other BINOL derivatives.^111,252−254,336^ For example, the Suzuki coupling reaction of (*R*)-**191** was used to prepare bisBINOL compounds such as (*R*,*R*)-**192**.^111^

**Scheme 63:**
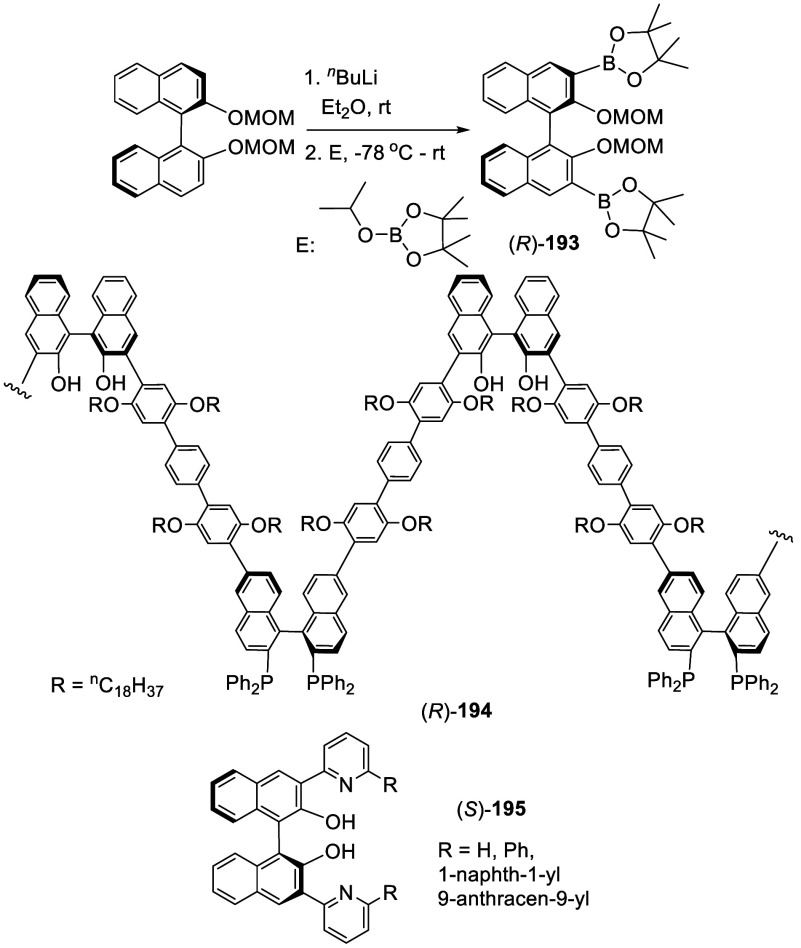
Diborylation of (*S*)-BINOL-MOM by Ortho-lithiation

The ortho-lithiated (*S*)-BINOL-MOM was reacted with 2-isopropyl-4,4,5,5-tetramethyl-1,3,2-dioxaborolane to give the 3,3′-diboronic ester (*R*)-**193** in 75% yield (Scheme 63).^255^ This compound was used to prepare various 3,3′-substituted compounds by the Suzuki coupling reactions.^255−264^ For example, a BINOL-BINAP copolymer (*R*)-**194** was synthesized from the Suzuki coupling of (*R*)-**193** with a BINAP monomer and an aryl diiodide for the tandem asymmetric alkyl addition to aldehydes and asymmetric hydrogenation of ketones.^255^ Compound (*S*)-**195** was prepared from the coupling reaction of (*S*)-**193** with 2-bromopyridine,^256^ which was used to catalyze the asymmetric reaction of ZnEt_2_ with aldehydes.^239^

**Scheme 64:**
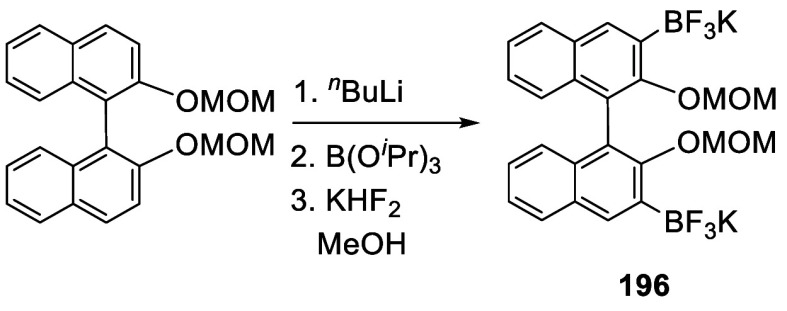
Trifluoroborylation of BINOL-MOM by Ortho-lithiation

Trifluoroborates of BINOL-MOM were prepared. Treatment of BINOL-MOM with ^*n*^BuLi followed by the addition of B(O^*i*^Pr)_3_ and then KHF_2_ gave **196** in 99% yield (Scheme 64).^265^ The trifluoroborate groups served as the protected boronic acids for use in the cross-coupling reactions to prepare the 3,3′-dipyridyl compounds such as (*S*)-**195**.

**Scheme 65:**
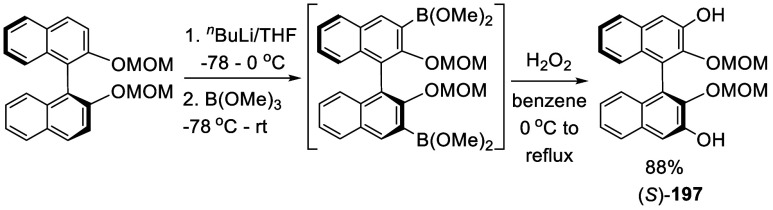
Ortho-lithiation Borylation Oxidation of (*S*)-BINOL-MOM

3,3′-Dihydroxy BINOL was prepared from ortho-lithiation followed by borylation and oxidation. As shown in Scheme 65, after ortho-lithiation of (*S*)-BINOL-MOM with ^*n*^BuLi in THF, B(OMe)_3_ was added to give a 3,3′-diborylated compound. Without isolation of this diborylated compound, it was oxidized by H_2_O_2_ (30% aq) to give (*S*)-**197** in 88% yield.^231^

#### 7.4.3 Zincation

**Scheme 66:**
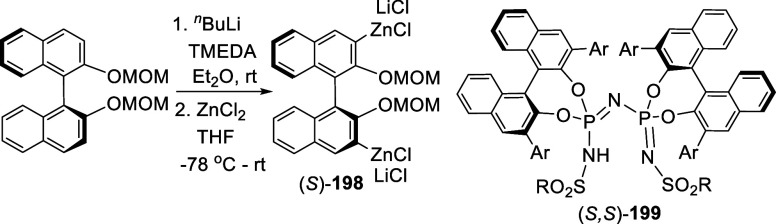
Ortho-lithiation- Zincation of (*R*)-BINOL-MOM

When the ortho-lithiated (*S*)-BINOL-MOM was reacted with ZnCl_2_, the arylzinc product (*S*)-**198** was obtained in 93% yield (by ^1^H NMR analysis) (Scheme 66).^266^ The stock solution of (*S*)-**198** in THF was stored at −20 °C under argon for subsequent cross coupling reactions with aryl bromides, followed by further conversions to make the 3,3′-diarylBINOL-based catalysts like (*S*,*S*)-**199** for the asymmetric Diels–Alder reactions of α,β-unsaturated esters.

#### 7.4.4 Silylation

**Scheme 67:**
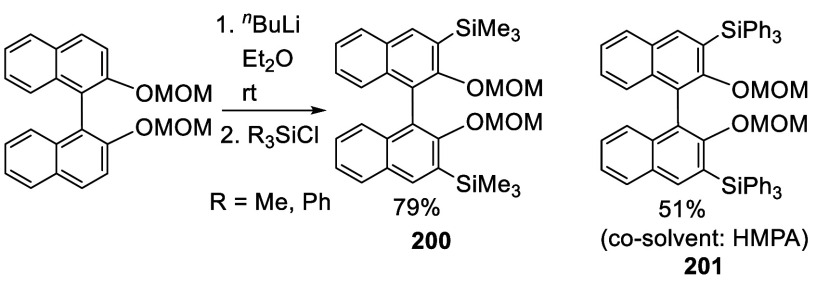
Ortho-lithiation Silylation of BINOL-MOM

When BINOL-MOM was treated with 3 equiv of ^*n*^BuLi in Et_2_O at room temperature for 3 h, followed by the addition of R_3_SiCl, the 3,3′-disilyl products **200** and **201** were obtained in 79% and 51% yields, respectively (Scheme 67). In the reaction of Ph_3_SiCl to form **201**, HMPA was added as a cosolvent.^228^

The 3,3′-disilyl BINOLs such as (*R*)-**202a**–**d** were prepared in a similar way after removal of the MOM groups and used for asymmetric catalysis.^102,267−275^ For example, these compounds in combination with AlMe_3_ were used to catalyze the asymmetric hetero-Diels–Alder reaction of enamide aldehydes with Danishefsky’s diene.^102^
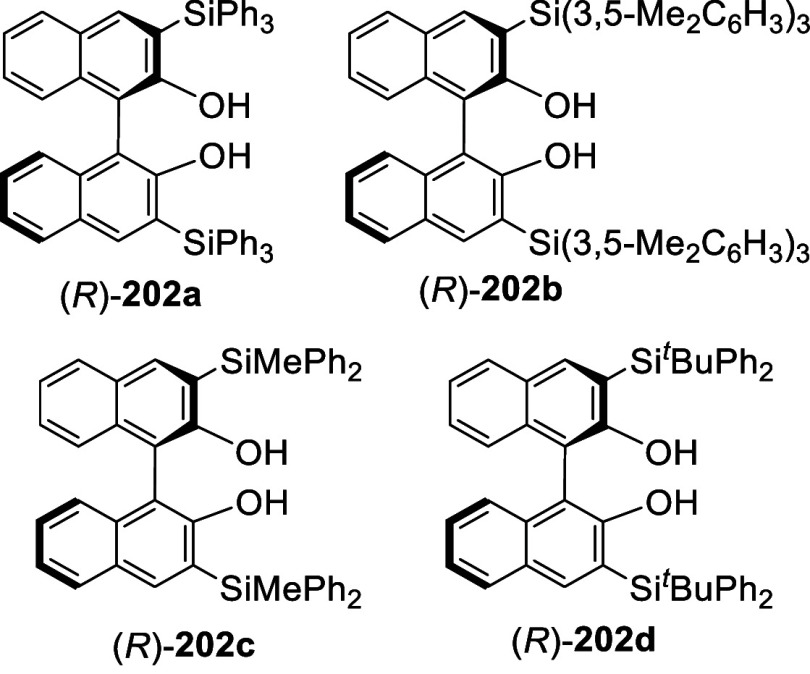


#### 7.4.5 Thiolation and Selenation

**Scheme 68:**
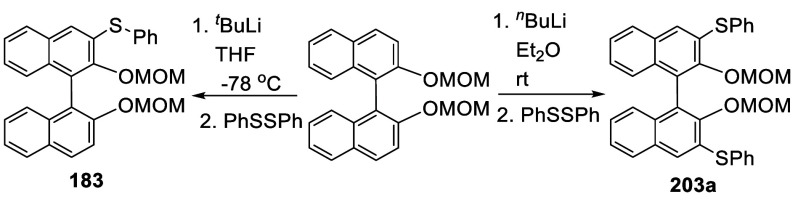
Ortho-lithiation Thiolation of BINOL-MOM

When BINOL-MOM was treated with 2.2 equiv of ^*t*^BuLi in THF at −78 °C for 1 h followed by the addition of PhSSPh, the monothiolated product **183** was obtained in 71% yield (Scheme 68).^228^ When BINOL-MOM was treated with 3 equiv of ^*n*^BuLi in Et_2_O at room temperature for 3 h followed by the addition of PhSSPh, the dithiolated product **203a** was obtained in 89% yield.^228^

The optically active 3,3′-dithiolated compounds (*S*)-**203a**–**c** were obtained by ortho-lithiation of (*S*)-BINOL-MOM with ^*n*^BuLi in Et_2_O followed by treatment with various RSSR.^276,277^ When the electrophile was MeSeSeMe, the 3,3′-diselenate (*S*)-**204** was obtained.^276^ These compounds were deprotected by reaction with HCl (37%) in methanol at room temperature to give the deprotected products (*S*)-**205a**–**c** and (*S*)-**206**. They were used in combination with [Cu(MeCN)_4_BF_4_ to catalyze the Michael addition of dialkylzincs to enones.^276^ The undeprotected compound (*S*)-**203b** was used for the asymmetric Simmons–Smith-mediated epoxidation.^277^ The 3,3′-dimercapto compound (*R*)-**207** was obtained in 85% yield from the reaction of the ortho-lithiated (*R*)-BINOL-MOM with S_8_ followed by deprotection.^278^ This compound was used to catalyze the asymmetric intramolecular Morita–Baylis–Hillman and Rauhut–Currier reactions.
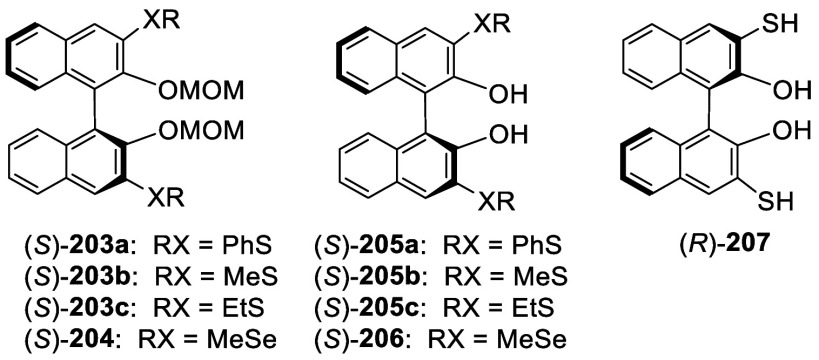


#### 7.4.6 Azidation

**Scheme 69:**
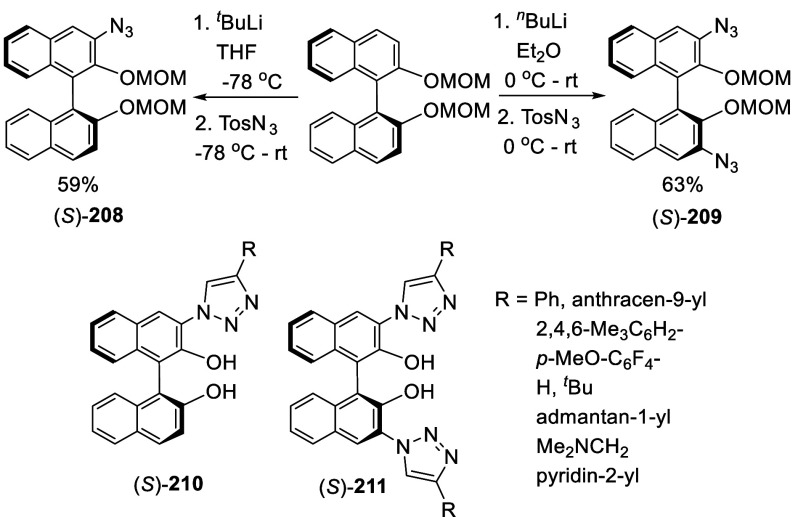
Ortho-lithiation Azidation of (*S*)-BINOL-MOM

Azide groups were incorporated into BINOL by ortho-lithiation. Treatment of (*S*)-BINOL-MOM with ^*t*^BuLi at −78 °C in THF followed by the addition of TosN_3_ gave the monoazide product (*S*)-**208** in 59% yield (Scheme 69).^279^ Reaction of (*S*)-BINOL-MOM with ^*n*^BuLi in Et_2_O at 0 °C to room temperature followed by the addition of TosN_3_ gave the diazide (*S*)-**209** in 63% yield. The “click” cyclization of these compounds with a variety of terminal alkynes were conducted to give compounds (*S*)-**210** and (*S*)-**211**. These compounds were used in combination with Ti(O^*i*^Pr)_4_ to catalyze the reaction of ZnEt_2_ with aldehydes.

#### 7.4.7 Phosphorylation

**Scheme 70:**
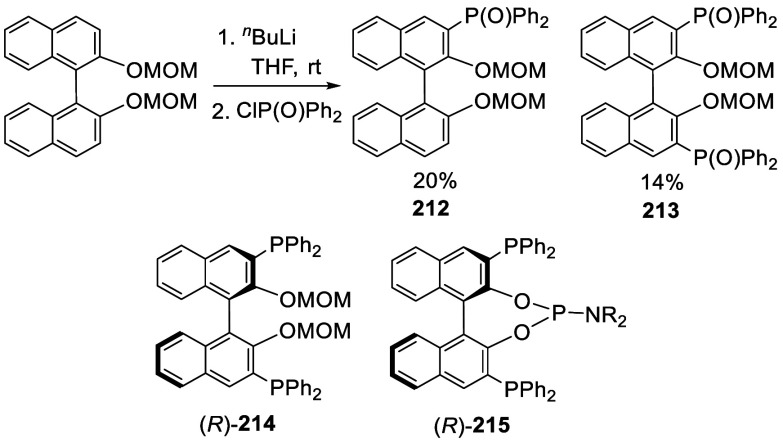
Ortho-lithiation Phosphorylation of BINOL-MOM

Ortho-lithiation of BINOL-MOM with ^*n*^BuLi in THF solution at room temperature followed by the addition of diphenylphosphinic chloride gave the mono and disubstituted products **212** and **213** (Scheme 70).^212^ (*R*)-**214** was obtained in 70% yield from the reaction of (*R*)-BINOL-MOM with ^*n*^BuLi in Et_2_O/THF followed by treatment with Ph_2_PCl.^280^ After removal of the MOM groups of (*R*)-**214** with HCl/MeOH, it was then converted to (*R*)-**215**, which in combination with [Rh(cod)_2_]BF_4_ was used to catalyze the asymmetric hydrogenation of aryl enamides.

#### 7.4.8 Alkylation

**Scheme 71:**
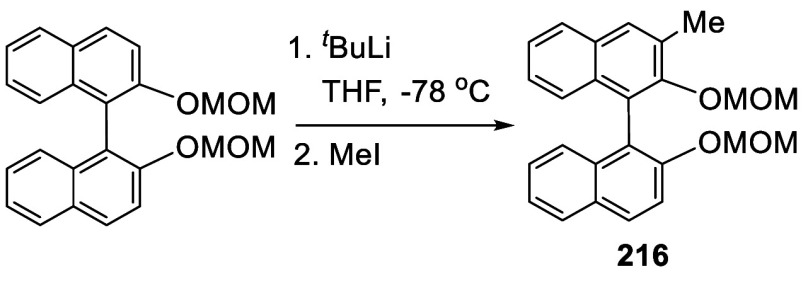
Ortho-lithiation Methylation of BINOL-MOM

**Scheme 72:**
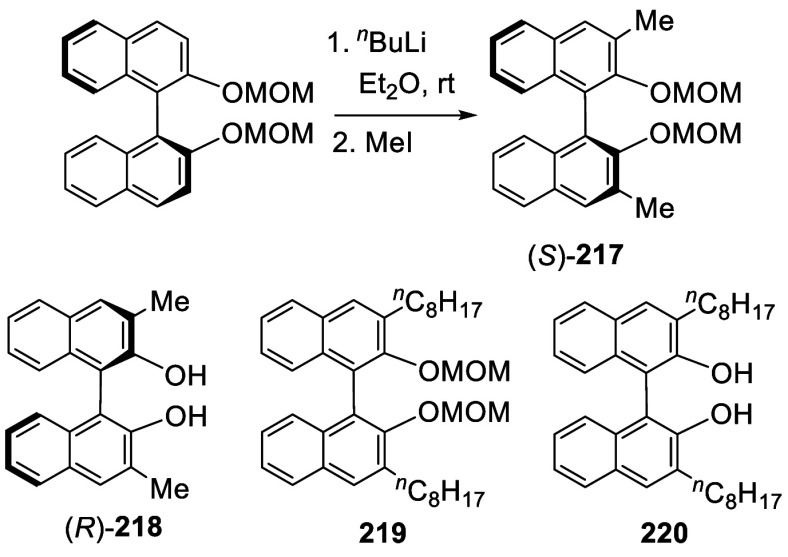
Ortho-lithiation Dimethylation of (*S*)-BINOL-MOM

When BINOL-MOM was treated with 2.2 equiv of ^*t*^BuLi in THF at −78 °C for 1 h, followed by the addition of MeI, the monomethylated product **216** was obtained in 71% yield (Scheme 71).^228^ When (*S*)-BINOL-MOM was treated with 3 equiv of ^*n*^BuLi in Et_2_O at room temperature for 3 h, followed by the addition of MeI, the 3,3′-dimethylated product (*S*)-**217** was obtained in 82% yield (Scheme 72). Retention of the chiral configuration of BINOL in this reaction was established.^228^ (*R*)-**217** was obtained in 96% yield by a slightly modified procedure.^229,260,270^ After deprotection with Amberlyst 15, the resulting compound (*R*)-**218** was used for the asymmetric allyboronate addition to aldehydes. The ortho-lithiated racemic BINOL-MOM was reacted with 1-bromooctane to give **219** in 81.9% yield.^230^ Treatment of **219** in THF with HCl (concentrated) removed the MOM groups to give **220** in >99% yield without the need for further purification.

**Scheme 73:**
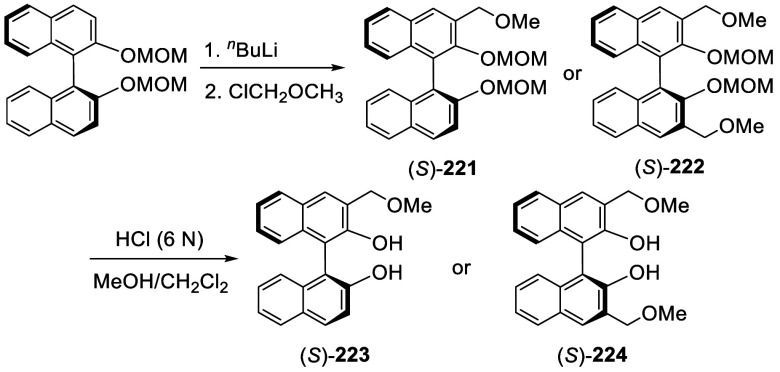
Methoxymethylation of BINOL-MOM by Ortho-lithiation

When (*S*)-BINOL-MOM was ortho-lithiated with 1.2 equiv and 1.9 equiv of ^*n*^BuLi followed by reaction with chloromethyl methyl ether, the 3-mono- and 3,3′-di(methoxymethyl)-substituted products (*S*)-**221** and (*S*)-**222** were obtained in 57% and 80% yields, respectively (Scheme 73).^281^ After removal of the protecting groups with 6 N HCl in MeOH/CH_2_Cl_2_, the resulting compounds (*S*)-**223** and (*S*)-**224** in combination with Ti(O^*i*^Pr)_4_ were used to catalyze the asymmetric reaction of ZnEt_2_ with aryl aldehydes.

#### 7.4.9 Addition to Ethylene Oxide and Propylene Oxide

**Scheme 74:**
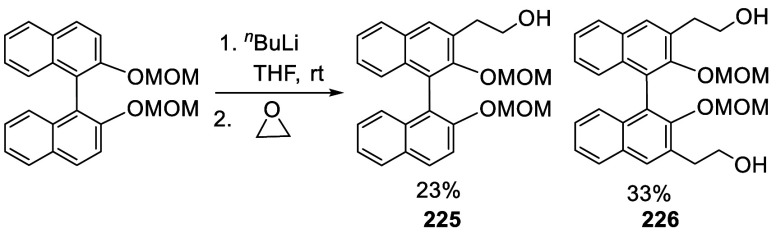
Addition of the Ortho-lithiated BINOL-MOM to Ethylene Oxide

Ortho-lithiation of BINOL-MOM with ^*n*^BuLi in THF solution at room temperature followed by the addition of ethylene oxide gave the mono- and disubstituted products **225** and **226** (Scheme 74).^212^

**Scheme 75:**
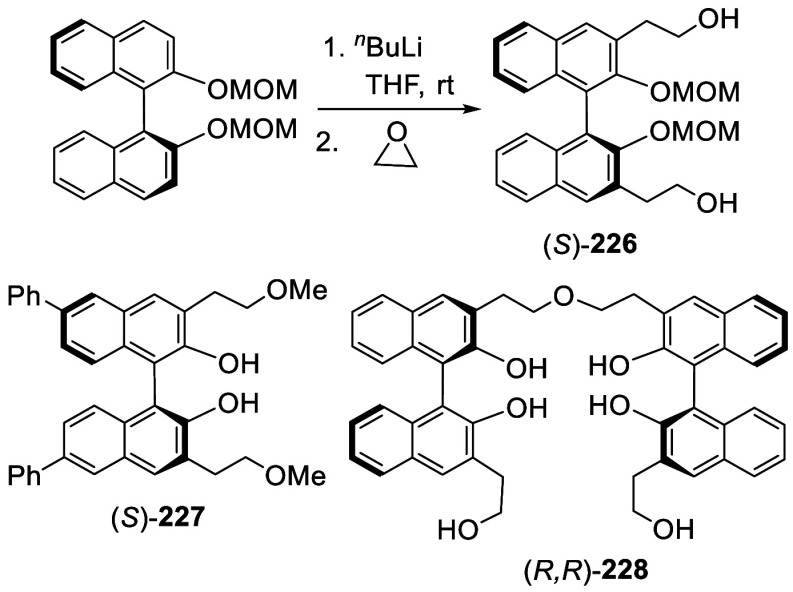
Addition of the Ortho-lithiated (*S*)-BINOL-MOM to Ethylene Oxide

The reaction of the ortho-lithiated (*S*)-BINOL-MOM with ethylene oxide was further investigated to synthesize (*S*)-**226** (Scheme 75). After the starting material was treated with 3 equiv of ^*n*^BuLi in THF at room temperature for 8 h, 50 equiv of ethylene oxide was added at −78 °C and the reaction mixture was allowed to slowly warm up to 0 °C before workup to give (*S*)-**226** in 56% yield.^282^ Addition of ethylene oxide and BF_3_·OEt_2_ at −96 °C after ortho-lithiation of (*R*)-BINOL-MOM in Et_2_O solution gave (*R*)-**226** in 50% yield.^283^ (*S*)- and (*R*)-**226** were used to prepare compounds such as (*S*)-**227**^282^ and (*R*,*R*)-**228**,^283^ which in combination with La(III) were used to catalyze the asymmetric reaction of dialkyl phosphites with aldehydes (Pudovik reaction)^282^ and the aldol reactions.^283^

**Scheme 76:**
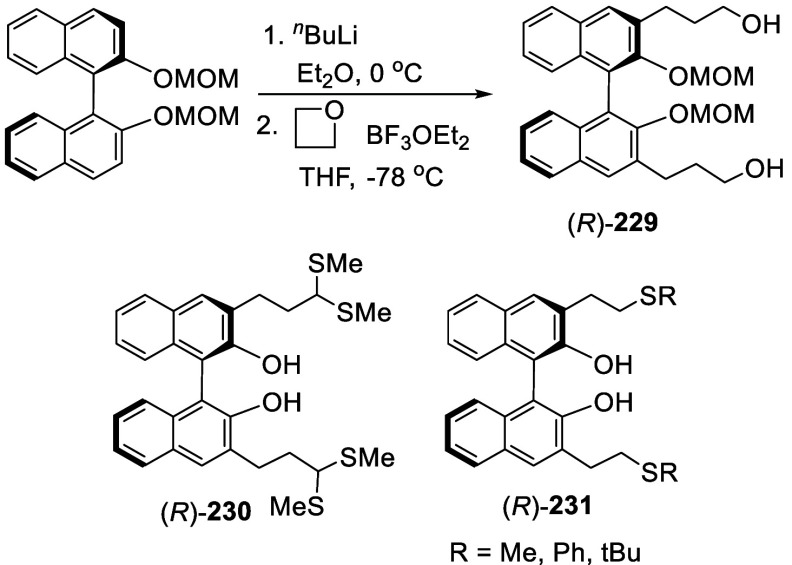
Addition of the Ortho-lithiated (*S*)-BINOL-MOM to Propylene Oxide

Ortho-lithiation of (*R*)-BINOL-MOM followed by reaction with propylene oxide gave the 3,3′-bis(3-hydroxylpropyl)BINOL, (*R*)-**229**, in 51% yields (Scheme 76).^284^ This compound was converted to the thioacetal (*R*)-**230**. The thiolated compounds (*R*)-**231** were prepared from (*R*)-**226**. These chiral sulfur-containing compounds were used in combination with CuX_2_ (X = OAc, OTf, etc.) to catalyze the asymmetric Michael addition of ZnEt_2_ to enones and nitroalkenes.

#### 7.4.10 Addition to Aldehydes

**Scheme 77:**
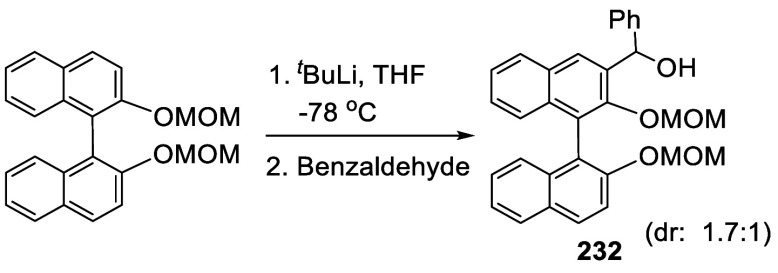
Addition of the Ortho-lithiated BINOL-MOM to Benzaldehyde

When BINOL-MOM was treated with 2.2 equiv of ^*t*^BuLi in THF at −78 °C for 1 h followed by the addition of benzaldehyde, the monoaddition product **232** was obtained as a 1.7:1 mixture of diastereomers in 58% yield (Scheme 77).^228^

**Scheme 78:**
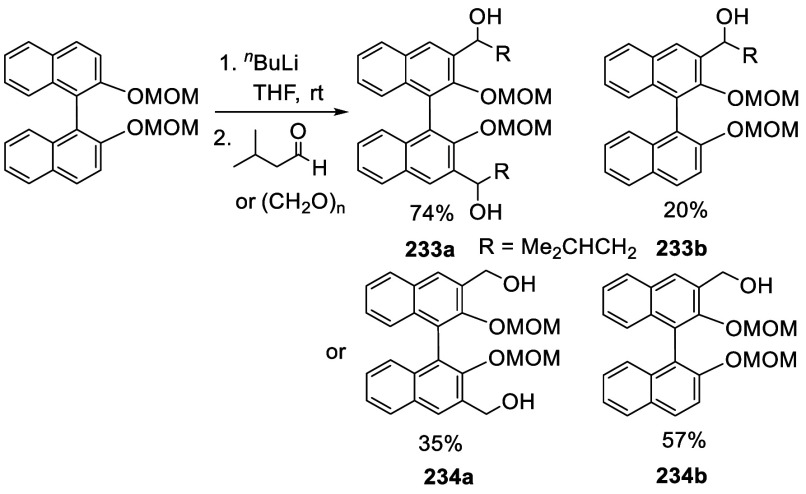
Addition of the Ortho-lithiated BINOL-MOM to Isovaleraldehyde and Paraformaldehyde

Ortho-lithiation of BINOL-MOM with ^*n*^BuLi in THF solution at room temperature followed by the addition of isovaleraldehyde and paraformaldehyde gave the mono- and disubstituted products **233a**,**b** or **234a**,**b** (Scheme 78).^212^ The MOM groups of the products were removed by reaction with 10% HCl (aq) in methanol solution for 3 h.

**Scheme 79:**
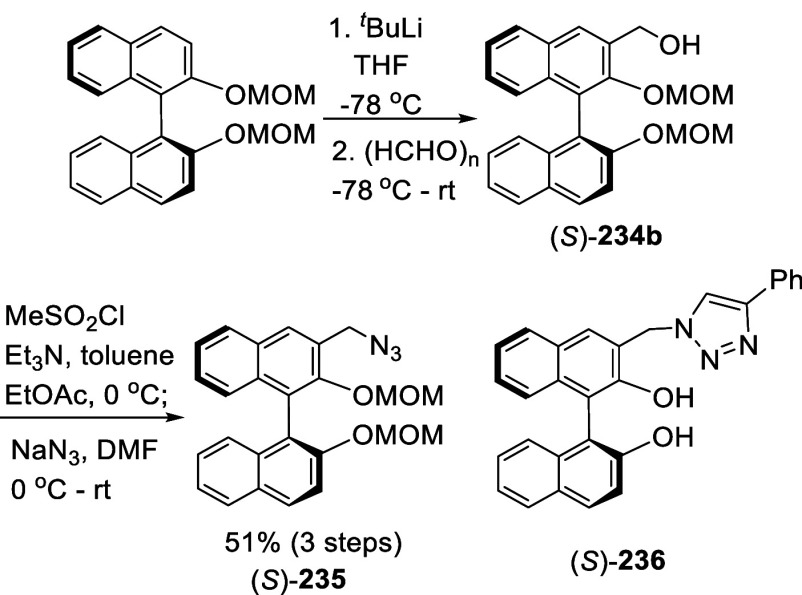
Addition of the Ortho-lithiated (*S*)-BINOL-MOM to Paraformaldehyde

The optically active compound (*S*)-**234b** was prepared from the reaction of (*S*)-BINOL-MOM with ^*t*^BuLi in THF at −78 °C followed by the addition of paraformaldehyde (Scheme 79).^279^ (*S*)-**234b** was used to make the azide compound (*S*)-**235**, which was subjected to the “click” cyclization with phenyl acetylene to make compound (*S*)-**236**. This compound was used in combination with Ti(O^*i*^Pr)_4_ to catalyze the asymmetric reaction of ZnEt_2_ with benzaldehyde.

**Scheme 80:**
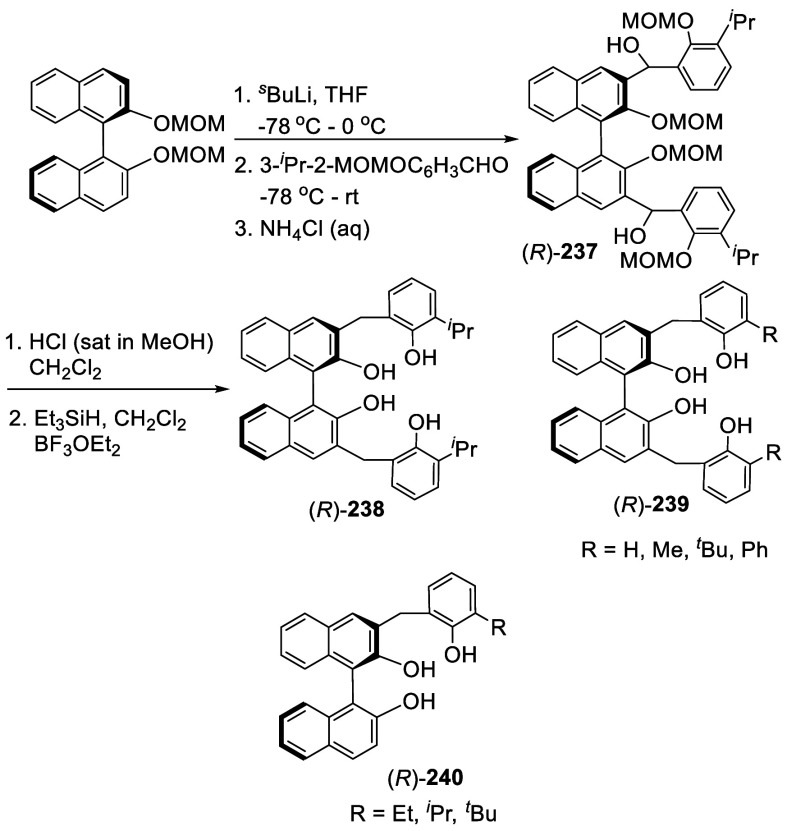
Addition of the Ortho-lithiated (*R*)-BINOL-MOM to a Ketone

Addition of the ortho-lithiated (*R*)-BINOL-MOM to an aromatic aldehyde gave (*R*)-**237** as a mixture of diastereomers because of the newly formed chiral alcohol centers (Scheme 80).^285^ (*R*)-**237** was converted to (*R*)-**238** by deprotection and Et_3_SiH reduction. This tetraalcohol product in combination with Nb(OMe)_5_ was used to catalyze the enantioselective desymmetrization of *meso*-epoxides with aromatic amines. Additional compounds (*R*)-**239** with R = H, Me, ^*t*^Bu, and Ph were also prepared and investigated for asymmetric catalysis.^286^ The monosubstituted BINOLs (*R*)-**240** were synthesized in a similar way by using 1.2 equiv of ^*n*^BuLi for the ortho-lithiation of (*R*)-BINOL-MOM. These compounds in combination with Nb(OR)_5_ (R = alkyl) were used to catalyze the asymmetric Mannich-type reaction of imines with silyl enolates.^287^

**Scheme 81:**
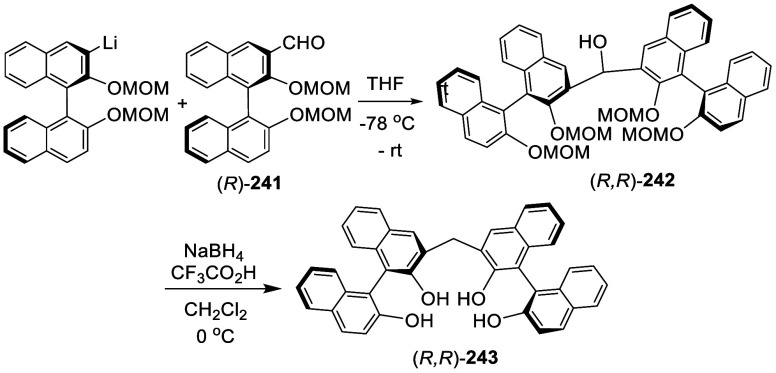
Addition of the Ortho-lithiated (*R*)-BINOL-MOM to (*R*)-3-FormylBINOL-MOM

When the ortho-lithiated (*R*)-BINOL-MOM was added to (*R*)-3-formylBINOL-MOM, (*R*)-**241**, the bisBINOL-MOM compound (*R*,*R*)-**242** was obtained in 62% yield (Scheme 81).^288^ Reduction of (*R*,*R*)-**242** with NaBH_4_ in the presence of CF_3_CO_2_H in CH_2_Cl_2_ gave the bisBINOL (*R*,*R*)-**243** in 89% yield. This bisBINOL compound in combination with Zr(O^*t*^Bu)_4_ was used to catalyze the asymmetric Mannich-type reaction of imines with ketene silyl acetals.

#### 7.4.11 Addition to Ketones

**Scheme 82:**
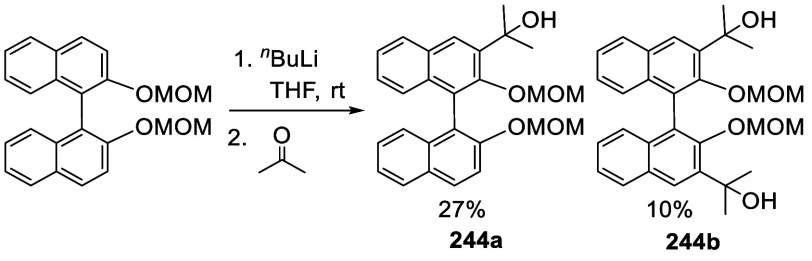
Addition of the Ortho-lithiated BINOL-MOM to Acetone

Reaction of BINOL-MOM with ^*n*^BuLi in THF solution at room temperature followed by the addition of acetone gave the mono- and disubstituted products **244a** and **244b** in 27% and 10% yields, respectively (Scheme 82).^212^

**Scheme 83:**
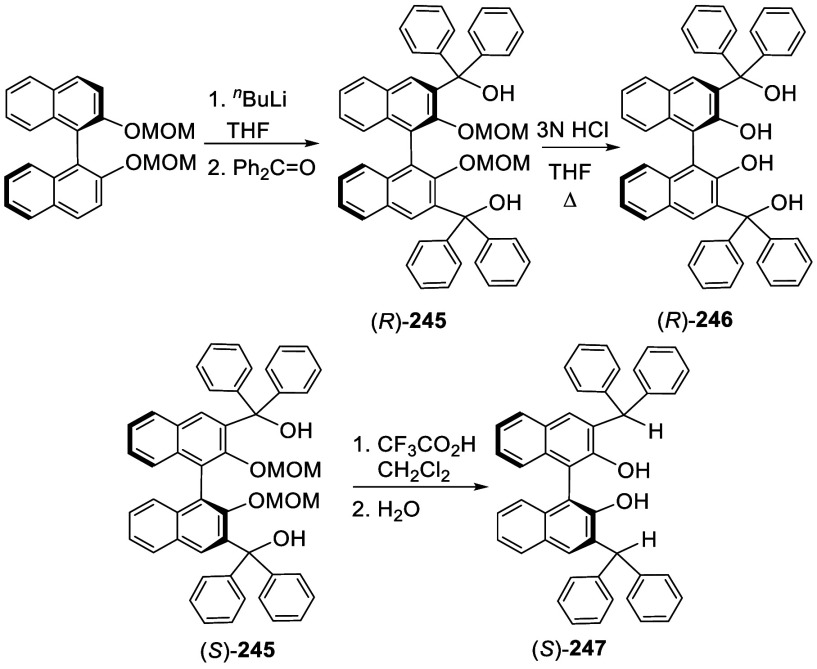
Addition of the Ortho-lithiated (*R*)-BINOL-MOM to Benzophenone

When the ortho-lithiated (*R*)- or (*S*)-BINOL-MOM was reacted with benzophenone, compounds (*R*)-**245** or (*S*)-**245** were obtained in 91–95% yields (Scheme 83).^213,240,289^ Deprotection of the MOM groups of (*R*)-**245** with 3 N HCl in THF gave the tetrahydroxyl product (*R*)-**246** in 90% yield.^162^ This compound was used for enantioselective fluorescent recognition of chiral amino alcohols. When (*S*)-**245** was treated with CF_3_CO_2_H in CH_2_Cl_2_, besides the removal of the MOM protecting groups, the two diphenylmethanol units were also reduced to diphenylmethane to give (*S*)-**247** in 88% yield.^213^ This compound was used to catalyze the asymmetric reaction of ZnEt_2_ with aldehydes.

**Scheme 84:**
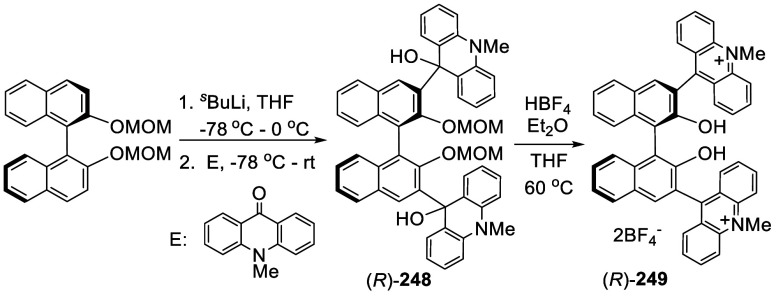
Addition of the Ortho-lithiated (*R*)-BINOL-MOM to 10-Methyl-acridin-9(10*H*)-one

Ortho-lithiation of (*R*)-BINOL-MOM followed by the addition of 10-methyl-acridin-9(10*H*)-one gave (*R*)-**248** (Scheme 84).^290^ Treatment of (*R*)-**248** with HBF_4_ gave the dication (*R*)-**249** in 63% yield over the two steps. This compound was used as a visible light induced photoacid to catalyze the synthesis of 2-deoxyglycosides from glycals.

#### 7.4.12 Addition to Esters

**Scheme 85:**
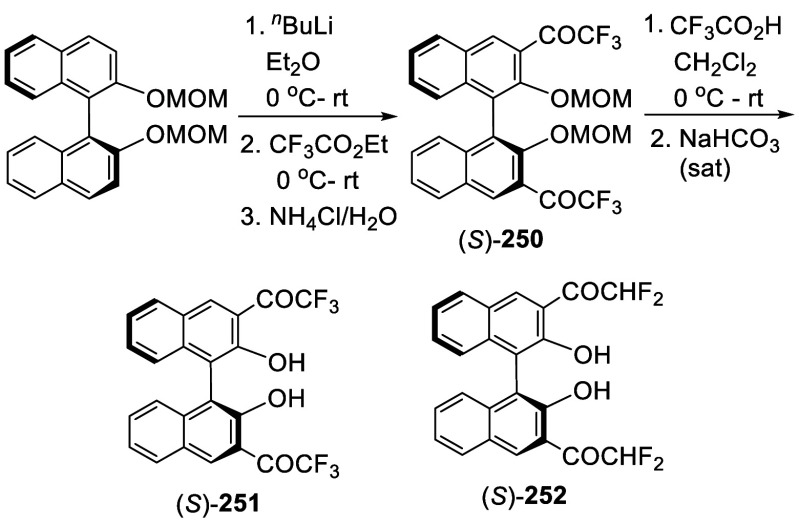
Addition of the Ortho-lithiated (*S*)-BINOL-MOM to Ethyl Trifluoroacetate

Reaction of the ortho-lithiated (*S*)-BINOL-MOM with ethyl trifluoroacetate gave the BINOL-derived trifluoromethyl ketone (*S*)-**250** in 62% yield (Scheme 85).^291^ Deprotection of (*S*)-**250** was conducted by using CF_3_CO_2_H in CH_2_Cl_2_ at 0 °C to room temperature to give (*S*)-**251** in 84% yield. The use of CF_3_CO_2_H avoided the reflux conditions in the use of HCl/EtOH. (*S*)-**251** showed different fluorescence responses toward a chiral diamine at two different emission wavelengths and was used to simultaneously determine the concentration and enantiomeric composition of the chiral substrates. Compound (*S*)-**252** was obtained in a similar way by using (*S*)-BINOL-MOM and ethyl difluoroacetate.^292^

#### 7.4.13 Addition to Acyl Chlorides

**Scheme 86:**
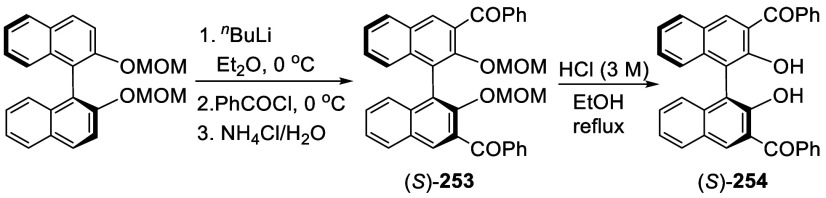
Addition of the Ortho-lithiated (*R*)-BINOL-MOM to Benzoyl Chloride

After (*S*)-BINOL-MOM was treated with 5 equiv of ^*n*^BuLi in Et_2_O at 0 °C for 3 h, addition of 5 equiv of benzoyl chloride gave (*S*)-**253** in 84% yield upon acidic workup (Scheme 86).^214^ The MOM groups of (*S*)-**253** were removed by heating in HCl (3N, aq) and ethanol at reflux to give (*S*)-**254** in 86% yield. Enantioselective fluorescence quenching by N-Boc amino carboxylates, amino alcohols, and mandelate was investigated.

**Scheme 87:**
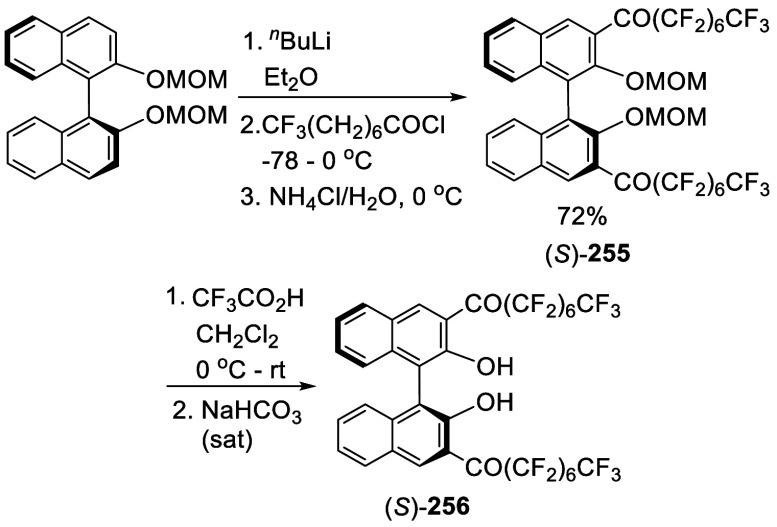
Addition of the Ortho-lithiated (*R*)-BINOL-MOM to Perfluorooctanoyl Chloride

A perfluoroalkyl ketone (*S*)-**255** was obtained in 72% yield by treatment of (*S*)-BINOL-MOM with ^*n*^BuLi at room temperature followed by reaction with perfluorooctanoyl chloride at −78 to 0 °C (Scheme 87).^293^ Deprotection of (*S*)-**255** was conducted by using CF_3_CO_2_H in CH_2_Cl_2_ at 0 °C to room temperature to give (*S*)-**256** in 85% yield. (*S*)-**256** showed enantioselective fluorescence enhancement in the presence of amino alcohols in the fluorous phase.

**Scheme 88:**
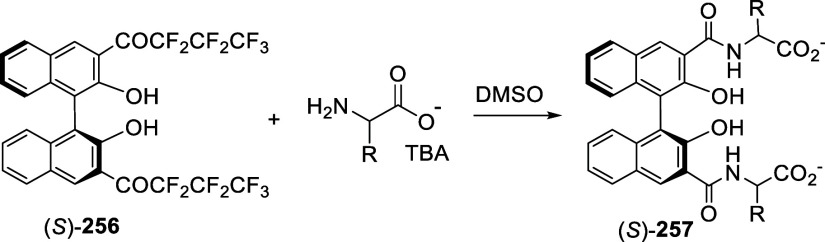
Nucleophilic Substitution of a Perfluoroalkyl BINOL Ketone with Amino Acid Salts to Form Amides

Compound (*S*)-**256** with a shorter perfluoroalkyl group was prepared in a similar way. It reacted with amino acid TBA salts (TBA = ^*n*^Bu_4_N^+^) in DMSO at room temperature to give the amides (*S*)-**257** with enantioselective fluorescence enhancement (Scheme 88).^294^

#### 7.4.14 Addition to DMF

**Scheme 89:**
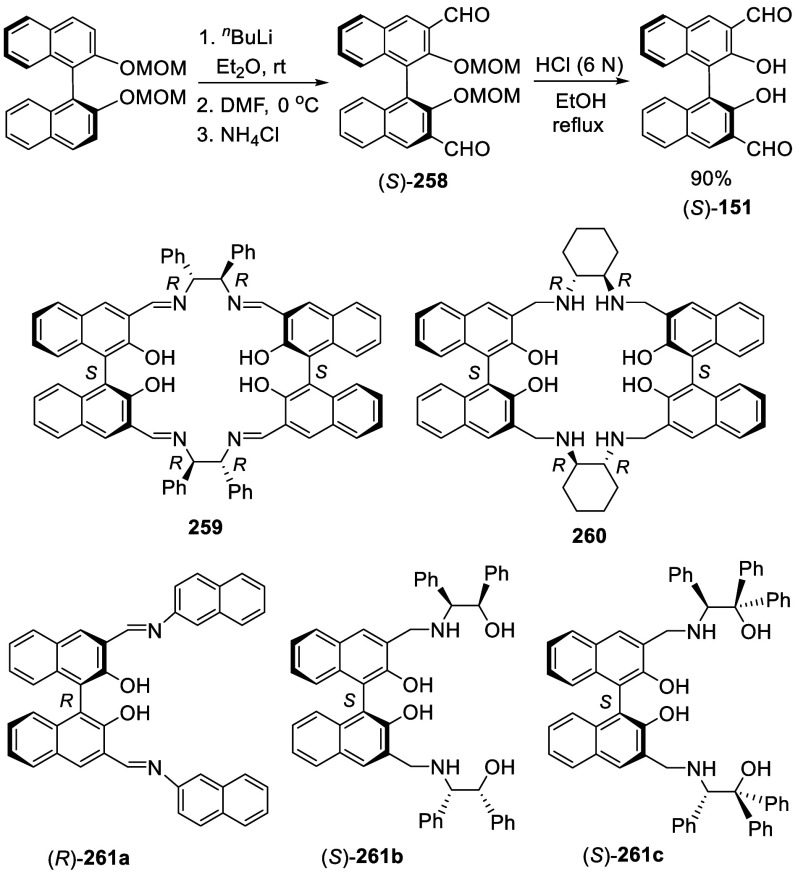
Addition of the Ortho-lithiated (*S*)-BINOL-MOM to DMF

Ortho-lithiation of (*S*)-BINOL-MOM with ^*n*^BuLi and TMEDA in refluxing Et_2_O followed by reaction with DMF gave (*S*)-**258** in 78% yield (Scheme 89).^216^ It was later found that the addition of TMEDA and the refluxing conditions were not necessary. Ortho-lithiation of (*S*)-BINOL-MOM with ^*n*^BuLi in Et_2_O at room temperature in the absence of TMEDA followed by the reaction with DMF gave (*S*)-**258** in 68% yield.^295^ The MOM protecting groups of (*S*)-**258** were removed by treatment with HCl (6 N) in refluxing ethanol solution to give (*S*)-3,3′-diformylBINOL, (*S*)-**151**, in 90% yield. Polymerization of (*S*)-**151** with diamines in the presence of Ni(II) was studied.^295^ In an earlier study, (*S*)-**151** was obtained from the resolution of BINOL 3,3′-dicarboxylic acid followed by reduction^296^ and was found to react with (*R*,*R*)-1,2-diphenyletnylenediamine to form the [2 + 2] macrocycle **259** but with (*S*,*S*)-1,2-diphenylethylenediamine to give polymers.^297^ Macrocycle **260** was prepared from the reaction of (*S*)-**151** with (*R*,*R*)-1,2-diaminocyclohexane followed by reduction with NaBH_4_.^298^ This compound was used to carry out the enantioselective fluorescent recognition of mandelic acid, an α-hydroxyl carboxylic acid. It was also used for the fluorescent recognition of Hg^2+^.^299^ (*S*)- or (*R*)-**151** in combination with Zn(OAc)_2_ was used to conduct the enantioselective fluorescent recognition of chiral functional amines, including diamines, amino alcohols, and amino acids.^300−302^ Compound (*R*)-**261a** was prepared from the condensation of (*R*)-**151** with 2-naphthylamine and used as a dual responsive fluorescent probe to simultaneously determine the concentration and enantiomeric composition of chiral functional amines.^301^ Compounds (*S*)-**261b** and (*S*)-**261c** were prepared from the condensation of (*S*)-**151** with the corresponding chiral amino alcohols, followed by reduction for the enantioselective fluorescent recognition of chiral α-hydroxycarboxylic acids.^303−305^

The 3,3′-diformylBINOLs also served as the precursors to prepare other BINOL derivatives for application in asymmetric catalysis. For example, the sulfur-containing compounds (*R*)-**262a**,**b** were prepared from (*R*)-**151** for the asymmetric reaction of ZnEt_2_ with enones in the presence of Cu(OTf)_2_.^284^ Compound (*S*)-**262c** was prepared from (*S*)-**258** and was used in combination with Ti(O^*i*^Pr)_4_ to catalyze the asymmetric reaction of ZnEt_2_ with benzaldehyde.^279^
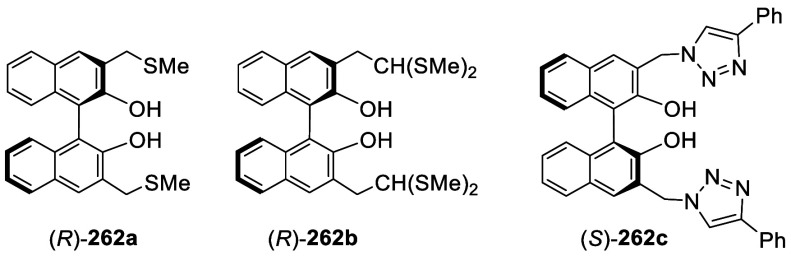


**Scheme 90:**
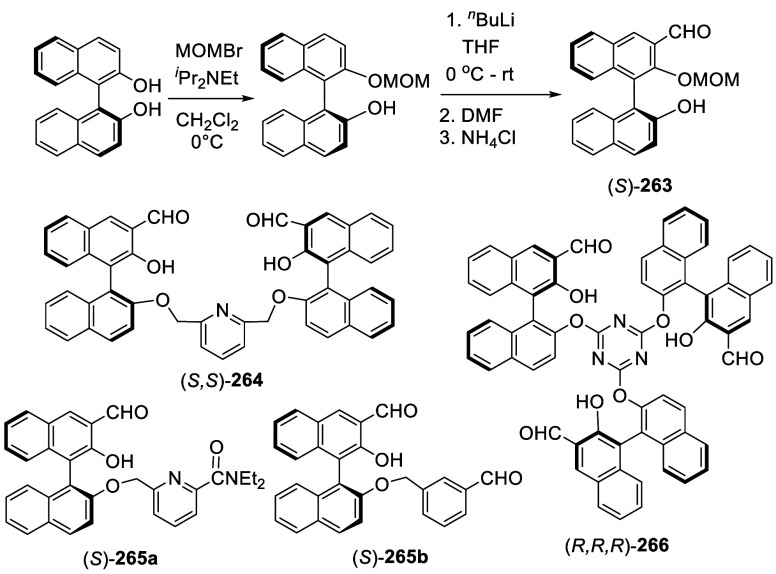
Synthesis of a BINOL Monoaldehyde

A BINOL monoMOM ether was synthesized from the reaction of (*S*)-BINOL with 1.5 equiv of bromomethyl methyl ether in the presence of diisopropylethylamine (Scheme 90).^306^ Treatment of the THF solution of the BINOL monoMOM ether with 3.2 equiv of ^*n*^BuLi at 0 °C to room temperature followed by the addition of DMF gave the monoaldehyde compound (*S*)-**263** in 62% yield over the three steps (Scheme 90). This compound and its enantiomer served as the precursor to a variety of BINOL-aldehyde-based fluorescent probes such as compounds (*S*,*S*)-**264**,^307^ (*S*)-**265a**,^308^ (*S*)-**265b**,^309^ and (*R*,*R*,*R*)-**266**^310^ for the recognition of amino acids and metal ions.

#### 7.4.15 Addition to CO_2_

**Scheme 91:**
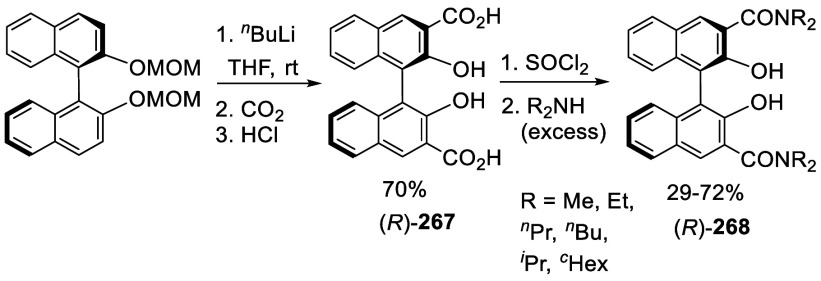
Addition of the Ortho-lithiated (*R*)-BINOL-MOM to CO_2_

BINOL 3,3′-dicarboxylic acid and its derivatives were prepared from the ortho-lithiation of BINOL-MOM. As shown in Scheme 91, after ortho-lithiation with ^*n*^BuLi in THF, CO_2_ was added as the electrophile, which after treatment with HCl in an alcoholic solution gave (*R*)-**267** in 70% yield.^311,312^ The dicarboxylic acid BINOL has been used as the precursor to prepare other BINOL derivatives for various applications.^311−314^ For example, (*R*)-**267** was converted to several amide derivatives (*R*)-**268** with >99% ee. These chiral amides were used to catalyze the Simmons–Smith cyclopropanation of allylic alcohols with ZnEt_2_ and CH_2_I_2_ to give chiral cyclopropanes, and the asymmetric reaction of ZnEt_2_ with propargyl aldehyde.^311,312^

#### 7.4.16 Nucleophilic Reactions with Arenes and Heteroarenes

**Scheme 92:**
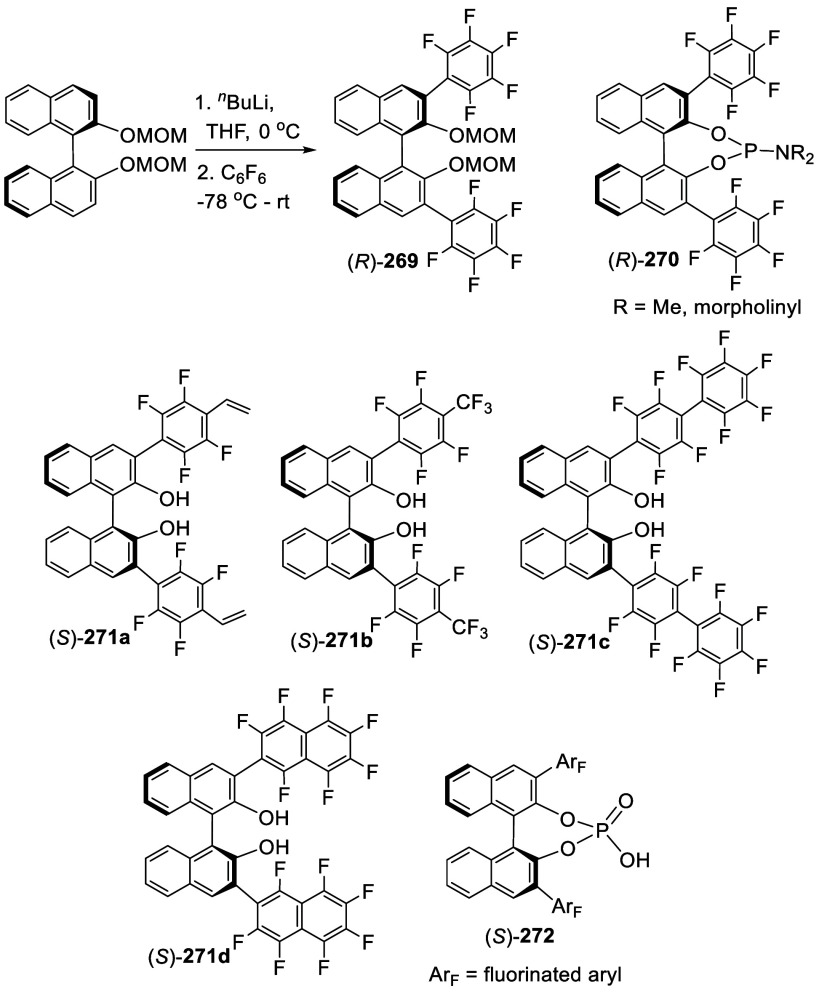
Nucleophilic Substitution of Hexafluorobenzene with the Ortho-Lithiated (*R*)-BINOL-MOM

Reaction of the ortho-lithiated (*R*)-BINOL-MOM with hexafluorobenzene gave the nucleophilic substitution product (*R*)-**269** in 71% yield (Scheme 92).^242,260^ After removal of the MOM groups, it was used to catalyze the asymmetric vinyl boronic acid addition to enones. Compound (*R*)-**270** was prepared from (*R*)-**269** to catalyze the asymmetric ring expansion of cyclic silanes with alkynes in the presence of [Rh(CH_2_=CH_2_)_2_Cl]_2_.^315^ Similar to the synthesis of (*R*)-**269**, compounds (*S*)-**271a**–**d** were synthesized from the reaction of the ortho-lithiated (*S*)-BINOL-MOM with the fluorinated arenes followed by removal of the MOM groups with HCl/MeOH under refluxing.^316^ These compounds were converted to the phosphoric acids (*S*)-**272** to catalyze the asymmetric α-hydroxylation of α-branched cyclic ketones with PhNO.

**Scheme 93:**
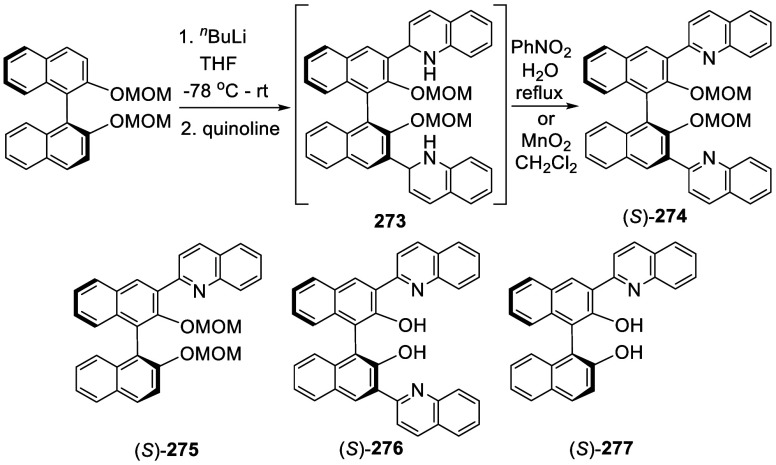
Addition of Ortho-lithiated (*R*)-BINOL-MOM to Quinoline

Nucleophilic addition of the ortho-lithiated (*S*)-BINOL-MOM to quinoline was conducted. After (*S*)-BINOL-MOM was treated with 3 equiv of ^*n*^BuLi in THF at −78 °C to room temperature, it was reacted with quinoline at 0 °C to room temperature to give **273** (Scheme 93).^281^ Addition of nitrobenzene and water followed by heating at reflux converted **273** to (*S*)-**274** in 86% yield in this one-pot reaction. When 1.2 equiv of ^*n*^BuLi was used for the ortho-lithiation, the monosubstituted product (*S*)-**275** was obtained in 55% yield. After removal of the MOM groups with 6 N HCl in MeOH/CH_2_Cl_2_, the resulting compounds (*S*)-**276** and (*S*)-**277** in combination with Ti(O^*i*^Pr)_4_ were used to catalyze the asymmetric reaction of ZnEt_2_ with aryl aldehydes. Oxidation of **273** with MnO_2_ in CH_2_Cl_2_ also gave **274** in 78% yield over the two steps.^114^ This compound was used to make BINOL-phosphoramidities that are useful for asymmetric catalysis. The aggregation-induced red phosphorescence and circularly polarized luminescence of the Pt(II) complexes of (*S*)-**276** were studied.^317^

**Scheme 94:**
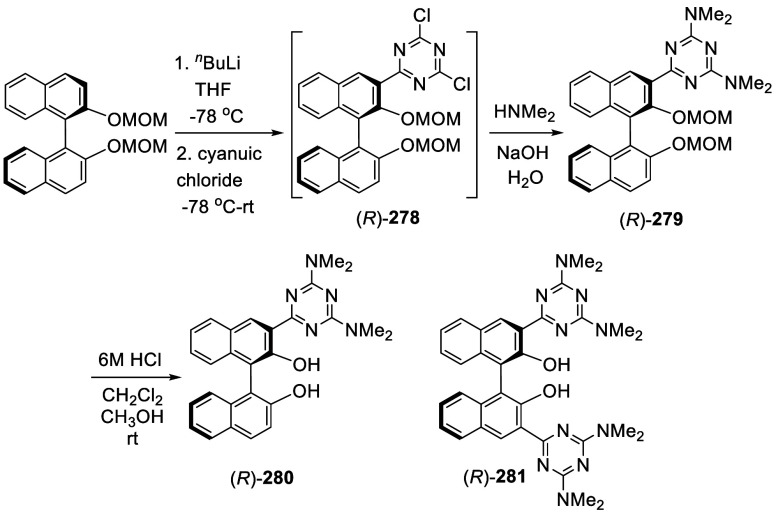
Nucleophilic Substitution of Cyanuic Chloride with Ortho-lithiated (*R*)-BINOL-MOM

Nucleophilic substitution of cyanuric chloride by the mono-*ortho*-lithiated (*R*)-BINOL-MOM formed from the reaction with 1.1 equiv of ^*n*^BuLi gave (*R*)-**278**, which upon reaction with HNMe_2_ and NaOH gave (*R*)-**279** in 52% yield over the two steps (Scheme 94).^252^ Removal of the MOM protecting groups of (*R*)-**279** gave (*R*)-**280**. In a similar way, the disubstituted compound (*R*)-**281** was obtained. These compounds were used to catalyze the reaction of ZnEt_2_ with aryl aldehydes.

### 7.5 Reactions of Other BINOL Alkoxymethyl Ethers

**Scheme 95:**
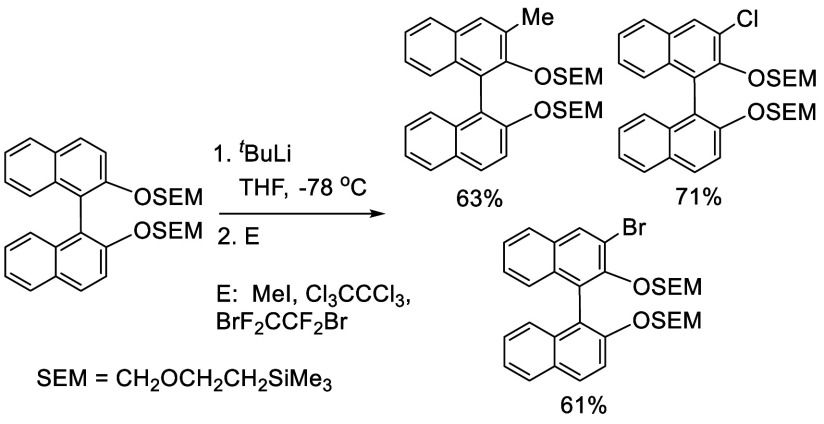
Preparation of 3-Monosubstituted BINOLs from BINOL-SEM

Ortho-lithiation of BINOL trimethylsilylethoxymethyl ether (BINOL-SEM) was studied.^228^ As shown in Scheme 95, after BINOL-SEM was treated with 2.7 equiv of ^*t*^BuLi in THF at −78 °C, it was reacted with several electrophiles to give the monomethylated, monochlorinated, and monobrominated products in 61–71% yield.

**Scheme 96:**
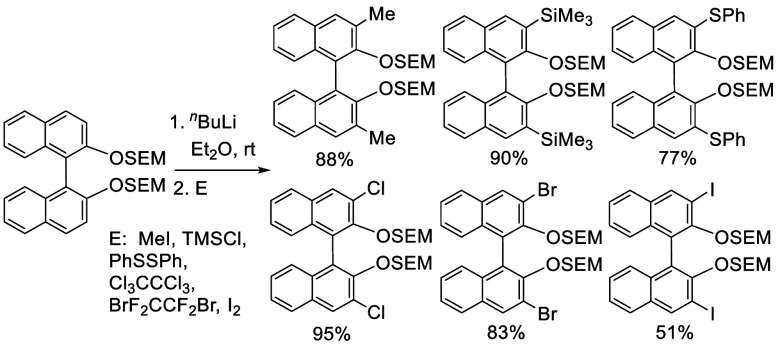
Preparation of 3,3′-Disubstituted BINOLs from BINOL-SEM

**Scheme 97:**
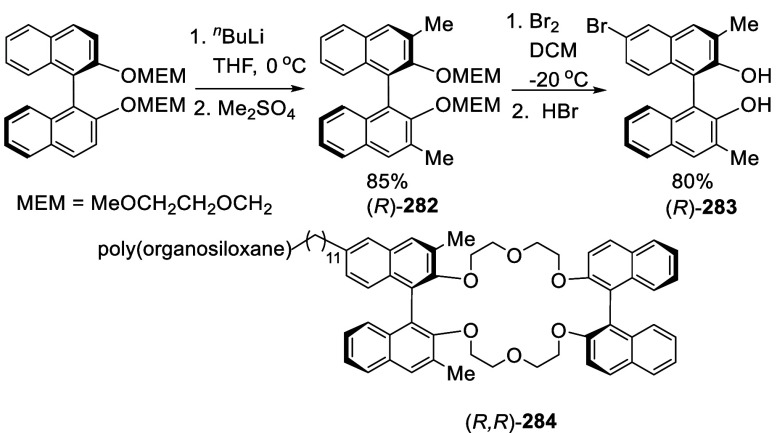
Ortho-lithiation Methylation of (*R*)-BINOL-MEM

When BINOL-SEM was treated with 2.5 equiv of ^*n*^BuLi in Et_2_O at room temperature for 3 h, its reaction with a number of electrophiles gave 3,3′-disubstitited products in 51–90% yields (Scheme 96). When the optically active (*S*)-BINOL-SEM was used for the methylation, (*S*)-3,3′-dimethylBINOL-SEM was obtained with the retention of the chiral configuration.

(*R*)-BINOL 2-methoxyethoxymethyl ether (BINOL-MEM) was used to prepare (*R*)-3,3′-dimethyl BINOL.^318^ As shown in Scheme 97, after (*R*)-BINOL-MEM in THF was treated with 3 equiv of ^*n*^BuLi at 0 °C for 45 min, it was then reacted with 3 equiv of dimethyl sulfate at room temperature for 16 h to give the 3,3′-dimethyl product (*R*)-**282** in 85% yield. (*R*)-**282** was converted to the monobromo compound (*R*)-**283** by reaction with Br_2_ followed by HBr promoted deprotection of the MEM groups. This compound was used to prepare a poly(organosiloxane)-supported bisBINOL crown ether (*R*,*R*)-**284** for chiral resolution of amino esters.

### 7.6 Reactions of BINOL Carbamates

**Scheme 98:**
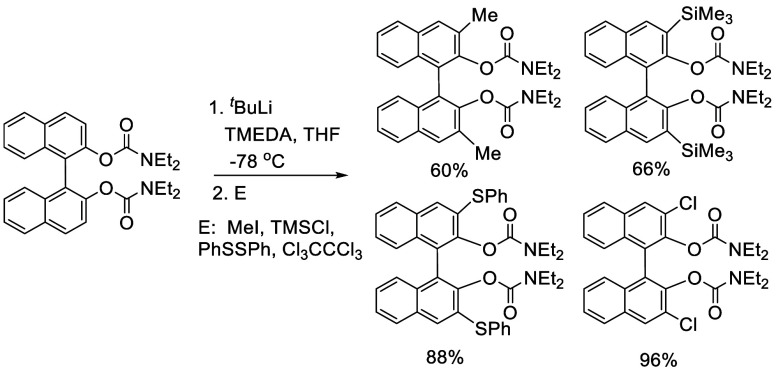
Preparation of 3,3′-Disubstituted BINOLs from a BINOL Dicarbamate

The carbamate groups of BINOL were used to direct the ortho-lithiation at the 3,3′-positions. As shown in Scheme 98, after the BINOL dicarbamate was treated with 2.5 equiv of ^*t*^BuLi in the presence of TMEDA in THF at −78 °C in 1 h, several electrophiles were added to give the 3,3′-disubstituted products in 60–96% yields.^228^

**Scheme 99:**
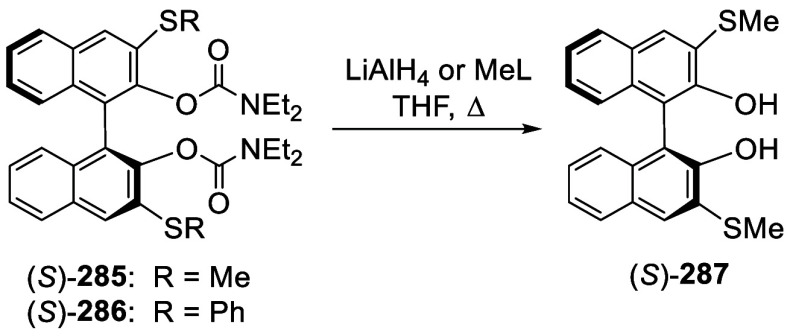
Deprotection of BINOL Carbamates

The optically active (*S*)-**285** and (*S*)-**286** were prepared according to Scheme 98 from the corresponding (*S*)-BINOL carbamates and were then subjected to deprotection with LiAlH_4_ or MeLi (Scheme 99).^276^ It was found that (*S*)-**285** was converted to (*S*)-**287** cleanly, but the reaction of (*S*)-**286** was sluggish and failed to give clean deprotection. Thus, it is easier to remove the MOM protecting groups of BINOLs than the carbamate groups.

**Scheme 100:**
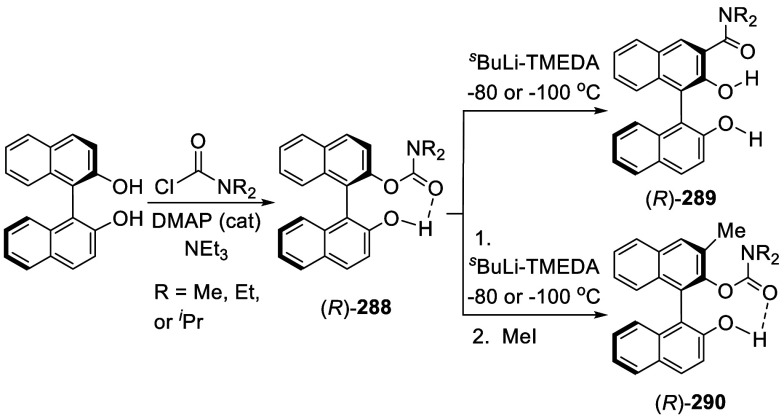
Fries Rearrangement and Methylation of BINOL Monocarbamate

BINOL carbamates were found to undergo the Fries rearrangements. When (*R*)-BINOL was reacted with even excess amount of dialkylcarbamoyl chlorides in the presence of DMAP-NEt_3_, only the monocarbamte products (*R*)-**288** were obtained in 61–87% yields (Scheme 100).^319^ This is attributed to the intramolecular hydrogen bond of (*R*)-**288**, which makes the second hydroxyl group less reactive. When compounds (*R*)-**288** were treated with 2.2 equiv of ^*s*^BuLi-TMEDA at −80 or −100 °C, a Fries rearrangement took place to give the 3-amido products (*R*)-**289** in 33–62% yields. When 1 equiv of MeI was added to the above reaction mixture before warming up, the 3-methylated product (*R*)-**290** was obtained in 35–77% yields.

**Scheme 101:**
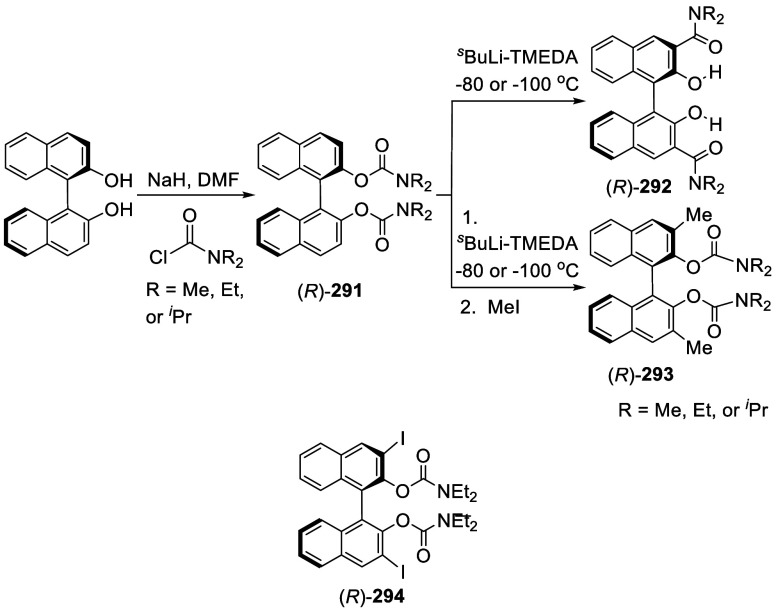
Fries Rearrangement and Methylation of BINOL Dicarbamates

The BINOL dicarbamate (*R*)-**291** was obtained by reaction of (*R*)-BINOL with NaH then dialkylcarbamoyl chloride (Scheme 101).^320^ When compounds (*R*)-**291** were treated with 2.3 equiv of ^*s*^BuLi-TMEDA at −80 or −100 °C, a Fries rearrangement took place to give the 3,3′-diamido products (*R*)-**292** in 25–80% yields. When 2 equiv of MeI was added to the above reaction mixture before warming up, the 3,3′-dimethylated products (*R*)-**293** were obtained in 30–50% yields. When CF_3_CH_2_I was used as the iodonation reagent after ortho-lithiation of (*R*)-**291** (R = Et), the diiodide (*R*)-**294** was obtained in 79% yield.

The carbamate groups of compounds (*R*)-**290** (R = Et) and (*R*)-**293** (R = Et) were removed by reaction with an excess amount of LiAlH_4_ to give compounds (*R*)-**295** and (*R*)-**218** in 46% and 73% yields, respectively.
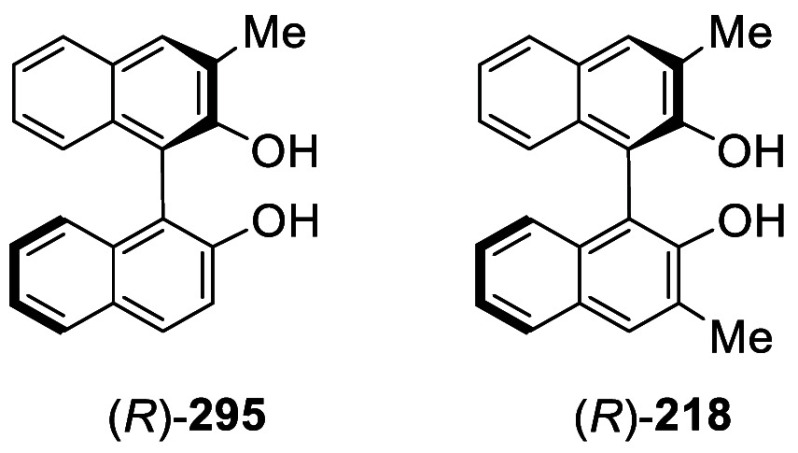


**Scheme 102:**
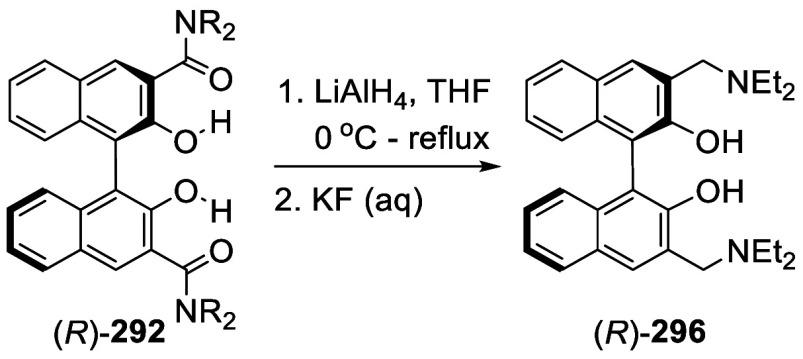
Conversion of 3,3′-DiamidoBINOL to 3,3′-Diaminomethyl BINOL

Reduction of the 3,3′-diamido compound (*R*)-**292** with LiAlH_4_ in THF at 0 °C – reflux followed by quenching the reaction with aqueous KF gave the 3,3′-diaminomethyl compound (*R*)-**296** in 63% yield (Scheme 102).^321^ The aluminum complex of this compound was used to catalyze the asymmetric reactions of a variety of cyanides with aldehydes.

**Scheme 103:**
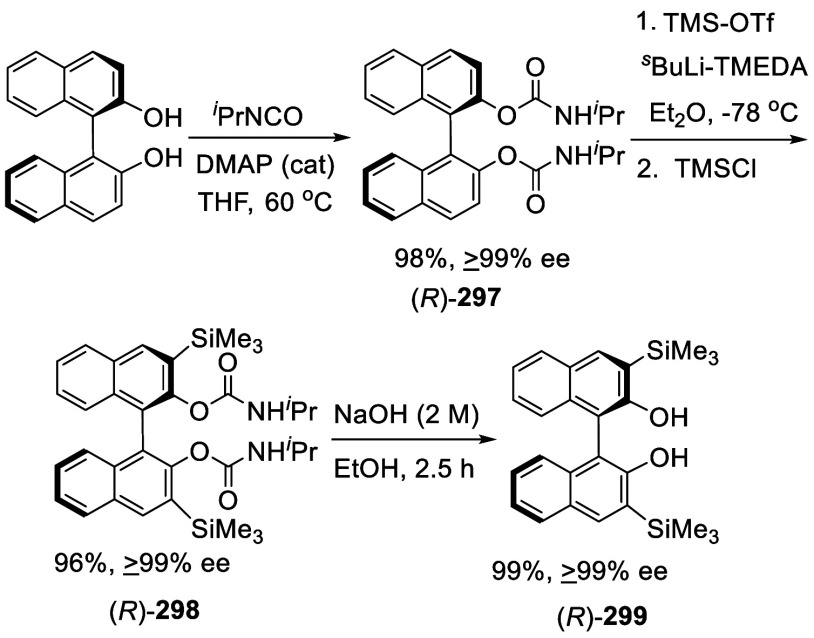
Ortho-lithiation Silylation of a BINOL-Carbamate

The BINOL carbamate (*R*)-**297** (≥99% ee) was prepared from the reaction of (*R*)-BINOL with ^*i*^PrNCO in THF in the presence of DMAP at 60 °C for 2 d (Scheme 103).^322^ Treatment of (*R*)-**297** with TMS-OTf gave a N-silylated intermediate which underwent ortho-lithiation in the presence of 5 equiv of ^*s*^BuLi-TMEDA at −78 °C. Addition of 6 equiv of TMSCl followed by workup gave (*R*)-**298** in 96% yield. The carbamate groups of (*R*)-**298** were quantitatively removed by reaction with NaOH (2 M) in ethanol to give (*R*)-**299** with high enantiomeric purity.

### 7.7 Reactions of BINOL Phosphorus Derivatives

#### 7.7.1 Phosphor–Fries Rearrangement

**Scheme 104:**
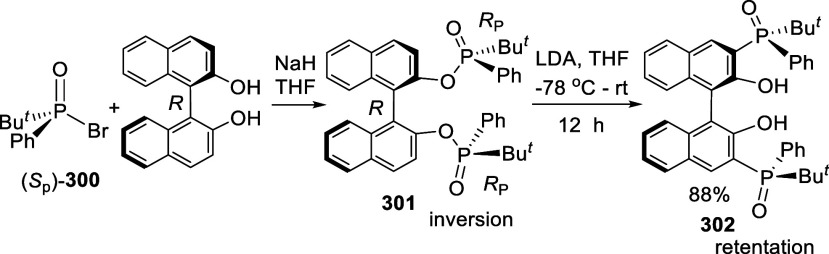
Phosphor–Fries Rearrangement of a BINOLPhosphinate

Reaction of P-chiral phosphinobromidate (*S*_P_)-**300** with NaH-deprotonated (*R*)-BINOL gave the BINOL phosphinate **301** with the inversion of the configuration at the phosphorus center (Scheme 104).^323^ Ortho-lithiation of **301** with LDA led to a phosphor–Fries rearrangement to form the 3,3′-bisphosphine oxide **302** with the retention of the configuration at the phosphorus center.^324^

**Scheme 105:**
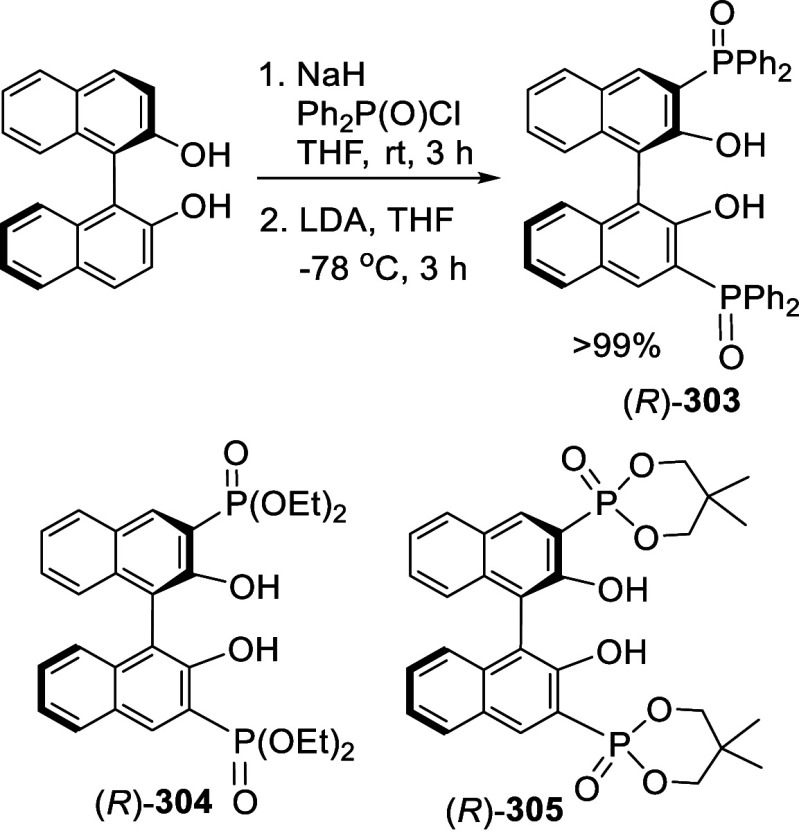
Phosphor–Fries Rearrangement of a BINOLPhosphine Oxide

The two-step reaction from (*R*)-BINOL to the 3,3′-bisphosphine oxide (*R*)-**303** was conducted in almost quantitative yield by a phosphor–Fries rearrangement (Scheme 105).^325^ The single crystal X-ray structure of (*R*)-**303** was obtained. Compounds (*R*)-**304** and (*R*)-**305** were obtained by using the method similar to the preparation of (*R*)-**303**.^326,327^

**Scheme 106:**
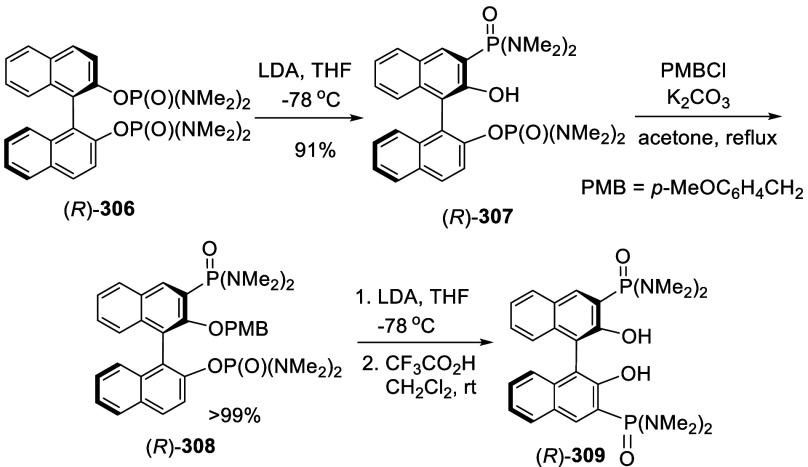
Phosphor–Fries Rearrangement of a BINOL-Phosphoramide

When the phosphoramide (*R*)-**306** was treated with 10 equiv of LDA, only the monophospho–Fries rearrangement took place to give (*R*)-**307** (Scheme 106).^326,327^ Protection of (*R*)-**307** with a PMB group gave (*R*)-**308**, which underwent the phosphor–Fries rearrangement in the presence of LDA, followed by acidic deprotection to give the 3,3′-diphosphoamidate BINOL (*R*)-**309** in 92% and 68% yields respectively for the two steps. These 3,3′-disubstituted BINOLs were used to catalyze the asymmetric reaction of dialkylzincs and diphenylzinc with aldehydes.

#### 7.7.2 Iodonation

**Scheme 107:**
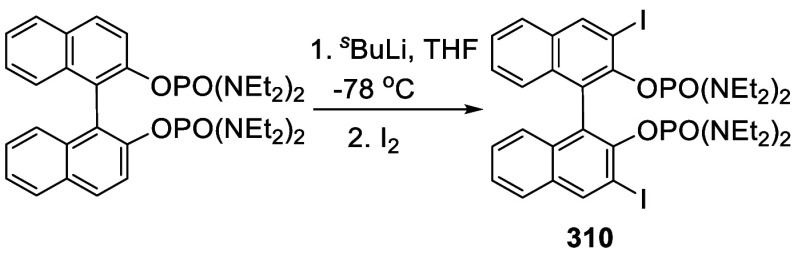
Ortho-lithiation Iodonation of a BINOL-Phosphoramide

The phosphorodiamidate groups of BINOL can direct the ortho-lithiation-iodonation at 3,3′-positions (Scheme 107).^328^ After the BINOL-phosphorodiamidate was treated with 2.5 equiv of ^*s*^BuLi in THF at −78 °C for 90 min, it was quenched with the addition of I_2_ to give the diiodide **310** in 39% yield.

**Scheme 108:**
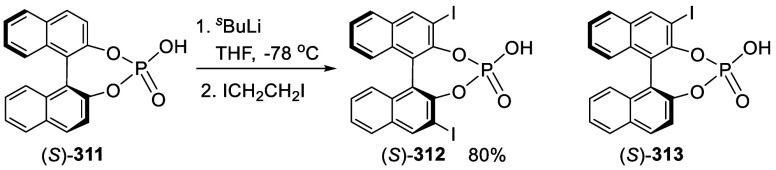
Ortho-lithiation Iodonation of a BINOL-Phosphoric Acid

**Scheme 109:**
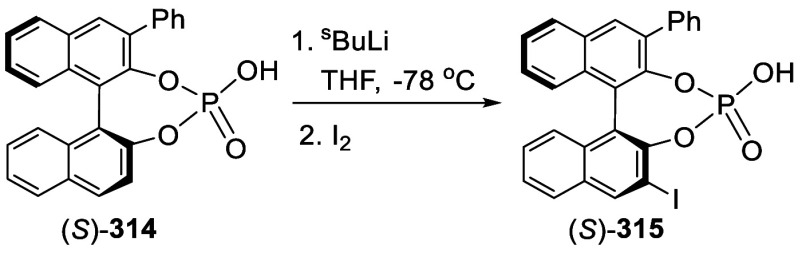
Mono-*ortho*-lithiation Iodonation of a BINOL-Phosphoric Acid

When (*S*)-BINOL-phosphoric acid (*S*)-**311** was treated with 3.1 equiv of ^*s*^BuLi in THF at −78 °C for 1.5 h followed by addition of 1,2-diiodoethane, the 3,3′-diiodo product (*S*)-**312** was obtained in 80% yield (Scheme 108).^329^ The monoiodo product (*S*)-**313** was obtained in 68% yield from the reaction of (*S*)-**311** with 3.4 equiv of ^*n*^BuLi and TMEDA in THF at −78 °C to −40 °C for 2 h, followed by the addition of I_2_. Monoiodonation of the monophenyl compound (*S*)-**314** gave the unsymmetrically disubstituted compound (*S*)-**315** in 41% yield (Scheme 109).

**Scheme 110:**
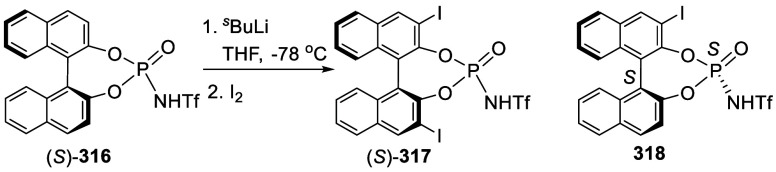
Ortho-lithiation Iodonation of a BINOL-Phosphoramide

Ortho-lithiation of (*S*)-BINOL-*N*-triflylphosphoramide (*S*)-**316** with 6 equiv of ^*s*^BuLi in THF at −78 °C for 1–3 h followed by addition of I_2_ gave the diiodo product (*S*)-**317** in 42% yield (Scheme 110).^329^ When (*S*)-**316** was treated with 2.5–3.0 equiv of ^*n*^BuLi in THF at −78 to 30 °C for 1–3 h followed by addition of I_2_, the monoiodide **318** was obtained in 40% yield with a diastereomeric ratio >9:1. In **318**, the phosphorus center is chiral due to the unsymmetric 3,3′-positions of the BINOL unit.

#### 7.7.3 Zincation

**Scheme 111:**
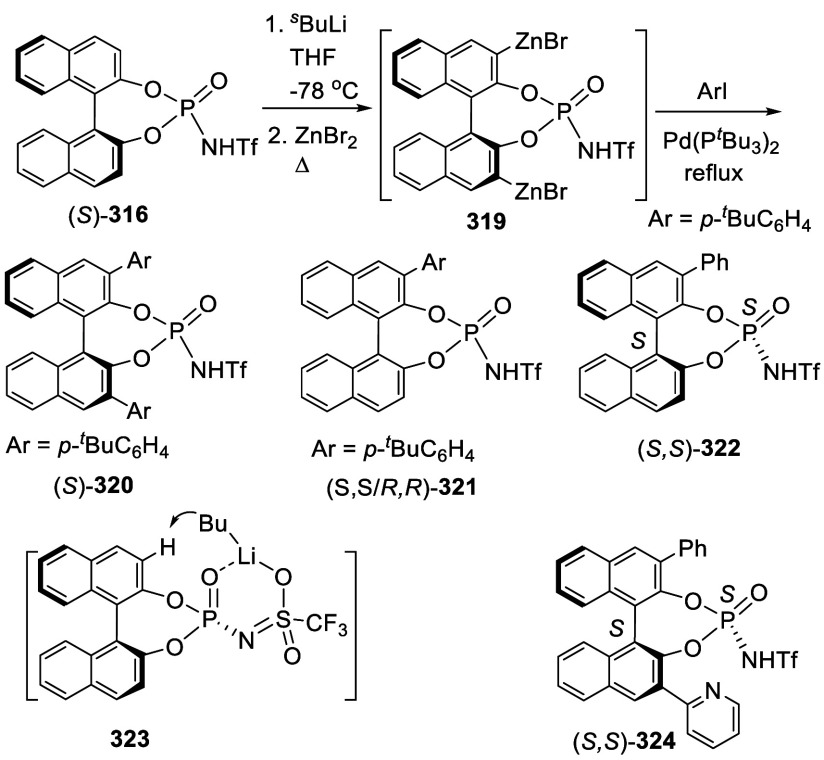
Ortho-lithiation Zincation of a BINOL Phosphoramide

Ortho-lithiation of (*S*)-**316** with 6 equiv of ^*s*^BuLi followed by addition of ZnBr_2_ gave the dizinc complex **319** (Scheme 111).^329^ Without isolation, **319** was reacted with *p*-^*t*^BuC_6_H_4_I to give the Negishi coupling product (*S*)-**320** in 27% yield. When racemic **316** was treated with 2.8 equiv of ^*n*^BuLi at −78 °C to −50 °C followed by reaction with ZnBr_2_ at 80 °C, the resulting monozinc complex was coupled with *p*-^*t*^BuC_6_H_4_I to give (*S*,*S*/*R*,*R*)-**321** in 56% yield. A single crystal X-ray analysis established its structure. In a way similar to the synthesis of (*S*,*S*/*R*,*R*)-**321**, when (*S*)-**316** and PhI were used, (*S*,*S*)-**322** was obtained in 50% yield as one diastereomer. On the basis of the X-ray analysis, an intermediate like **323** was proposed to account for the diastereoselectivity of the first ortho-lithiation step. When (*S*,*S*)-**322** was treated with ^*s*^BuLi and ZnBr_2_ followed by the Negishi coupling with 2-bromopyridine, the unsymmetrically disubstituted compound (*S*,*S*)-**324** was obtained in 47% yield.

#### 7.7.4 Silylation

**Scheme 112:**
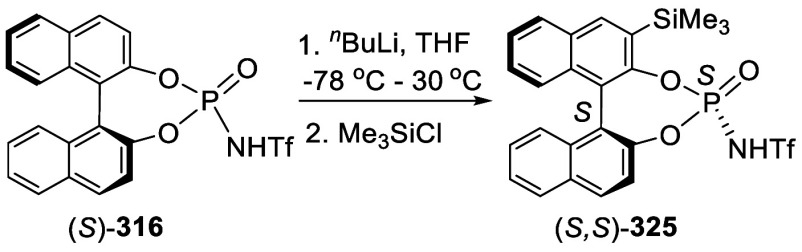
Mono-*ortho*-lithiation Silylation of a BINOL Phosphoramide

Ortho-lithiation of (*S*)-**316** with 2.5–3.0 equiv of ^*n*^BuLi in THF at −78 to 30 °C for 1–3 h followed by addition of Me_3_SiCl gave the monosilyl product (*S*,*S*)-**325** in 64% yield as one diastereomer (Scheme 112).^329^ An intermediate like **323** can be used to explain the observed diastereoselectivity.

#### 7.7.5 Phosphorylation

**Scheme 113:**
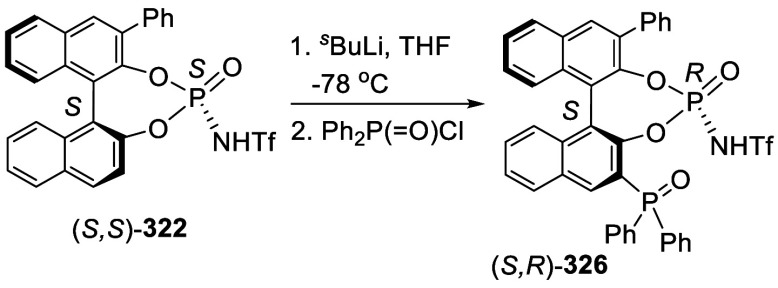
Ortho-lithiation Phosphorylation of a BINOL Phosphoramide

Ortho-lithiation of (*S*,*S*)-**322** with 3 equiv of ^*s*^BuLi in THF at −78 °C followed by the addition of Ph_2_P(=O)Cl gave the unsymmetrically disubstituted product (*S*,*R*)-**326** in 56% yield (Scheme 113).^329^

#### 7.7.6 Addition to Ketones

**Scheme 114:**
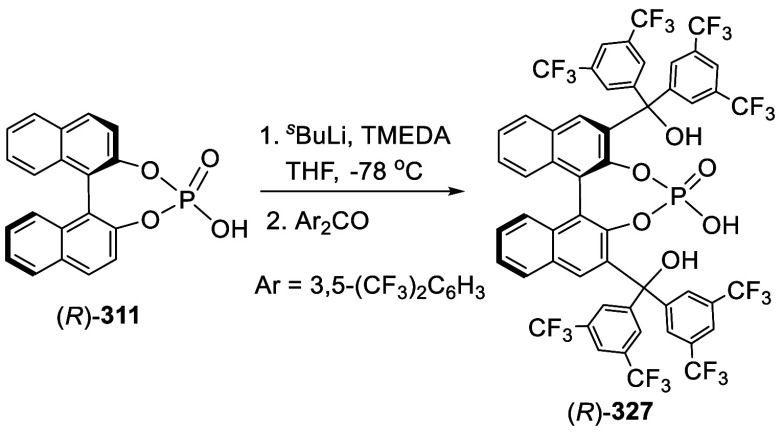
Addition of an Ortho-lithiated BINOL Phosphoric Acid to a Ketone

When (*R*)-BINOL-phosphoric acid, (*R*)-**311**, was treated with 3.5 equiv of ^*s*^BuLi and TMEDA followed by addition of a diaryl ketone, the 3,3′-disubstituted product (*R*)-**327** was obtained in 42% yield (Scheme 114).^330^

### 7.8 Reactions of BINOL Sulfonates

**Scheme 115:**
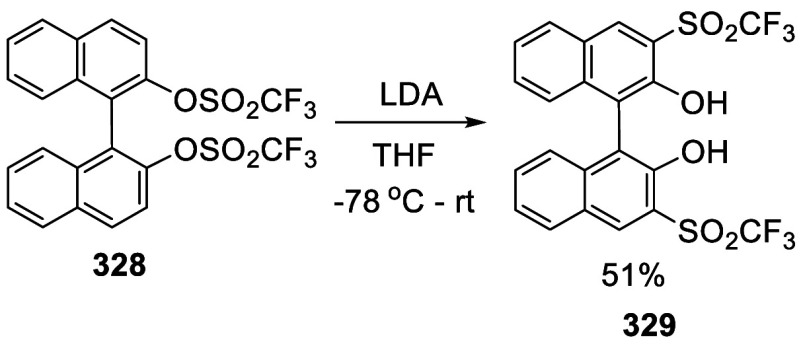
Thia-Fries Rearrangement of BINOL Triflate

Ortho-lithiation of BINOL triflate **328** led to a thia-Fries rearrangement to 3,3′-substututed BINOL **329** (Scheme 115).^331^ Sequential treatment of BINOL triflate with 2 × 1 equiv of LDA at −78 °C to room temperature in THF led to the formation of **329** in 51% yield. The single crystal X-structure of **329** was obtained. Much lower yields (7%) were observed when the CF_3_ group of the BINOL triflate were replaced with other fluorinated alkyl groups such as C_4_F_9_ and C_8_F_17_.^332^ The optically active (*R*)-**329** was used to catalyze the asymmetric reactions of allylic halides with imines in the presence of In(0).

## 8 Transition Metal Catalyzed C–H Activation

Transition metal-catalyzed C–H activation has been used to functionalize BINOLs. As described in this section, with the assistance of a functional group at an adjacent position, highly regioselective C–H activations of BINOL in the presence of transition metal catalysts have been achieved at various positions.

### 8.1 Pd-Catalyzed Arylation and Vinylation at 3-Position

**Scheme 116:**
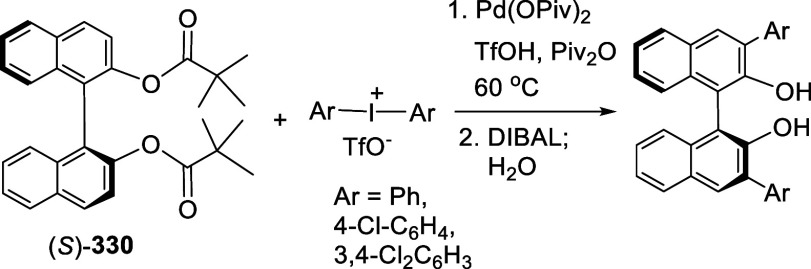
Pd-Catalyzed Arylation of a BINOL Ester

In 2010, Xiao et al. reported a Pd(II)-catalyzed C–H activation of the BINOL dipivalate (*S*)-**330** to generate the 3,3′-diarylBINOLs (Scheme 116).^333^ The reaction was carried out in dichloroethane in the presence of 20 mol % Pd(OPiv)_2_ (Piv = ^*t*^BuCO), 20 mol % HOTf, 3 equiv of Ar_2_I^+^TfO^–^, and 1 equiv of Piv_2_O at 60 °C for 24 h. After the reaction, deprotection with DIBAL gave the 3,3′-diarylBINOLs in 35–43% yields.

**Scheme 117:**
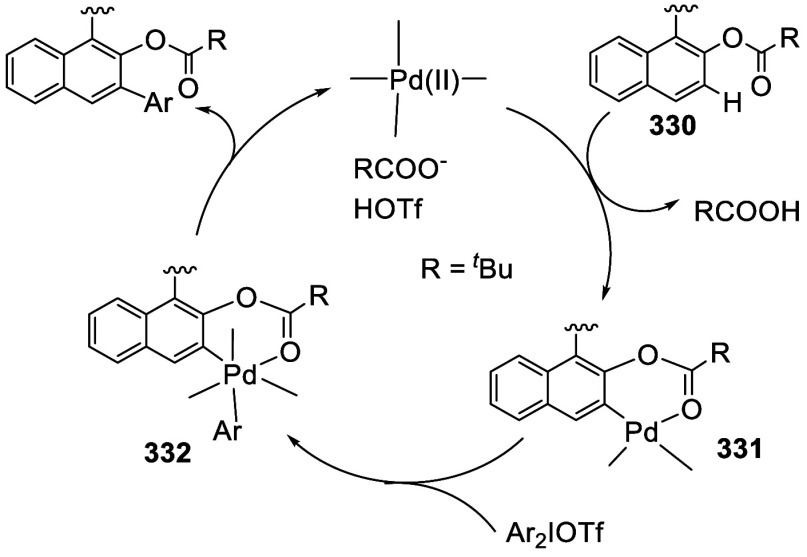
Proposed Mechanism for the Pd(II)-Catalyzed Arylation

Scheme 117 shows a proposed mechanism for this reaction. In the first step, coordination of the ester group of the substrate **330** with the Pd(II) center directs an adjacent C–H activation to form a Pd(II) intermediate **331**. Oxidative addition of an aryl group to **331** by Ar_2_IOTf can generate a Pd(IV) intermediate **332**. Reductive elimination of **332** should give the desired arylation product and regenerate the Pd(II) catalyst.

**Scheme 118:**
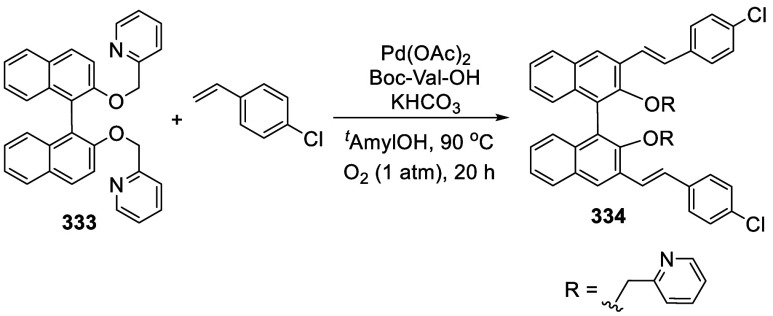
Pd(II)-Catalyzed Alkenylation of a BINOL 2-Pyridylmethyl Ether

In 2012, Cong et al. reported a Pd(II)-catalyzed 3,3′-divinylation of BINOL directed by the 2-pyridylmethyl ether groups at the 2,2′-positions of **333** (Scheme 118).^334^ It was found that in the presence of 10 mol % Pd(OAc)_2_, 20 mol % amino acid ligand Boc-Val-OH, 4 equiv KHCO_3_ in ^*t*^AmylOH under 1 atm O_2_ at 90 °C over 20 h, and racemic **333** reacted with 5 equiv of *p*-chlorostyrene to give the 3,3′-divinyl product **334** in 55% yield.

**Scheme 119:**
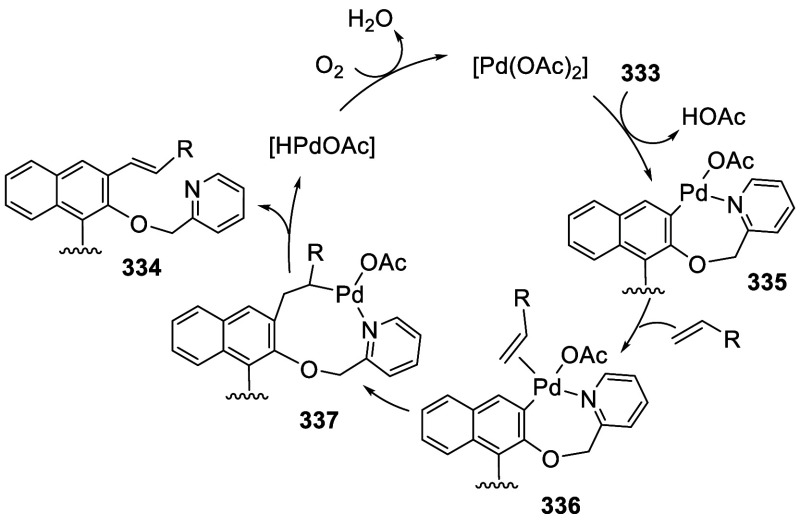
Proposed Mechanism for the Pd(II)-Catalyzed Vinylation

A proposed mechanism of this reaction is shown in Scheme 119. Coordination of the pyridyl methyl group of **333** with Pd(OAc)_2_ can direct the C–H activation at the 3-position to form the 7-membered metallacycle intermediate **335**. An alkene substrate can then coordinate to the Pd(II) center to form the intermediate **336**, which can undergo a regioselective migratory insertion to form **337**. β-Elimination of **337** will give the vinylation product **334**, and the resulting Pd intermediate can be oxidized by O_2_ to the catalytically active Pd(II) complex.

### 8.2 Rh-Catalyzed Reactions at 3-, 4-, and 5-Positions

**Scheme 120:**
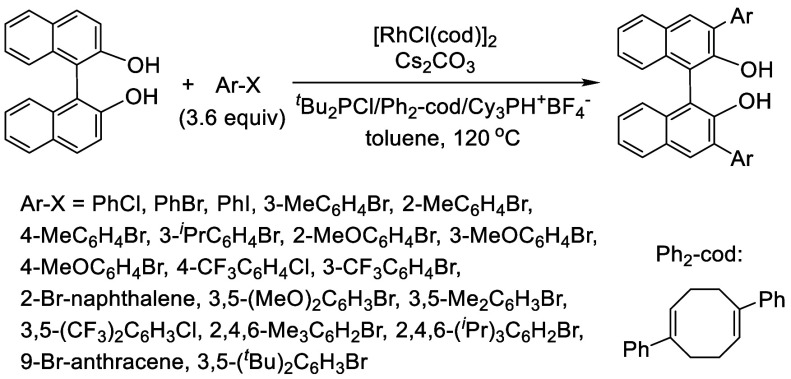
Rh(I)-Catalyzed Reaction of BINOL with Aryl Halides

In 2016, Yang et al. reported a rhodium-catalyzed C–H activation of BINOL to make racemic 3,3′-diarylBINOL in one step.^335^ As shown in Scheme 120, the reaction of BINOL with an aryl halide (3.6 equiv) catalyzed by [RhCl(cod)]_2_ (2.5 mol %) complex in the presence of Cs_2_CO_3_ (2.0 equiv), and three ligands including ^*t*^Bu_2_PCl (50 mol %), Ph_2_-cod (5 mol %), and Cy_3_PH^+^BF_4_^–^ (2.5 mol %) was conducted in toluene at 120 °C to give a 3,3′-diarylBINOL in 10–96% yields. This method allows a variety of aryl groups to be readily introduced to BINOL.

**Scheme 121:**
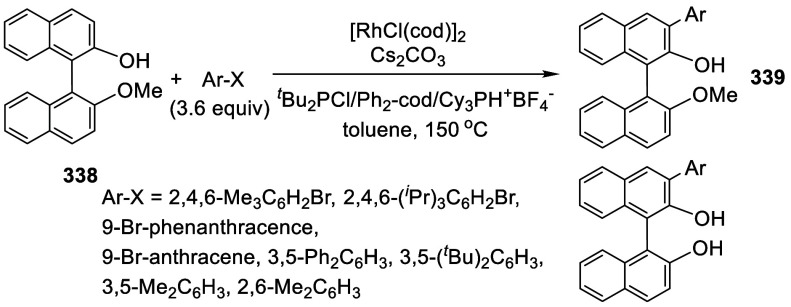
Rh(I)-Catalyzed Reaction of the Monomethylated BINOL with Aryl Halides

When one of the hydroxyl group of BINOL is methylated, the reaction can be controlled at monoarylation as shown in Scheme 121. Reaction of **338** with an aryl halide (3.6 equiv)) catalyzed by [RhCl(cod)]_2_ (2.5 mol %) complex in the presence of Cs_2_CO_3_ (2.0 equiv) and three ligands, including ^*t*^Bu_2_PCl (20 mol %), Ph_2_-cod (5 mol %), and Cy_3_PH^+^BF_4_^–^ (2.5 mol %), was conducted in toluene at 150 °C to give **339** in up to 96% yields. The reaction of the monomethylated BINOL gave higher yields than BINOL for the reaction with the sterically bulky aryl bromides. The methyl group of **339** can be removed by using BBr_3_, and the resulting 3-arylBINOLs are also very useful in asymmetric catalysis.^336−338^

It was found that the above reaction conditions to prepare the 3-aryl and 3,3′-diaryl BINOLs led to racemization when the optically active BINOL was used. The racemization took place even at 70 °C in the presence of Cs_2_CO_3_. The racemic 3,3′-diphenylBINOL can be resolved into the optically active (*R*)- and (*S*)-compounds with 95% to >99% ee’s by using *N*-benzylcinchonidinium chloride.

**Scheme 122:**
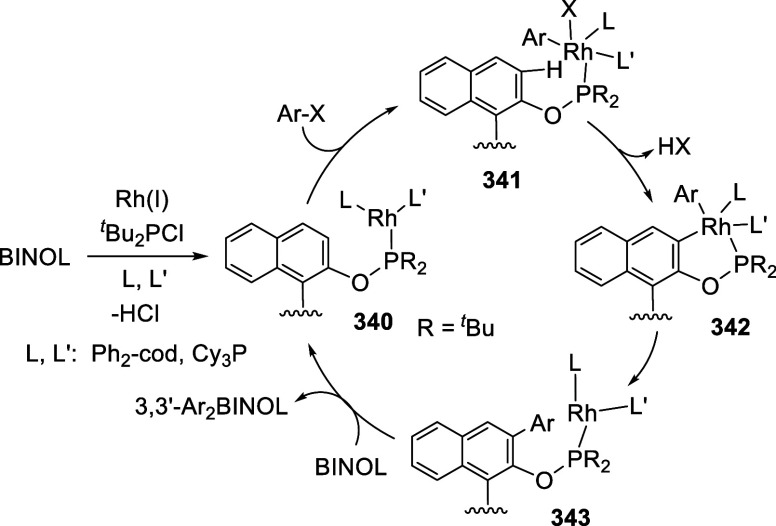
Proposed Mechanism for the Rh(I)-Catalyzed Arylation of BINOL

Scheme 122 shows a proposed mechanism for the Rh(I)-catalyzed arylation of BINOL. Under basic conditions, BINOL reacts with ^*t*^BuPCl_2_ and the Rh(I) complex in the presence of other ligands (L and L′) to give complex **340**. The bulky ligands allow the Rh(I) to coordinate to only one phosphorus atom rather than the formation of the chelated diphosphine coordination. This is important for the catalytic activity of the intermediate **340**. Oxidative addition of an aryl halide with **340** will form the intermediate **341**. The subsequent C–H activation and elimination of HX converts **341** to **342**. Reductive elimination of **342** gives **343**. Reaction of **343** with BINOL releases the arylated product and regenerates the catalytically active intermediate **340**.

**Scheme 123:**
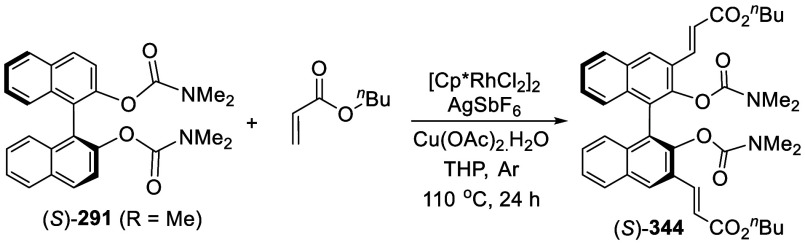
Rh(III)-Catalyzed Alkenylation of a BINOL Carbamate

In 2011, Liu and co-worker conducted a Rh(III)-catalyzed 3,3′-divinylation of BINOL directed by 2,2′-carbamate groups (Scheme 123).^339^ In the presence of 1 mol % [Cp*RhCl_2_]_2_, 4 mol % AgSbF_6_, 2 equiv of Cu(OAc)_2_, (*S*)-**291** (R = Me) reacted with 5 equiv of *n*-butyl acrylate in THP at 110 °C for 24 h to give (*S*)-**344** in 82% yield with 96% ee. The enantiomeric purity of BINOL was maintained in this reaction.

**Scheme 124:**
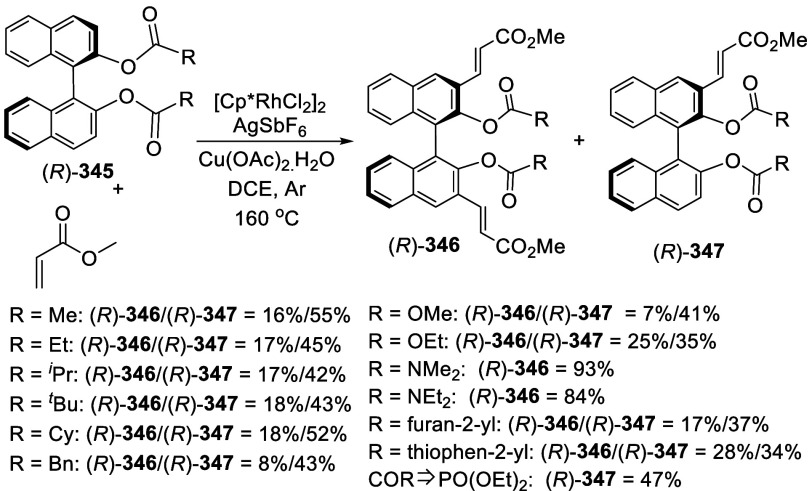
Rh(III)-Catalyzed Alkenylation of BINOL Esters, Carbonates, and Carbamates

In 2020, Dong and co-workers reported the use of the BINOL esters and other derivatives (*R*)-**345** for the similar reactions (Scheme 124).^340^ It was found that in the presence of 7 mol % [Cp*RhCl_2_]_2_, 0.3 equiv of AgSbF_6_, and 3.0 equiv of Cu(OAc)_2_·H_2_O, (*R*)-**345** reacted with 6.0 equiv of methyl acrylate in DCE at 160 °C over 20–24 h to give a mixture of (*R*)-**346** and (*R*)-**347**. It is remarkable that the BINOL compounds maintained their optical purity under this high reaction temperature. Among the BINOL starting materials, the reactions of the BINOL carbamates (R = NMe_2_ and NEt_2_) gave the corresponding divinyl products (*R*)-**346** in 93% and 84% yields, respectively, similar to that shown in Scheme 123.

The reactions of other alkyl acrylates with (*R*)-**345** (R = Me) gave results similar to those of methyl acrylate, and the reaction of vinyl phenyl sulfone gave predominately the monovinyl product (52%). Two fluorinated alkyl acrylates gave a mixture of divinyl and monovinyl products in higher yields. The substrates such as phenyl acrylate, acrylonitrile, acryl aldehyde, acrylic acid, methyl methacrylate, styrene, an enone, an acrylamide, and a vinyl ester gave little or no product for this reaction. When the 6,6′-positions of (*R*)-**345** (R = Me) were substituted with groups such as Me, ^*t*^Bu, and OMe, the resulting compounds were reacted with methyl acrylate to give product mixtures similar to the reactions of (*R*)-**345** (R = Me). The reaction of 6,6′-diphenyl-substituted (*R*)-**345** (R = Me) with methyl acrylate gave only the monovinyl product in 41% yield, and those of the 6,6′-dihalogenated (*R*)-**345** (R = Me) gave almost no product at all.

**Scheme 125:**
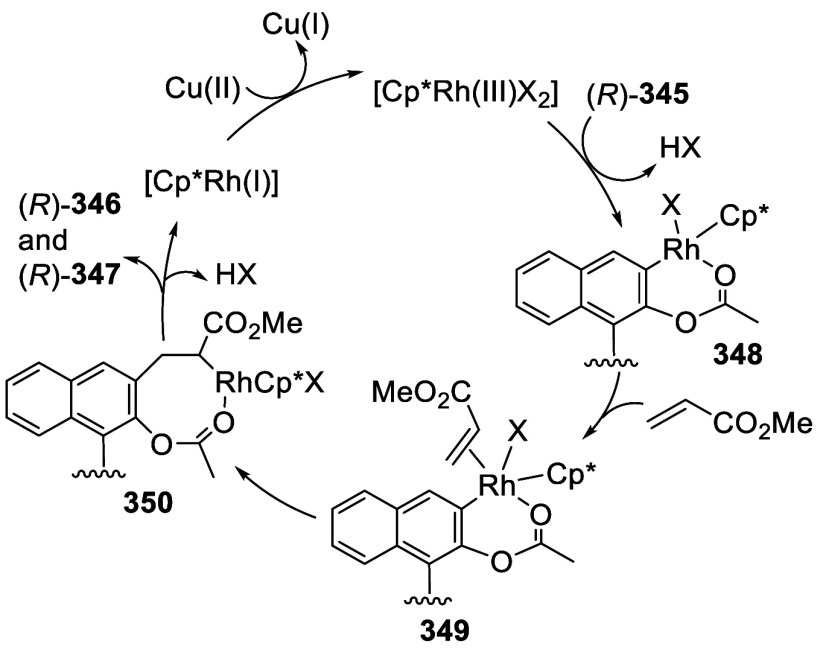
Proposed Mechanism for the Rh(III)-Catalyzed C–H Activation of BINOL Acetate

Scheme 125 shows a proposed mechanism for the Rh-catalyzed vinylation. When (*R*)-**345** is treated with the Rh(III) complex, the ester group of the BINOL can direct an *ortho*-C–H activation at the 3-position to generate the intermediate **348**. Coordination of **348** with methyl acrylate can generate the intermediate **349**, which can undergo a regioselective migratory insertion to form **350**. β-Elimination of **350** followed by reductive elimination will give the products (*R*)-**346** and (*R*)-**347** and form a Rh(I) intermediate. Oxidation of the Rh(I) intermediate with the Cu(II) complex can regenerate the catalytically active Rh(III) complex.

**Scheme 126:**
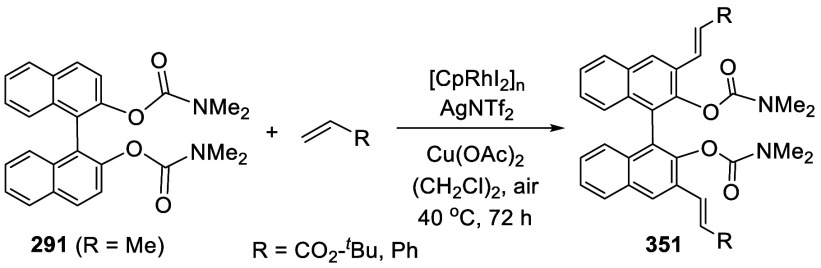
Rh(III)-Catalyzed Alkenylation of a BINOL Carbamate

In 2020, Tanaka et al. reported that by using [CpRhI_2_]*_n_* as the catalyst, the reaction of a BINOL carbamate with alkenes can be conducted under much milder conditions in high yields.^341^ It was found that in the presence of 10 mol % (Rh content) [CpRhI_2_]_*n*_, 20 mol % AgNTf_2_ and 40 mol % Cu(OAc)_2_, **291** (R = Me) (0.2 mmol) reacted with 3.0 equiv of methyl acrylate or styrene in DCE at 40 °C over 72 h in air to give the corresponding 3,3′-divinyl product **351** in 95% or 99% yield (Scheme 126). When 1 mmol of **291** was used to react with styrene, the 3,3′-divinyl product was obtained in 79% yield. In this case, the conversion of **291** was incomplete, but no monovinylation product was obtained. It suggests that the monovinylation product might undergo a faster vinylation to give the divinyl product. This reaction used a catalytic amount of both the Rh complex and Cu(OAc)_2_ in air. Thus, after oxidation of a Rh(I) intermediate to a catalytically active Rh(III) species by the Cu(II) complex similar to that shown in Scheme 125, the resulting Cu(I) can be oxidized back to Cu(II) by air.

**Scheme 127:**
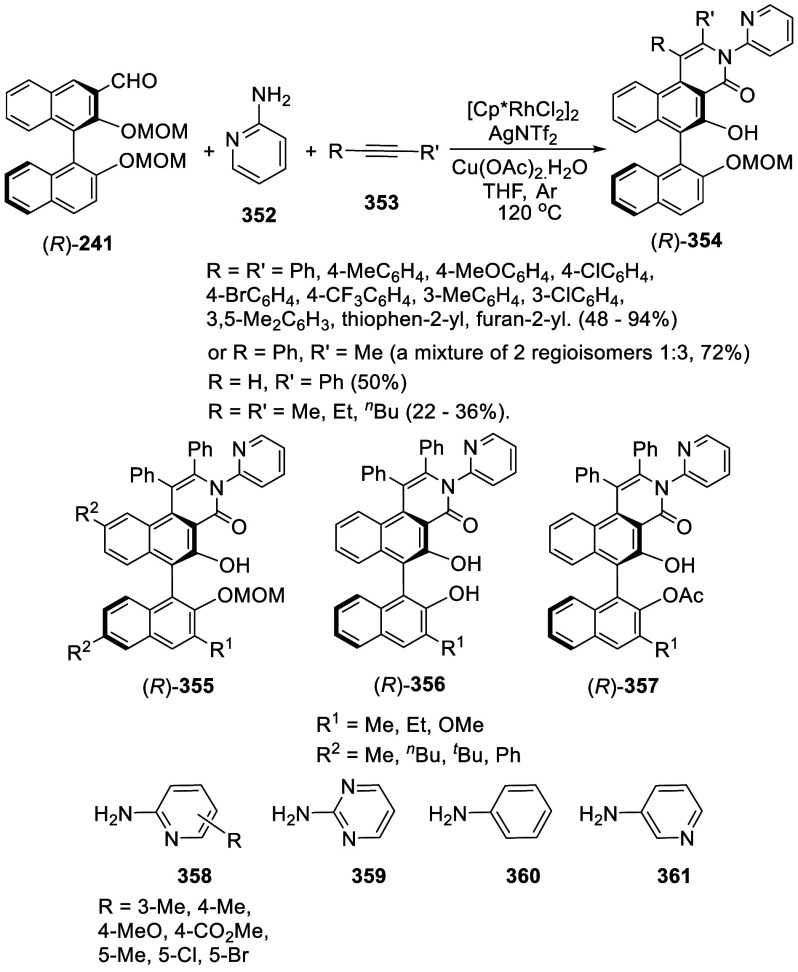
Rh-Catalyzed Reaction at 4-Position of BINOL

In 2021, Dong and co-workers reported the reaction of (*R*)-3-formylBINOL-MOM, (*R*)-**241**, prepared by the ortho-lithiation of BINOL-MOM followed by reaction with DMF, with pyridine-2-amine (**352**, 1.5 equiv) and an alkyne (**353**, 2.5 equiv) catalyzed by [Cp*RhCl_2_]_2_ (Scheme 127).^342^ This reaction was carried out in THF at 120 °C by using [Cp*RhCl_2_]_2_ (5 mol %) in the presence of AgNTf (0.2 equiv) and Cu(OAc)_2_·H_2_O (2.0 equiv). It directed a C–H activation at the 4-position of (*R*)-**241** to give the products (*R*)-**354** in up to 94% yields with the retention of the optical purity. The highest yield was observed for the reaction of the alkyne with R = R′ = 4-MeOC_6_H_4_ and much lower yields for the reaction of aliphatic alkynes. Other BINOL substrates with various 3-substituents (R^1^) and 6-substituents (R^2^) were also reacted with diphenylacetylene and **352** to give (*R*)-**355** in 64–87% yield. However, when R^2^ = Br (R^1^ = H), only a trace of the product was observed. Thus, a bromine substituent interfered the reaction. When the BINOL substrate did not have the MOM protecting groups, the product (*R*)-**356** was obtained in 43% yield. When the Ac protected substrate was used, the product (*R*)-**357** was obtained in 37% yield. When the substrate had other protecting groups such as Me, Bn, TBDPS, and OMSM, little product formation was observed. They also tested the reaction of (*R*)-**241** with diphenylacetylene and various derivatives of **352** such as **358**–**361**. When **358** with various R substituents were used, the corresponding products were obtained in 44–80% yields, but little product was obtained when R = 4-CN or 6-Me. Compound **359** gave the corresponding product in 65% yield, but compounds **360** and **361** gave little product. This indicates that the ring nitrogen and its proximity with the amine group are important for the reaction.

**Scheme 128:**
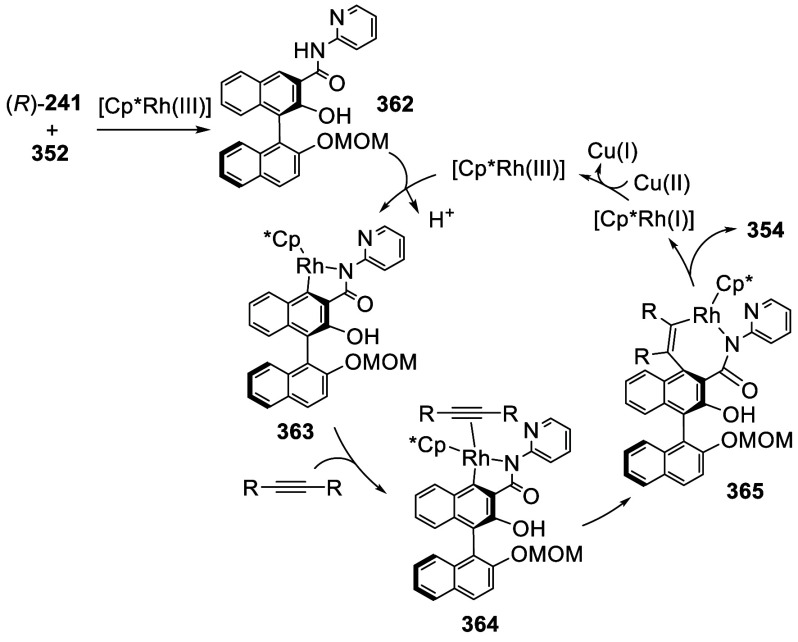
Proposed Mechanism for the Rh-Catalyzed C–H Activation

It was found that when (*R*)-**241** was treated with **352** under the reaction conditions, an oxidative amide formation and a partial hydrolysis took place to form **362** before C–H activation (Scheme 128). In the presence of a catalytically active Rh(III) complex, C–H activation of **362** at the 4-position can give the intermediate **363**, which upon coordination with an alkyne substrate will give **364**. This intermediate can undergo a migratory insertion to form **365**. Reductive elimination of **365** will give the functionalized BINOL product **354** and generate a Rh(I) intermediate. Oxidation of the Rh(I) intermediate by the Cu(II) complex will regenerate the catalytically active Rh(III) complex.

**Scheme 129:**
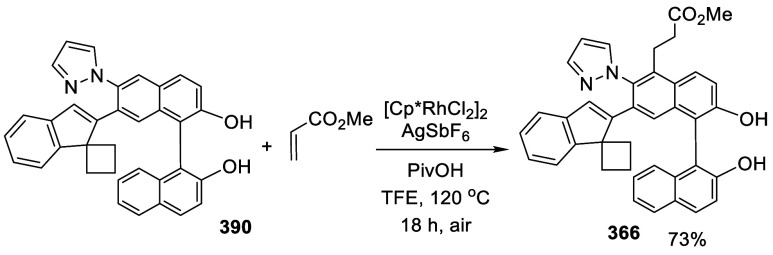
Rh-Catalyzed Reaction at 5-Position of BINOL

As shown in Scheme 134 (vide infra), the 6-pyrazole group of BINOL can direct the Co-catalyzed C–H activation at the less sterically hindered 7-position to form the product **390**.^343^ The pyrazole group of **390** was found to further direct a C–H activation at the 5-position upon Rh(III) catalysis (Scheme 129). In the presence of 5 mol % [Cp*RhCl_2_]_2_, 0.2 equiv of AgSbF_6_, and 4.0 equiv of PivOH, **390** reacted with 3.0 equiv of methyl acrylate in TFE at 120 °C under air for 18 h to give the 5-alkylated product **366** in 73% yield.

The proposed mechanism in Scheme 125 shows that the intermediate **350** undergoes β-elimination to give the vinylation product. However, the intermediate **367** generated from the reaction of **390** with methyl acrylate might undergo a protonation demetalation faster than the β-elimination to give the alkylation product **366**.
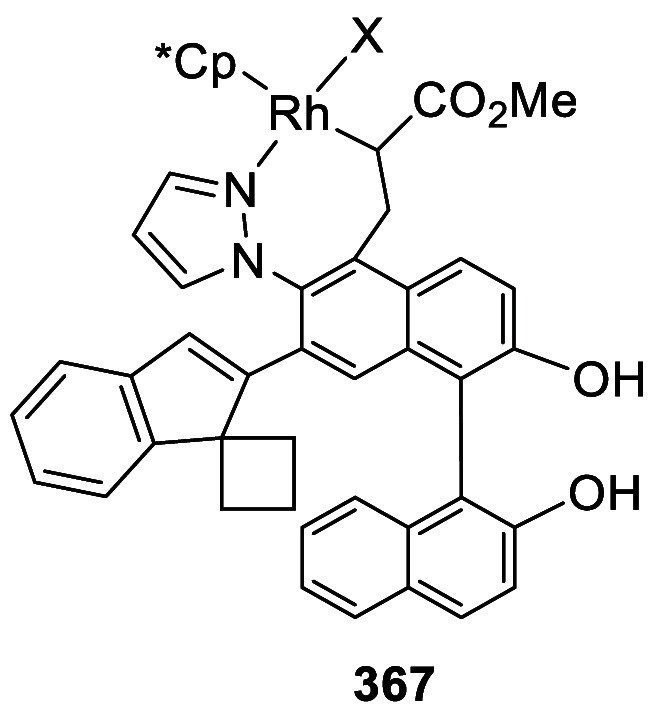


### 8.3 Co-Catalyzed Reactions at 3- and 7-Positions

**Scheme 130:**
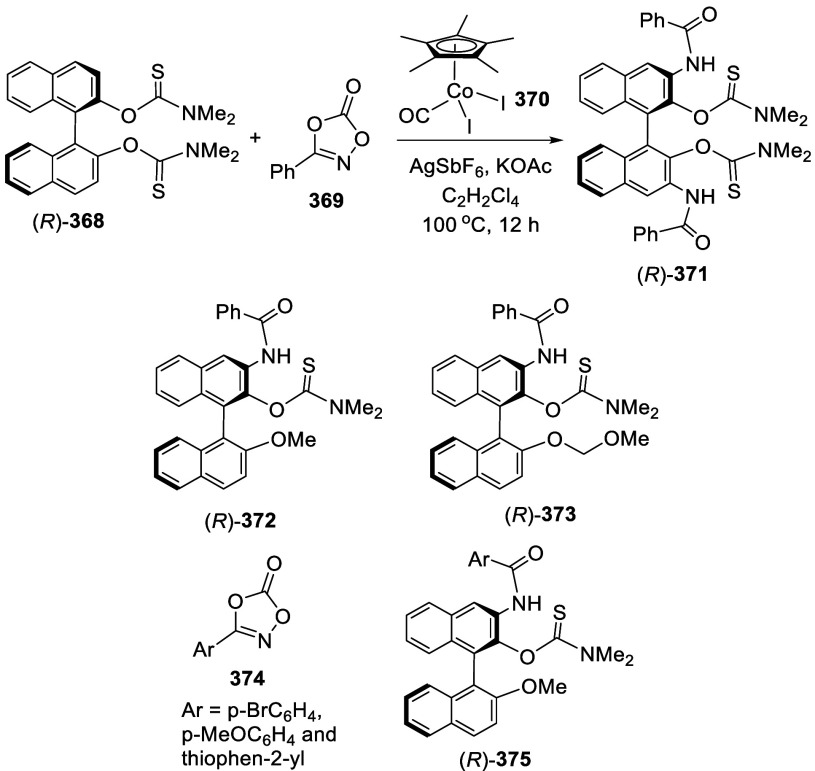
Co-Catalyzed Amidation at 3-Position of a BINOL Thiocarbamate

In 2020, Bera and Maji reported a Co(III)-catalyzed C–H activation of BINOL to introduce amide and alkyl groups to the 3-position.^344^ Compound (*R*)-**368** containing thiocarbamate directing groups was reacted with 2 equiv of phenyl dioxazolone, **369**, in the presence of the Co(III) catalyst **370** (20 mol %), AgSbF_6_ (40 mol %), and KOAc (40 mol %) in 1,1,2,2-tetrachloroethane at 100 °C for 12 h, which gave the 3,3′-dibenzamidyl product (*R*)-**371** in 78% yield (Scheme 130). The monobenzamidyl compounds (*R*)-**372** and (*R*)-**373** were obtained in 84% and 75% yields, respectively, by starting with the corresponding monothiocarbamate BINOLs and using **369** (2 equiv), **370** (10 mol %), AgSbF_6_ (20 mol %), and KOAc (30 mol %). Other aryl dioxazolones **374** were also used to react with the monothiocarbamate BINOL to give the corresponding monoamidation products (*R*)-**375** in 76–90% yields.

**Scheme 131:**
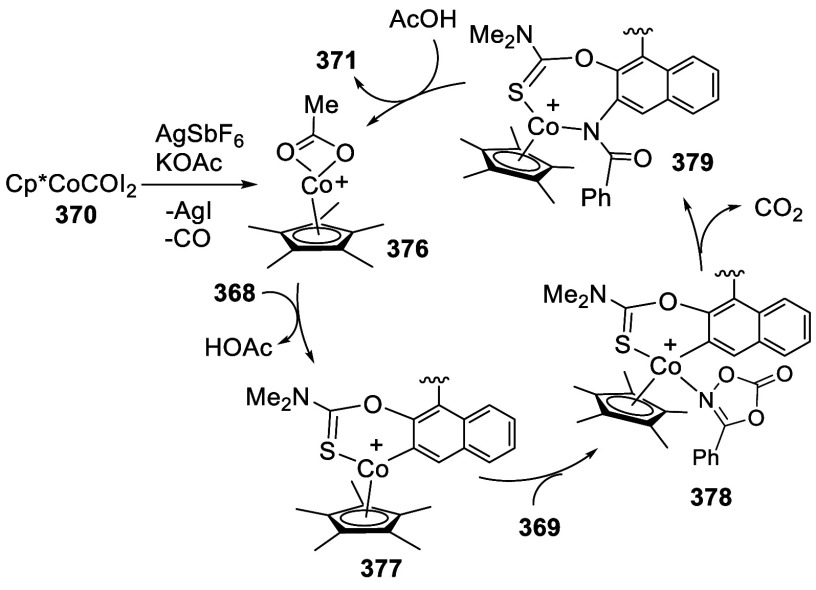
Proposed Mechanism for the Co(III)-Catalyzed C–H Activation and Amidation

Scheme 131 shows a proposed mechanism for the Co-catalyzed C–H activation and amidation. In the first step, the Co(III) complex **370** reacts with AgSbF_6_ and KOAc to form a catalytically active intermediate **376**. Reaction of **376** with the thiocarbamate substrate **368** to form the intermediate **377**. This C–H activation step might be a base-assisted internal electrophilic substitution. Coordination of **377** with the dioxazolone **369** gives **378**. Intermediate **378** undergoes migratory insertion with the extrusion of CO_2_ to give **379**. Proto-demetalation of **379** with acetic acid releases the amidation product **371** and regenerates the catalytically active **376**.

**Scheme 132:**
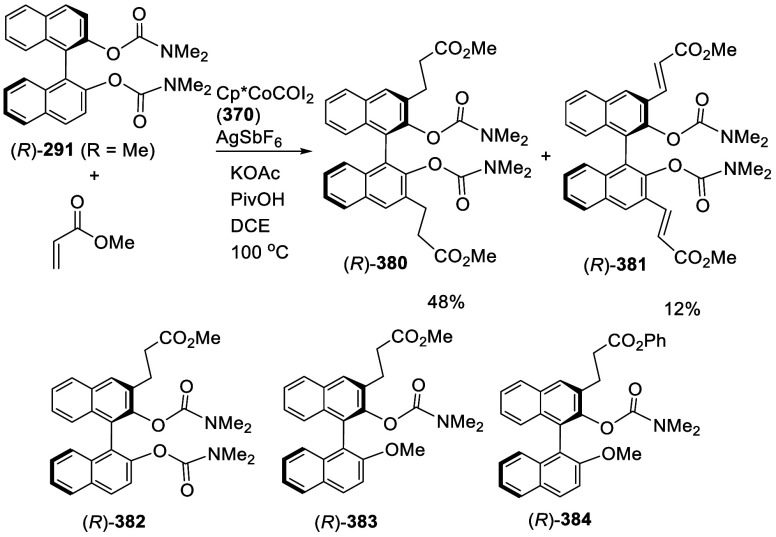
Co-Catalyzed Alkylation at 3-Position of a BINOL Carbamate

Reaction of the BINOL dicarbamate (*R*)-**291** (R = Me) with methyl acrylate in dichloroethane in the presence of the Co(III) catalyst **370** (30 mol %), AgSbF_6_ (60 mol %), KOAc (40 mol %), and pivalic acid (80 mol %) at 100 °C for 18 h gave the 3,3′-dialkylBINOL product (*R*)-**380** in 48% yield (Scheme 132).^344^ A small amount (12%) of the 3,3′-dinvinylBINOL compound (*R*)-**381** was also obtained. The addition of pivalic acid was to reduce the amount of the vinyl product. It was found that an alkylation did not occur for a substrate like (*R*)-**368** that contains a thiocarbamate directing group rather than a carbamate group. The monoalkylated compounds (*R*)-**382** and (*R*)-**383** were obtained in 52% and 60% yields, respectively, in the presence of Cp*CoCOI_2_ (15 mol %), AgSbF_6_ (30 mol %), KOAc (30 mol %), and pivalic acid (50 mol %) under similar conditions. When phenyl acrylate was used, (*R*)-**384** was obtained in 36% yield.

**Scheme 133:**
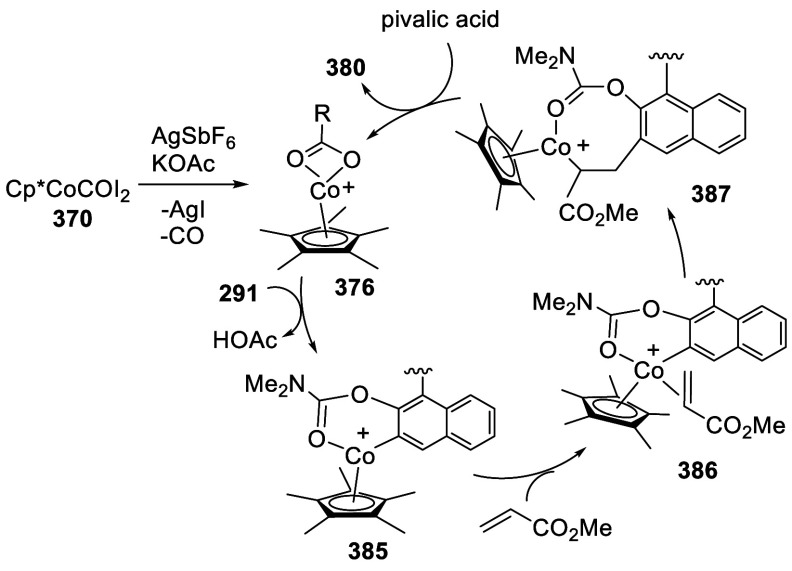
Proposed Mechanism for the Co(III)-Catalyzed C–H Activation and Alkylation

Scheme 133 shows a proposed mechanism for the Co(III)-catalyzed alkylation. Similar to that shown in Scheme 131, the catalytically active intermediate **376** (R = Me) can be generated from the reaction of **370** with AgSbF_6_. The reaction of **376** with the BINOL carbamate **291** will give **385** via C–H activation of the substrate. Coordination of **385** with methyl acrylate gives **386**. The intermediate **386** undergoes a regioselective migratory insertion to give **387**. Proto-demetalation of **387** with pivailic acid will give the alkylated product **380** and regenerate the catalytically active **376** (R = ^t^Bu). The vinyl side product **381** can be generated from the β-elimination of **387**. Thus, addition of pivalic acid suppresses this side reaction.

**Scheme 134:**
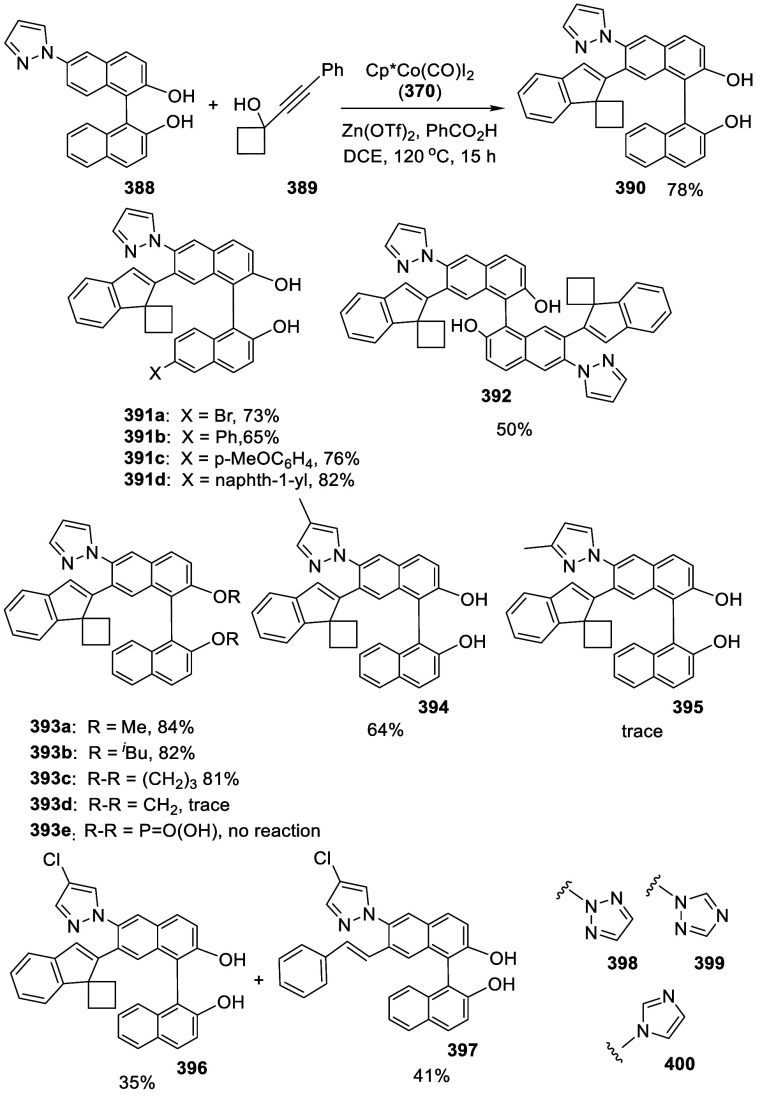
Co-Catalyzed Reaction at 7-Position of BINOL

In 2023, Liu et al. reported the use of a 6-pyrazole group to direct a C–H activation at the 7-position of BINOL by using the Co(III) catalyst **370** (Scheme 134).^343^ Compound **388** was synthesized from the reaction of 6-bromoBINOL with pyrazole in the presence of Cu_2_O and K_2_CO_3_ in DMSO at 130 °C over 2 d under argon. Significant racemization of BINOL was observed in this step. As shown in Scheme 134, in the presence of 5 mol % Cp*Co(CO)I_2_ (**370**), 0.2 equiv of Zn(OTf)_2_, 0.5 equiv of PhCO_2_H, and **388** were heated with 2 equiv of **389** in DCE at 120 °C for 15 h to give **390** in 78% yield. The crystal structure of **390** was obtained by X-ray analysis. When the enantiomerically enriched substrate was used, significant racemization was also observed in this reaction. When the 6′-substituted BINOL starting materials were used, the resulting products **391a**–**d** were also obtained in similar yields. When the 6,6′-di(pyrazole) substituted BINOL was used, the 7,7′-disubstituted product **392** was obtained in 50% yield. The reaction of the acyclic and cyclic alkyl ethers of the BINOL starting material gave products **393a**–**c** in over 80% yields, but only a trace amount of **393d** was obtained for the reaction of the BINOL acetal. Compound **393e** cannot be obtained from the BINOL phosphoric acid. When the R groups of the substrate were methoxymethyls, a similar product was obtained with the hydrolysis of the methoxymethyl groups.

When 4-methyl pyrazole was used as the directing group, the corresponding product **394** was obtained in 64% yield over 48 h. Only trace amount of **395** was observed when 3-methyl pyrazole was used as the directing group, probably due to steric hindrance. When 4-chloropyrazole was used, besides the expected product **396**, a styrenyl product **397** was also obtained due to the C–C bond cleavage of **389** at the tertiary carbon. Other aza-cycles such as **398**–**400** were found to be ineffective for this reaction.

When various substitutents were introduced to the phenyl group of **389** for the reaction with **388**, the corresponding products were obtained except when the strong electron-withdrawing groups like *p*-CO_2_Me or *p*-NO_2_ was incorporated, which gave little or no reaction. When the cyclohexanol derivative **401** was used, product **402** was obtained in 65% yield. When phenyl acetylene and diphenyl acetylene were used in place of **389**, products **403** and **404** were obtained in 87 and 88% yields, respectively.
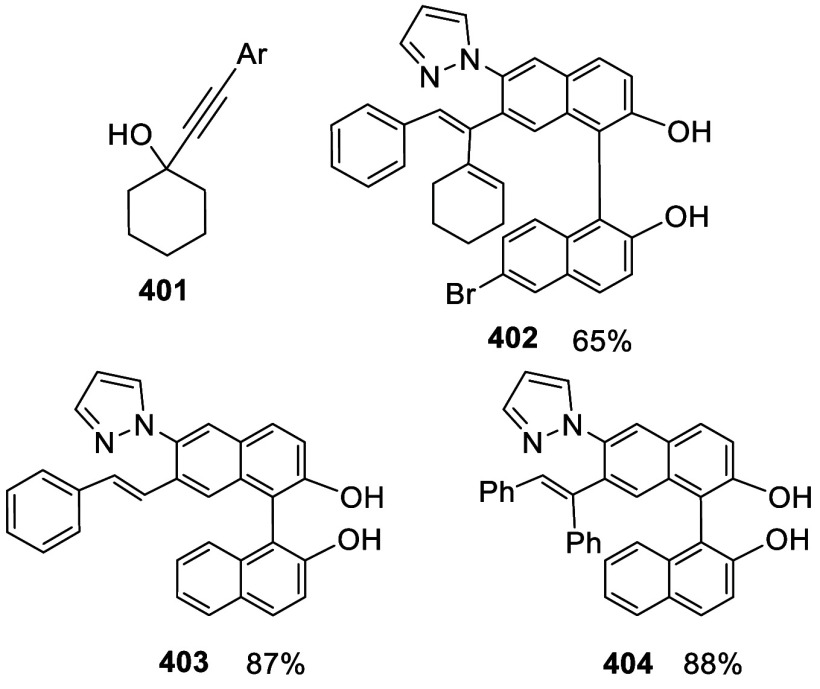


**Scheme 135:**
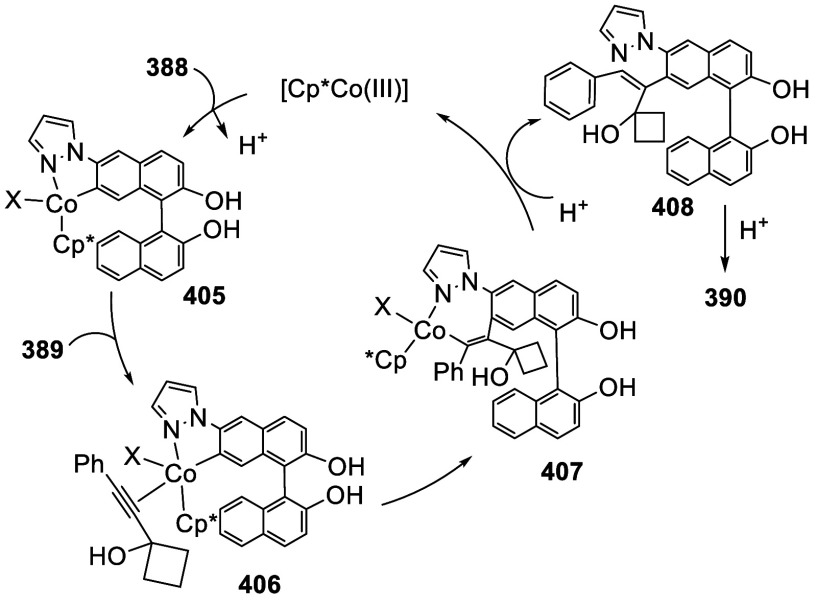
Proposed Mechanism for the Co(III)-Catalyzed C–H Activation

Scheme 135 shows a proposed mechanism for the Co(III)-catalyzed conversion of **388**. The Cp*Co(III)-promoted C–H activation of **388** will give the intermediate **405** directed by the 6-pyrazole group to the 7-position, probably due to the less steric hindrance than the 5-position. Coordination of **389** to **405** will give **406**. Migratory insertion of **406** will give **407**. Proto-demetalation of **407** will give **408** and regenerate the catalytically active Co(III) complex. Under acidic conditions, **408** undergoes an intramolecular Friedel–Crafts alkylation to give the final product **390**.

### 8.4 Ir-Catalyzed Borylation at 3- and 6-Positions

**Scheme 136:**
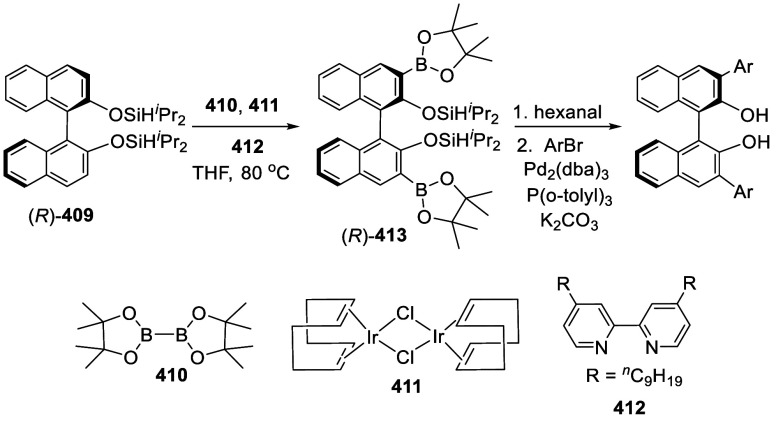
Ir(I)-Catalyzed Borylation of a BINOL Silane

Activation of a C–H bond to form a C–B bond is synthetically useful because of the diverse transformation of C–B bonds. In 2014, Ahmed and Clark^345^ adopted the Hartiwig’s Ir-catalyzed ortho-borylation of phenols for the synthesis of 3,3′-diborylated BINOLs.^346^ When compound (*R*)-**409** containing silane directing groups was reacted with 2 equiv of diborylate **410** in the presence of 1 mol % Ir(I) complex **411** and 4 mol % bipyridyl ligand **412** in THF at 80 °C for 2.5 h, the 3,3′-diborylate BINOL (*R*)-**413** was obtained in 72% yield (Scheme 136). With the addition of hexanal (4 equiv) to quench the borane and silane formed in this step, the subsequent Suzuki coupling of (*R*)-**413** with aryl bromides in the presence of Pd_2_dba_3_, P(*o*-tolyl)_3_, and K_2_CO_3_ was conducted in one-pot to generate the 3,3′-diarylBINOLs (Ar = Ph, 3,5-C_6_H_3_, *p*-CF_3_C_6_H_4_, *p*-NO_2_C_6_H_4_, *p*-MeOC_6_H_4_, 2-naphthyl, 1-naphthyl, etc.) in 43–78% yields.^345^ These products were obtained with high enantiomeric purity (92–99% ee).

**Scheme 137:**
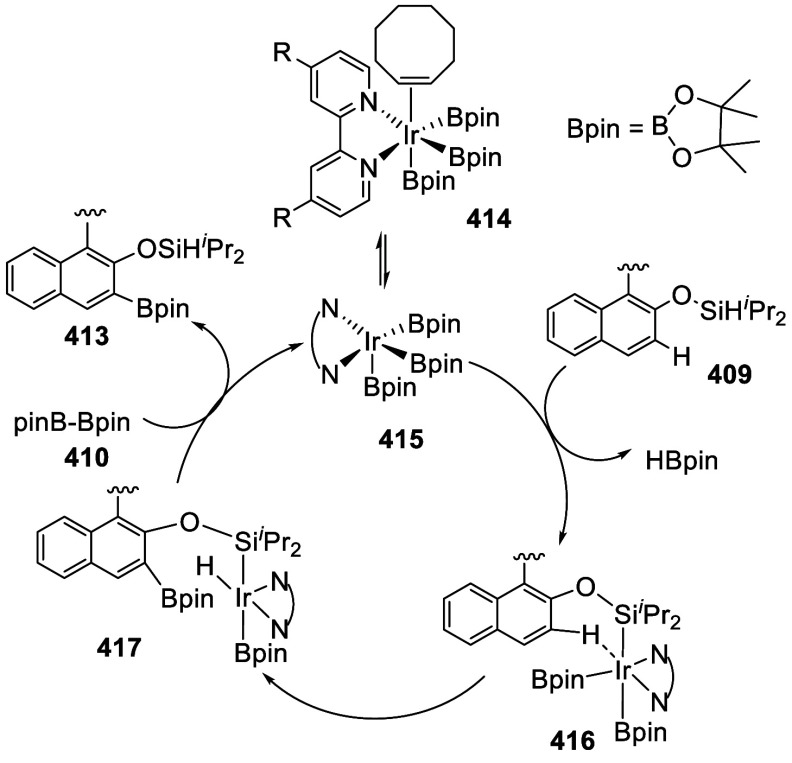
Proposed Mechanism for the Ir-Catalyzed C–H Activation

Scheme 137 shows a proposed mechanism for the Ir(I)-catalyzed borylation.^346^ Complex **414** can be generated from the reaction of the Ir complex **411** with the ligand **412** and the diborylate **410**. Dissociation of the coordinated cyclooctene in **414** generates the active catalyst **415**. Reaction of **415** with the silane substrate **409** generates the intermediate **416**, which can undergo the silyl-directed *ortho*-C–H activation to give the intermediate **417**. Reductive elimination of **417** gives the desired product **413**, and oxidative addition with the diborylate **410** regenerates the active catalyst **415**.

**Scheme 138:**
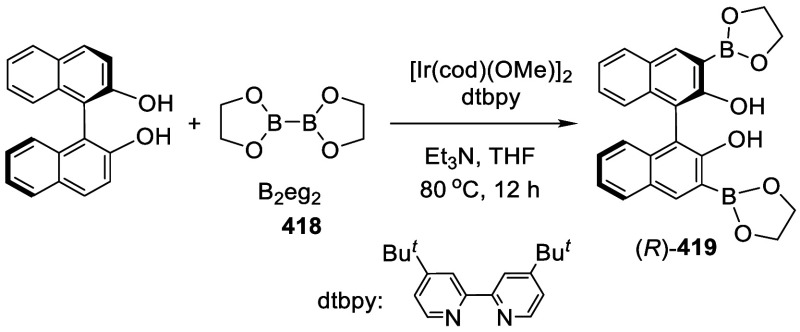
Ir(I)-Catalyzed Borylation of BINOL with B_2_eg_2_

In 2019, Chattopadhyay reported a Ir-catalyzed C–H activation and borylation of BINOL without the need to preincorporate a directing group.^347^ As shown in Scheme 138, the reaction of (*R*)-BINOL with the ethylene glycol-derived diborolane **418** (B_2_eg_2_, 4.0 equiv) catalyzed by [Ir(cod)(OMe)]_2_ (2.0 mol %) in the presence of 4,4′-^*t*^Bu_2_bipyridine (dtbpy, 4.0 mol %) in THF at 80 °C for 12 h gave the 3,3′-diborylated BINOL product (*R*)-**419** in 79% yield, which was converted to the more stable pinacol boroester for isolation. The Ir-catalyzed borylation of BINOL can be combined with the Pd-catalyzed Suzuki coupling with a variety of aryl bromides to generate 3,3′-diarylBINOLs in one-pot in good yields with high enantiomeric purity.

**Scheme 139:**
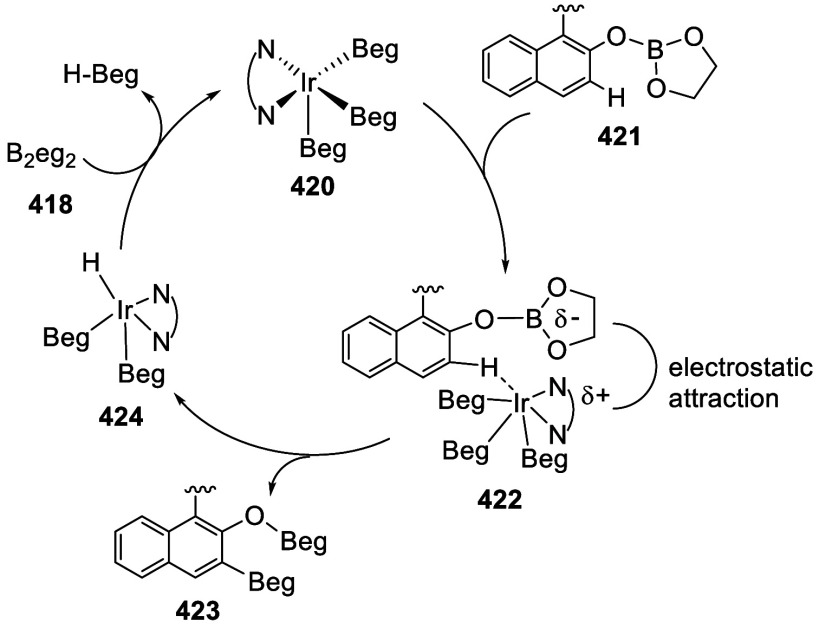
Proposed Mechanism for the Ir-Catalyzed C–H Activation

Scheme 139 shows a proposed mechanism for the Ir-catalyzed borylation of BINOL.^348^ Similar to that shown in Scheme 137 for the formation of the catalytically active Ir(III) complex **415**, complex **420** can be generated from the reaction of [Ir(cod)(OMe)]_2_ with B_2_eg_2_ and dtbpy. Compound **421** can be generated in situ from the reaction of (*R*)-BINOL with B_2_eg_2_. Interaction of **420** with **421** might achieve a transition state as shown by **422** in which the electrostatic attraction between the partially negative OBeg unit and the partially positive bipyridine unit directs the *ortho*-C–H activation and borylation to give **423**. Hydrolysis of **423** gives the 3,3′-diborylated BINOL product (*R*)-**419**. The Ir intermediate **424** can react with B_2_eg_2_ to regenerate the catalytically active **420**.

**Scheme 140:**
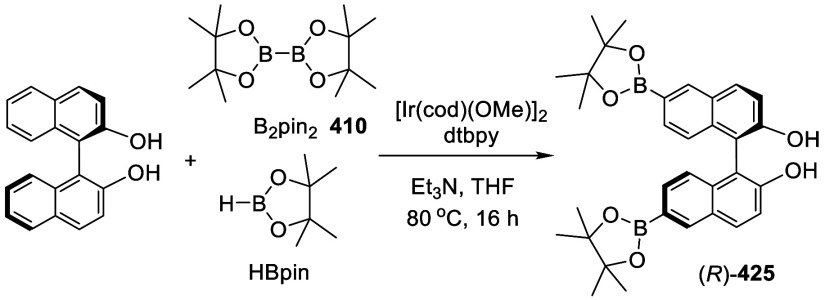
Ir(I)-Catalyzed Borylation of BINOL with B_2_eg_2_

It was found that in the Ir-catalyzed borylation shown in Scheme 138 when the diborolane substrate B_2_eg_2_ (**418**) was replaced with the sterically more bulky B_2_pin_2_ (**410**) in combination with HBpin, the regioselectivity of the C–H activation and borylation changed to the least sterically hindered 6,6′-positions (Scheme 140).^347^ The reaction of (*R*)-BINOL with B_2_pin_2_ (2.5 equiv) and HBpin (3.0 equiv) catalyzed by [Ir(cod)(OMe)]_2_ (1.5 mol %) in the presence of dtbpy (3.0 mol %) in THF at 80 °C for 12 h gave the 6,6′-diborylated BINOL product (*R*)-**425** in 87% yield. This Ir-catalyzed borylation of BINOL can be combined with the Pd-catalyzed Suzuki coupling with a variety of aryl bromides to generate 6,6′-diarylBINOLs in one pot in good yields with high enantiomeric purity.

It was proposed that the bulky Bpin group inhibits the *ortho*-C–H activation as shown in **426** because of the large steric repulsion. This allows the C–H activation and borylation to take place at the least sterically hindered 6-position as shown in **427**.
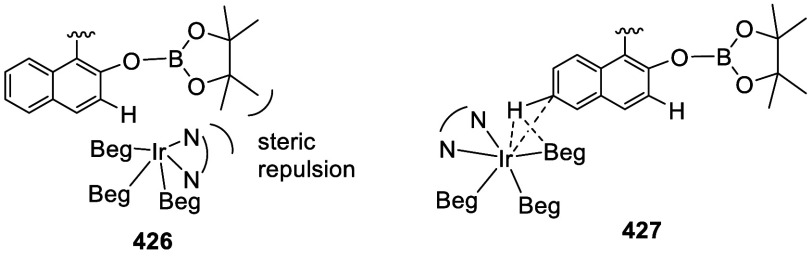


## 9 Summary and Outlook

Three major strategies to selectively substitute the protons of BINOL at the specific positions, including electrophilic substitution, ortho-lithiation, and transition metal-catalyzed C–H activation, have been developed to prepare various functionalized BINOLs for applications in asymmetric catalysis, molecular recognition, chiral sensing, and materials.

Electrophilic substitution of the optically active BINOL displays selectivity at the 3-, 4-, 5-, and 6-positions with the retention of the chiral configuration. The most prevalent pathway involves the substitution at the 6- or 6,6′-positions of BINOL as well as its dialkyl ethers. This pronounced 6-substitution selectivity is attributed to the potent electron-donating effect exerted by the 2-hydroxyl or 2-alkoxy groups on the naphthol rings. When the two hydroxyl groups of BINOL are converted to acetate groups, the reaction with Br_2_ occurs mainly at the 5,5′-positions (see Scheme 21). Thus, reducing the electron-donating ability of the 2,2′-hydroxy groups of BINOL significantly alters the regioselectivity. However, no systematic effort has been devoted to studying the effect of varying electronic properties of the hydroxy groups of BINOL on the regioselective electrophilic substitution. When the 6,6′-positions of BINOL are occupied by the first electrophilic substitution, the next electrophilic reaction occurs at the 5,5′-positions (see Schemes 22–24). Whereas, when the 6,6′-positions of BINOL dialkyl ethers are occupied by the first electrophilic substitution, the next reaction with electrophiles occurs at the 4,4′-positions (see Schemes 26–28). These observations further demonstrate the high sensitivity of the regioselectivity for the electrophilic substitution of BINOL when the electronic property of its two hydroxy groups alters. This calls for a systematic investigation in order to gain a better understating on these effects and to further develop their synthetic applications.

Electrophilic substitution of BINOL is also found to be significantly influenced by the steric environment. When the 6,6′-positions of BINOL are occupied by sterically bulky tertiary butyl groups, the subsequent electrophilic reaction occurs at the less sterically hindered 3,3′-positions rather than the sterically more crowded 5,5′-positions (see Scheme 36, 37) even though the 3,3′-positions are not electronically favorable for electrophilic reaction as discussed in section 2.1. The hydroxyl groups at the 2,2′-positions of BINOL are also found to direct certain electrophilic reactions at the 3,3′-positions when there are possible favorable interactions between the hydroxyl groups and the electrophiles (see Schemes 29–35). Although the 8,8′-positions of BINOL should be electronically favorable for an electrophilic reaction, there are only two reports on low yield electrophilic reactions at the 8-position due to significant steric hindrance around this position (see section 2.1 and Scheme 10 and 28). No electrophilic substitution at the 7-position of BINOL has been reported yet. This position does not sense the resonance electron-donating effect of the 2-hydroxyl group, and it is also at the edge of the node plan of the HOMO orbital of BINOL as shown in Figure 2. It would be both fundamentally interesting and potentially useful to vary the electronic properties of BINOL in order to facilitate the substitution at the 7-position.

Although the 3,3′-positions of BINOL are not electronically favorable for many electrophilic reactions, ortho-lithiation at the 3-position, directed by the functional groups, such as ethers, alkoxymethyl ethers, and carbamates at the 2,2′-positions, have been extensively used to prepare many 3- or 3,3′-substituted BINOLs for diverse applications. Among these reactions, the use of the ortho-lithiation of the methoxymethyl ether of BINOL is the most popular because of the easy introduction and removal of this directing group (see section 7.4).

The use of the transition metal-catalyzed C–H activations of BINOL has shown very promising application in preparing the selectively substituted BINOLs. These reactions generally require the introduction of a functional group to direct the C–H activation at the ortho position. This strategy has allowed selective substitutions at the 3-, 4-, 5-, 6-, and 7- positions. For example, Schemes 138 and 140 show that BINOL can be directly functionalized at the 3,3′- or 6,6′-positions in the presence of an Ir(I) catalyst by using two borylating agents that have very different steric demands. In principle, all the positions on BINOL can be selectively functionalized by using the transition metal-catalyzed C–H activation as long as an appropriate directing group is introduced at the adjacent position. However, there are still significant challenges in using this strategy to prepare structurally modified BINOLs for various applications.

Although functionalization at the 3,3′-positions has become more readily applicable because of the easy introduction and removal of the directing groups at the 2,2′-positions via transformation of the two hydroxy groups, and introduction and removal of the directing groups at other positions are not as straightforward and can become difficult. No work has been reported to remove the directing groups at the 3- and 6-positions that were used to direct the reactions at the 4-, 7-, and 5-positions. There is also little research conducted on the transition metal-catalyzed C–H activation at the 5- and 8-positions of BINOL. As shown in Schemes 129 and 134, the functional group at the 6-position directs the reaction at the less sterically hindered 7-position first. Only when the 7-position is occupied, can the reaction take place at the more sterically hindered 5-position. In order to introduce a substituent to the 8-position, one needs to introduce a directing group to the 7-position, which requires a directing group at 6-position. The directing group at the 6-position will also direct a substitution at the 5-position after the 7-position is occupied. Thus, this approach would fill the 6-, 7-, and 5-positions before the 8-position could be filled. It remains a challenge to selectively introduce a substituent to the 8-position. A few of the C–H activation reactions also lead to racemization of BINOL because of the high temperature and strongly basic conditions. For example, Scheme 120 shows that the Rh(I)-catalyzed conversion of BINOL to the synthetically very useful 3,3′-diarylBINOLs can be conducted in one step, but the products were completely racemized. Therefore, continuous research in this area is necessary in order to develop efficient methods to selectively functionalize BINOL especially at positions other than the 3- and 6-positions.

In summary, the techniques outlined in this article, including the electrophilic substitution, the ortho lithiation, and the transition metal-catalyzed C–H functionalization at various positions, have enabled researchers to leverage the distinctive structure of BINOL to craft a wide range of chiral materials for exciting applications. It is anticipated that research into the development and understanding of BINOL’s reactivity, as well as further exploration of its applications, will continue to expand. The versatility of BINOL will always present challenges to the imagination of researchers in diverse fields.

## Dedication

Dedicated to Professor Joseph M. O’Connor at the University of California San Diego on the occasion of his 70th birthday.

## References

[ref1] Jacques J.; Fouquey C.; Viterbo R. Enantiomeric Cyclic Binaphthyl Phosphoric Acids as Resolving Agents. Tetrahedron Lett. 1971, 12, 4617–4620. 10.1016/S0040-4039(01)97544-6.

[ref2] Kyba E. P.; Gokel G. W.; de Jong F.; Koga K.; Sousa L. R.; Siegel M. G.; Kaplan L.; Sogah G. D. Y.; Cram D. J. Host-Guest Complexation. 7. The Binaphthyl Structural Unit in Host Compounds. J. Org. Chem. 1977, 42, 4173–4184. 10.1021/jo00862a001.

[ref3] Kazlauskas R. J. Resolution of Binaphthols and Spirobiindanols Using Cholesterol Esterase. J. Am. Chem. Soc. 1989, 111, 4953–4959. 10.1021/ja00195a059.

[ref4] Jacques J.; Fouquey C. Enantiomeric (*S*)-(+)- and (*R*)-(−)-1,1′-Binaphthyl-2,2′-diyl Hydrogen Phosphate Organic Synthesis; Wiley, 1989; Vol. 67, p 1 10.1002/0471264180.os067.01

[ref5] Kawashima M.; Hirayama A. Direct Optical Resolution of 2,2′-Dihydroxy-1,1′-binaphthyl. Chem. Lett. 1990, 19, 2299–2300. 10.1246/cl.1990.2299.

[ref6] Fabbri D.; Delogu G.; De Lucchi O. Preparation of Enantiomerically Pure l,l’-Binaphthalene-2,2’-diol and 1, l’-Binaphthalene-2,2’-dithiol. J. Org. Chem. 1993, 58, 1748–1750. 10.1021/jo00059a025.

[ref7] Brunel J.-M.; Buono G. A New and Efficient Method for the Resolution of 1,1’-Binaphthalene-2,2’-diol. J. Org. Chem. 1993, 58, 7313–7314. 10.1021/jo00077a072.

[ref8] Wang M.; Liu S. Z.; Liu J.; Hu B. F. Diastereoselective Synthesis of 1,1 ’-Binaphthyl-2,2’-diol. J. Org. Chem. 1995, 60, 7364–7365. 10.1021/jo00127a053.

[ref9] Chow H.-F.; Wan C.-W.; Ng M.-K. A Versatile Method for the Resolution and Absolute Configuration Assignment of Substituted 1,1’-Bi-2-naphthols. J. Org. Chem. 1996, 61, 8712–8714. 10.1021/jo9610927.

[ref10] Shan Z. X.; Wang G. P.; Duan B.; Zhao D. J. Preparation of Enantiomerically Pure 1,1′-Bi-2-naphthol via Cyclic Borate Ester. Tetrahedron: Asymmetry 1996, 7, 2847–2850. 10.1016/0957-4166(96)00375-8.

[ref11] Moustafa G. A. I.; Oki Y.; Akai S. Lipase-Catalyzed Dynamic Kinetic Resolution of C_1_- and C_2_-Symmetric Racemic Axially Chiral 2,2’-Dihydroxy-1,1’-biaryls. Angew. Chem., Int. Ed. 2018, 57, 10278–10282. 10.1002/anie.201804161. 29704286

[ref12] Li H.-H.; Zhang J.-Y.; Li S.; Wang Y.-B.; Cheng J. K.; Xiang S.-H.; Tan B. Asymmetric Synthesis of Binaphthyls through Photocatalytic Cross-Coupling and Organocatalytic Kinetic Resolution. Science China: Chem. 2022, 65, 1142–1148. 10.1007/s11426-022-1246-8.

[ref13] Tanaka K.; Okada T.; Toda F. Separation of the Enantiomers of 2,2′-Dihydroxy-1,1′-binaphthyl and 10,10′-Dihydroxy-9,9′-biphenanthryl by Complexation with N-Alkylcinchonidinium Halides. Angew. Chem., Int. Ed. Engl. 1993, 32, 1147–1148. 10.1002/anie.199311471.

[ref14] Toda F.; Tanaka K.; Stein Z.; Goldberg I. Optical Resolution of Binaphthyl and Biphenanthryl Diols by Inclusion Crystallization with N-Alkylcinchonidium Halides. Structural Characterization of the Resolved Materials. J. Org. Chem. 1994, 59, 5748–5751. 10.1021/jo00098a038.

[ref15] Hu Q.-S.; Vitharana D. R.; Pu L. An Efficient and Practical Direct Resolution of Racemic 1,1′-Bi-2-naphthol to Both of Its Pure Enantiomers. Tetrahedron: Asymmetry 1995, 6, 2123–2126. 10.1016/0957-4166(95)00280-3.

[ref16] Cai D.; Hughes D.; Verhoeven T. R.; Reider D. J. Simple and Efficient Resolution of l, 1′-Bi-2-naphthol. Tetrahedron Lett. 1995, 36, 7991–7994. 10.1016/0040-4039(95)01722-T.

[ref17] da Silva E. M.; Vidal H. D. A.; Januário M. A. P.; Corrêa A. G. Advances in the Asymmetric Synthesis of BINOL Derivatives. Molecules 2023, 28, 12 10.3390/molecules28010012. PMC9821997 36615207

[ref18] Tkachenko N. V.; Bryliakov K. P. Transition Metal Catalyzed Aerobic Asymmetric Coupling of 2-Naphthols. Mini-Rev. Org. Chem. 2019, 16, 392–398. 10.2174/1570193X15666180418153713.

[ref19] Miyano S.; Tobita M.; Hashimoto H. Asymmetric Synthesis of Axially Dissymmetric 1,1′-Binaphthyls via an Intramolecular Ullmann Coupling Reaction of (*R*)- and (*S*)-2,2′-Bis(1-bromo-2-naphthylcarbonyloxy)-1,1′-binaphthyl. Bull. Chem. Soc. Jpn. 1981, 54, 3522–3526. 10.1246/bcsj.54.3522.

[ref20] Brussee J.; Groenendijk J. L. G.; te Koppele J. M.; Jansen A. C. A. On the Mechanism of the Formation of S(−)-(1.1’-Binaphthalene)-2,2’-diol via Copper(II) Amine Complexes. Tetrahedron 1985, 41, 3313–3319. 10.1016/S0040-4020(01)96682-7.

[ref21] Yamamoto K.; Fukushima H.; Yumioka H.; Nakazaki M. Absolute Configurations of Novel Axially Dissymmetric 10,10′-Dihydroxy-9,9′-biphenanthryl and Its Related Compounds. Bull. Chem. Soc. Jpn. 1985, 58, 3633–3634. 10.1246/bcsj.58.3633.

[ref22] Smrcina M.; Poláková J.; Vyskocil S.; Kocovský P. Synthesis of Enantiomerically Pure Binaphthyl Derivatives. Mechanism of the Enantioselective, Oxidative Coupling of Naphthols and Designing a Catalytic Cycle. J. Org. Chem. 1993, 58, 4534–4538. 10.1021/jo00069a010.

[ref23] Osa T.; Kashiwagi Y.; Yanagisawa Y.; Bobbitt J. M. Enantioselective, Electrocatalytic Oxidative Coupling of Naphthol, Naphthyl Ether and Phenanthrol on a TEMPO-modified Graphite Felt Electrode in the Presence of (−)-Sparteine (TEMPO = 2,2,6,6-tetramethylpiperidin-1-yloxyl). J. Chem. Soc. Chem. Commun. 1994, 2535–2537. 10.1039/C39940002535.

[ref24] Irie R.; Masutani K.; Katsuki T. Asymmetric Aerobic Oxidative Coupling of 2-Naphthol Derivatives Catalyzed by Photo-Activated Chiral (NO)Ru(II)-Salen Complex. Synlett 2000, 2000, 1433–1436. 10.1055/s-2000-7654.

[ref25] Barhate N. B.; Chen C. T. Catalytic Asymmetric Oxidative Couplings of 2-Naphthols by Tridentate N-Ketopinidene-Based Vanadyl Dicarboxylates. Org. Lett. 2002, 4, 2529–2532. 10.1021/ol026156g. 12123368

[ref26] Luo Z.; Liu Q.; Gong L.; Cui X.; Mi A.; Jiang Y. Novel Achiral Biphenol-Derived Diastereomeric Oxovanadium(IV) Complexes for Highly Enantioselective Oxidative Coupling of 2-Naphthols. Angew. Chem. 2002, 114, 4714–4717. 10.1002/1521-3757(20021202)114:23<4714::AID-ANGE4714>3.0.CO;2-3. 12458529

[ref27] Li X.; Hewgley J. B.; Mulrooney C. A.; Yang J.; Kozlowski M. C. Enantioselective Oxidative Biaryl Coupling Reactions Catalyzed by 1,5-Diazadecalin Metal Complexes: Efficient Formation of Chiral Functionalized BINOL Derivatives. J. Org. Chem. 2003, 68, 5500–5511. 10.1021/jo0340206. 12839440

[ref28] Gao J.; Reibenspies J. H.; Martell A. E. Structurally Defined Catalysts for Enantioselective Oxidative Coupling Reactions. Angew. Chem. 2003, 115, 6190–6194. 10.1002/ange.200351978. 14679556

[ref29] Somei H.; Asano Y.; Yoshida T.; Takizawa S.; Yamataka H.; Sasai H. Dual Activation in a Homolytic Coupling Reaction Promoted by an Enantioselective Dinuclear Vanadium(IV) Catalyst. Tetrahedron Lett. 2004, 45, 1841–1844. 10.1016/j.tetlet.2004.01.007.

[ref30] Guo Q.-X.; Wu Z.-J.; Luo Z.-B.; Liu Q.-Z.; Ye J.-L.; Luo S.- W.; Cun L.-F.; Gong L.-Z. Highly Enantioselective Oxidative Couplings of 2-Naphthols Catalyzed by Chiral Bimetallic Oxovanadium Complexes with Either Oxygen or Air as Oxidant. J. Am. Chem. Soc. 2007, 129, 13927–13938. 10.1021/ja074322f. 17956093

[ref31] Hewgley J. B.; Stahl S. S.; Kozlowski M. C. Mechanistic Study of Asymmetric Oxidative Biaryl Coupling: Evidence for Self-Processing of the Copper Catalyst to Achieve Control of Oxidase vs Oxygenase Activity. J. Am. Chem. Soc. 2008, 130, 12232–12233. 10.1021/ja804570b. 18710234 PMC2897702

[ref32] Egami H.; Katsuki T. Iron-Catalyzed Asymmetric Aerobic Oxidation: Oxidative Coupling of 2-Naphthols. J. Am. Chem. Soc. 2009, 131, 6082–6083. 10.1021/ja901391u. 19361160

[ref33] Alamsetti S. K.; Poonguzhali E.; Ganapathy D.; Sekar G. Enantioselective Oxidative Coupling of 2-Naphthol Derivatives by Copper-(R)-1,1′-Binaphthyl-2,2′-diamine-TEMPO Catalyst. Adv. Synth. Catal. 2013, 355, 2803–2808. 10.1002/adsc.201300513.

[ref34] Tkachenko N. V.; Lyakin O. Y.; Samsonenko D. G.; Talsi E. P.; Bryliakov K. P. Highly Efficient Asymmetric Aerobic Oxidative Coupling of 2-Naphthols in the Presence of Bioinspired Iron Aminopyridine Complexes. Catal. Commun. 2018, 104, 112–117. 10.1016/j.catcom.2017.10.025.

[ref35] Narute S.; Parnes R.; Toste F. D.; Pappo D. Enantioselective Oxidative Homocoupling and Cross-Coupling of 2-Naphthols Catalyzed by Chiral Iron Phosphate Complexes. J. Am. Chem. Soc. 2016, 138, 16553–16560. 10.1021/jacs.6b11198. 27959518

[ref36] Meca L.; Řeha D.; Havlas Z. Racemization Barriers of 1,1′- Binaphthyl and 1,1′-Binaphthalene-2,2′-diol: A DFT Study. J. Org. Chem. 2003, 68, 5677–5680. 10.1021/jo034344u. 12839462

[ref37] Whitesell J. K. *C*_2_ Symmetry and Asymmetric Induction. Chem. Rev. 1989, 89, 1581–1590. 10.1021/cr00097a012.

[ref38] Rosini C.; Franzini L.; Raffaelli A.; Salvadori P. Synthesis and Applications of Binaphthylic *C*_2_-Symmetry Derivatives as Chiral Auxiliaries in Enantioselective Reactions. Synthesis 1992, 1992, 503–517. 10.1055/s-1992-26147.

[ref39] Pu L. 1,1‘-Binaphthyl Dimers, Oligomers, and Polymers: Molecular Recognition, Asymmetric Catalysis, and New Materials. Chem. Rev. 1998, 98, 2405–2494. 10.1021/cr970463w. 11848968

[ref40] Chen Y.; Yekta S.; Yudin A. K. Modified BINOL Ligands in Asymmetric Catalysis. Chem. Rev. 2003, 103, 3155–3212. 10.1021/cr020025b. 12914495

[ref41] Kočovský P.; Vyskočil S.; Smrčina M. Non-Symmetrically Substituted 1,1’-Binaphthyls in Enantioselective Catalysis. Chem. Rev. 2003, 103, 3213–3245. 10.1021/cr9900230. 12914496

[ref42] Telfer S. G.; Kuroda R. 1,1′-Binaphthyl-2,2′-diol and 2,2′-Diamino-1,1′-binaphthyl: Versatile Frameworks for Chiral Ligands in Coordination and Metallosupramolecular Chemistry. Coord. Chem. Rev. 2003, 242, 33–46. 10.1016/S0010-8545(03)00026-2.

[ref43] Brunel J. M. BINOL: A Versatile Chiral Reagent. Chem. Rev. 2005, 105, 857–897. 10.1021/cr040079g. 15755079

[ref44] Shibasaki M.; Matsunaga S. Design and Application of Linked-BINOL Chiral Ligands in Bifunctional Asymmetric Catalysis. Chem. Soc. Rev. 2006, 35, 269–279. 10.1039/b506346a. 16505920

[ref45] Eberhardt L.; Armspach D.; Harrowfield J.; Matt D. BINOL-derived Phosphoramidites in Asymmetric Hydrogenation: Can the Presence of a Functionality in the Amino Group Influence the Catalytic Outcome?. Chem. Soc. Rev. 2008, 37, 839–864. 10.1039/b714519e. 18362987

[ref46] Parmar D.; Sugiono E.; Raja S.; Rueping M. Complete Field Guide to Asymmetric BINOL-Phosphate Derived Brønsted Acid and Metal Catalysis: History and Classification by Mode of Activation; Brønsted Acidity, Hydrogen Bonding, Ion Pairing, and Metal Phosphates. Chem. Rev. 2014, 114, 9047–9153. 10.1021/cr5001496. 25203602

[ref47] Pu L. Enantioselective Fluorescent Sensors: A Tale of BINOL. Acc. Chem. Res. 2012, 45, 150–163. 10.1021/ar200048d. 21834528

[ref48] Pu L. Asymmetric Functional Organozinc Additions to Aldehydes Catalyzed by 1,1′-Bi-2-naphthols (BINOLs). Acc. Chem. Res. 2014, 47, 1523–1535. 10.1021/ar500020k. 24738985 PMC4033666

[ref49] Pu L. Simultaneous Determination of Concentration and Enantiomeric Composition in Fluorescent Sensing. Acc. Chem. Res. 2017, 50, 1032–1040. 10.1021/acs.accounts.7b00036. 28287702

[ref50] Pu L. Enantioselective Fluorescent Recognition of Free Amino Acids: Challenges and Opportunities. Angew. Chem., Int. Ed. 2020, 59, 21814–21828. 10.1002/anie.202003969. 32602243

[ref51] Li G.; Liu F.; Wu M. BINOLs Modified at the 3,3′-Positions: Chemists’ Preferred Choice in Asymmetric Catalysis. Arkivoc 2015, 6, 140–174. 10.3998/ark.5550190.p009.060.

[ref52] Nájera C.; Sansano J. M.; Saá J. M. Bifunctional Binols: Chiral 3,3′-Bis(aminomethyl)-1,1′-bi-2-naphthols (Binolams) in Asymmetric Catalysis. Eur. J. Org. Chem. 2009, 2009, 2385–2400. 10.1002/ejoc.200801069.

[ref53] Sogah G. D. Y.; Cram D. J. Host-Guest Complexation. 14. Host Covalently Bound to Polystyrene Resin for Chromatographic Resolution of Enantiomers of Amino Acid and Ester Salts1. J. Am. Chem. Soc. 1979, 101, 3035–3042. 10.1021/ja00505a034. 1262635

[ref54] Ishitani H.; Ueno M.; Kobayashi S. Enantioselective Mannich-Type Reactions Using a Novel Chiral Zirconium Catalyst for the Synthesis of Optically Active β-Amino Acid Derivatives. J. Am. Chem. Soc. 2000, 122, 8180–8186. 10.1021/ja001642p.

[ref55] Kieffer M. E.; Repka L. M.; Reisman S. E. Enantioselective Synthesis of Tryptophan Derivatives by a Tandem Friedel-Crafts Conjugate Addition/Asymmetric Protonation Reaction. J. Am. Chem. Soc. 2012, 134, 5131–5137. 10.1021/ja209390d. 22390403 PMC3310256

[ref56] Ito K.; Takahashi M.; Hoshino T.; Nishiki M.; Ohba Y. Study on Host-Guest Complexation of Anions Based on 2,2’-Dihydroxyl-1,1’-Binaphtalene Derivatives. Lett. Org. Chem. 2006, 3, 735–740. 10.2174/157017806779025898.

[ref57] Dong J.; Li T.; Dai C.; Weng W.; Xue X.; Zhang Y.; Zeng Q. Protecting-Group-Free Palladium-Catalyzed Hydroxylation, C-O and C-N Coupling of Chiral 6-Bromo- and 6,6’-Dibromo- 1,1’-binaphthols. Appl. Organometal. Chem. 2013, 27, 337–340. 10.1002/aoc.2982.

[ref58] Yang L.; Yan Y.; Cao N.; Hao J.; Li G.; Zhang W.; Cao R.; Wang C.; Xiao J.; Xue D. Ni(I)-Catalyzed Hydroxylation of Aryl Halides with Water under Thermal Catalysis. Org. Lett. 2022, 24, 9431–9435. 10.1021/acs.orglett.2c03840. 36534081

[ref59] Alcazar V.; Moran J. R.; Diederich F. Chiral Molecular Clefts for Dicarboxylic Acid Complexation. Israel J. Chem. 1992, 32, 69–77. 10.1002/ijch.199200010.

[ref60] Hu Q.-S.; Vitharana D.; Liu G.; Jain V.; Wagaman M. W.; Zhang L.; Lee T. R.; Pu L. Conjugated Polymers with Main Chain Chirality. 1. Synthesis of an Optically Active Poly (arylenevinylene). Macromolecules 1996, 29, 1082–1084. 10.1021/ma951414t.

[ref61] Hu Q.-S.; Vitharana D.; Liu G.; Jain V.; Pu L. Conjugated Polymers with Main Chain Chirality. 2. Synthesis of Optically Active Polyarylenes. Macromolecules 1996, 29, 5075–5082. 10.1021/ma960249u.

[ref62] Hu Q.-S.; Vitharana D.; Zheng X.-F.; Wu C.; Kwan C. M. S.; Pu L. Poly(1,1‘-bi-2-naphthol)s: Synthesis, Characterization, and Application in Lewis Acid Catalysis. J. Org. Chem. 1996, 61, 8370–8377. 10.1021/jo961407i.

[ref63] Musick K. Y.; Hu Q.-S.; Pu L. Synthesis of Binaphthyl- Oligothiophene Copolymers with Emissions of Different Colors: Systematically Tuning the Photoluminescence of Conjugated Polymers. Macromolecules 1998, 31, 2933–2942. 10.1021/ma9711710.

[ref64] Park J.-W.; Ediger M. D.; Green M. M. Chiral Studies in Amorphous Solids: The Effect of the Polymeric Glassy State on the Racemization Kinetics of Bridged Paddled Binaphthyls. J. Am. Chem. Soc. 2001, 123, 49–56. 10.1021/ja0023231. 11273600

[ref65] Jin L.-M.; Li Y.; Ma J.; Li Q. Synthesis of Novel Thermally Reversible Photochromic Axially Chiral Spirooxazines. Org. Lett. 2010, 12, 3552–3555. 10.1021/ol1014152. 20670019

[ref66] An S.; Tang G.; Zhong Y.; Ma L.; Liu Q. Novel π-Expanded Chrysene-based Axially Chiral Molecules: 1,1′-Bichrysene-2,2′-diols and Thiophene Analogs. J. Chem. Res. 2020, 44, 641–645. 10.1177/1747519820914837.

[ref67] Yamaguchi T.; Takami S. Synthesis and Photochromic Reaction of 6,6′-Bis(diarylethenyl)-1,1′-binaphthyl-2,2′-diether. Chem. Lett. 2022, 51, 610–613. 10.1246/cl.220083.

[ref68] Verga D.; Percivalle C.; Doria F.; Porta A.; Freccero M. Protecting Group Free Synthesis of 6-Substituted Naphthols and Binols. J. Org. Chem. 2011, 76, 2319–2323. 10.1021/jo1025892. 21384814

[ref69] Chen R.; Qian C.; de Vries J. G. Asymmetric Epoxidation of α,β-Unsaturated Ketones Catalyzed by Chiral Ytterbium Complexes. Tetrahedron Lett. 2001, 42, 6919–6921. 10.1016/S0040-4039(01)01336-3.

[ref70] Li Z. – B.; Lin J.; Zhang H. – C.; Sabat M.; Hyacinth M.; Pu L. Macrocyclic Bisbinaphthyl Fluorophores and Their Acyclic Analogues: Signal Amplification and Chiral Recognition. J. Org. Chem. 2004, 69, 6284–6293. 10.1021/jo049366a. 15357587

[ref71] Shi M.; Chen L.-H.; Li C.-Q. Chiral Phosphine Lewis Bases Catalyzed Asymmetric Aza-Baylis–Hillman Reaction of N-Sulfonated Imines with Activated Olefins. J. Am. Chem. Soc. 2005, 127, 3790–3800. 10.1021/ja0447255. 15771513

[ref72] Matsushita S.; Yan B.; Yamamoto S.; Jeong Y. S.; Akagi K. Helical Carbon and Graphite Films Prepared from Helical Poly(3,4-ethylenedioxythiophene) Films Synthesized by Electrochemical Polymerization in Chiral Nematic Liquid Crystals. Angew. Chem., Int. Ed. 2014, 53, 1659–1663. 10.1002/anie.201308462. 24453181

[ref73] Liang Z.; Chen J.; Chen X.; Zhang K.; Lv J.; Zhao H.; Zhang G.; Xie C.; Zong L.; Jia X. Porous Organic Polymer Supported Rhodium as a Heterogeneous Catalyst for Hydroformylation of Alkynes to α,β-Unsaturated aldehydes. Chem. Commun. 2019, 55, 13721–13724. 10.1039/C9CC06834A. 31658306

[ref74] Sasai H.; Tokugana T.; Watanabe S.; Suzuki T.; Itoh N.; Shibasaki M. Efficient Diastereoselective and Enantioselective Nitroaldol Reactions from Prochiral Starting Materials: Utilization of La-Li-6,6’-Disubstituted BINOL Complexes as Asymmetric Catalysts. J. Org. Chem. 1995, 60, 7388–7389. 10.1021/jo00128a005.

[ref75] Nakamura Y.; Takeuchi S.; Ohgo Y.; Curran D. P. Asymmetric Alkylation of Aromatic Aldehydes with Diethylzinc Catalyzed by a Fluorous BINOL-Ti Complex in an Organic and Fluorous Biphase System. Tetrahedron Lett. 2000, 41, 57–60. 10.1016/S0040-4039(99)01999-1.

[ref76] Nakamura Y.; Takeuchi S.; Okumura K.; Ohgo Y.; Curran D. P. Recyclable Fluorous Chiral Ligands and Catalysts: Asymmetric Addition of Diethylzinc to Aromatic Aldehydes Catalyzed by Fluorous BINOL-Ti Complexes. Tetrahedron 2002, 58, 3963–3969. 10.1016/S0040-4020(02)00245-4.

[ref77] Yamashita Y.; Ishitani H.; Shimizu H.; Kobayashi S. Highly anti-Selective Asymmetric Aldol Reactions Using Chiral Zirconium Catalysts. Improvement of Activities, Structure of the Novel Zirconium Complexes, and Effect of a Small Amount of Water for the Preparation of the Catalysts. J. Am. Chem. Soc. 2002, 124, 3292–3302. 10.1021/ja016293t. 11916413

[ref78] Ma L.; Hu Q.-S.; Musick K.; Vitharana D.; Wu C.; Kwan C. M. S.; Pu L. Conjugated Polymers with Main Chain Chirality. 3. Synthesis of Optically Active Poly(aryleneethynylene)s. Macromolecules 1996, 29, 5083–5090. 10.1021/ma960250t.

[ref79] Ma L.; Hu Q.-S.; Pu L. Synthesis of an Optically Active Poly(aryleneethynylene) Containing Extended Conjugation in the Repeat Unit. Tetrahedron: Asymmetry 1996, 7, 3103–3106. 10.1016/0957-4166(96)00408-9.

[ref80] Cheng H.; Ma L.; Hu Q.-S.; Zheng X.-F.; Anderson J.; Pu L. The First Sterically Regular Chiral Conjugated Crown Ether Polymer. Tetrahedron: Asymmetry 1996, 7, 3083–3086. 10.1016/0957-4166(96)00403-X.

[ref81] Ma L.; Hu Q.-S.; Vitharana D.; Wu C.; Kwan C. M. S.; Pu L. A New Class of Chiral Conjugated Polymers with a Propeller-Like Structure. Macromolecules 1997, 30, 204–218. 10.1021/ma961192e.

[ref82] Ma L.; Pu L. A Dipole Oriented Chiral Conjugated Polymer and Multipolar Chiral Macrocycles. Macromol. Chem. Physic. 1998, 199, 2395–2401. 10.1002/(SICI)1521-3935(19981101)199:11<2395::AID-MACP2395>3.0.CO;2-M.

[ref83] Simonsen K. B.; Jørgensen K. A.; Hu Q.-S.; Pu L. The First Highly Diastereo- and Enantioselective Polymeric Catalyst for the 1,3-Cycloaddition Reaction of Nitrones with Alkenes. Chem. Commun. 1999, 811–812. 10.1039/a901316d.

[ref84] Johannsen M.; Jørgensen K. A.; Zheng X.-F.; Hu Q.-S.; Pu L. A Highly Enantioselective Hetero-Diels-Alder Reaction Catalyzed by Chiral Polybinaphthyl-Aluminum Complexes. J. Org. Chem. 1999, 64, 299–301. 10.1021/jo981466r. 11674119

[ref85] Feng L.; Liang F.; Wang Y.; Xu M.; Wang X. A Highly Sensitive Water-Soluble System to Sense Glucose in Aqueous Solution. Org. Bio. Chem. 2011, 9, 2938–2942. 10.1039/c0ob01224f. 21380440

[ref86] Feng L.-H.; Wang Y.; Liang F.; Xu M.; Wang X.-J. Highly Selective Recognition of Monosaccharide Based on Two-component System in Aqueous Solution. Tetrahedron 2011, 67, 3175–3180. 10.1016/j.tet.2011.03.025.

[ref87] Elshocht S. V.; Verbiest T.; Kauranen M.; Ma L.; Cheng H.; Musick K. Y.; Pu L.; Persoons A. Chiral 1,1′-Binaphthyl-Based Helical Polymers as Nonlinear Optical Materials. Chem. Phys. Lett. 1999, 309, 315–320. 10.1016/S0009-2614(99)00730-7.

[ref88] Persoons A.; Kauranen M.; Van Elshocht S.; Verbiest T.; Ma L.; Pu L.; Langeveld-Voss B. M. W.; Meijer E. W. Chiral Effects in Second-Order Nonlinear Optics. Mol. Cryst. Liq. Cryst. 1998, 315, 93–98. 10.1080/10587259808044315.

[ref89] Zhang S.; Sheng Y.; Wei G.; Quan Y.; Cheng Y.; Zhu C. Aggregation-Induced Circularly Polarized Luminescence of an (*R*)-Binaphthyl-based AIE-active Chiral Conjugated Polymer with Self-assembled Helical Nanofibers. Poly. Chem. 2015, 6, 2416–2422. 10.1039/C4PY01689K.

[ref90] Min S. H.; Kim H.-I.; Kim K.-s.; Cha I.; Ha S.; Yun W. S.; Kwak S. K.; Kim J.-H.; Kim B.-S.; Song C. Selective Dispersion of Single-walled Carbon Nanotubes by Binaphthyl-based Conjugated Polymers: Integrated Experimental and Simulation Approach. Polymer 2016, 96, 63–69. 10.1016/j.polymer.2016.04.063.

[ref91] Dong C.; Zhang J.; Zheng W.; Zhang L.; Yu Z.; Choi M. C. K.; Chan A. S. C. Heterogeneous Asymmetric Addition of Diethylzinc to Aromatic Aldehydes Catalyzed by Ti(IV)/Imine Bridged Poly(*R*)-binaphthol. Tetrahedron: Asymmetry 2000, 11, 2449–2454. 10.1016/S0957-4166(00)00190-7.

[ref92] Wei J.; Zhang X.; Zhao Y.; Li R. Chiral Conjugated Microporous Polymers as Novel Chiral Fluorescence Sensors for Amino Alcohols. Chem. Phys. 2013, 214, 2232–2238. 10.1002/macp.201300321.

[ref93] Xu M. H.; Lin J.; Hu Q.-S.; Pu L. Fluorescent Sensors for the Enantioselective Recognition of Mandelic Acid: Signal Amplification by Dendritic Branching. J. Am. Chem. Soc. 2002, 124, 14239–14246. 10.1021/ja020989k. 12440923

[ref94] Yuan C.; Fu S.; Yang K.; Hou B.; Liu Y.; Jiang J.; Cui Y. Crystalline C—C and C=C Bond-Linked Chiral Covalent Organic Frameworks. J. Am. Chem. Soc. 2021, 143, 369–381. 10.1021/jacs.0c11050. 33356183

[ref95] Kobayashi S.; Kusakabe K. -i.; Komiyama S.; Ishitani H. A Switch of Enantiofacial Selectivities Using Designed Similar Chiral Ligands in Zirconium-Catalyzed Asymmetric Aza Diels-Alder Reactions. J. Org. Chem. 1999, 64, 4220–4221. 10.1021/jo9902300.

[ref96] Dolman S. J.; Hultzsch K. C.; Pezet F.; Teng X.; Hoveyda A. H.; Schrock R. R. Supported Chiral Mo-Based Complexes as Efficient Catalysts for Enantioselective Olefin Metathesis. J. Am. Chem. Soc. 2004, 126, 10945–10953. 10.1021/ja047663r. 15339179

[ref97] Liu Y.; Zhang S.; Miao Q.; Zheng L.; Zong L.; Cheng Y. Fluorescent Chemosensory Conjugated Polymers Based on Optically Active Polybinaphthyls and 2,2’-Bipyridyl Units. Macromolecules 2007, 40, 4839–4847. 10.1021/ma0701437.

[ref98] Matsuda A.; Ushimaru T.; Kobayashi Y.; Harada T. Catalytic Enantioselective Arylation and Heteroarylation of Ketones with Organotitanium Reagents Generated In Situ. Chem.—Eur. J. 2017, 23, 8605–8609. 10.1002/chem.201701395. 28497884

[ref99] Yan Z.; Xie G.; Zhang J.; Ma X. Hollow Organic Polymeric Nano-bowls-supported BINOL-derived Chiral Phosphoric Acid: Enhanced Catalytic Performances in the Enantioselective Allylation of Aromatic Aldehydes. Molecular Catalysis 2019, 464, 39–47. 10.1016/j.mcat.2018.12.014.

[ref100] Nimmagadda S. K.; Liu M.; Karunananda M. K.; Gao D.-W.; Apolinar O.; Chen J. S.; Liu P.; Engle K. M. Catalytic, Enantioselective α-Alkylation of Azlactones with Nonconjugated Alkenes by Directed Nucleopalladation. Angew. Chem., Int. Ed. 2019, 58, 3923–3927. 10.1002/anie.201814272. PMC6595491 30729619

[ref101] Wang S.; Zhelavskyi O.; Lee J.; Argüelles A. J.; Khomutnyk Y. Y.; Mensah E.; Guo H.; Hourani R.; Zimmerman P. M.; Nagorny P. Studies of Catalyst-Controlled Regioselective Acetalization and Its Application to Single-Pot Synthesis of Differentially Protected Saccharides. J. Am. Chem. Soc. 2021, 143, 18592–18604. 10.1021/jacs.1c08448. 34705439 PMC8585716

[ref102] Gong L.-Z.; Pu L. The Asymmetric Hetero-Diels-Alder Reaction of Enamide Aldehydes with Danishefsky’s Diene and an Efficient Synthesis of Chiral Binaphthyl Ligands. Tetrahedron Lett. 2000, 41, 2327–2331. 10.1016/S0040-4039(00)00193-3.

[ref103] Huang W.; Pu L. New and Improved Ligands for Highly Enantioselective Catalytic Diphenylzinc Additions to Aryl Aldehydes. Tetrahedron Lett. 2000, 41, 145–149. 10.1016/S0040-4039(99)02041-9.

[ref104] Zhang X.-P.; Wang C.; Wang P.; Du J.-J.; Zhang G.-Q.; Pu L. Conjugated Polymer-Enhanced Enantioselectivity in Fluorescent Sensing. Chem. Sci. 2016, 7, 3614–3620. 10.1039/C6SC00266H. 29997853 PMC6008581

[ref105] Nian S.; Pu L. Amphiphilic Polymer-Based Fluorescent Probe for Enantioselective Recognition of Amino Acids in Immiscible Water and Organic Phases. Chem.—Eur. J. 2017, 23, 18066–18073. 10.1002/chem.201704473. 29069528

[ref106] Nian S.; Pu L. Racemic Fluorescence Probe for Enantiomeric Excess Determination: Application of Cononsolvency of a Polymer in Sensing. J. Org. Chem. 2019, 84, 909–913. 10.1021/acs.joc.8b02793. 30547584

[ref107] Danjo H.; Hamaguchi H.; Asai K.; Nakatani M.; Kawanishi H.; Kawahata M.; Yamaguchi K. Proton-Induced Assembly-Disassembly Modulation of Spiroborate Twin-Bowl Polymers Bearing Pyridyl Groups. Macromolecules 2017, 50, 8028–8032. 10.1021/acs.macromol.7b01924.

[ref108] Takizawa S.; Kodera J.; Yoshida Y.; Sako M.; Breukers S.; Enders D.; Sasai H. Enantioselective Oxidative-Coupling of Polycyclic Phenols. Tetrahedron 2014, 70, 1786–1793. 10.1016/j.tet.2014.01.017.

[ref109] Jayaprakash D.; Sasai H. Synthesis and Catalytic Applications of Soluble Polymer-Supported BINOL. Tetrahedron: Asymmetry 2001, 12, 2589–2595. 10.1016/S0957-4166(01)00443-8.

[ref110] Regnouf de Vains J. B. Selective Mono-Functionalisation at the 6-Position of (*R*)-(+)-2,2′-Diethoxy-1,1′-binaphtalene. Tetrahedron Lett. 2001, 42, 6507–6509. 10.1016/S0040-4039(01)01252-7.

[ref111] Bai X. L.; Liu X. D.; Wang M.; Kang C. Q.; Gao L. X. Synthesis of New Bis-BINOLs Linked by a 2,2’-Bipyridine Bridge. Synthesis 2005, 2005, 458–464. 10.1055/s-2004-837306.

[ref112] Cai D.; Larsen R. D.; Reider P. J. Efficient Synthesis of 6-Mono-bromo-1,1′-bi-2-naphthol. Tetrahedron Lett. 2002, 43, 4055–4057. 10.1016/S0040-4039(02)00730-X.

[ref113] Hocke H.; Uozumi Y. A Simple Synthetic Approach to Homochiral 6- and 6′-Substituted 1,1′-Binaphthyl Derivatives. Tetrahedron 2003, 59, 619–630. 10.1016/S0040-4020(02)01584-3.

[ref114] Recsei C.; McErlean C. S. P. Synthesis of Modified Binol-Phosphoramidites. Tetrahedron 2012, 68, 464–480. 10.1016/j.tet.2011.11.020.

[ref115] Ogunlaja A. S.; Hosten E.; Betz R.; Tshentu Z. R. Selective Removal of Isoquinoline and Quinoline from Simulated Fuel Using 1,1’-Binaphthyl-2,2’-diol (BINOL): Crystal Structure and Evaluation of the Adduct Electronic Properties. RSC Adv. 2016, 6, 39024–39038. 10.1039/C6RA03854A.

[ref116] Yuan X.-Y.; Li H.-Y.; Hodge P.; Kilner M.; Tastard C. Y.; Zhang Z.-P. An Easy Synthesis of Robust Polymer-Supported Chiral 1,1′-Bi-(2-naphthol)s (BINOLs): Application to the Catalysis of the Oxidation of Prochiral Thioethers to Chiral Sulfoxides. Tetrahedron: Asymmetry 2006, 17, 2401–2407. 10.1016/j.tetasy.2006.08.011.

[ref117] Monteiro C. J. P.; Carabineiro S. A. C.; Lauterbach T.; Hubbert C.; Hashmi A. S. K.; Figueiredo J. L.; Pereira M. M. (S)-BINOL Immobilized onto Multiwalled Carbon Nanotubes through Covalent Linkage: A New Approach for Hybrid Nanomaterials Characterization. ChemNanoMat 2015, 1, 178–187. 10.1002/cnma.201500028.

[ref118] Wang Y.; Liu D.; Zhang Y.; Tang Y.; Zhao J.; Shen B. Synthesis of a Novel Chiral Stationary Phase by (R)-1,1′-Binaphthol and the Study on Mechanism of Chiral Recognition. Symmetry 2018, 10, 704 10.3390/sym10120704.

[ref119] Kamei T.; Shibaguchi H.; Sako M.; Toribatake K.; Shimada T. Scandium Triflate-Catalyzed 6,6′-Diiodination of 2,2′-Dimethoxy-1,1′-binaphthyl with 1,3-Diiodo-5,5-dimethylhydantoin. Tetrahedron Lett. 2012, 53, 3894–3896. 10.1016/j.tetlet.2012.05.063.

[ref120] Jiang R.; Ding L.; Zheng C.; You S. L. Iridium-Catalyzed Z-Retentive Asymmetric Allylic Substitution Reactions. Science 2021, 371, 380–386. 10.1126/science.abd6095. 33479149

[ref121] Fehér C.; Urbán B.; Ürge L.; Darvas F.; Bakos J.; Skoda-Földes R. Facile Synthesis of 6-Iodo-2,2′-dipivaloyloxy-1,1′-binaphthyl, A Key Intermediate of High Reactivity for Selective Palladium-Catalyzed Monofunctionalization of the 1,1′-Binaphthalene Core. Tetrahedron Lett. 2010, 51, 3629–3632. 10.1016/j.tetlet.2010.05.022.

[ref122] Fehér C.; Urbán B.; Ürge L.; Darvas F.; Bakos J.; Skoda-Földes R. Palladium-Catalysed Reactions of 6-Halogeno-1,1′-binaphthyl Derivatives. A Detailed Investigation of Structure/Reactivity and Structure/Selectivity Relationships. Tetrahedron 2011, 67, 6327–6333. 10.1016/j.tet.2011.06.011.

[ref123] Hu H.-Y.; Xiang J.-F.; Yang Y.; Chen C.-F. A Helix-Turn-Helix Supersecondary Structure Based on Oligo(phenanthroline dicarboxamide)s. Org. Lett. 2008, 10, 69–72. 10.1021/ol702720q. 18067309

[ref124] Urbano A.; Vallejo S.; Cabrera-Afonso M. J.; Yonte E. Chirality Transfer from the Oxidative Dearomatization of Axially Chiral Binols with Oxone under Mild Conditions. Org. Lett. 2020, 22, 6122–6126. 10.1021/acs.orglett.0c02194. 32648761

[ref125] Chen Y.; Wang X.-z.; Pang J.-y.; Xu Z.-l. Synthesis and Biomimetic Oxidation Reaction of 6-Nitro-1,1′-bis-2-naphthol Derivatives. Acta Sci. Natu. Univ. Sunyatseni 2007, 46, 45–48.

[ref126] Li F.; Wei G.; Sheng Y.; Quan Y.; Cheng Y.; Zhu C. (*S*)-Binaphthalene-Based Fluorescence Polymer Sensors for Direct and Visual F^–^ Detection. Polymer 2014, 55, 5689–5694. 10.1016/j.polymer.2014.09.017.

[ref127] Harada S.; Kuwano S.; Yamaoka Y.; Yamada K. -i.; Takasu K. Kinetic Resolution of Secondary Alcohols Catalyzed by Chiral Phosphoric Acids. Angew. Chem., Int. Ed. 2013, 52, 10227–10230. 10.1002/anie.201304281. 23940027

[ref128] Kuroda Y.; Harada S.; Oonishi A.; Kiyama H.; Yamaoka Y.; Yamada K.; Takasu K. Use of a Catalytic Chiral Leaving Group for Asymmetric Substitutions at sp^3^-Hybridized Carbon Atoms: Kinetic Resolution of β-Amino Alcohols by p-Methoxybenzylation. Angew. Chem., Int. Ed. 2016, 55, 13137–13141. 10.1002/anie.201607208. 27709822

[ref129] Das S.; Liu L.; Zheng Y.; Alachraf M. W.; Thiel W.; Kanta De C.; List B. Nitrated Confined Imidodiphosphates Enable a Catalytic Asymmetric Oxa-Pictet-Spengler Reaction. J. Am. Chem. Soc. 2016, 138, 9429–9432. 10.1021/jacs.6b06626. 27457383

[ref130] Liu L.; Kaib P. S. J.; Tap A.; List B. A General Catalytic Asymmetric Prins Cyclization. J. Am. Chem. Soc. 2016, 138, 10822–10825. 10.1021/jacs.6b07240. 27547839

[ref131] Thoß M.; Seidel R. W.; Feigel M. The Artificial Binaphthyl Amino Acid 6-Amino-6′-carboxyethyl-2-methoxy-2′-hydroxy-1,1′-binaphthyl (Bna): Synthesis and Assembly of Bna Peptides. Tetrahedron 2010, 66, 8503–8511. 10.1016/j.tet.2010.08.066.

[ref132] Leydier A.; Lecerclé D.; Pellet-Rostaing S.; Favre-Reguillon A.; Taran F.; Lemaire M. Sequestering Agent for Uranyl Chelation: New Binaphtyl Ligands. Tetrahedron Lett. 2011, 52, 3973–3977. 10.1016/j.tetlet.2011.05.075.

[ref133] Parvez M. M.; Salam M. A.; Haraguchi N.; Itsuno S. Synthesis of Chiral Ionic Polymers Containing Quaternary Ammonium Sulfonate Structure and Their Catalytic Activity in Asymmetric Alkylation. J. Chin. Chem. Soc. 2012, 59, 815–821. 10.1002/jccs.201100724.

[ref134] Mouhtady O.; Castellan T.; André-Barrès C.; Gornitzka H.; Fabing I.; Saffon-Merceron N.; Génisson Y.; Gaspard H. (*R*)-BINOL-6,6’-bistriflone: Shortened Synthesis, Characterization, and Enantioselective Catalytic Applications. Eur. J. Org. Chem. 2021, 2021, 6674–6681. 10.1002/ejoc.202101137.

[ref135] Turiel M. G.; Garrido-González J. J.; Simón L.; Sanz F.; Lithgow A. M.; Morán J. R.; Fuentes de Arriba Á. L.; Alcázar V. Highly Enantioselective Extraction of Phenylglycine by a Chiral Macrocyclic Receptor Based on Supramolecular Interactions. Org. Lett. 2020, 22, 867–872. 10.1021/acs.orglett.9b04379. 31928015

[ref136] Zhao F.; Wang Y.; Wu X.; Yu S.; Yu X.; Pu L. Sulfonation of 3,3′-Diformyl-BINOL for Enantioselective Fluorescent Recognition of Amino Acids in Water. Chem.—Eur. J. 2020, 26, 7258–7262. 10.1002/chem.202000423. 32128894

[ref137] Zhao F.; Tian J.; Wu X.; Li S.; Chen Y.; Yu S.; Yu X.; Pu L. A near-IR Fluorescent Probe for Enantioselective Recognition of Amino Acids in Aqueous Solution. J. Org. Chem. 2020, 85, 7342–7348. 10.1021/acs.joc.0c00725. 32397707

[ref138] Chen Y.; Zhao F.; Tian J.; Jiang L.; Lu K.; Jiang Y.; Li H.; Yu S.; Yu X.; Pu L. Semiquantitative Visual Chiral Assay with a Pseudoenantiomeric Fluorescent Sensor Pair. J. Org. Chem. 2021, 86, 9603–9609. 10.1021/acs.joc.1c00875. 34165295

[ref139] Wang D.; Carlton C. G.; Tayu M.; McDouall J. J. W.; Perry G. J. P.; Procter D. J. Trifluoromethyl Sulfoxides: Reagents for Metal-Free C-H Trifluoromethylthiolation. Angew. Chem., Int. Ed. 2020, 59, 15918–15922. 10.1002/anie.202005531. PMC7540508 32463942

[ref140] Berger F.; Alvarez E. M.; Frank N.; Bohdan K.; Kondratiuk M.; Torkowski L.; Engl P. S.; Barletta J.; Ritter T. Cine-Substitutions at Five-Membered Hetarenes Enabled by Sulfonium Salts. Org. Lett. 2020, 22, 5671–5674. 10.1021/acs.orglett.0c02067. 32640160 PMC7467811

[ref141] Balaraman E.; Kumara Swamy K. C. A Convenient Chromatography-Free Access to Enantiopure 6,6′-Di-tert-butyl-1,1′-binaphthalene-2,2′-diol and Its 3,3′-Dibromo, Di-tert-butyl and Phosphorus Derivatives: Utility in Asymmetric Aynthesis. Tetrahedron: Asymmetry 2007, 18, 2037–2048. 10.1016/j.tetasy.2007.06.028.

[ref142] Yang J.; Wu S.; Chen F.-X. Chiral Sodium Phosphate Catalyzed Enantioselective 1,4-Addition of TMSCN to Aromatic Enones. Synlett 2010, 2010, 2725–2728. 10.1055/s-0030-1258817.

[ref143] Wang Y.-F.; Zeng W.; Sohail M.; Guo J.; Wu S.; Chen F.-X. Highly Efficient Asymmetric Conjugate Hydrocyanation of Aromatic Enones by an Anionic Chiral Phosphate Catalyst. Eur. J. Org. Chem. 2013, 2013, 4624–4633. 10.1002/ejoc.201300406.

[ref144] Navarro R.; Monterde C.; Iglesias M.; Sánchez F. Readily Available Highly Active [Ti]-Adamantyl-BINOL Catalysts for the Enantioselective Alkylation of Aldehydes. ACS Omega 2018, 3, 1197–1200. 10.1021/acsomega.7b02013. 31457961 PMC6641534

[ref145] Horibe T.; Sakakibara M.; Hiramatsu R.; Takeda K.; Ishihara K. One-Pot Tandem Michael Addition/Enantioselective Conia-Ene Cyclization Mediated by Chiral Iron(III)/Silver(I) Cooperative Catalysis. Angew. Chem., Int. Ed. 2020, 59, 16470–16474. 10.1002/anie.202007180. 32500562

[ref146] Zhou Y.; Zhang D.; Zhu L.; Shuai Z.; Zhu D. Binaphthalene Molecules with Tetrathiafulvalene Units: CD Spectrum Modulation and New Chiral Molecular Switches by Reversible Oxidation and Reduction of Tetrathiafulvalene Units. J. Org. Chem. 2006, 71, 2123–2130. 10.1021/jo052579v. 16497001

[ref147] Maeyama K.; Ogura K.; Okamoto A.; Sakurai K.; Yoshida Y.; Ogino K.; Yonezawa K. P_2_O_5_-MsOH Mediated Regioselective Diaroylation of 2,2′-Dimethoxy-1,1′-binaphthyl. Synth. Commun. 2004, 34, 3243–3250. 10.1081/SCC-200028641.

[ref148] Maeyama K.; Hikiji I.; Ogura K.; Okamoto A.; Ogino K.; Saito H.; Yonezawa N. Synthesis of Optically Active Aromatic Poly(ether ketone)s via Nucleophilic Aromatic Substitution Polymerization. Polymer J. 2005, 37, 707–710. 10.1295/polymj.37.707.

[ref149] Maeyama K.; Tsukamoto T.; Kumagai H.; Higashibayashi S.; Sakurai H. Synthesis of organosoluble and fluorescent aromatic polyketones bearing 1,1′-binaphthyl units through Suzuki-Miyaura coupling polymerization. Polym. Bull. 2015, 72, 2903–2916. 10.1007/s00289-015-1443-z.

[ref150] Periasamy M.; Nagaraju M.; Kishorebabu N. Convenient Method for the Synthesis of 6,6′-Diacyl-1,1′-bi-2-naphthyl Ethers. Synthesis 2007, 2007, 3821–3826. 10.1055/s-2007-990880.

[ref151] Wongma K.; Bunbamrung N.; Thongpanchang T. Synthesis of Bridged Biarylbisquinones and Effects of Biaryl Dihedral Angles on Photo- and Electro-Chemical Properties. Tetrahedron 2016, 72, 1533–1540. 10.1016/j.tet.2016.02.001.

[ref152] Sk M. R.; Bhattacharyya A.; Saha S.; Brahma A.; Maji M. S. Annulative π-Extension by Rhodium(III)-Catalyzed Ketone-Directed C-H Activation: Rapid Access to Pyrenes and Related Polycyclic Aromatic Hydrocarbons (PAHs). Angew. Chem., Int. Ed. 2023, 62, e202305258 10.1002/anie.202305258. 37218605

[ref153] Roy B.; Roy S.; Kundu M.; Maji S.; Pal B.; Mandal M.; Singh N. D. P. Enhanced Electrochemical Water Splitting with Chiral Molecule-Coated Fe_3_O_4_ Nanoparticles. Org. Lett. 2021, 23, 2308–2313. 10.1021/acs.orglett.1c00445. 33689383

[ref154] Bayston D. J.; Fraser J. L.; Ashton M. R.; Baxter A. D.; Polywka M. E. C.; Moses E. Preparation and Use of a Polymer Supported BINAP Hydrogenation Catalyst. J. Org. Chem. 1998, 63, 3137–3140. 10.1021/jo972330g.

[ref155] Kumaraswamy G.; Jena N.; Sastry M. N. V.; Rao G. V.; Ankamma K. Synthesis of 6,6′- and 6-MeO-PEG-BINOL-Ca Soluble Polymer Bound Ligands and Their Application in Asymmetric Michael and Epoxidation Reactions. J. Mol. Catal. A. Chem. 2005, 230, 59–67. 10.1016/j.molcata.2004.12.011.

[ref156] Kumeda K.; Ono M.; Kawai A.; Oike H.; Noguchi K.; Yonezawa N. A Bidirectional Tunnel-like Structure with a Rigid, Thick, and Chiral Aromatic Macrocycle Prepared by Self-complementary 6,6′-Substituted Binaphthyl Monomer. Chem. Lett. 2008, 37, 660–661. 10.1246/cl.2008.660.

[ref157] Yang L.; Yang F.; Lan J.; Gao G.; You J.; Su X. Rational Design of BINOL-Based Diimidazolyl Ligands: Homochiral Channel-Like Mono-Component Organic Frameworks by Hydrogen-Bond-Directed Self-Assembly. Org. Biomol. Chem. 2011, 9, 2618–2621. 10.1039/c1ob00026h. 21403946

[ref158] Kohlhaas M.; Lutz F.; Paransothy N.; Octa-Smolin F.; Wölper C.; Niemeyer J. Synthesis of Bis-BINOL Derivatives: Linking via the 3-, 4-, or 5-Position by Generation of Suitable *C*_1_-Symmetric Precursors. Synthesis 2020, 52, 853–860. 10.1055/s-0039-1690763.

[ref159] Agnes M.; Sorrenti A.; Pasini D.; Wurst K.; Amabilino D. B. Crystal Structure Analyses Facilitate Understanding of Synthesis Protocols in the Preparation of 6,6′-Dibromo-substituted BINOL Compounds. CrystEngComm 2014, 16, 10131–10138. 10.1039/C4CE01160K.

[ref160] Cram D. J.; Helgeson R. C.; Peacock S. C.; Kaplan L. J.; Domeier L. A.; Moreau P.; Koga K.; Mayer J. M.; Chao Y.; Siegel M. G.; Hoffman D. H.; Sogah G. D. Y.; et al. Host-Guest Complexation. 8. Macrocyclic Polyethers Shaped by Two Rigid Substituted Dinaphthyl or Ditetralyl Units. J. Org. Chem. 1978, 43, 1930–1946. 10.1021/jo00404a019.

[ref161] Zhang S.; Liu Y.; Huang H.; Zheng L.; Wu L.; Cheng Y. Direct and Facile Bromination at the 5- and 5′-Positions of (*R*)-6,6′-Dialkyl- and (*R*)-6,6′-Diaryl-2,2′-Binaphthols. Synlett 2008, 2008, 053–856. 10.1055/s-2008-1042902.

[ref162] Wu L.; Zheng L.; Zong L.; Xu J.; Cheng Y. Asymmetric Addition of Phenylacetylene to Aldehydes Catalyzed by Soluble Optically Active Polybinaphthols Ligand. Tetrahedron 2008, 64, 2651–2657. 10.1016/j.tet.2007.12.058.

[ref163] Huang H.; Miao Q.; Kang Y.; Huang X.; Xu J.; Cheng Y. Synthesis and Fluorescent Properties of a Chiral Conjugated Polymer Based on (S)-2,2′-Binaphtho-20-crown-6. Bull. Chem. Soc. Jpn. 2008, 81, 1116–1124. 10.1246/bcsj.81.1116.

[ref164] Iqbal S.; Yu S.; Jiang L.; Wang X.; Chen Y.; Wang Y.; Yu X.; Pu L. Simultaneous Determination of Concentration and Enantiomeric Composition of Amino Acids in Aqueous Solution by Using a Tetrabromobinaphthyl Dialdehyde Probe. Chem.—Eur. J. 2019, 25, 9967–9972. 10.1002/chem.201901374. 31056773

[ref165] Zhang H.-C.; Pu L. Synthesis of Helical Polybinaphthyls. Macromolecules 2004, 37, 2695–2702. 10.1021/ma0354703.

[ref166] Hu L.; Wang Y.; Duan P.; Du Y.; Tian J.; Shi D.; Wang X.; Yu S.; Yu X.; Pu L. Fluorescent Discrimination of Primary Alkyl Amines by Using a Binaphthyl Ladder Polymer. Eur. J. Org. Chem. 2018, 2018, 1896–1901. 10.1002/ejoc.201701774.

[ref167] Hu Q.-S.; Pugh V.; Sabat M.; Pu L. Structurally Rigid and Optically Active Dendrimers. J. Org. Chem. 1999, 64, 7528–7536. 10.1021/jo990856q.

[ref168] Tian Y.; Chan K. S. An Asymmetric Catalytic Carbon-Carbon Bond Formation in a Fluorous Biphasic Ssystem Based on Perfluoroalkyl-BINOL. Tetrahedron Lett. 2000, 41, 8813–8816. 10.1016/S0040-4039(00)01519-7.

[ref169] Pugh V.; Hu Q.-S.; Pu L. The First Dendrimer-Based Enantioselective Fluorescent Sensor for the Recognition of Chiral Amino Alcohols. Angew. Chem., Int. Ed. 2000, 39, 3638–3641. 10.1002/1521-3773(20001016)39:20<3638::AID-ANIE3638>3.0.CO;2-G. 11091423

[ref170] Gong L.-Z.; Hu Q.-S.; Pu L. Optically Active Dendrimers with a Binaphthyl Core and Phenylene Dendrons: Light Harvesting and Enantioselective Fluorescent Sensing. J. Org. Chem. 2001, 66 (7), 2358–2367. 10.1021/jo001565g. 11281776

[ref171] Ma J.; Falkowski J. M.; Abney C.; Lin W. A Series of Isoreticular Chiral Metal-Organic Frameworks as a Tunable Platform for Asymmetric Catalysis. Nature: Chem. 2010, 2, 838–846. 10.1038/nchem.738. 20861899

[ref172] Wanderley M. M.; Wang C.; Wu C.-D.; Lin W. A Chiral Porous Metal-Organic Framework for Highly Sensitive and Enantioselective Fluorescence Sensing of Amino Alcohols. J. Am. Chem. Soc. 2012, 134, 9050–9053. 10.1021/ja302110d. 22607498

[ref173] Li P.; He Y.; Guang J.; Weng L.; Zhao J. C.-G.; Xiang S.; Chen B. A Homochiral Microporous Hydrogen-Bonded Organic Framework for Highly Enantioselective Separation of Secondary Alcohols. J. Am. Chem. Soc. 2014, 136, 547–549. 10.1021/ja4129795. 24392725

[ref174] Wei J.; Zhang X.; Zhao Y.; Li R. Chiral Conjugated Microporous Polymers as Novel Chiral Fluorescence Sensors for Amino Alcohols. Macromol. Chem. Phys. 2013, 214, 2232–2238. 10.1002/macp.201300321.

[ref175] Cui Y.; Evans O. R.; Ngo H. L.; White P. S.; Lin W. Rational Design of Homochiral Solids Based on Two-Dimensional Metal Carboxylates. Angew. Chem., Int. Ed. 2002, 41, 1159–1162. 10.1002/1521-3773(20020402)41:7<1159::AID-ANIE1159>3.0.CO;2-5. 12491246

[ref176] Lee S. J.; Lin W. A Chiral Molecular Square with Metallo-Corners for Enantioselective Sensing. J. Am. Chem. Soc. 2002, 124, 4554–4555. 10.1021/ja0256257. 11971690

[ref177] Lee S. J.; Hu A.; Lin W. The First Chiral Organometallic Triangle for Asymmetric Catalysis. J. Am. Chem. Soc. 2002, 124, 12948–12949. 10.1021/ja028099s. 12405812

[ref178] Valente C.; Choi E.; Belowich M. E.; Doonan C. J.; Li Q.; Gasa T. B.; Botros Y. Y.; Yaghi O. M.; Stoddart F. Metal-Organic Frameworks with Designed Chiral Recognition Sites. Chem. Commun. 2010, 46, 4911–4913. 10.1039/c0cc00997k. 20523946

[ref179] Wu X.; Han X.; Xu Q.; Liu Y.; Yuan C.; Yang S.; Liu Y.; Jiang J.; Cui Y. Chiral BINOL-Based Covalent Organic Frameworks for Enantioselective Sensing. J. Am. Chem. Soc. 2019, 141, 7081–7089. 10.1021/jacs.9b02153. 30971083

[ref180] Liu C.; Jin Y.; Qi D.; Ding X.; Ren H.; Wang H.; Jiang J. Enantioselective Assembly and Recognition of Heterochiral Porous Organic Cages Deduced from Binary Chiral Components. Chem. Sci. 2022, 13, 7014–7020. 10.1039/D2SC01876D. 35774155 PMC9200113

[ref181] Valverde-Gonzalez A.; Borrallo-Aniceto M. C.; Pintado-Sierra M.; Sanchez F.; Arnanz A.; Boronat M.; Iglesias M. BINOL-Containing Chiral Porous Polymers as Platforms for Enantiorecognition. ACS Appl. Mater. Interfaces 2022, 14, 53936–53946. 10.1021/acsami.2c18074. 36417669 PMC10471007

[ref182] Nakazawa H.; Sako M.; Masui Y.; Kurosaki R.; Yamamoto S.; Kamei T.; Shimada T. C-H Triflation of BINOL Derivatives Using DIH and TfOH. Org. Lett. 2019, 21, 6466–6470. 10.1021/acs.orglett.9b02358. 31386383

[ref183] Qin Y. C.; Liu L.; Pu L. One-Step Synthesis of a Bifunctional BINOL Ligand for the Highly Enantioselective Cyanation of Aliphatic Aldehydes. Org. Lett. 2005, 7, 2381–2383. 10.1021/ol050660e. 15932203

[ref184] Qin Y. C.; Liu L.; Sabat M.; Pu L. Synthesis of the Bifunctional BINOL Ligands and Their Applications in the Asymmetric Additions to cCarbonyl Compounds. Tetrahedron 2006, 62, 9335–9348. 10.1016/j.tet.2006.06.049.

[ref185] Truong T.; Daugulis O. Divergent Reaction Pathways for Phenol Arylation by Arynes: Synthesis of Helicenes and 2-Arylphenols. Chem. Sci. 2013, 4, 531–535. 10.1039/C2SC21288A. 24077102 PMC3783345

[ref186] Mahapatra B. B.; Mishra R. R. Polymetallic Complexes of Cobalt(II), Nickel(II), Copper(II), Zinc(II), Cadmium(II) and Mecury(II) with Bis-Bidentate Chelating Azodye Ligand. Ultra Science 2000, 12, 253–255.

[ref187] Mahapatra B. B.; Ray P. Polymetallic complexes.: Part LXXVIII.: Complexes of MnII, CoII, NiII, CuII, ZnII, CdII and HgII with NOON and ONOONO Donor Azodye Ligands. J. Indian Chem. Soc. 2002, 79, 609–610.

[ref188] Urbano A.; Vallejo S.; Cabrera-Afonso M. J.; Yonte E. Chirality Transfer from the Oxidative Dearomatization of Axially Chiral Binols with Oxone under Mild Conditions. Org. Lett. 2020, 22, 6122–6126. 10.1021/acs.orglett.0c02194. 32648761

[ref189] Rueping M.; Sugiono E.; Steck A.; Theissmann T. Synthesis and Application of Polymer-Supported Chiral Brønsted Acid Organocatalysts. Adv. Synth. Catal. 2010, 352, 281–287. 10.1002/adsc.200900746.

[ref190] Svejstrup T. D.; Ruffoni A.; Juliá F.; Aubert V. M.; Leonori D. Synthesis of Arylamines via Aminium Radicals. Angew. Chem., Int. Ed. 2017, 56, 14948–14952. 10.1002/anie.201708693. PMC5698739 28967171

[ref191] Lingenfelter D. S.; Helgeson R. C.; Cram D. J. Host-Guest Complexation. 23. High Chiral Recognition of Amino Acid and Ester Guests by Hosts Containing One Chiral Element. J. Org. Chem. 1981, 46, 393–406. 10.1021/jo00315a033.

[ref192] Wipf P.; Jung J.-K. Formal Total Synthesis of (+)-Diepoxin σ. J. Org. Chem. 2000, 65, 6319–6337. 10.1021/jo000684t. 11052073

[ref193] Klussmann M.; Ratjen L.; Hoffmann S.; Wakchaure V.; Goddard R.; List B. Synthesis of TRIP and Analysis of Phosphate Salt Impurities. Synlett 2010, 2010, 2189–2192. 10.1055/s-0030-1258505.

[ref194] Zimmer R.; Schefzig L.; Peritz A.; Dekaris V.; Reissig H.-U. Functionalized BINOL Derivatives as Ligands for Enantioselectively Catalyzed Aldol Additions: Highly Enantioselective Synthesis of Chiral β-Hydroxy Thioesters. Synthesis 2004, 2004, 1439–1445. 10.1055/s-2004-822364.

[ref193a] Wu P.; Wu Y.; Zhang J.; Lu Z.; Zhang M.; Chen X.; Yuan L. Chromatographic Resolution of α-Amino Acids by (R)-(3,3′-Halogen Substituted-1,1’-binaphthyl)-20-crown-6 Stationary Phase in HPLC. Chin. J. Chem. 2017, 35, 1037–1042. 10.1002/cjoc.201600664.

[ref194a] Ma Q.; Jiang Y.; Lin J.; Zhang X.; Shao H.; Liao S. Organocatalytic Orthogonal ATRP and Ring-Opening Polymerization Using a Single Dual-Function Photocatalyst. Polym. Chem. 2022, 13, 4284–4289. 10.1039/D2PY00633B.

[ref195] Pinxterhuis E. B.; Visser P.; Esser I.; Gualtierotti J.-B.; Feringa B. L. Fast, Efficient and Low E-Factor One-Pot Palladium-Catalyzed Cross-Coupling of (Hetero)Arenes. Angew. Chem., Int. Ed. 2018, 57, 9452–9455. 10.1002/anie.201707760. 29044901

[ref196] Zhang H.; Wirth T. Oxidation of BINOLs by Hypervalent Iodine Reagents: Facile Synthesis of Xanthenes and Lactones. Chem.—Eur. J. 2022, 28, e202200181 10.1002/chem.202200181. 35225370 PMC9311707

[ref197] Ankireddy A. R.; Paidikondala K.; Syed R.; Gundla R.; Reddy Ch. V. R.; Ganapathi T. Synthesis of Chiral 3,3′-Disubstituted (S)-BINOL Derivatives via the Kumada and Suzuki Coupling and Their Antibacterial Activity. Russian J. General Chem. 2020, 90, 1507–1517. 10.1134/S1070363220080198.

[ref198] Meng Y.; Slaven W. T.; Wang D.; Liu T.; Chow H.; Li C. Stepwise Synthesis and Characterization of Oligomers Based on 1,1′-Binaphthol with 3,3′-Acetylene Spacer. Tetrahedron: Asymmetry 1998, 9, 3693–3707. 10.1016/S0957-4166(98)00381-4.

[ref199] Gobbi L.; Seiler P.; Diederich F.; Gramlich V. Molecular Switching: A Fully Reversible, Optically Active Photochemical Switch Based on a Tetraethynylethene-1,1’-BinaphthaleneHybrid System. Helv. Chim. Acta 2000, 83, 1711–1723. 10.1002/1522-2675(20000809)83:8<1711::AID-HLCA1711>3.0.CO;2-#.

[ref200] Gribkov D. V.; Hultzsch K. C.; Hampel F. Synthesis and Characterization of New Biphenolate and Binaphtholate Rare-Earth-Metal Amido Complexes: Catalysts for Asymmetric Olefin Hydroamination/Cyclization. Chem.—Eur. J. 2003, 9, 4796–4810. 10.1002/chem.200304975. 14566888

[ref201] Singh R.; Czekelius C.; Schrock R. R.; Müller R.; Hoveyda A. H. Molybdenum Imido Alkylidene Metathesis Catalysts that Contain Electron Withdrawing Biphenolates or Binaphtholates. Organometallics 2007, 26, 2528–2539. 10.1021/om061134+. 18953421 PMC2572080

[ref202] Bremner J. B.; Coates J. A.; Coghlan D. R.; David D. M.; Keller P. A.; Pyne S. G. The Synthesis of a Novel Binaphthyl-Based Cyclic Peptoid with anti-Bacterial Activity. New J. Chem. 2002, 26, 1549–1551. 10.1039/b205894b.

[ref203] Kickova A.; Donovalova J.; Kasak P.; Putala M. A Chiroptical Binaphthopyran Switch: Amplified CD Response in a Polystyrene Film. New J. Chem. 2010, 34, 1109–1115. 10.1039/c0nj00102c.

[ref204] Zheng M.; Liu Y.; Wang C.; Liu S.; Lin W. Cavity-Induced Enantioselectivity Reversal in a Chiral Metal-Organic Framework Bronsted Acid Catalyst. Chem. Sci. 2012, 3, 2623–2627. 10.1039/c2sc20379k.

[ref205] Jiang Z.; Gao T.; Liu H.; Shaibani M. S. S.; Liu Z. Bronsted Acid-Catalyzed, Highly Enantioselective Addition of Enamides to In Situ-Generated ortho-Quinone Methides: A Domino Approach to Complex Acetamidotetrahydroxanthenes. Chem.—Eur. J. 2015, 21, 2348–2352. 10.1002/chem.201406044. 25488376

[ref206] Jiang Z.; Gao T.; Liu H.; Shaibani M. S. S.; Liu Z. Easily Accessible Axial Chiral Binaphthalene-Triarylborane Dyes Displaying Intense Circularly Polarized Luminescence Both in Solution and in Solid-State. Dye. Pig. 2020, 175, 108168 10.1016/j.dyepig.2019.108168.

[ref207] Simonsen K. B.; Gothelf K. V.; Jørgensen K. A. A Simple Synthetic Approach to 3,3′-Diaryl BINOLs. J. Org. Chem. 1998, 63, 7536–7538. 10.1021/jo980959t. 11672412

[ref208] He Q.; Lin H.; Weng Y.; Zhang B.; Wang Z.; Lei G.; Wang L.; Qiu Y.; Bai F. A Hole-Transporting Material with Controllable Morphology Containing Binaphthyl and Triphenylamine Chromophores. Adv. Funct. Mater. 2006, 16, 1343–1348. 10.1002/adfm.200500541.

[ref209] Lu Q.-S.; Dong L.; Zhang J.; Li J.; Jiang L.; Huang Y.; Qin S.; Hu C.-W.; Yu X.-Q. Imidazolium-Functionalized BINOL as a Multifunctional Receptor for Chromogenic and Chiral Anion Recognition. Org. Lett. 2009, 11, 669–672. 10.1021/ol8027303. 19143512

[ref210] Akiyama T.; Itoh J.; Yokota K.; Fuchibe K. Enantioselective Mannich-Type Reaction Catalyzed by a Chiral Brønsted Acid. Angew. Chem., Int. Ed. 2004, 43, 1566–1568. 10.1002/anie.200353240. 15022235

[ref211] Kratky P.; Haslinger U.; Widhalm M. 2,2′-Dimethoxy-1,1′-binaphthyl-3,3′-diboronic Acid, a Versatile Intermediate in the Synthesis of 3,3′-Diaryl Substituted Binaphthyl Derivatives. Monatshefte für Chemie 1998, 129, 1319–1327. 10.1007/PL00010145.

[ref212] Li J.; Li W.; Li Y.; Li Y.; Yang S. Syntheses of 3- and 3,3′-Substituted 1,1’-bi-2-naphthols. Org. Prep. Proced. Int. 1995, 27, 685–690. 10.1080/00304949509458533.

[ref213] Wang Q.; Chen X.; Tao L.; Wang L.; Xiao D.; Yu X.-Q.; Pu L. Enantioselective Fluorescent Recognition of Amino Alcohols by a Chiral Tetrahydroxyl 1,1’-Binaphthyl Compound. J. Org. Chem. 2007, 72, 97–101. 10.1021/jo061769i. 17194086 PMC2536621

[ref214] Xu K. -x.; Qiu Z.; Zhao J.-J.; Zhao J.; Wang C. -j. Enantioselective Fluorescent Sensors for Amino Acid Derivatives Based on BINOL Bearing Benzoyl Unit. Tetrahedron: Asymmetry 2009, 20, 1690–1696. 10.1016/j.tetasy.2009.06.016.

[ref215] Brunner H.; Schiessling H. Enantioselective Catalysis 89. Acyclic and Macrocyclic Binaphthyl Derivatives. Bull. Soc. Chim. Belg. 1994, 103, 119–126. 10.1002/bscb.19941030307.

[ref216] Stock H. T.; Kellogg R. M. Synthesis of Enantiomerically Pure Thiocrown Ethers Derivedfrom 1,1′-Binaphthalene-2,2′-diol. J. Org. Chem. 1996, 61, 3093–3105. 10.1021/jo952107o. 11667172

[ref217] James T. D.; Sandanayake K. R. A. S.; Shinkai S. Chiral Discrimination of Monosaccharides Using a Fluorescent Molecular Sensor. Nature 1995, 374, 345–347. 10.1038/374345a0.

[ref218] Zhao J.; Fyles T. M.; James T. D. Chiral Binol—Bisboronic Acid as Fluorescence Sensor for Sugar Acids. Angew. Chem. Int. Ed 2004, 43, 3461–3464. 10.1002/anie.200454033. 15221840

[ref219] Li Q.; Guo H.; Wu Y.; Zhang X.; Liu Y.; Zhao J. Enhanced Enantioselective Recognition with Diastereoisomeric BINOL Based Chiral Fluorescent Boronic Acid Sensors. J. Fluores. 2011, 21, 2077–2084. 10.1007/s10895-011-0906-3. 21637998

[ref220] Zhou Y.; Zhang D.; Zhang Y.; Tang Y.; Zhu D. Tuning the CD Spectrum and Optical Rotation Value of a New Binaphthalene Molecule with Two Spiropyran Units: Mimicking the Function of a Molecular “AND” Logic Gate and a New Chiral Molecular Switch. J. Org. Chem. 2005, 70, 6164–6170. 10.1021/jo050489k. 16050673

[ref221] Naruta Y.; Tani F.; Ishihara N.; Maruyama K. Catalytic and Asymmetric Epoxidation of Olefins with Iron Complexes of “Twin-Coronet” Porphyrins. A Mechanistic Insight into the Chiral Induction of Styrene Derivatives. J. Am. Chem. Soc. 1991, 113, 6865–6872. 10.1021/ja00018a024.

[ref222] Dötz K. H.; Tomuschata P.; Niegerh M. Reactions of Complex Ligands, LXXVII. Axial-Chiral Metal Carbenes: Synthesis and Structure of 1,1-Binaphthyl-Derived Carbonyl-Carbene Complexes of Chromium. Chem. Ber. 1997, 130, 1605–1609. 10.1002/cber.19971301108.

[ref223] Tomuschat P.; Kröner L.; Steckhan E.; Nieger M.; Dötz K. H. Reactions of Complex Ligands, Part 86. Benzannulation of Axial Chiral Biscarbene Complexes of Chromium:An Approach to NovelC2-Symmetrical Redox-ActiveBi(phenanthrenequinones). Chem.—Eur. J. 1999, 5, 700–707. 10.1002/(SICI)1521-3765(19990201)5:2<700::AID-CHEM700>3.0.CO;2-D.

[ref224] Zhan X.; Wang S.; Liu Y.; Wu X.; Zhu D. New Series of Blue-Emitting and Electron-Transporting Copolymers Based on Cyanostilbene. Chem. Mater. 2003, 15, 1963–1969. 10.1021/cm020843k.

[ref225] Harada T.; Matsui S. -i.; Tuyet T. M. T.; Hatsuda M.; Ueda S.; Oku A.; Shiro M. Asymmetric Synthesis of Macrocyclic Binaphthol Dimers Using a Sonogashira Coupling Reaction. Tetrahedron: Asymmetry 2003, 14, 3879–3884. 10.1016/j.tetasy.2003.09.038.

[ref226] Will S. G.; Magriotis P.; Marinelli E. R.; Dolan J.; Johnson F. Halocarbon Chemistry. 2. Use of the l,l-Difluoro-2,2-dichloroethyl Group for Phenol Protection. Regulation of Ionization during the Torgov Steroid Synthesis. J. Org. Chem. 1985, 50, 5432–5433. 10.1021/jo00225a101.

[ref227] Thijs M.; Van Goethem C. V.; Vankelecom I. F. J.; Koeckelberghs G. Binaphthalene-Based Polymer Membranes with Enhanced Performance for Solvent-Resistant Nanofiltration. J. Membr. Sci. 2020, 606, 118066 10.1016/j.memsci.2020.118066.

[ref228] Cox P. J.; Wang W.; Snieckus V. Expedient Route to 3- and 3,3′-Substituted 1,1′-Bi-2-naphthols by Directed Ortho Metalation and Suzuki Cross Coupling Methods. Tetrahedron Lett. 1992, 33, 2253–2256. 10.1016/S0040-4039(00)74182-7.

[ref229] Wu T. R.; Shen L.; Chong J. M. Asymmetric Allylboration of Aldehydes and Ketones Using 3,3′-Disubstitutedbinaphthol-Modified Boronates. Org. Lett. 2004, 6, 2701–2704. 10.1021/ol0490882. 15281748

[ref230] Lv N.; Xie M.; Gu W.; Ruan H.; Qiu S.; Zhou C.; Cui Z. Synthesis, Properties, and Structures of Functionalized peri-Xanthenoxanthene. Org. Lett. 2013, 15, 2382–2385. 10.1021/ol400790d. 23659211

[ref231] Ooi T.; Kameda M.; Maruoka K. Design of N-Spiro *C*_2_-Symmetric Chiral Quaternary Ammonium Bromides as Novel Chiral Phase-Transfer Catalysts: Synthesis and Application to Practical Asymmetric Synthesis of r-Amino Acids. J. Am. Chem. Soc. 2003, 125, 5139–5151. 10.1021/ja021244h. 12708866

[ref232] Hermeke J.; Mewald M.; Irran E.; Oestreich M. Chemoselective Tin-Boron Exchange Aided by the Use of Dummy Ligands at the Tin Atom. Organometallics 2014, 33, 5097–5100. 10.1021/om500851r.

[ref233] Xu Y.; Clarkson G. C.; Docherty G.; North C. L.; Woodward G.; Wills M. Ruthenium(II) Complexes of Monodonor Ligands: Efficient Reagents for Asymmetric Ketone Hydrogenation. J. Org. Chem. 2005, 70, 8079–8087. 10.1021/jo051176s. 16277330

[ref234] Hu Q.-S.; Huang W.-S.; Vitharana D.; Zheng X.-F.; Pu L. Functionalized Major-Groove and Minor-Groove Chiral Polybinaphthyls: Application in the Asymmetric Reaction of Aldehydes with Diethylzinc. J. Am. Chem. Soc. 1997, 119, 12454–12464. 10.1021/ja972623r.

[ref235] Hu Q.-S.; Huang W.-S.; Pu L. A New Approach to Highly Enantioselective Polymeric Chiral Catalysts. J. Org. Chem. 1998, 63, 2798–2799. 10.1021/jo980004a.

[ref236] Huang W.-S.; Hu Q.-S.; Pu L. A Highly General Catalyst for the Enantioselective Reaction of Aldehydes with Diethylzinc. J. Org. Chem. 1998, 63, 1364–1365. 10.1021/jo971985e.

[ref237] Yu H.-B.; Zheng X.-F.; Lin Z.-M.; Hu Q.-S.; Huang W.-S.; Pu L. Asymmetric Epoxidation of α,β-Unsaturated Ketones Catalyzed by Chiral Polybinaphthyl Zinc Complexes: Greatly Enhanced Enantioselectivity by a Cooperation of the Catalytic Sites in a Polymer Chain. J. Org. Chem. 1999, 64, 8149–8155. 10.1021/jo990749w. 11674730

[ref238] Huang W.-S.; Hu Q.-S.; Pu L. From Highly Enantioselective Monomeric Catalysts to Highly Enantioselective Polymeric Catalysts: Application of Rigid and Sterically Regular Chiral Binaphthyl Polymers to the Asymmetric Synthesis of Chiral Secondary Alcohols. J. Org. Chem. 1999, 64, 7940–7956. 10.1021/jo990992v.

[ref239] Milburn R. R.; Hussain S. M. S.; Prien O.; Ahmed Z.; Snieckus V. 3,3′-Dipyridyl BINOL Ligands. Synthesis and Application in Enantioselective Addition of Et_2_Zn to Aldehydes. Org. Lett. 2007, 9, 4403–4406. 10.1021/ol071276f. 17900193

[ref240] Sälinger D.; Brückner Rr. The First Asymmetric Halogen/Metal-Exchange Reaction: Desymmetrization of Alcohols with Enantiotopic Bromoarene Substituents. Chem.—Eur. J. 2009, 15, 6688–6703. 10.1002/chem.200802488. 19492368

[ref241] Goldys A.; McErlean C. S. P. An Efficient Synthesis of 3,3′-Dipyridyl BINOL Ligands. Tetrahedron Lett. 2009, 50, 3985–2987. 10.1016/j.tetlet.2009.04.092.

[ref242] Le P. Q.; Nguyen T. S.; May J. A. A General Method for the Enantioselective Synthesis of α-Chiral Heterocycles. Org. Lett. 2012, 14, 6104–6107. 10.1021/ol3030605. 23157440

[ref243] Lutzen A.; Thiemann F.; Meyer S. Synthesis of Tetra(BINOL) Substituted Spirobifluorenes. Synthesis 2002, 18, 2771–2777. 10.1055/s-2002-35997.

[ref244] Wen W.; Luo M.-J.; Yuan Y.; Liu J.-H.; Wu Z.-L.; Cai T.; Wu Z.-W.; Ouyang Q.; Guo Q.-X. Diastereodivergent Chiral Aldehyde Catalysis for Asymmetric 1,6-Conjugated Addition and Mannich Reactions. Nature Commun. 2020, 11, 5372 10.1038/s41467-020-19245-3. 33097724 PMC7584650

[ref245] La Londe R. L.; Wang Z. J.; Mba M.; Lackner A. D.; Toste F. D. Gold(I)-Catalyzed Enantioselective Synthesis of Pyrazolidines, Isoxazolidines, and Tetrahydrooxazines. Angew. Chem., Int. Ed. 2010, 49, 598–601. 10.1002/anie.200905000. PMC2995290 20014376

[ref246] Guetz C.; Hovorka R.; Schnakenburg G.; Luetzen A. Homochiral Supramolecular M_2_L_4_ Cages by High-Fidelity Self-Sorting of Chiral Ligands. Chem.—Eur. J. 2013, 19, 10890–10894. 10.1002/chem.201301499. 23824836

[ref247] Li F.; Wang Y.; Sheng Y.; Wei G.; Cheng Y.; Zhu C. CPL Emission of Chiral BINOL-Based Polymers via Chiral Transfer of the Conjugated Chain Backbone Structure. RSC Adv. 2015, 5, 105851–105854. 10.1039/C5RA23329A.

[ref248] Romanov-Michailidis F.; Guenee L.; Alexakis A. Enantioselective Organocatalytic Fluorination-Induced Wagner-Meerwein Rearrangement. Angew. Chem., Int. Ed. 2013, 52, 9266–9270. 10.1002/anie.201303527. 23852804

[ref249] Meng F.; Li Y.; Zhang W.; Li S.; Quan Y.; Cheng Y. Circularly Polarized Luminescence Based Chirality Transfer of the Chiral BINOL Moiety via Rigid π-Conjugation Chain Backbone Structures. Polymer Chem. 2017, 8, 1555–1561. 10.1039/C6PY02218A.

[ref250] Zhang Y.; Zhang Z.; Ma S.; Jia J.; Xia H.; Liu X. Hypercrosslinking Chiral Bronsted Acids into Porous Organic Polymers for Efficient Heterogeneous Asymmetric Organosynthesis. J. Mater. Chem. A 2021, 9, 25369–25373. 10.1039/D1TA07449K.

[ref251] Yonesaki R.; Kondo Y.; Akkad W.; Sawa M.; Morisaki K.; Morimoto H.; Ohshima T. 3-Mono-Substituted BINOL Phosphoric Acids as Effective Organocatalysts in Direct Enantioselective Friedel-Crafts-Type Alkylation of N-Unprotected α-Ketiminoester. Chem.—Eur. J. 2018, 24, 15211–15214. 10.1002/chem.201804078. 30098059

[ref252] Guo Q.-S.; Liu B.; Lu Y. N.; Jiang F.-Y.; Song H.-B.; Li J. S. Synthesis of 3 or 3,3′-Substituted BINOL Ligands and Their Application in the Asymmetric Addition of Diethylzinc to Aromatic Aldehydes. Tetrahedron: Asymmetry 2005, 16, 3667–3671. 10.1016/j.tetasy.2005.09.018.

[ref253] Lu Y.-N.; Guo Q.-S.; Jiang F.-Y.; Li J.-S. Synthesis of Modified H_4_-BINOL Ligands and Their Applications in the Asymmetric Addition of Diethylzinc to Aromatic Aldehydes. Tetrahedron: Asymmetry 2006, 17, 1842–1845. 10.1016/j.tetasy.2006.06.042.

[ref254] Zhang Z.-G.; Dong Z.-B.; Li J.-S. Synthesis and Application of 3-Substituted (S)-BINOL as Chiral Ligands for the Asymmetric Ethylation of Aldehydes. Chirality 2010, 22, 820–826. 10.1002/chir.20842. 20803746

[ref255] Yu H.-B.; Hu Q.-S.; Pu L. The First Optically Active BINOL- BINAP Copolymer Catalyst: Highly Stereoselective Tandem Asymmetric Reactions. J. Am. Chem. Soc. 2000, 122, 6500–6501. 10.1021/ja000778k.

[ref256] Jin R.-Z.; Bian Z.; Kang C. -q.; Guo H. -q.; Gao L. -x. Synthesis of 3,30-Di(2-Pyridyl)-1,10-Bi-2-Naphthol Derivatives. Synth. Commun. 2005, 35, 1897–1902. 10.1081/SCC-200064925.

[ref257] Ma L.; Jin R.-Z.; Lu G.-H.; Bian Z.; Ding M.-X.; Gao L.-X. Synthesis and Applications of 3-[6-(Hydroxymethyl)pyridin-2-yl]-1,1’-bi-2-naphthols or 3,3′-Bis[6-(hydroxymethyl)pyridin-2-yl]-1,1’-bi-2-naphthols. Synthesis 2007, 2007, 2461–2470. 10.1055/s-2007-983787.

[ref258] He Y.; Bian Z.; Kang C.; Gao L. Stereoselective and Hierarchical Self-Assembly from Nanotubular Homochiral Helical Coordination Polymers to Supramolecular Gels. Chem. Commun. 2010, 46, 5695–5697. 10.1039/c0cc01433h. 20596555

[ref259] Xu Y.; Yu S.; Chen Q.; Chen X.; Li Y.; Yu X.; Pu L. Fluorescent Recognition of 1,2-Diamines by a 1,1’-Binaphthyl-Based Trifluoromethyl Ketone. Chem.—Eur. J. 2016, 22, 12061–12067. 10.1002/chem.201601540. 27415468

[ref260] Liang J.; Zhi X.; Zhou Q.; Yang J. Binaphthol-Derived Phosphoric Acids as Efficient Organocatalysts for the Controlled Ring-Opening Polymerization of γ-Benzyl-L-glutamate N-carboxyanhydrides. Polymer 2019, 165, 83–90. 10.1016/j.polymer.2019.01.027.

[ref261] Diaz-Oviedo C. D.; Maji R.; List B. The Catalytic Asymmetric Intermolecular Prins Reaction. J. Am. Chem. Soc. 2021, 143, 20598–20604. 10.1021/jacs.1c10245. 34846874 PMC8679094

[ref262] Grossmann O.; Maji R.; Aukland M. H.; Lee S.; List B. Catalytic Asymmetric Additions of Enol Silanes to In Situ Generated Cyclic, Aliphatic N-Acyliminium Ions. Angew. Chem., Int. Ed. 2022, 61, e202115036 10.1002/anie.202115036. PMC9303265 34897932

[ref263] Chen H.; Peng J.; Pang Q.; Du H.; Huang L.; Gao L.; Lan Y.; Yang C.; Song Z. Enantioselective Synthesis of Spirosilabicyclohexenes by Asymmetric Dual Ring Expansion of Spirosilabicyclobutane with Alkynes. Angew. Chem., Int. Ed. 2022, 61, e202212889 10.1002/anie.202212889. 36203376

[ref264] Yue Y.; Turlington M.; Yu X.-Q.; Pu L. 3,3′-Anisyl-Substituted BINOL, H_4_BINOL, and H_8_BINOL Ligands: Asymmetric Synthesis of Diverse Propargylic Alcohols and Their Ring-Closing Metathesis to Chiral Cycloalkenes. J. Org. Chem. 2009, 74, 8681–868. 10.1021/jo9018446. 19860396

[ref265] Goldys A.; McErlean C. S. P. An Efficient Synthesis of 3,3′-Dipyridyl BINOL Ligands. Tetrahedron Lett. 2009, 50, 3985–3987. 10.1016/j.tetlet.2009.04.092.

[ref266] Gatzenmeier T.; Turberg M.; Yepes D.; Xie Y.; Neese F.; Bistoni G.; List B. Scalable and Highly Diastereo- and Enantioselective Catalytic Diels-Alder Reaction of α,β-Unsaturated Methyl Esters. J. Am. Chem. Soc. 2018, 140, 12671–12676. 10.1021/jacs.8b07092. 30277760

[ref267] Fernández-Ibáñez M. Á.; Maciá B.; Minnaard A. J.; Feringa B. L. Catalytic Enantioselective Reformatsky Reaction with ortho-Substituted Diarylketones. Org. Lett. 2008, 10, 4041–4044. 10.1021/ol801574m. 18715009

[ref800] Storer R. I.; Carrera D. E.; Ni Y.; MacMillan D. W. C. Enantioselective Organocatalytic Reductive Amination. J. Am. Chem. Soc. 2006, 128, 84–86. 10.1021/ja057222n. 16390133

[ref801] Akiyama T.; Suzuki T.; Mori K. Enantioselective Aza-Darzens Reaction Catalyzed by A Chiral Phosphoric Acid. Org. Lett. 2009, 11, 2445–2447. 10.1021/ol900674t. 19397337

[ref270] Bedford R. B.; Chang Y.-N.; Haddow M. F.; McMullin C. L. Tuning Ligand Structure in Chiral Bis(phosphite) and Mixed Phosphite-Phosphinite PCP-Palladium Pincer Complexes. Dalton Trans. 2011, 40, 9034–9041. 10.1039/c1dt10356c. 21818490

[ref271] Hashimoto T.; Naganawa Y.; Maruoka K. Desymmetrizing Asymmetric Ring Expansion of Cyclohexanones with α-Diazoacetates Catalyzed by Chiral Aluminum Lewis Acid. J. Am. Chem. Soc. 2011, 133, 8834–8837. 10.1021/ja202070j. 21553888

[ref272] Simonin C.; Awale M.; Brand M.; van Deursen R.; Schwartz J.; Fine M.; Kovacs G.; Haefliger P.; Gyimesi G.; Sithampari A.; Charles R.-P.; Hediger M. A.; Reymond J.-L. Optimization of TRPV6 Calcium Channel Inhibitors Using a 3D Ligand-Based Virtual Screening Method. Angew. Chem., Int. Ed. 2015, 54, 14748–14752. 10.1002/anie.201507320. 26457814

[ref273] Morisaki K.; Morimoto H.; Ohshima T. Direct Access to N-Unprotected Tetrasubstituted Propargylamines via Direct Catalytic Alkynylation of N-Unprotected Trifluoromethyl Ketimines. Chem. Commun. 2017, 53, 6319–6322. 10.1039/C7CC02194A. 28447089

[ref274] See J. Y.; Yang H.; Zhao Y.; Wong M. W.; Ke Z.; Yeung Y.-Y. Desymmetrizing Enantio- and Diastereoselective Selenoetherification through Supramolecular Catalysis. ACS Catal. 2018, 8, 850–858. 10.1021/acscatal.7b03510.

[ref275] Wang P.; Ma G.-R.; Yu S.-L.; Da C.-S. Enantioselective vinylation of aldehydes with the vinyl Grignard reagent catalyzed by magnesium complex of chiral BINOLs. Chirality 2019, 31, 79–86. 10.1002/chir.23038. 30520127

[ref276] Börner C.; Dennis M. R.; Sinn E.; Woodward S. Copper-Catalysed Asymmetric 1,4-Addition of Organozinc Compounds to Linear Aliphatic Enones Using 2,2′-Dihydroxy 3,3′-Dithioether Derivatives of 1,1′-Binaphthalene. Eur. J. Org. Chem. 2001, 2001, 2435–2446. 10.1002/1099-0690(200107)2001:13<2435::AID-EJOC2435>3.0.CO;2-0.

[ref277] Aggarwal V. K.; Coogan M. P.; Stenson R. A.; Jones R. V. H.; Fieldhouse R.; Blacker J. Developments in the Simmons-Smith-Mediated Epoxidation Reaction. Eur. J. Org. Chem. 2002, 2002, 319–326. 10.1002/1099-0690(20021)2002:2<319::AID-EJOC319>3.0.CO;2-R.

[ref278] Selig P. S.; Miller S. J. ortho-Acidic Aromatic Thiols as Efficient Catalysts of Intramolecular Morita-Baylis-Hillman and Rauhut-Currier Reactions. Tetrahedron Lett. 2011, 52, 2148–2151. 10.1016/j.tetlet.2010.11.077.

[ref279] Beckendorf S.; Mancheño G. Click”-BINOLs: A New Class of Tunable Ligands for Asymmetric Catalysis. Synthesis 2012, 44, 2162–2172. 10.1055/s-0031-1291009.

[ref280] Zhang W.; Zhang X. Synthesis of Triphosphorous Bidentate Phosphine-Phosphoramidite Ligands: Application in the Highly Enantioselective Hydrogenation of ortho-Substituted Aryl Enamides. Angew. Chem., Int. Ed. 2006, 45, 5515–5518. 10.1002/anie.200601501. 16892468

[ref281] Guo Q.-S.; Lu Y. N.; Liu B.; Xiao J.; Li J.-S. A Facile Synthesis of 3 or 3,3′-Substituted Binaphthols and Their Applications in the Asymmetric Addition of Diethylzinc to Aldehydes. J. Organomet. Chem. 2006, 691, 1282–1287. 10.1016/j.jorganchem.2005.12.042.

[ref282] Qian C.; Huang T.; Zhu C.; Sun J. Synthesis of 3,3′-, 6,6′- and 3,3′,6,6′-Substituted Binaphthols and Their Application in the Asymmetric Hydrophosphonylation of Aldehydes: An Obvious Effect of Substituents of BINOL on the Enantioselectivity. J. Chem. Soc., Perkin Trans. 1. 1998, 2097–2103. 10.1039/a800146d.

[ref283] Yoshikawa N.; Shibasaki M. Direct Catalytic Asymmetric Aldol Reactions Promoted by Novel Heterobimetallic Catalysts Possessing Strong Bronsted Base: A New Strategy for the Development of Lewis Acid-Bronsted Base Bifunctional Catalysts. Tetrahedron 2001, 57, 2569–2579. 10.1016/S0040-4020(01)00070-9.

[ref284] Kang J.; Lee J. H.; Lim D. S. Stereoselective Conjugate Addition of Diethylzinc to Enones and Nitroalkenes. Tetrahedron: Asymmetry 2003, 14, 305–315. 10.1016/S0957-4166(02)00838-8.

[ref285] Arai K.; Salter M. M.; Yamashita Y.; Kobayashi S. Enantioselective Desymmetrization of meso Epoxides with Anilines Catalyzed by a Niobium Complex of a Chiral Multidentate Binol Derivative. Angew. Chem., Int. Ed. 2007, 46, 955–957. 10.1002/anie.200603787. 17173328

[ref286] Arai K.; Lucarini S.; Salter M. M.; Ohta K.; Yamashita Y.; Kobayashi S. The Development of Scalemic Multidentate Niobium Complexes as Catalysts for the Highly Stereoselective Ring Opening of meso-Epoxides and meso-Aziridines. J. Am. Chem. Soc. 2007, 129, 8103–8111. 10.1021/ja0708666. 17567008

[ref287] Kobayashi S.; Arai K.; Shimizu H.; Ihori Y.; Ishitani H.; Yamashita Y. A Novel Dinuclear Chiral Niobium Complex for Lewis Acid Catalyzed Enantioselective Reactions: Design of a Tridentate Ligand and Elucidation of the Catalyst Structure. Angew. Chem., Int. Ed. 2005, 44, 761–764. 10.1002/anie.200462204. 15612063

[ref288] Ishitani H.; Kitazawa T.; Kobayashi S. Efficient Catalytic Enantioselective Mannich-Type Reactions Using a Zirconium-Bis(binaphthol)methane Complex. Tetrahedron Lett. 1999, 40, 2161–2164. 10.1016/S0040-4039(99)00138-0.

[ref289] Zhang Y.-L.; Zhang F.; Tang W.-J.; Wu Q.-L.; Fan Q.-H. Synthesis and Application of 3,3′-Diarylmethyl BINOLs. Synlett 2006, 2006, 1250–1254. 10.1055/s-2006-932475.

[ref290] Zhao G.; Wang T. Stereoselective Synthesis of 2-Deoxyglycosides from Glycals by Visible-Light-Induced Photoacid Catalysis. Angew. Chem., Int. Ed. 2018, 57, 6120–6124. 10.1002/anie.201800909. 29569307

[ref291] Yu S.; Plunkett W.; Kim M.; Pu L. Simultaneous Determination of Both the Enantiomeric Composition and Concentration of a Chiral Substrate with One Fluorescent Sensor. J. Am. Chem. Soc. 2012, 134, 20282–20285. 10.1021/ja3101165. 23214478

[ref292] Yu S.; Plunkett W.; Kim M.; Wu E.; Pu L. 1,1’-Bi-2-naphthol-Fluoroacetyl Compounds in Fluorescent Recognition of Amines. Org. Chem. Front. 2014, 1, 395–404. 10.1039/C3QO00076A.

[ref293] Wang C.; Wu E.; Wu X.; Xu X.; Zhang G.; Pu L. Enantioselective Fluorescent Recognition in the Fluorous Phase: Enhanced Reactivity and Expanded Chiral Recognition. J. Am. Chem. Soc. 2015, 137, 3747–3750. 10.1021/ja512569m. 25761050

[ref294] Wang C.; Zeng C.; Zhang X.; Pu L. Enantioselective Fluorescent Recognition of Amino Acids by Amide Formation: An Unusual Concentration Effect. J. Org. Chem. 2017, 82, 12669–12673. 10.1021/acs.joc.7b02456. 29096058

[ref295] Zhang H.-C.; Huang W.-S.; Pu L. Biaryl-Based Macrocyclic and Polymeric Chiral (Salophen)Ni(II)Complexes: Synthesis and Spectroscopic Study. J. Org. Chem. 2001, 66, 481–487. 10.1021/jo001276s. 11429818

[ref296] Brunner H.; Goldbrunner J. Asymmetric Catalysis 49. Optically Active Binaphthyl Derivatives - Synthesis and Use in Transition-Metal Catalysts. Chem. Ber. 1989, 122, 2005–2009. 10.1002/cber.19891221028.

[ref297] Brunner H.; Schiessling H. Dialdehyde + Diamine Polymer or Macrocycle?. Angew. Chem., Int. Ed. Engl. 1994, 33, 125–126. 10.1002/anie.199401251.

[ref298] Li Z. B.; Lin J.; Pu L. A Cyclohexyl-1,2-diamine-Derived Bis(binaphthyl) Macrocycle: Enhanced Sensitivity and Enantioselectivity in the Fluorescent Recognition of Mandelic Acid. Angew. Chem., Int. Ed. 2005, 44, 1690–1693. 10.1002/anie.200462471. 15688352

[ref299] Xu X.; Trindle C. O.; Zhang G.; Pu L. Fluorescent Recognition of Hg^2+^ by a 1,1’-Binaphthyl-Based Macrocycle: A Highly Selective Off-on-off Response. Chem. Commun. 2015, 51, 8469–8472. 10.1039/C5CC02457A. 25891178

[ref300] Huang Z.; Yu S.; Wen K.; Yu X.; Pu L. Zn(II) Promoted Dramatic Enhancement in the Enantioselective Fluorescent Recognition of Chiral Amines by a Chiral Aldehyde. Chem. Sci. 2014, 5, 3457–3462. 10.1039/C4SC00818A.

[ref301] Wen K.-L.; Yu S.; Huang Z.; Chen L.; Xiao M.; Yu X.; Pu L. Rational Design of a Fluorescent Sensor to Simultaneously Determine Both the Enantiomeric Composition and the Concentration of Chiral Functional Amines. J. Am. Chem. Soc. 2015, 137 (13), 4517–4524. 10.1021/jacs.5b01049. 25790271

[ref302] Mao Y.; Thomae E.; Davis S.; Wang C.; Li Y.; Pu L. One Molecular Probe with Opposite Enantioselective Fluorescence Enhancement at Two Distinct Emissions. Org. Lett. 2023, 25 (12), 2157–2161. 10.1021/acs.orglett.3c00692. 36940095

[ref303] Liu H.-L.; Hou X.-L.; Pu L. Enantioselective Precipitation and Solid-State Fluorescence Enhancement in the Recognition of α-Hydroxycarboxylic Acids. Angew. Chem., Int. Ed. 2009, 48, 382–385. 10.1002/anie.200804538. 19065550

[ref304] Liu H.-L.; Peng Q.; Wu Y.-D.; Chen D.; Hou X.-L.; Sabat M.; Pu L. Highly Enantioselective Recognition of Structurally Diverse α-Hydroxycarboxylic Acids using a Fluorescent Sensor. Angew. Chem., Int. Ed. 2010, 49, 602–606. 10.1002/anie.200904889. 20014266

[ref305] Yu S.; Pu L. Pseudoenantiomeric Fluorescent Sensors in a Chiral Assay. J. Am. Chem. Soc. 2010, 132, 17698–17700. 10.1021/ja1086408. 21121601

[ref306] Zeng C.; Zhang X.; Pu L. Enhanced Enantioselectivity in the Fluorescent Recognition of a Chiral Diamine by Using a Bisbinaphthyl Dialdehyde. ACS Omega 2018, 3, 12545–12548. 10.1021/acsomega.8b01870. 31457988 PMC6644887

[ref307] Zhu Y.-Y.; Wu X.-D.; Gu S.-X.; Pu L. Free Amino Acid Recognition: A Bisbinaphthyl-Based Fluorescent Probe with High Enantioselectivity. J. Am. Chem. Soc. 2019, 141, 175–181. 10.1021/jacs.8b07803. 30525565

[ref308] Mao Y.; Abed M. A.; Lee N. B.; Wu X.; Du G.; Pu L. Determining the Concentration and Enantiomeric Composition of Histidine Using One Fluorescent Probe. Chem. Commun. 2021, 57, 587–590. 10.1039/D0CC07498E. 33345262

[ref309] Mao Y.; Davis S.; Pu L. Regio-and Enantioselective Macrocyclization from Dynamic Imine Formation: Chemo-and Enantioselective Fluorescent Recognition of Lysine. Org. Lett. 2023, 25, 7639–7644. 10.1021/acs.orglett.3c02949. 37843813

[ref310] Lu K.; Guo H.; Jiang Y.; Yang J.; Yu S.; Yu X.; Pu L. Synthesis of a BINOL-Based *C*_3_ Symmetric Schiff Base and Its Fluorescence Response to Zn^2+^. ChemPlusChem 2023, 88, e202300036 10.1002/cplu.202300036. 36800303

[ref311] Kitajima H.; Aoki Y.; Ito K.; Katsuki T. Asymmetric Simmons-Smith Cyclopropanation of *E*-Allylic Alcohols Using 1,1′-Bi-2-naphthol-3,3′-dicarboxamide as a Chiral Auxiliary. Chem. Lett. 1995, 24, 1113–1114. 10.1246/cl.1995.1113.

[ref312] Kitajima H.; Ito K.; Aoki Y.; Katsuki T. N,N,N’,N’-Tetraalkyl-2,2’-dihydroxy-1,1’-binaphthyl-3,3′-dicarboxamides: Novel Chiral Auxiliaries for Asymmetric Simmons-Smith Cyclopropanation of Allylic Alcohols and for Asymmetric Diethylzinc Addition to Aldehydes. Bull. Chem. Soc. Jpn. 1997, 70, 207–217. 10.1246/bcsj.70.207.

[ref313] Kodama H.; Ito J.; Hori K.; Ohta T.; Furukawa I. Lanthanide-Catalyzed Asymmetric 1,3-Dipolar Cycloaddition of Nitrones to Alkenes Using 3,3′-Bis(2-oxazolyl)-1,1′-bi-2-naphthol (BINOL-Box) Ligands. J. Organomet. Chem. 2000, 603, 6–12. 10.1016/S0022-328X(00)00024-3.

[ref314] Liu G.-H.; Fan Q.-H.; Yang X.-Q.; Chen X. M. Chiral BINOL-centered dendrimers for the enantioselective Lewis acid catalyzed diethylzinc addition to aldehydes. Arkivoc 2003, 2003, 123–132. 10.3998/ark.5550190.0004.214.

[ref315] Chen H.; Zhang H.; Du H.; Kuang Y.; Pang Q.; Gao L.; Wang W.; Yang C.; Song Z. Enantioselective Synthesis of 6/5-Spirosilafluorenes by Asymmetric Ring Expansion of 4/5-Spirosilafluorenes with Alkynes. Org. Lett. 2023, 25, 1558–1563. 10.1021/acs.orglett.3c00346. 36847236

[ref316] Shevchenko G. A.; Pupo G.; List B. Direct Asymmetric α-Hydroxylation of Cyclic α-Branched Ketones through Enol Catalysis. Synlett 2019, 30, 49–53. 10.1055/s-0037-1611084.

[ref317] Song J.; Wang M.; Xu X.; Qu L.; Zhou X.; Xiang H. 1D-Helical Platinum(II) Complexes Bearing Metal Induced Chirality, Aggregation-Induced Red Phosphorescence, and Circularly Polarized Luminescence. Dalton Trans. 2019, 48, 4420–4428. 10.1039/C8DT03615B. 30865737

[ref318] Brisdon B. J.; England R.; Reza K.; Sainsbury M. Monofunctional Chiral Crowns 1. Tetrahedron 1993, 49, 1103–1114. 10.1016/S0040-4020(01)86291-8.

[ref319] Dennis M. R.; Woodward S. Enantiopure 3-amido or 3,3′-Bisamido Substituted 1,1′-Bi-2-naphthols by Anionic Fries Rearrangements. J. Chem. Soc. Perkin Trans. 1. 1998, 1081–1085. 10.1039/a708436f.

[ref320] Blackmore I. J.; Boa A. N.; Murray E. J.; Dennis M.; Woodward S. A Simple Preparation of Iodoarenes, Iodoalkenes and Iodoalkynes by Reaction of Organolithiums with 2,2,2-Trifluoro-1-iodoethane. Tetrahedron Lett. 1999, 40, 6671–6672. 10.1016/S0040-4039(99)01343-X.

[ref321] North M.; Villuendas P.; Williamson C. Mechanistic Comparison of Aluminium Based Catalysts for Asymmetric Cyanohydrin Synthesis. Tetrahedron 2010, 66, 1915–1924. 10.1016/j.tet.2010.01.004.

[ref322] Kauch M.; Snieckus V.; Hoppe D. Substitution of Hydroxybiaryls via Directed ortho-Lithiation of N-Silylated O-Aryl N-Isopropylcarbamates. J. Org. Chem. 2005, 70, 7149–7158. 10.1021/jo0506938. 16122233

[ref323] Au-Yeung T.-L.; Chan K.-Y.; Chan W.-K.; Haynes R. K.; Williams I. D.; Yeung L. L. Reactions of (RP)- and (SP)-tert-Butylphenylphosphinobromidates and tert-Butylphenylthionophosphinochloridates with Heteroatom Nucleophiles; Preparation of P-chiral Binol Phosphinates and Related Compounds. Tetrahedron Lett. 2001, 42, 453–456. 10.1016/S0040-4039(00)01950-X.

[ref324] Au-Yeung T.- L.; Chan K.-Y.; Haynes R. K.; Williams I. D.; Yeung L. L. Completely Stereoselective P-C Bond Formation via Base-Induced [1,3]- and [1,2]-Intramolecular Rearrangements of Aryl Phosphinates, Phosphinoamidates and Related Compounds: Generation of *P*-Chiral β-Hydroxy, β-Mercapto- and α-Amino tertiary Phosphine Oxides and Phosphine Sulphides. Tetrahedron Lett. 2001, 42, 457–460. 10.1016/S0040-4039(00)01952-3.

[ref325] Hatano M.; Miyamoto T.; Ishihara K. Enantioselective Addition of Organozinc Reagents to Aldehydes Catalyzed by 3,3′-Bis(diphenylphosphinoyl)-BINOL. Adv. Synth. Catal. 2005, 347, 1561–1568. 10.1002/adsc.200505221.

[ref326] Hatano M.; Miyamoto T.; Ishihara K. Enantioselective Dialkylzinc Addition to Aldehydes Catalyzed by Chiral Zn(II)-BINOLates Bearing Phosphonates and Phosphoramides in the 3,3′-Positions. Synlett 2006, 2005, 1762–1764. 10.1055/s-2006-944201.

[ref327] Hatano M.; Miyamoto T.; Ishihara K. 3,3′-Diphosphoryl-1,1’-bi-2-naphthol-Zn(II) Complexes as Conjugate Acid-Base Catalysts for Enantioselective Dialkylzinc Addition to Aldehydes. J. Org. Chem. 2006, 71, 6474–6484. 10.1021/jo060908t. 16901133

[ref328] Patel J. J.; Blackburn T.; Alessi M.; Sawinski H.; Snieckus V. Tetraethylphosphorodiamidate-Directed Metalation Group: Directed Ortho and Remote Metalation, Cross Coupling, and Remote Phospha Anionic Fries Rearrangement Reactions. Org. Lett. 2020, 22, 3860–3864. 10.1021/acs.orglett.0c01123. 32319779

[ref329] Volpe R.; Law H.Y.-L.; White J. M.; Flynn B. L. Selective Synthesis of C1-Symmetric BINOL-phosphates and P-chiral Phosphoramides Using Directed ortho-Lithiation. Org. Lett. 2021, 23, 7055–7058. 10.1021/acs.orglett.1c02430. 34448592

[ref330] Ratjen L.; van Gemmeren M.; Pesciaioli F.; List B. Towards High-Performance Lewis Acid Organocatalysis. Angew. Chem., Int. Ed. 2014, 53, 8765–8769. 10.1002/anie.201402765. 24802027

[ref331] Charmant J. P. H.; Dyke A. M.; Lloyd-Jones G. C. The Anionic Thia-Fries Rearrangement of Aryl Triflates. Chem. Commun. 2003, 380–381. 10.1039/b210648e. 12613622

[ref332] Kargbo R.; Takahashi Y.; Bhor S.; Cook G. R.; Lloyd-Jones G. C.; Shepperson I. R. Readily Accessible, Modular, and Tuneable BINOL 3,3′-Perfluoroalkylsulfones: Highly Efficient Catalysts for Enantioselective In-Mediated Imine Allylation. J. Am. Chem. Soc. 2007, 129, 3846–3847. 10.1021/ja070742t. 17352482

[ref333] Xiao B.; Fu Y.; Xu J.; Gong T. J.; Dai J. J.; Yi J.; Liu L. Pd(II)-Catalyzed C-H Activation/Aryl-Aryl Coupling of Phenol Esters. J. Am. Chem. Soc. 2010, 132, 468–469. 10.1021/ja909818n. 20020760

[ref334] Cong X.; You J.; Gao G.; Lan J. 2-Pyridylmethyl Ether: A Readily Removable and Efficient Directing Group for Amino Acid Ligand Accelerated ortho-C-H Olefination of Phenols. Chem. Commun. 2013, 49, 662–664. 10.1039/C2CC37291F. 23146996

[ref335] Yang J. F.; Wang R. H.; Wang Y. X.; Yao W. W.; Liu Q. S.; Ye M. Ligand-Accelerated Direct C-H Arylation of BINOL: A Rapid One-Step Synthesis of Racemic 3,3′-Diaryl BINOLs. Angew. Chem., Int. Ed. 2016, 55, 14116–14120. 10.1002/anie.201607893. 27726256

[ref336] Harada T.; Kanda K. Enantioselective Alkylation of Aldehydes Catalyzed by a Highly Active Titanium Complex of 3-Substituted Unsymmetric BINOL. Org. Lett. 2006, 8, 3817–3819. 10.1021/ol061407x. 16898825

[ref337] Harada T.; Ukon T. Practical and Highly Enantioselective Alkylation of Aldehydes Catalyzed by a Titanium Complex of 3-Aryl H_8_-BINOL. Tetrahedron: Asymmetry 2007, 18, 2499–2502. 10.1016/j.tetasy.2007.10.012.

[ref338] Yang X.-B.; Feng J.; Zhang J.; Wang N.; Wang L.; Liu J.-L.; Yu X.-Q. Highly Enantioselective Hetero-Diels-Alder Reaction of trans-1-Methoxy-2-methyl-3-trimethylsiloxybuta-1–3-diene with Aromatic and Aliphatic Aldehydes Catalyzed by 3-Substituted BINOL-Titanium Complex. Org. Lett. 2008, 10, 1299–1302. 10.1021/ol8001706. 18303907

[ref339] Gong T.-J.; Xiao B.; Liu Z.-F.; Wan J.; Xu J.; Luo D.-F.; Fu Y.; Liu L. Rhodium-Catalyzed Selective C-H Activation/Olefination of Phenol Carbamates. Org. Lett. 2011, 13, 3235–3237. 10.1021/ol201140q. 21604790

[ref340] Liu H.; Luo Y.; Zhang J.; Liu M.; Dong L. Rapid Synthesis of Alkenylated BINOL Derivatives via Rh(III)-Catalyzed C-H Bond Activation. Org. Lett. 2020, 22, 4648–4652. 10.1021/acs.orglett.0c01415. 32496793

[ref341] Tanaka J.; Nagashima Y.; Tanaka K. Rhodium(III)-Catalyzed Oxidative ortho-Olefination of Phenyl Carbamates with Alkenes: Elucidation of Acceleration Mechanisms by Using an Unsubstituted Cyclopentadienyl Ligand. Org. Lett. 2020, 22, 7181–7186. 10.1021/acs.orglett.0c02499. 32806145

[ref342] Liu H.; Lin M.-L.; Chen Y.-J.; Huang Y.-H.; Dong L. Rh(III)-Catalyzed One-Pot Three-Component Cyclization Reaction: Rapid Selective Synthesis of Monohydroxy Polycyclic BINOL Derivatives. Org. Chem. Front. 2021, 8, 4967–4973. 10.1039/D1QO00779C.

[ref343] Liu H.; Gong Z.-R.; Zhao Y.; Zheng J.; Xu Y.-J.; Dong L. Cascade Alkenylation/Intramolecular Friedel-Crafts Alkylation: High Selectivity at the C7-Position of BINOL. J. Org. Chem. 2023, 88, 6108–6119. 10.1021/acs.joc.3c00537. 37010424

[ref344] Bera S. S.; Maji M. S. Carbamates: A Directing Group for Selective C-H Amidation and Alkylation under Cp*Co(III) Catalysis. Org. Lett. 2020, 22, 2615–2620. 10.1021/acs.orglett.0c00589. 32207626

[ref345] Ahmed I.; Clark D. A. Rapid Synthesis of 3,3′ Bis-Arylated BINOL Derivatives Using a C-H Borylation in Situ Suzuki-Miyaura Coupling Sequence. Org. Lett. 2014, 16, 4332–4335. 10.1021/ol502126r. 25079395

[ref346] Boebel T. A.; Hartwig J. F. Silyl-Directed, Iridium-Catalyzed ortho-Borylation of Arenes. A One-Pot ortho-Borylation of Phenols, Arylamines, and Alkylarenes. J. Am. Chem. Soc. 2008, 130, 7534–7535. 10.1021/ja8015878. 18494474

[ref347] Bisht R.; Chaturvedi J.; Pandey G.; Chattopadhyay B. Double-Fold Ortho and Remote C-H Bond Activation/Borylation of BINOL: A Unified Strategy for Arylation of BINOL. Org. Lett. 2019, 21, 6476–6480. 10.1021/acs.orglett.9b02347. 31373495

[ref348] Chattopadhyay B.; Dannatt J. E.; Andujar-De Sanctis I. L.; Gore K. A.; Maleczka R. E. Jr; Singleton D. A.; Smith M. R. III Ir-Catalyzed ortho-Borylation of Phenols Directed by Substrate-Ligand Electrostatic Interactions: A Combined Experimental/in Silico Strategy for Optimizing Weak Interactions. J. Am. Chem. Soc. 2017, 139, 7864–7871. 10.1021/jacs.7b02232. 28453268 PMC5608269

